# Assessment of Germplasm Improvement in Three Farmed Grass Carp Populations Based on Genetic Variability

**DOI:** 10.3390/biology14030230

**Published:** 2025-02-25

**Authors:** Zhongyuan Shen, Liming Shao, Xixi Liu, Haiqi Li, Haipeng Guo, Lang Qin, Kaikun Luo, Wuhui Li, Jing Wang, Shengnan Li, Qianhong Gu, Liang Guo, Xu Huang, Qinbo Qin, Shaojun Liu

**Affiliations:** Engineering Research Center of Polyploid Fish Reproduction and Breeding of the State Education Ministry, College of Life Sciences, Hunan Normal University, Changsha 410081, China; zyshen@hunnu.edu.cn (Z.S.); slm@hunnu.edu.cn (L.S.); lxx_12192022@163.com (X.L.); liiihaiq@163.com (H.L.); 202470142964@hunnu.edu.cn (H.G.); qinlang9977@163.com (L.Q.); kaikunluo@163.com (K.L.); liwuhui11@163.com (W.L.); hwangjing0826@163.com (J.W.); shnli@hunu.edu.cn (S.L.); gqh@hunnu.edu.cn (Q.G.); hnsfgf@hunnu.edu.cn (L.G.); xiuhuang1993@163.com (X.H.)

**Keywords:** grass carp (*Ctenopharyngodon idella*), genetic improvement, genetic variability, gynogenesis, backcross breeding

## Abstract

The assessment of genetic improvement was comprehensively analyzed using the mtDNA *Cyt b* gene and SSR markers among three farmed grass carp populations caught in May 2024. The results of an mtDNA *Cyt b* gene analysis in 198 individuals showed that the haplotype diversity index (*H_i_*) and nucleotide diversity index (*P_i_*) were 0.555 and 0.00058, respectively. The results of the analysis of SSR marker data in 196 individuals indicated that the unequal dosage amplification at the same locus was found in the CC population. Moreover, the total number of alleles (*A*: 338), number of alleles per locus (*Na*: 15.36), observed heterozygosity (*Ho*: 0.8391), expected heterozygosity (*He*: 0.8380), and polymorphic information content (*PIC*: 0.8191) in the KC population was relatively higher than that in the CC (*A*: 129; *Na*: 5.86; *Ho*: 0.0025; *He*: 0.6191; *PIC*: 0.5747) and CY (*A*: 293; *Na*: 8.77; *Ho*: 0.821; *He*: 0.7483; and *PIC*: 0.5747) populations. The *FST* and AMOVA analysis showed the existence of a significant differentiation (*p* < 0.001), with a high genetic differentiation between the CC and CY populations. In summary, a high genetic variability exists in the KC population, while the father (CY) and mother (CC) populations have relatively low genetic variability. This study reveals evidence of the existence of a “micro-hybrid”. Moreover, the results demonstrated that combining both gynogenesis and backcross breeding technology is vital for the genetic improvement of grass carp. Moreover, continuous research into the genetic health of these populations is required as well as support for the protection of germplasm resources and artificial breeding.

## Simple Summary

In recent decades, grass carp (*Ctenopharyngodon idella*) has become an important freshwater aquaculture fish species following a breakthrough in key technologies for the artificial breeding of this species. However, people often neglect the selection and renewal of parent fish in the artificial breeding process, leading to the introduction of inferior traits into the breeding population of parent fish and causing the degeneration of the germplasm resources in grass carp. In this study, three germplasm resources of farmed grass carp were assessed based on genetic variability using the mitochondrial cytochrome b (mtDNA *Cyt b*) gene and nuclear microsatellite markers (simple sequence repeat, SSR). In the three cultured populations, one consists of gynogenetic grass carp which was produced to use the ultraviolet-irradiated sperm of koi carp (*Cyprinus carpio*) as a source of sperm stimulation (named the CC population), another comprises common grass carp (named the CY population), and the third is composed of a new disease-resistant grass carp which were gynogenetic grass carp mated with common grass carp. The results demonstrated that a “micro-hybrid” was found in the CC population, and there were differences in genetic variability among the three populations, especially between the CC and CY populations. These findings revealed that gynogenesis technology might cause a certain degree of reduction in the level of genetic variability in the directional selection and then purifies and fixes the maternal traits in grass carp populations. However, the genetic variability of the offspring can be improved using backcrossing technology.

## 1. Introduction

Grass carp (*Ctenopharyngodon idella*) is widely distributed in the Yangtze River, Pearl River, and Heilongjiang River systems in China, as well as its tributaries and affiliated reservoirs, ponds, and lakes [1]. With the breakthrough in key technologies for the artificial breeding of grass carp from 1958 to 1962, the supply of fish fry increased and became reliable [2]. Coupled with its fast growth rate, relatively low breeding costs, low consumer prices, and high protein quality, grass carp has been an important freshwater aquaculture fish species in China, providing people with a large amount of high-quality protein. In recent years, the yield of grass carp cultivation has ranked at the top of the list among the single species yields of freshwater aquaculture in China [3,4]. However, people often neglect the selection and renewal of parent fish in the artificial breeding process, leading to the introduction of inferior traits into the breeding population of parent fish and causing the degeneration of the germplasm resources in grass carp. In such cases, the main economic traits of offspring may worsen. Moreover, non-random inbreeding and adverse selection may also lead to genetic drift or genetic bottlenecks in the population of the artificial breeding grass carp, resulting in germplasm variation [5,6,7]. Therefore, the current germplasm status of the species has been always widely concerned. 

Fortunately, the development of molecular biology technology, such as gynogenesis and molecular marker-assisted selection, has greatly increased the genetic improvement of germplasm resources, especially for large-scale captive fishes, including rainbow trout (*Oncorhynchus mykiss*) [8,9,10], yellow catfish (*Pelteobagrus fulvidraco*) [11,12], Chinese tongue sole (*Cynoglossus semilaevis*) [13,14,15], crucian carp (*Carassius auratus*) [16,17,18], etc. In particular, the technique of gynogenesis has been widely used in fish breeding [19]. Gynogenesis technology is generally termed artificial gynogenesis, in which sperm triggers egg development, resulting in a diploid embryo using the maternal DNA almost exclusively. Artificial gynogenesis is achieved through techniques such as irradiating sperm using UV and suppressing the second meiotic division of the egg or inducing the egg to duplicate its DNA [20]. Gynogenesis technology boasts a rapid purification speed. The recognized outcome after 3–5 generations of selective breeding is a significant increase in homozygosity, approaching or reaching 100%. Through several consecutive generations of induction, artificial gynogenesis can stabilize gene loci at the chromosomes in heterozygous and homozygous forms, respectively, eliminating unfavorable genes and thereby forming a stable genetic lineage. Since the offspring of gynogenetically developed fish only contain the genetic information of the female parent, it allows for the rapid fixation of maternal traits, significantly enhancing the efficiency of the breeding process of fish, especially those with longer reproductive intervals. This provides an important means for the selection and breeding of improved fish varieties [21,22]. 

Many studies have been conducted on the population genetics of grass carp, which have mainly focused on natural populations and farmed populations for sale with the purpose of nature conservation and manual releasing assessment [23,24,25,26,27]. However, there are very few comprehensive evaluation studies on germplasm resources in grass carp parents. In order to produce an improved grass carp population, sexually mature individuals of the common grass carp (named the CY population) were selected as broodstock for aquaculture breeding. Then, the ultraviolet-irradiated sperm of koi carp (*Cyprinus carpio*) was used as a source of sperm stimulation to produce a new type of gynogenetic grass carp (named the CC population) using the CY population as the female parents in our laboratory. After the gynogenetic grass carp reached sexual maturity, we mated gynogenetic grass carp with common grass carp (backcross breeding) to produce a new strain of disease-resistant grass carp (named the KC population), which has been proven to have the advantages of fast growth and low incidence rate (especially bacterial diseases) [20,28,29,30]. What is the genetic variability of the three populations after germplasm improvement? It is pressing to conduct detailed accurate grass carp population monitoring, the including monitoring of its germplasm populations, to assess the population’s status and implement effective management and conservation. In particular, genetic diversity assessment plays a key role in some populations that have experienced significant fluctuation interference. For example, Christie et al. found that captive-born steelhead trout frequently show poor fitness when reintroduced into the wild under captive breeding programs [31]. The genetic diversity assessment showed that fish from families in which a limited number of parental individuals generate many offspring are more likely to mate with relatives, and subsequently exhibit lower per capita fitness due to inbreeding depression. In addition, da Paz Aguiar et al. [32] and de Sá Teles Oliveira et al. [33] found a higher level of genetic diversity (polymorphic ratio, heterozygosity, and number of alleles) in natural populations of tambaqui (*Colossoma macropomum* (Cuvier, 1816)) compared with farmed populations’ germplasms, suggesting genetic bottlenecks caused by confinement. Therefore, from the perspective of germplasm protection, it is important to assess the potential genetic problems that a species may experience and take suitable conservation measures. 

In this study, we assess the germplasm resources of farmed grass carp based on the genetic variability using the partial mitochondrial cytochrome b (mtDNA *Cyt b*) gene and 22 nuclear microsatellite markers (simple sequence repeat, SSR). Specifically, our study specified the following two objectives: (1) evaluate the genetic variability of three different grass carp lineage populations; (2) compare the genetic variability of three different grass carp populations to examine whether the combination of gynogenesis and backcross breeding technology can purify and rejuvenate these populations and achieve an obvious genetic improvement of grass carp germplasm. Our analyses will provide data upon which the future genetic improvement of grass carp might be based, as well as support for the protection of germplasm resources and artificial breeding.

## 2. Materials and Methods

### 2.1. Statement on Animal Subjects

All experimental protocols involving fish in this study were approved by the Ethics Committee for Animal Experiments of the College of Life Sciences, Hunan Normal University. The methods used in this study were conducted in accordance with the Laboratory Animal Management Principles of China.

### 2.2. Materials and Sampling

The individuals used in this research were collected from the breeding bases in Yueyang of Research Center of Polyploid Fish Reproduction and Breeding of the State Education Ministry in May 2024. One hundred and thirty disease-resistant grass carp juveniles that were artificially bred the previous year were randomly caught in pond A1 (named the KC population). Forty 6-year-old sexually mature gynogenetic grass carp were randomly caught in pond D4 (named the CC population, which was sourced from artificial breeding). Moreover, thirty-two 5-year-old sexually mature common grass carp were randomly caught in pond D12 (named the CY population, which was sourced from the Hunan aquatic original seed farm). In total, 202 grass carp individuals from three farmed grass carp populations were used for genetic analysis. All of the individuals’ muscle tissues were preserved in a 95% ethanol solution and stored in a refrigerator at −20 °C for analysis. 

### 2.3. PCR, Sequencing and Genotyping

We extracted the total genomic DNA from the muscle tissues of the specimens using a high-salt protocol with slight modifications [34]. The partial mitochondrial cytochrome b (mtDNA *Cyt b*) gene sequence was used to estimate genetic variability and genetic structure. To further understand the differences in genetic variability between the disease-resistant grass carp and their parent populations, 22 microsatellite markers published in studies on grass carp, as presented in the Supplementary Materials Table S1, were used to assist in the assessment of genetic variability. The partial mtDNA *Cyt b* primers L14724 (5′-GACTTGAAAAACCACCGTTG-3′) and H15915 (5′-CTCCGATCTCCGGATTACAAGAC-3′), universal for Cyprinidae, were selected for PCR amplification of grass carp DNA [35]. Polymerase chain reaction (PCR) was conducted using a Veriti™ 96-well thermal cycler and a Mastercycler nexus gradient PCR (Eppendorf). PCR was performed in volumes of 20 μL (SSR) or 30 μL (mtDNA *Cyt b*) containing 30–50 ng of template DNA, 0.5 μL of dNTP mixture (2.5 mmol/L each), 0.3 U of Taq DNA Polymerase with MgSO4 (2 mmol/L of Mg2+), 2 μL of 10×Taq Buffer, 0.5 μL of each primer (10 μmol/L), and deionized H2O. The PCR profile for the mtDNA *Cyt b* gene was as follows: initial denaturation at 94 °C for 3 min; followed by 35 cycles at 94 °C for 30 s, annealing temperature for 40 s, and 72 °C for 1 min; and then one cycle at 72 °C for 10 min. Each locus of SSR was amplified separately. The PCR profile for the SSR markers was initial denaturation at 94 °C for 4 min; followed by 30 cycles at 94 °C for 45 s, annealing temperature for 45 s, and 72 °C for 1 min; and then one cycle at 72 °C for 10 min. The PCR products were electrophoresed on 1.0% nondenaturing polyacrylamide gels and purified with a DNA Agarose Gel Extraction Kit (Omega, New Orleans, LA, USA). The PCR products for mtDNA sequencing and SSR markers were haplotyped and genotyped on an ABI 3730 automated genetic analyzer, respectively. Fragment sizing was confirmed via manual proofreading. Singletons were sequenced three times with the same primer.

### 2.4. Genetic Variability

The partial mtDNA *Cyt b* gene sequences were edited and assembled using the software package DNASTAR.Lasergene.v7.1 [36], and multiple alignments were performed using ClustalX 2.0 [37]. The aligned sequences were revised according to a published sequence (NCBI Reference Sequence: NC_010288.1) using manual correction with SEAVIEW v5 [38]. The base composition of the sequences was analyzed using MEGA v6.0 [39]. The number of haplotypes (*H*), haplotype diversity (*H_i_*), nucleotide diversity (*P_i_*), and average number of nucleotide differences (*k*) was estimated in DNASP v5.10 [40].

The software package Micro-Checker [41] was used to check all microsatellite data in order to exclude any null alleles and large allelic dropouts. Subsequently, the total number of alleles (*A*), number of alleles per locus (Na), observed heterozygosity (*H_o_*), unbiased expected heterozygosity (*H_e_*), and inbreeding coefficient (*FIS*), as well as the polymorphic information content (*PIC*), were computed using Cervus v3.03 [42].

### 2.5. Genetic Structure

The neighbor-joining (NJ) tree of the partial mtDNA *Cyt b* gene sequences was constructed using Kimura’s 2-Parameter model in MEGA v6.0 [39], with the sampling times being 1000. Arlequin 3.5.1.2 software [43] was used to conduct Exact Tests of population differentiation [44] to estimate the *FST* values as a measure of genetic differentiation among the three populations and to assess the statistical significance of these *FST* values through 1000 permutations. 

An analysis of molecular variance (AMOVA) was also performed to estimate genetic structure indices in the population using information about haplotypes, as well as their frequencies using Arlequin 3.5.1.2 software [43]. The information on the differences between haplotypes was entered as a matrix of Euclidean squared distances. The significance of the covariance components associated with the different possible levels of genetic structure (within individuals, within populations, within groups of populations, and among groups) was tested using non-parametric permutation procedures in this software. In addition, the Nei’s genetic distance based on SSR data was calculated using POPGEN 3.2 [45], and the UPGMA phylogenetic tree was reconstructed based on the genetic distance using MEGA v6.0 [39] as an assistant tree.

## 3. Results

### 3.1. Sequence Variation and Microsatellite Genetic Characteristics

#### 3.1.1. Sequence Composition and Variation in the Partial *Cyt b* Gene

A total of 198 sequences were obtained from 202 grass carp that were sequenced, including 126 disease-resistant grass carp sequences, 40 gynogenetic grass carp sequences, and 32 common grass carp sequences. After aligning and revising the sequences of the mtDNA *Cyt b* gene, we obtained 198 sequences with a length of 1190 bp. Sequence analysis showed that the average base compositions of this gene in disease-resistant grass carp and common grass carp were the same, with the following results: A = 29.8%, T = 28.5%, C = 27.9%, and G = 13.8%. The average base composition of this gene in gynogenetic grass carp was as follows: A = 29.7%, T = 28.5%, C = 27.9%, and G = 13.9%. The A + T content was slightly higher than that of G+C in all populations. A total of 1184 invariable or monomorphic sites and 6 variable or polymorphic sites were found in the sequences. Among these variable or polymorphic sites, there were three singleton variable sites (site positions: 1,2,51) and three parsimony informative sites (site positions: 593,743,1072).

#### 3.1.2. Characteristics of Microsatellite Markers

**Figure 1:**
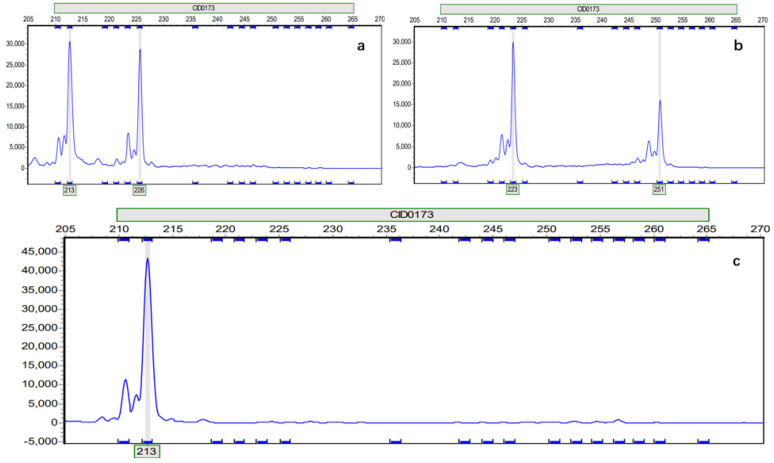
The original peak graph of three genotype types in gynogenetic grass carp at microsatellite locus CID0173. Note: (**a**): heterozygous type with isodose amplification; (**b**): heterozygous type with unequal dosage amplification; (**c**): homozygous type with isodose amplification.

Using automated fluorescent sequencing, all the disease-resistant grass carp and common grass carp were successfully genotyped with microsatellite markers, and 34 out of 40 gynogenetic grass carp were successful (Tables S2–S5). The microsatellite data showed that the genetic characteristics in common grass carp and disease-resistant grass carp were consistent with the hereditary law of fish, while that in the gynogenetic grass carp showed differences. Among the CC population, there were three types of amplification on the original peak graph, including two isodose amplification types (ratio about 1:1; Figure 1a: heterozygous type; Figure 1c: homozygous type) and one type of unequal dosage amplification with small amounts (ratio ≠ 1:1; Figure 1b). In addition, the isodose and unequal dosage amplification could be found at the same locus. The phenomena were found at other loci.

### 3.2. Genetic Diversity Analysis

#### 3.2.1. Genetic Diversity of the Partial *Cyt b* Gene

For the partial *Cyt b* gene sequences, only a total of seven different haplotypes were detected among 198 grass carp from the KC, CC, and CY populations. There were common haplotypes in all populations, with one unique haplotype in the CY population, three unique haplotypes in the KC population, and the other three haplotypes being shared by all populations (Table 1). 

A comparison of the sequences revealed that the number of haplotypes (*H*) ranged from three to six in the three populations. The overall haplotype diversity index (*H_i_*), the nucleotide diversity index (*P_i_*), and the average number of nucleotide differences (*k*) of the grass carp populations were 0.555, 0.00058, and 0.649, respectively. Moreover, all of the populations exhibited different genetic diversities from each other. The *H_i_*, *P_i_*, and *k* values of the KC population were the highest, at 0.477, 0.00049, and 0.533. The three indices of the CY population were the lowest (Table 2).

#### 3.2.2. Genetic Diversity of SSR

For microsatellite DNA data, there was no evidence of null alleles and large allelic dropout among the grass carp. A total of 373 alleles were detected in the 196 accessions using 22 pairs of markers. The number of alleles per locus varied from 9 (for primer CID0347) to 29 (for primer CID1533), with an average of 16.76 per locus (Supplementary Materials Table S2; Table 3). The total number of alleles (*A*: 338), number of alleles per locus (*Na*: 15.36), observed heterozygosity (*Ho*: 0.8391), expected heterozygosity (*He*: 0.8380), and polymorphic information content (*PIC*: 0.8191) in the KC population which had a larger size was higher than that in the CC and CY populations (Table 3). 

The detailed genetic diversity parameters of 22 microsatellite loci in all populations of grass carp are presented in the Supplementary Materials Tables S2–S5. The inbreeding coefficients (*FIS*) of the markers were relatively low, ranging from −0.0.0613 (CID0012) to 0.0930 (Ci03) in the KC population, −0.1939 (CID0347) to 0.2092 (Ci03) in the CY population, and 0.5698 (CID0869) to −1.0000 (CID0012, CID1533, CID0002, CID0382, CID0474, CID0347, CID1512, HLJC107, and CID0909) in the CC population. The detailed genetic diversity parameter analysis resulted in a somewhat excess of heterozygous individuals in the CY population, and an excess of homozygotes was evident in individuals in the CC population. Additionally, the CY and CC populations did not possess any rare alleles. However, a large number of rare alleles were found in the KC population. 

### 3.3. Genetic Structure Analysis

**Figure 2:**
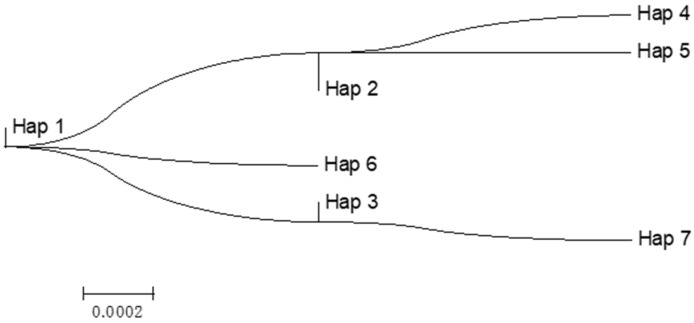
The topological structure among haplotypes based on the mtDNA data using the NJ tree of relatedness based on the distance matrix.

We calculated the pairwise distances among haplotypes based on the partial mtDNA data using the NJ tree of relatedness based on the distance matrix. As illustrated by the topological structure (Figure 2), the haplotype Hap1, which exhibits the highest frequency of individuals, is positioned at the root of the tree. From Hap1, three evolutionary branches emerge: Hap2, Hap3, and Hap6. Specifically, Hap2 subsequently diverges into Hap4 and Hap5, whereas Hap3 gives rise to Hap7. In addition, a UPGMA analysis of SSR data based on Nei’s genetic distance was also performed (Supplementary Materials, Figure S1). The trees yielded good agreement regarding topological relationships. 

Overall, the pairwise *FST* values based on the mitochondrial *Cyt b* gene among the three populations of grass carp are presented in Table 4. The results showed that there was significant genetic differentiation between any pair of the three populations. The FST values between the CC and CY populations in grass carp were as high as 0.543.

To examine the genetic differentiation between the offspring and its parents (especially the fathers of the common grass carp), the grass carp populations were divided into two groups: one group consisting of the CY and KC populations and the other group consisting of the CC population. The AMOVA analysis of molecular variance showed that the percentage of variation within populations accounted for 81.41%, while the percentage of variation between groups and within groups accounted for −44.59% and 63.18%, respectively (Table 5). The variations within the population and between groups within the population were the main sources of total variation, which was highly significant at the *p* < 0.01 level. 

## 4. Discussion

Molecular markers have been proven to be a powerful tool for assessing genetic variation and structure among populations of the grass carp species, which can reveal genetic differences at the DNA level in the absence of environmental effects [46,47]. Moreover, it is effective to evaluate the genetic variability of germplasm in breeding programs [20]. The evaluation of the genetic variability of grass carp germplasm is of critical importance for the efficient exploitation of valuable genetic resources present in both the landrace and diverse lineages during grass carp improvement. Previous studies on the evaluation of grass carp germplasm resources were based on simple descriptions and comparisons, often directly utilizing genetic parameters in some farmed and natural populations [22,23,24,25,26,27]. In this study, we evaluate the genetic diversity of grass carp and examine whether it can be used to achieve a significant genetic improvement of grass carp germplasm using the combination of gynogenesis and backcross breeding technology.

In recent years, the partial mitochondrial DNA *Cyt b* gene, with its moderate evolutionary rate and relatively high mutation rate, has been widely used in the study of population differentiation and phylogeny of fish and other animals as an excellent marker to explore interspecific and intraspecific inheritance and differentiation [48]. The results of the partial mtDNA *Cyt b* gene in our study showed that the average base compositions of this gene in the KC and CY populations were the same (A = 29.8%, T = 28.5%, C = 27.9%, and G = 13.8%), with a slight difference in the CC population (A = 29.7%, T = 28.5%, C = 27.9%, and G = 13.9%). Similar to the mtDNA sequences of vertebrates [49], the A+T content was significantly higher than that of G + C in all populations, which indicated that there is genetic stability and reduced mutation rates.

Microsatellite markers, also known as simple sequence repeat (SSR) or short tandem repeat (STR) markers, are widely used in the assessment of population genetic variability, control of inbreeding, and molecular marker-assisted breeding of aquatic animals due to their characteristics of wide distribution, high polymorphism, easy detection, and co-dominant inheritance [50]. Now especially, with the development of technology, accurate typing of microsatellite loci can be achieved through sequencing methods. Our study showed that six gynogenesis individuals were not successfully genotyped on microsatellite markers using automated fluorescent sequencing. Moreover, there were three types of amplification on the original peak graph, including two isodose amplification types (a ratio of about 1:1; Figure 1a: heterozygous type and Figure 1c: homozygous type) and one type of unequal dosage amplification with small amounts (ratio ≠ 1:1; Figure 1b) in the CC population. Several possible mechanisms could account for this phenomenon. The CC population is produced using the ultraviolet-irradiated sperm of koi carp as a source of sperm stimulation. In offspring resulting from gynogenetic stimulation with heterologous sperm, paternal DNA fragments may be inserted, as shown from some data in another study performed in our laboratory [28]; this was also found to be the case in gynogenetic silver crucian carp [51], gynogenesis Pengze crucian carp [52], gynogenesis red crucian carp [53], gynogenesis Japanese crucian carp [54], common carp [55], blunt snout bream [56], largemouth bass [57], mandarin fish [58], and gynogenetic oblique-banded grouper [59]. The breeding process of gynogenetic grass carp involves meiosis. The first meiotic division in oocytes involves recombination events between homologous chromosomes, wherein the recombination rate exhibits positional dependency along the chromosomal axis. Specifically, loci positioned at greater distances from the centromere demonstrate proportionally elevated recombination frequencies compared to those situated in proximal regions. If the mother of a gynogenetic carp is heterozygous and the loci are situated at greater distances from the centromere, this potentially results in some loci being heterozygous because of recombination events. Otherwise, the inserted paternal DNA fragments may also break the simple sequence repeat, thus causing some gynogenesis individuals not to be successfully genotyped or an unequal dosage amplification. Moreover, Liu et al. proposed that the concept of “micro-hybrid” refers to offspring, including autodiploid and autotetraploid lineages, as well as those resulting from artificial gynogenesis, whose genome almost originates solely from the maternal parent but in which certain DNA fragments derived from the paternal parent are inserted [20]. The amplification of heterozygous genotype types occurs in small amounts in our study, which proves the existence of a “micro-hybrid” from another point of view. However, the mutations in flanking regions or mutations in tandem repeats of SSR may also be one of the reasons for this phenomenon.

Gynogenesis technology is considered to be able to rapidly achieve the fixation of maternal traits, significantly enhance the breeding progress and efficiency of fish, especially those with longer generation cycles, and provide an important means for the selection and breeding of improved fish varieties. This is even more valuable for subsequent artificial breeding efforts [20]. Comparing the genetic diversity of the CC and CY populations, we found that the CC population did not possess any unique haplotype, and the *A*, *Na*, *Ho*, and *PIC* values were lower than those of the CY population, which is consistent with results reported by Zhu et al. [60], Xie et al. [61], Zhang et al. [62], and Lu et al. [63]. Liu et al. [20] show that artificial gynogenesis undergoes cold shock or heat shock processes, in which fish that survive these adverse conditions have undergone a “rigorous breeding” process. This “selection effect” may eventually lead to the loss of some alleles during gynogenesis, while some rare alleles related to resistance undergo directional selection. The effect of “strict screening” or “rigorous breeding” causes a certain degree of reduction in the level of genetic diversity in the directional selection and then purifies and fixes the maternal traits in grass carp populations. The somewhat excess of heterozygous individuals in the CY population and the excess of homozygotes evident in individuals in the CC population also support Liu et al.’s view [20].

The genetic diversity of a species is a product of its long-term evolution and serves as a prerequisite for its survival adaptation and evolutionary development [64]. The higher the genetic diversity of a species, the stronger its adaptability to environmental changes, and vice versa, leading to a decrease in the adaptability of the population. In our study, all of the populations exhibited different genetic diversity between each other, both in the mtDNA *Cyt b* gene and SSR markers. The parameters of the genetic diversity of the KC population were the highest (*H_i_*: 0.477, *P_i_*: 0.00049, and *k*: 0.533; *A*: 338, *Na*: 15.36, *Ho*: 0.8391, *He*: 0.8380, and *PIC*: 0.8191), which were higher than that of the CY and CC populations, as presented in Table 2 and Table 3, suggesting that the genetic improvement of grass carp germplasm can be achieved using the combination of gynogenesis and backcross breeding technology. Additionally, a large number of rare alleles were found in the KC population, with no rare alleles found in the CY and CC populations. Schlotterer [65] believes that the polymorphism of microsatellites is related to the evolutionary pressure borne by species. Microsatellite sequences located in different positions in the chromosomes bear different pressures, resulting in differences in the number of alleles and the degree of variation. Although the number of offspring samples is greater than that of the parents, the greater pressure on the parents (especially the CC population via the effect of “strict screening” or “rigorous breeding”) may be the reason why the KC population has a large number of rare alleles in the microsatellites. The ability of a population to evolve and adapt may be related to both heterozygosity (i.e., the proportion of diploid individuals that have two different alleles at a single locus) and the total number of alleles present within a population [66,67,68]. The higher genetic diversity and more alleles in the KC population produced by the combination of gynogenesis and backcross breeding technology indicate that KC grass carp may have a stronger ability to adapt. The value of *Na* reported by Yu et al. (ranged from 12.467 to 14.400) in different generations of a breeding population of grass carp [69] was lower than that of the KC population, implying that the breeding technique with the combination of gynogenesis and backcross breeding technology could be used as an alternative to the conventional selective breeding and unplanned artificial breeding in fish, which were reported in other fish species [10,12,15,69]. Conversely, Stéphanie Manel et al. [70] reported that freshwater fish genetic diversity was mainly associated with the region and average slope of river basins. Our research reveals that artificial genetic improvement may also be responsible for the change in and enhancement of the adaptability of freshwater fish. In addition, microsatellites are considered for neutral markers mostly, but sometimes they can be linked with genes that could affect them [63], which may also be the reason for the genetic diversity of the three populations presented.

As illustrated by the topological structure (Figure 2), the haplotype Hap1, which exhibits the highest frequency of individuals, is positioned at the root of the tree, inferring that it is a relatively primitive haplotype with strong adaptability to the external environment and can stably exist in the grass carp population. The results of the distribution of the haplotypes showed there may be a relatively close genetic relationship among three grass carp populations. In our study, a total of 198 sequences of the mt DNA *Cyt b* gene were obtained from 202 sequenced grass carp and a total of seven different haplotypes were detected. However, Hap4 disappeared, while Hap5 and Hap6 appeared in the KC population, probably as a result of the process of artificial gynogenesis and backcross breeding based on the mtDNA *Cyt b* gene in 198 grass carp, especially considering the cold shock or heat shock processes in artificial gynogenesis. The MtDNA follows strict maternal inheritance and generally does not undergo recombination. Offspring can fully preserve maternal genetic information, and one haplotype of mtDNA can represent a maternal lineage [71]. This means that there were not many maternal lines in our study.

Chen et al. [72] described the difference in genetic diversity among populations as an effective indicator of genetic differentiation. Although there were fewer maternal lines in this study, our results show that diversity distribution apparently varied among populations with regard to genetic diversity and *PIC* scores (Table 3, Supplementary Materials Tables S2–S5), suggesting the distinct levels of differentiation in these three populations. Further, the significant genetic differentiation (*p* < 0.001) among the three populations was illustrated by the AMOVA analysis (Table 4), which confirmed the differentiation of three populations in this germplasm resource. Moreover, the variation within the population and between groups within the population were the main sources of the total variation, which was highly significant at the *p* < 0.01 level. This result was not consistent with that (the genetic differentiation mainly came from between individuals, with small genetic differences between populations) reported by Zhu et al. [53]. Meanwhile, the results of the pairwise *FST* values based on the mitochondrial *Cyt b* gene among the three populations of grass carp in our study showed that there was different genetic differentiation in any pair of the three populations. Moreover, the value of *FST* is as high as 0.543, indicating that there was a high genetic differentiation between the CC and CY populations. The results of this study revealed that the combination of gynogenesis and backcross breeding technology can achieve the genetic improvement of grass carp germplasm.

## 5. Conclusions

This study provides data upon which the future genetic improvement of grass carp might be based, as well as support for the protection of germplasm resources and artificial breeding. In the future, the development of molecular biology technology, such as gynogenesis and molecular marker-assisted selection, can be considered to achieve the genetic improvement of germplasm resources. Meanwhile, the high-precision whole genome sequencing of the population of gynogenesis grass carp is an important means for comprehensively studying the existence of “micro-hybrids”, in combination with gynogenesis and backcross breeding technology in the future. Additionally, the epigenome and microbiome should be studied should be studied to explain the adaptive variation in those populations with low genetic diversity during farming.

## Figures and Tables

**Table 1:** Distribution of the haplotypes among the three grass carp populations.

Haplotype	Population	No. of Individuals
Hap1	CC/CY/KC	121
Hap2	CC/CY/KC	50
Hap3	CC/CY/KC	22
Hap4	CY	1
Hap5	KC	1
Hap6	KC	2
Hap7	KC	1

**Table 2:** Summary of the population genetic diversity in the three grass carp populations based on the mtDNA *Cyt b* gene data.

Population	*N*	*H*	*H_i_*	*P_i_*	*k*
CY	32	4	0.333	0.00035	0.397
CC	40	3	0.412	0.00039	0.438
KC	126	6	0.477	0.00049	0.553
Total	198	7	0.555	0.00058	0.649

*N*: the number of sampled grass carp; *H*: the number of *Cyt b* haplotypes; *H_i_*: haplotype diversity; *P_i_*: nucleotide diversity; and *k*: the average number of nucleotide differences.

**Table 3:** Summary of genetic diversity parameters of three grass carp populations based on 22 microsatellite loci.

Population	*N*	*A*	*N_a_*	*H_o_*	*H_e_*	*PIC*	*FIS*
CY	32	293	8.77	0.8211	0.7483	0.6993	−0.0546
CC	34	129	5.86	0.0025	0.6191	0.5747	0.9289
KC	130	338	15.36	0.8391	0.8380	0.8191	−0.0051
Total	196	373	16.76	0.6946	0.8445	0.8259	0.0968

*N*: the number of sampled individuals; *A*: the number of alleles; *N_a_*: the average number of alleles per locus; *H_o_*: observed heterozygosity; *H_e_*: expected heterozygosity; *FIS*: inbreeding coefficients calculated as 1 − (*H_o_*/*H_e_*); and *PIC*: polymorphism information content.

**Table 4:** The genetic differentiation index FST values among different populations of grass carp.

Population	CY	CC	KC
CY		0.00	0.00
CC	0.543		0.00
KC	0.428	0.011	

Note: The bottom diagonal indicates the genetic differentiation index *FST* values; the top diagonal stands for the corresponding *p* value.

**Table 5:** AMOVA analysis of molecular variance based on the *Cyt b* gene.

Source of Variation	d.f.	Sum of Squares	Variance Components	Percentage of Variation	Fixation Indices	*p*
Among groups	1	1.901	−0.14338Va	−44.59	FCT = −0.44592	0.67155
Among population within groups	1	10.870	0.20316Vb	63.18	FSC = 0.43697	0.00000
Within populations	196	50.782	0.26178Vc	81.41	FST = 0.18591	0.0000
Total	198	63.553	0.32154			

Note: d.f.: degree of freedom.

## Data Availability

The *Cyt b* gene and microsatellite data of grass carp can be obtained by contacting the author (zyshen@hunnu.edu.cn).

## Supplementary Materials

The following supporting information can be downloaded at https://www.mdpi.com/article/10.3390/biology14030230/s1: Figure S1: The UPGMA tree of SSR data based on Nei’s genetic distance; Table S1: The primer information used in this study; Table S2: Summary of genetic diversity parameters of all grass carp based on 22 microsatellite loci; Table S3: Summary of genetic diversity parameters of common grass carp based on 22 microsatellite loci; Table S4: Summary of genetic diversity parameters of gynogenetic grass carp based on 22 microsatellite loci; and Table S5: Summary of genetic diversity parameters of disease-resistant grass carp based on 22 microsatellite loci. Allele Report.
