# Supplementary material for: Assessment of Germplasm Improvement in Three Farmed Grass Carp Populations Based on Genetic Variability
Source: Biology (Basel). 2025 Feb 25;14(3):230. doi: 10.3390/biology14030230 (PMC11939604; doi:10.3390/biology14030230)
Supplement: Supplementary file 1 [file biology-14-00230-s001.zip › CID0173.pdf]

Allele Report

Project Comments:

Sample 1: CID0173_EST1573_CC10_C03.fsa Run date and time: 09/21/2024 - 03:00:19 -> 09/21/2024 - 03:27:31

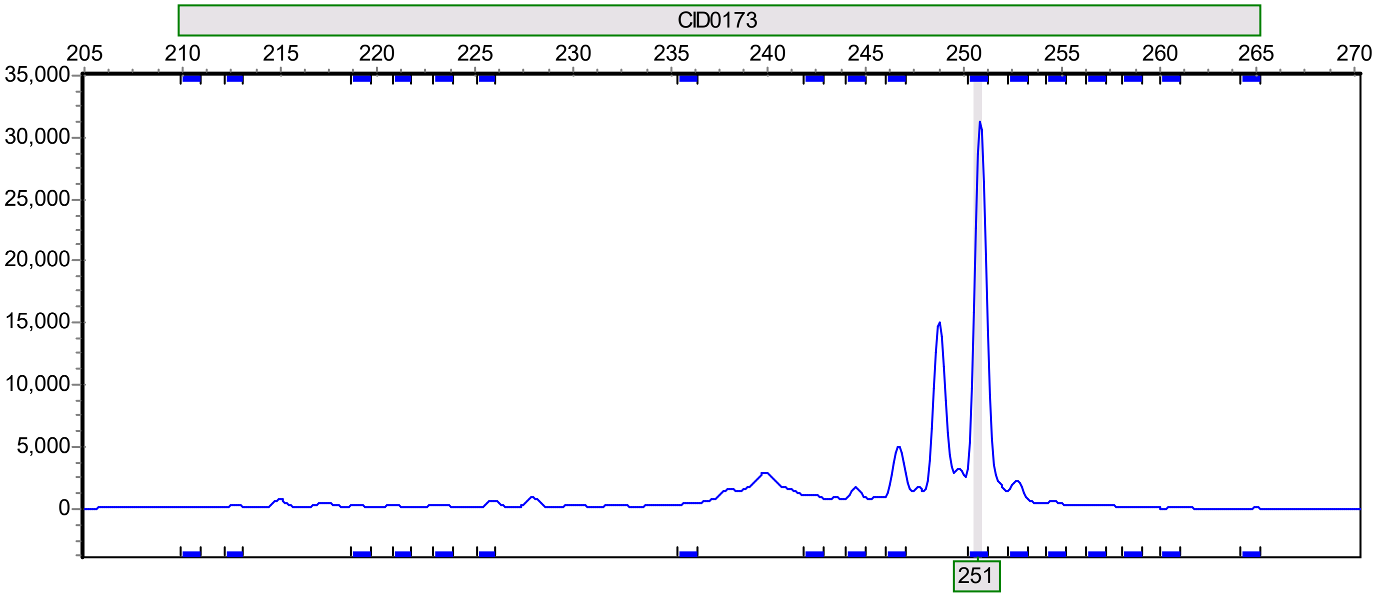

No	Size	Height	Area	Marker	Allele	Difference	Quality	Score	Allele Comments	Sample Comments
1	170.6	35484	284720	EST1573	171	0.00	Pass	500.0	[<SAT (Repaired)><Confirmed>]	
2	250.8	31275	212416	CID0173	251	0.00	Pass	500.0	[<Confirmed>]	

Sample 2: CID0173_EST1573_CC11_E03.fsa Run date and time: 09/21/2024 - 03:00:19 -> 09/21/2024 - 03:27:31

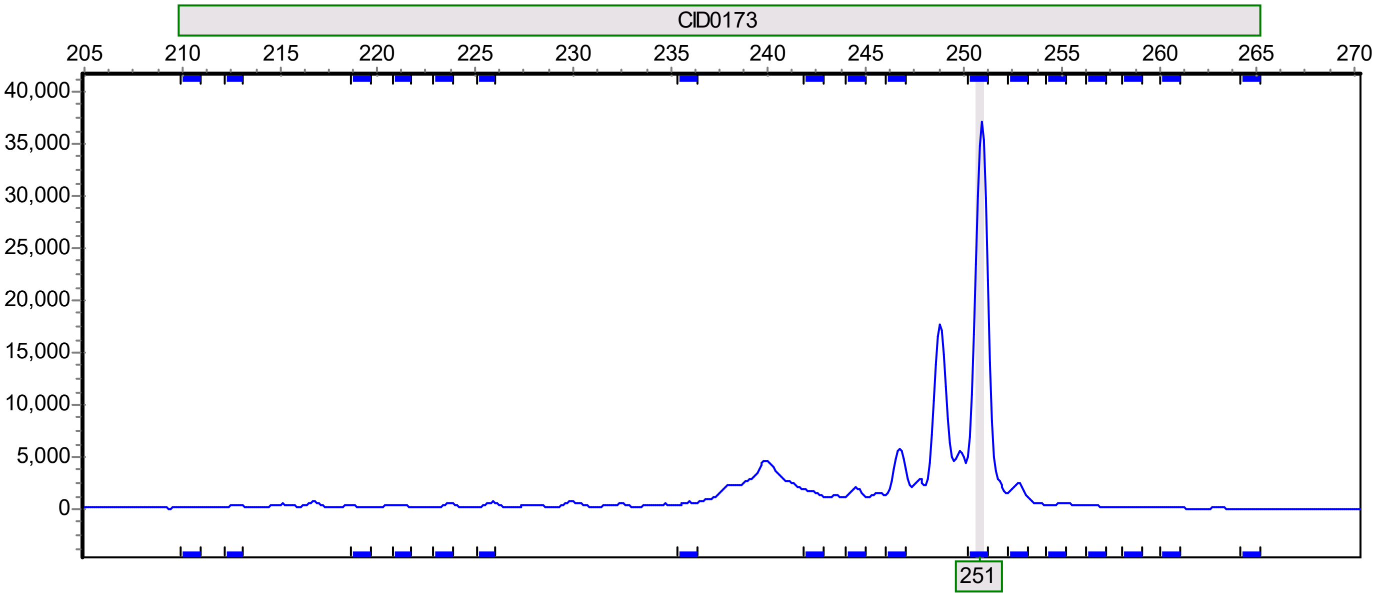

No	Size	Height	Area	Marker	Allele	Difference	Quality	Score	Allele Comments	Sample Comments
1	170.9	41447	348301	EST1573	171	0.30	Pass	500.0	[<SAT (Repaired)><Confirmed>]	
2	250.9	37108	263194	CID0173	251	0.10	Pass	500.0	[<SAT (Repaired)><Confirmed>]	

Sample 3: CID0173_EST1573_CC12_G03.fsa Run date and time: 09/21/2024 - 03:00:19 -> 09/21/2024 - 03:27:31

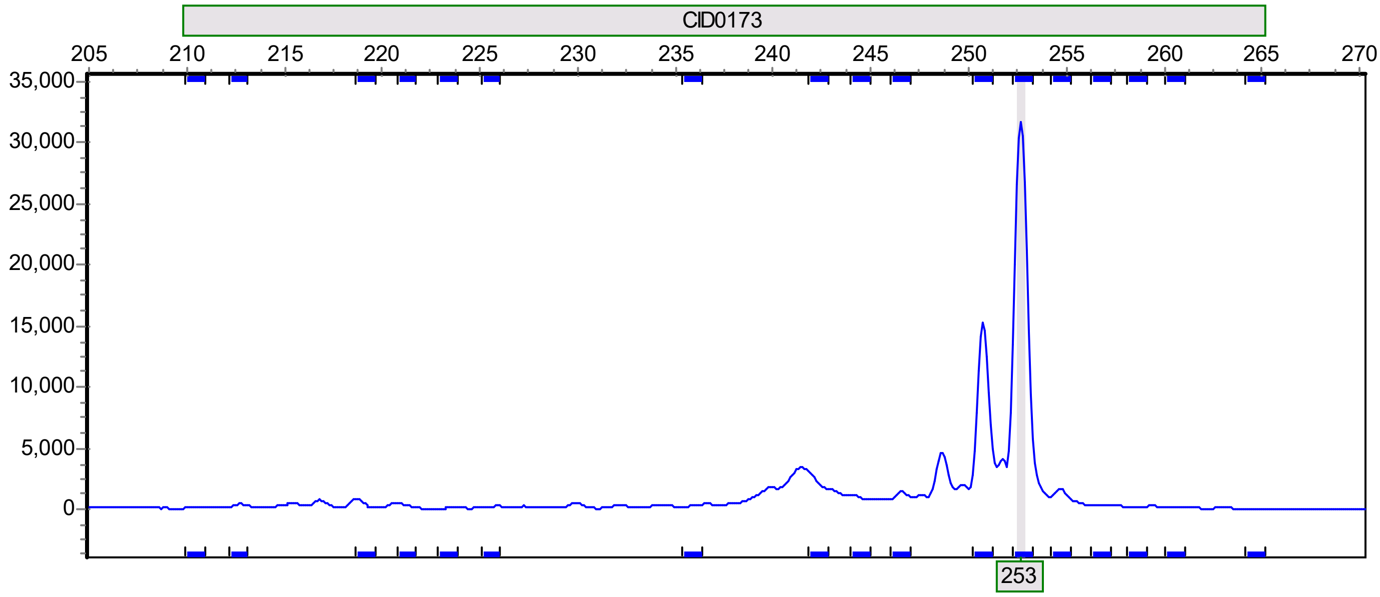

No	Size	Height	Area	Marker	Allele	Difference	Quality	Score	Allele Comments	Sample Comments
1	170.6	35560	286321	EST1573	171	0.00	Pass	500.0	[<SAT (Repaired)><Confirmed>]	
2	252.7	31703	233518	CID0173	253	0.10	Pass	500.0	[<Confirmed>]	

Sample 4: CID0173_EST1573_CC13_I03.fsa Run date and time: 09/21/2024 - 03:00:19 -> 09/21/2024 - 03:27:31

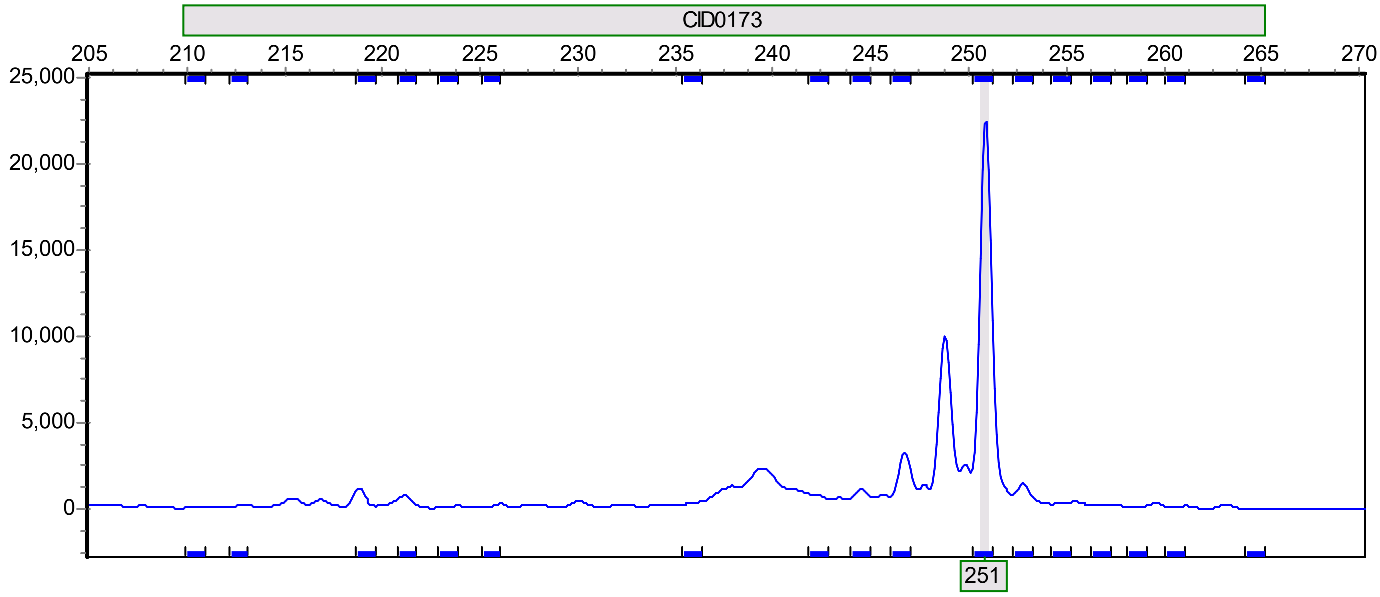

No	Size	Height	Area	Marker	Allele	Difference	Quality	Score	Allele Comments	Sample Comments
1	170.5	26571	202345	EST1573	171	0.10	Pass	500.0	[<SAT (Repaired)><Confirmed>]	
2	250.9	22436	148480	CID0173	251	0.10	Pass	500.0		

Sample 5: CID0173_EST1573_CC14_K03.fsa Run date and time: 09/21/2024 - 03:00:19 -> 09/21/2024 - 03:27:31

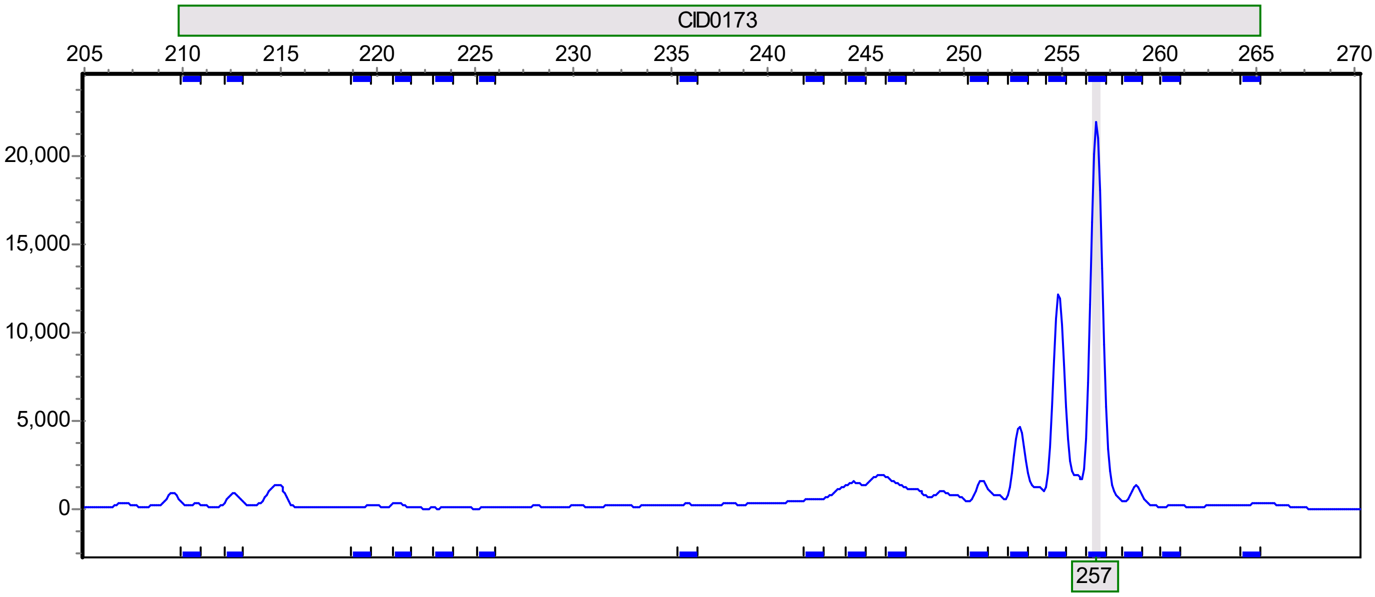

No	Size	Height	Area	Marker	Allele	Difference	Quality	Score	Allele Comments	Sample Comments
1	174.5	30744	216546	EST1573	174	0.10	Pass	500.0	[<Confirmed>]	
2	256.8	21978	146866	CID0173	257	0.00	Pass	500.0	[<Confirmed>]	

Sample 6: CID0173_EST1573_CC15_M03.fsa Run date and time: 09/21/2024 - 03:00:19 -> 09/21/2024 - 03:27:31

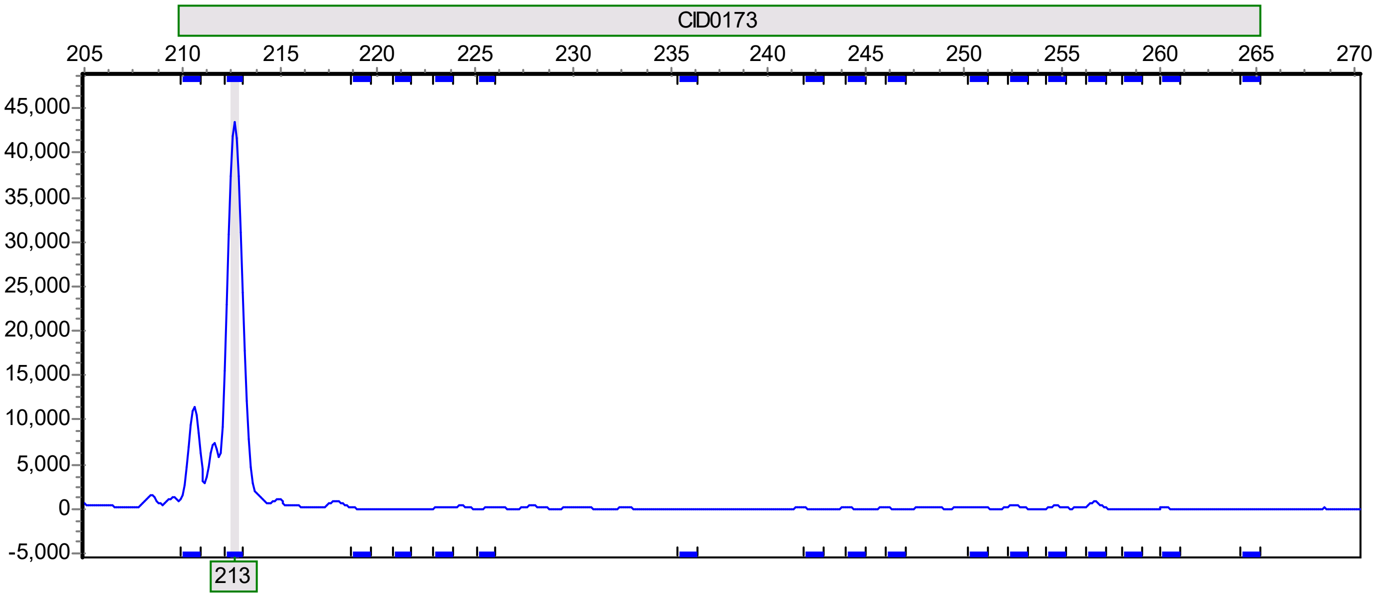

No	Size	Height	Area	Marker	Allele	Difference	Quality	Score	Allele Comments	Sample Comments
1	161.6	12919	92540	EST1573	161	0.30	Pass	500.0	[<Deleted>]	
2	170.6	35295	274591	EST1573	171	0.00	Pass	500.0	[<SAT (Repaired)><Confirmed>]	
3	201.7	13559	116935	CID0173	202	0.10	Pass	500.0	[<Deleted>]	
4	212.7	43471	364804	CID0173	213	0.00	Pass	500.0	[<SAT (Repaired)><Confirmed>]	

Sample 7: CID0173_EST1573_CC16_O03.fsa Run date and time: 09/21/2024 - 03:00:19 -> 09/21/2024 - 03:27:31

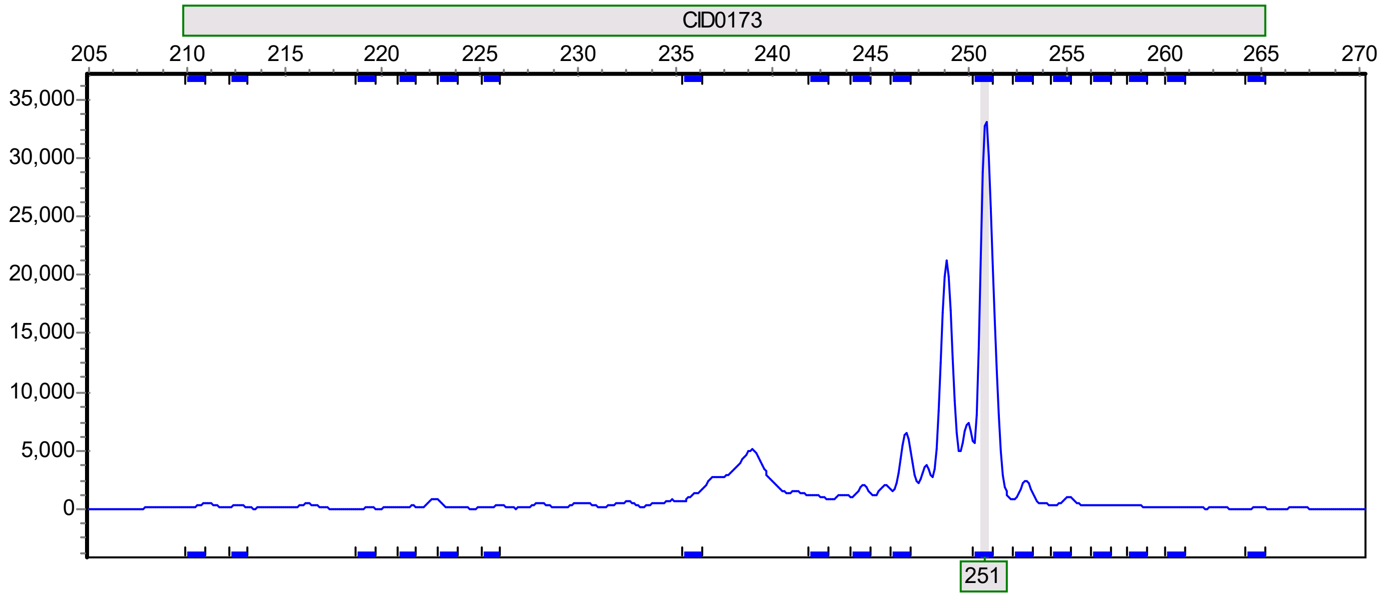

No	Size	Height	Area	Marker	Allele	Difference	Quality	Score	Allele Comments	Sample Comments
1	161.4	22254	163117	EST1573	161	0.10	Pass	500.0	[<Deleted>]	
2	170.7	30534	254192	EST1573	171	0.10	Pass	500.0	[<SAT (Repaired)><Confirmed>]	
3	250.9	33079	250363	CID0173	251	0.10	Pass	500.0	[<SAT (Repaired)><Confirmed>]	

Sample 8: CID0173_EST1573_CC17_A05.fsa Run date and time: 09/21/2024 - 03:00:19 -> 09/21/2024 - 03:27:31

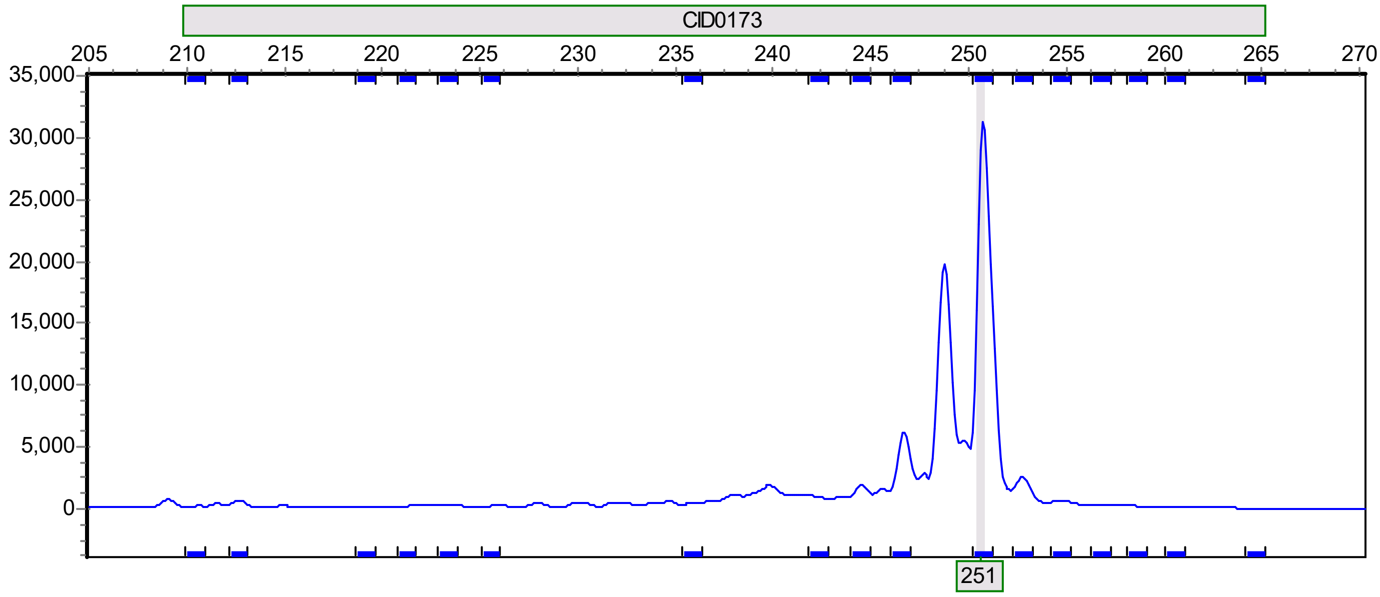

No	Size	Height	Area	Marker	Allele	Difference	Quality	Score	Allele Comments	Sample Comments
1	172.6	47981	393426	EST1573	173	0.10	Pass	500.0	[<SAT (Repaired)><Confirmed>]	
2	250.7	31304	255612	CID0173	251	0.10	Pass	500.0	[<SAT (Repaired)><Confirmed>]	

Allele Report

Sample 9: CID0173_EST1573_CC18_C05.fsa Run date and time: 09/21/2024 - 03:00:19 -> 09/21/2024 - 03:27:31

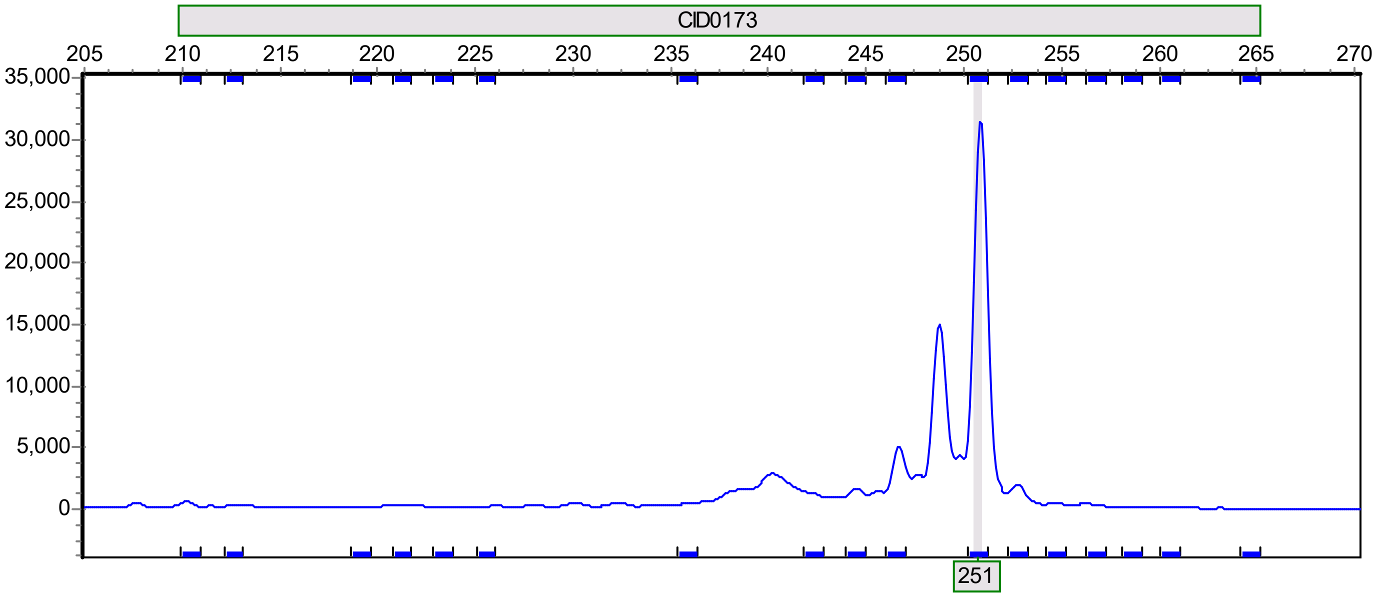

No	Size	Height	Area	Marker	Allele	Difference	Quality	Score	Allele Comments	Sample Comments
1	162.3	13110	99192	EST1573	163	0.60	Pass	500.0	[<Deleted>]	
2	170.8	41431	342562	EST1573	171	0.20	Pass	500.0	[<SAT (Repaired)><Confirmed>]	
3	250.8	31441	246479	CID0173	251	0.00	Pass	500.0	[<Confirmed>]	

Sample 10: CID0173_EST1573_CC19_E05.fsa Run date and time: 09/21/2024 - 03:00:19 -> 09/21/2024 - 03:27:31

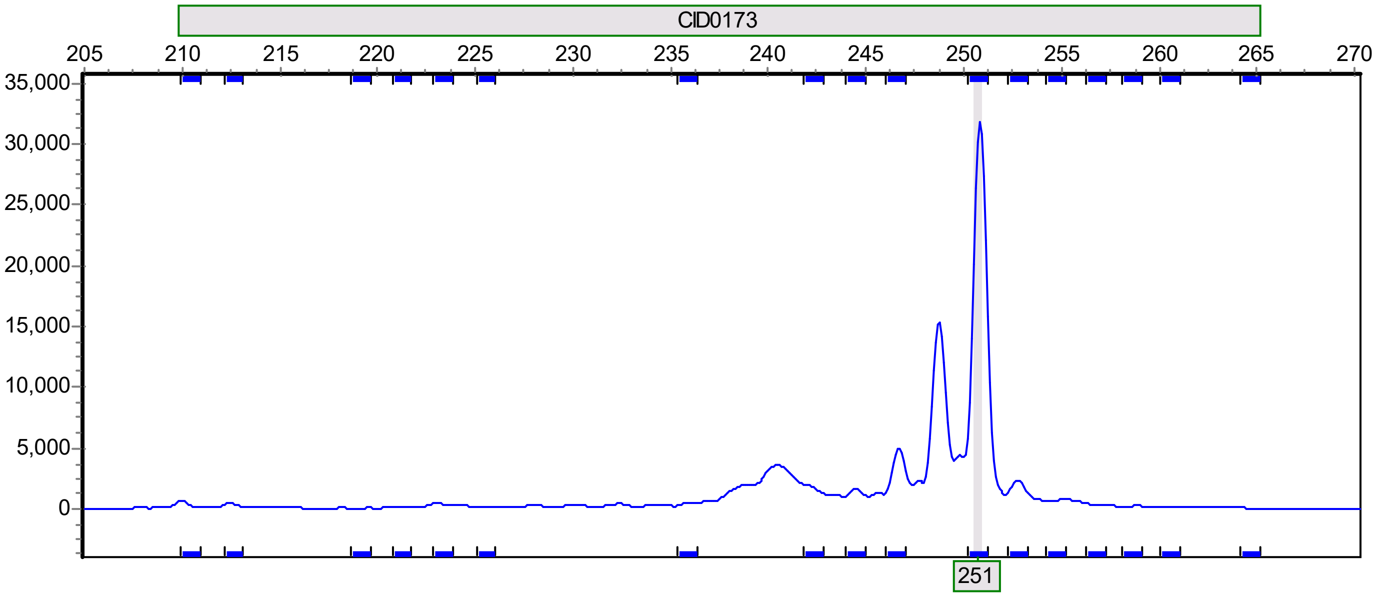

No	Size	Height	Area	Marker	Allele	Difference	Quality	Score	Allele Comments	Sample Comments
1	162.3	17576	126632	EST1573	163	0.60	Pass	500.0	[<Deleted>]	
2	170.7	42952	341954	EST1573	171	0.10	Pass	500.0	[<SAT (Repaired)><Confirmed>]	
3	250.8	31795	242075	CID0173	251	0.00	Pass	500.0	[<Confirmed>]	

Allele Report

Sample 11: CID0173_EST1573_CC1_A01.fsa Run date and time: 09/21/2024 - 03:00:19 -> 09/21/2024 - 03:27:31

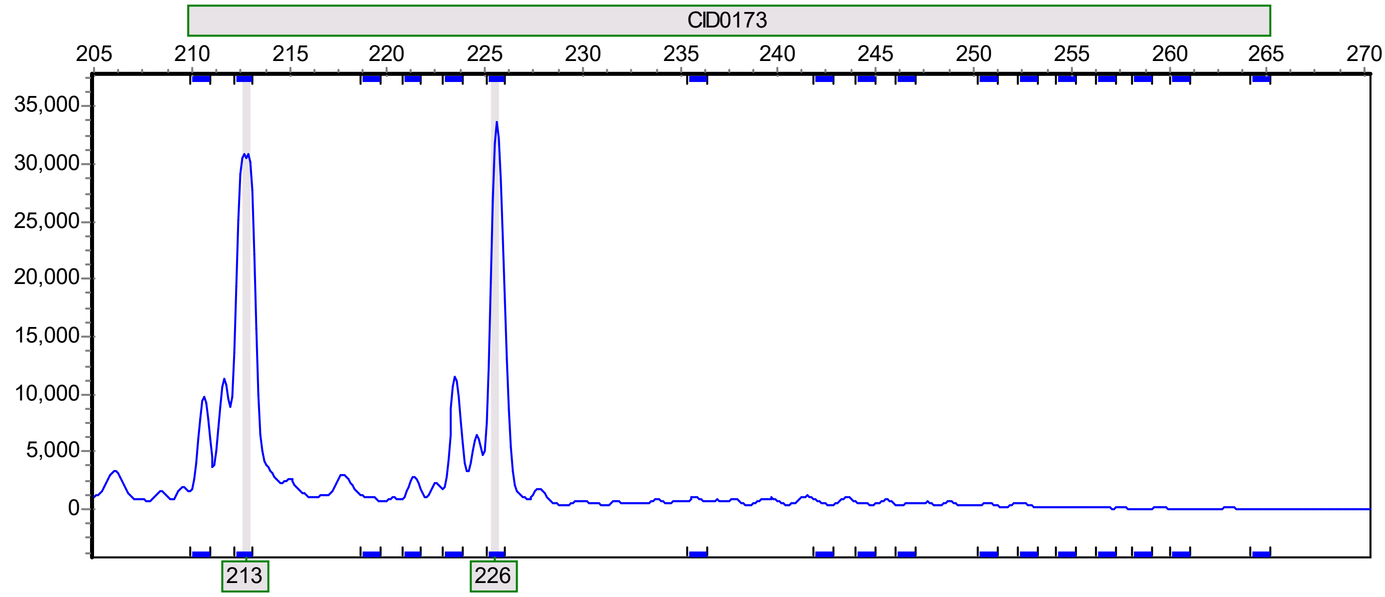

No	Size	Height	Area	Marker	Allele	Difference	Quality	Score	Allele Comments	Sample Comments
1	174.5	40877	325200	EST1573	174	0.10	Pass	500.0	[<SAT (Repaired)><Confirmed>]	
2	211.7	11241	75885	CID0173	212	0.10	Undetermined	500.0	[<Deleted>]	
3	212.8	30583	330126	CID0173	213	0.10	Pass	500.0	[<SAT (Repaired)><Confirmed>]	
4	225.6	33648	255044	CID0173	226	0.00	Pass	500.0	[<SAT (Repaired)><Confirmed>]	

Sample 12: CID0173_EST1573_CC20_G05.fsa Run date and time: 09/21/2024 - 03:00:19 -> 09/21/2024 - 03:27:31

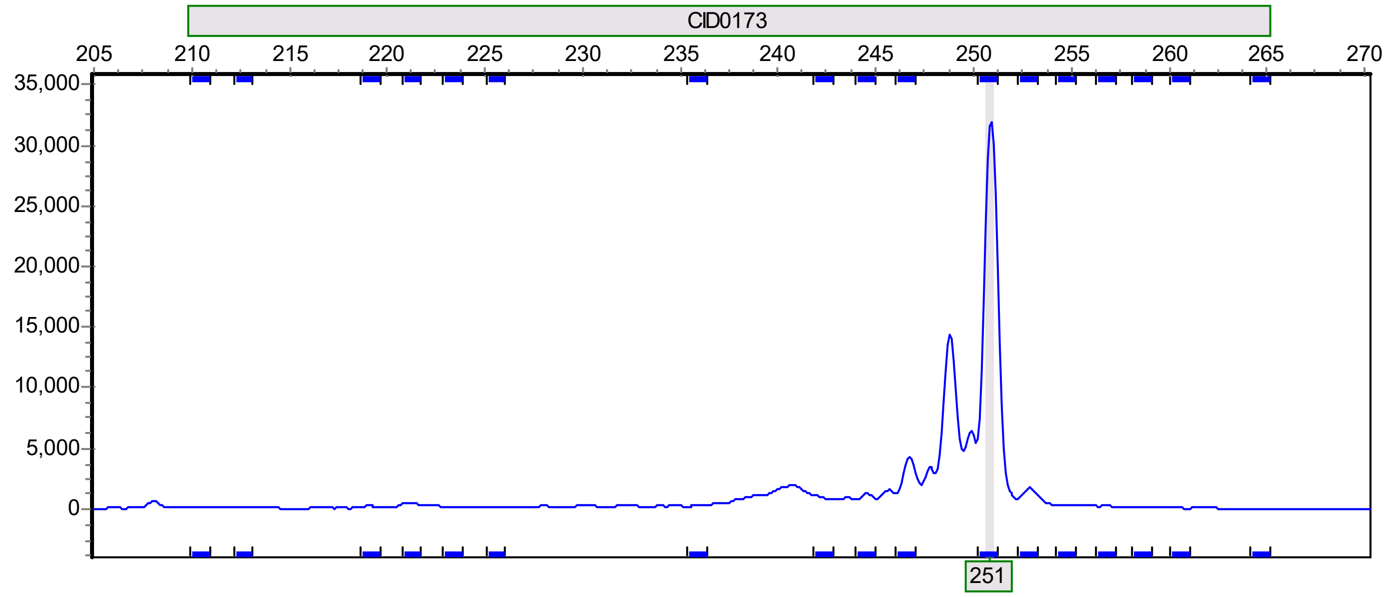

No	Size	Height	Area	Marker	Allele	Difference	Quality	Score	Allele Comments	Sample Comments
1	170.6	45777	367364	EST1573	171	0.00	Pass	500.0	[<SAT (Repaired)><Confirmed>]	
2	250.9	31927	250313	CID0173	251	0.10	Pass	500.0	[<Confirmed>]	

Sample 13: CID0173_EST1573_CC21_I05.fsa Run date and time: 09/21/2024 - 03:00:19 -> 09/21/2024 - 03:27:31

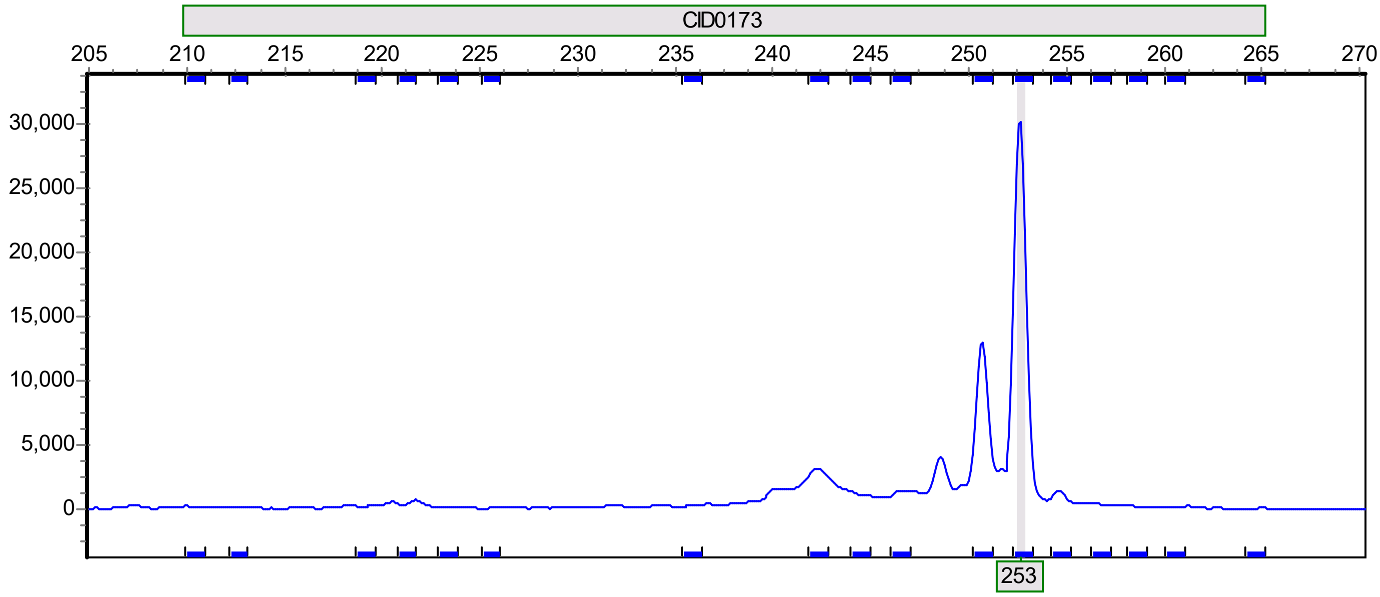

No	Size	Height	Area	Marker	Allele	Difference	Quality	Score	Allele Comments	Sample Comments
1	162.1	16085	121352	EST1573	163	0.80	Pass	500.0	[<Deleted>]	
2	170.6	30027	294234	EST1573	171	0.00	Pass	500.0	[<SAT (Repaired)><Confirmed>]	
3	252.7	30149	213229	CID0173	253	0.10	Pass	500.0	[<Confirmed>]	

Sample 14: CID0173_EST1573_CC22_K05.fsa Run date and time: 09/21/2024 - 03:00:19 -> 09/21/2024 - 03:27:31

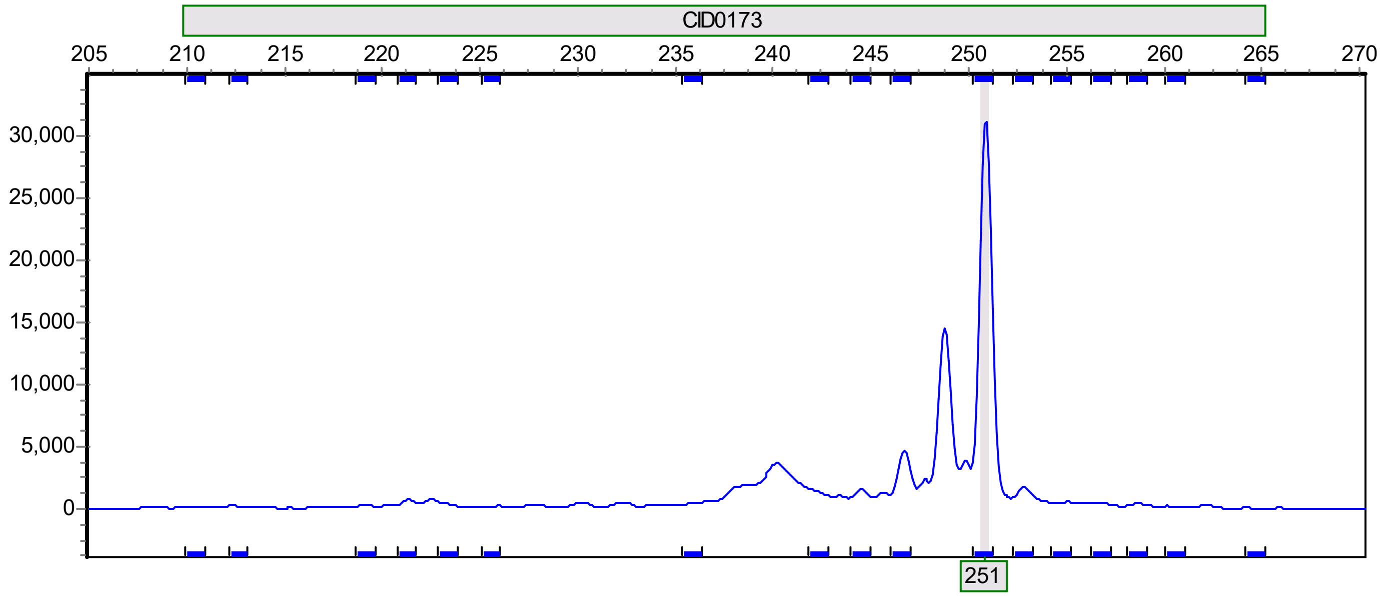

No	Size	Height	Area	Marker	Allele	Difference	Quality	Score	Allele Comments	Sample Comments
1	162.1	17917	135132	EST1573	163	0.80	Pass	500.0	[<Deleted>]	
2	170.5	30275	286886	EST1573	171	0.10	Pass	500.0	[<SAT (Repaired)><Confirmed>]	
3	250.9	31113	215567	CID0173	251	0.10	Pass	500.0	[<Confirmed>]	

Sample 15: CID0173_EST1573_CC23_M05.fsa Run date and time: 09/21/2024 - 03:00:19 -> 09/21/2024 - 03:27:31

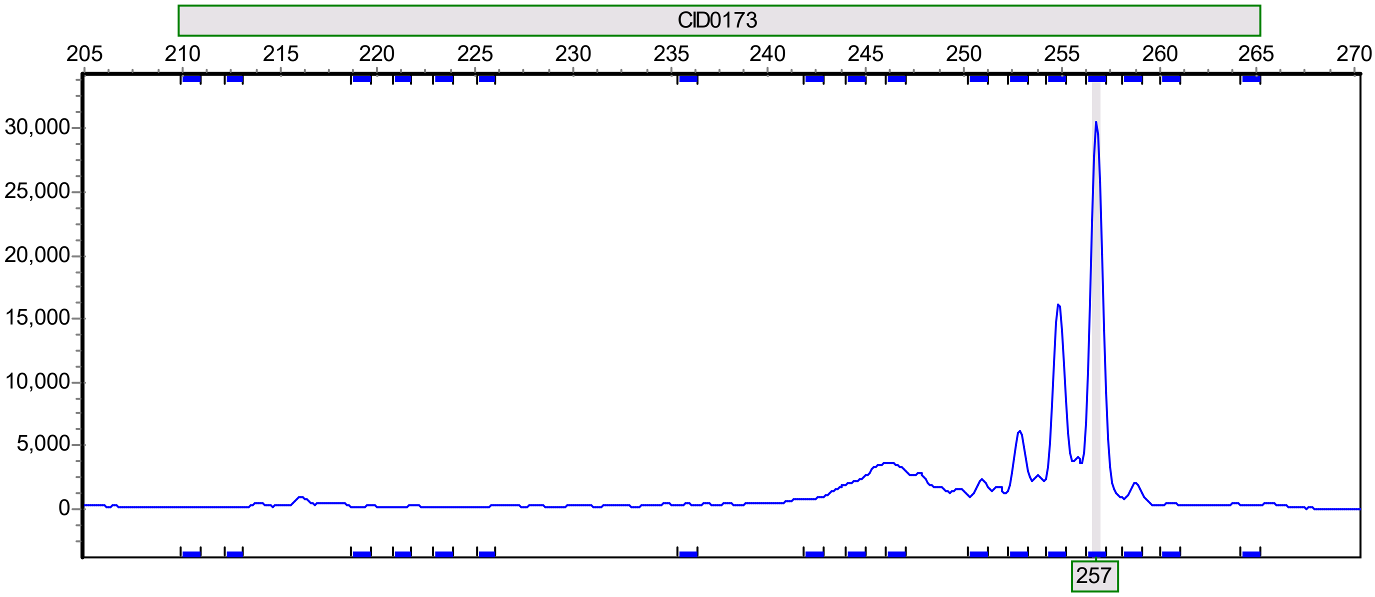

No	Size	Height	Area	Marker	Allele	Difference	Quality	Score	Allele Comments	Sample Comments
1	165.3	10434	73613	EST1573	165	0.50	Check	500.0	[<Deleted>]	
2	174.2	33524	252607	EST1573	174	0.20	Pass	500.0	[<SAT (Repaired)><Confirmed>]	
3	256.8	30519	214563	CID0173	257	0.00	Pass	500.0	[<Confirmed>]	

Sample 16: CID0173_EST1573_CC24_O05.fsa Run date and time: 09/21/2024 - 03:00:19 -> 09/21/2024 - 03:27:31

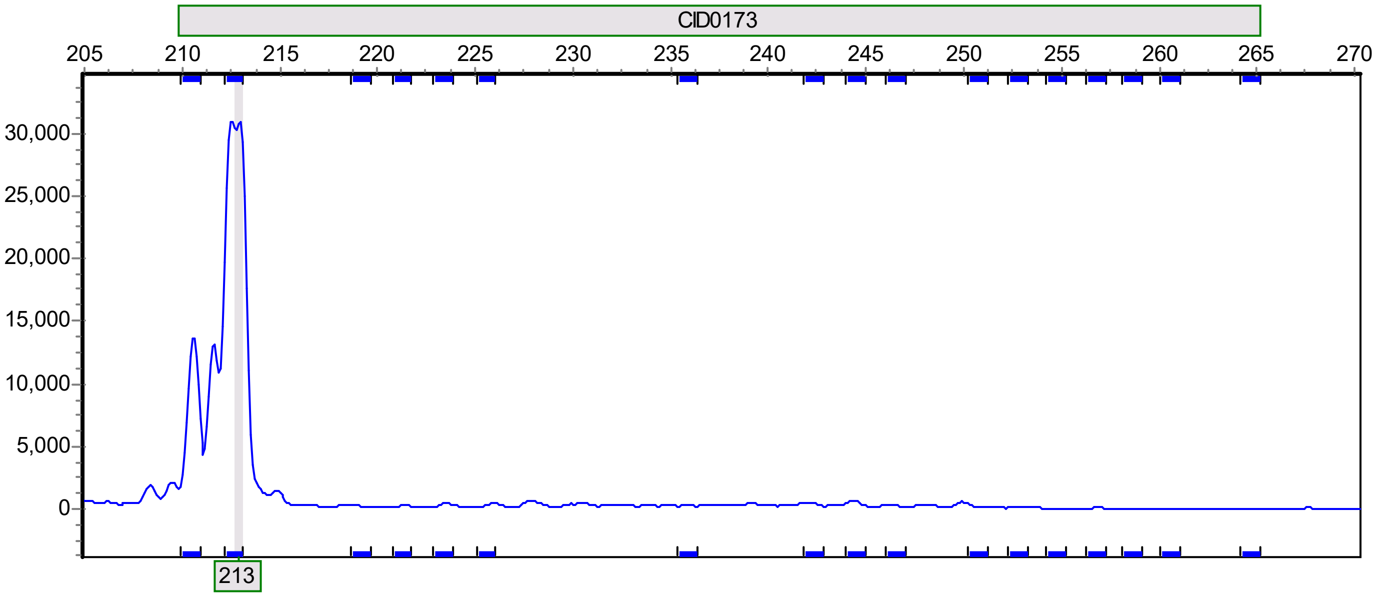

No	Size	Height	Area	Marker	Allele	Difference	Quality	Score	Allele Comments	Sample Comments
1	174.4	35426	256657	EST1573	174	0.00	Pass	500.0	[<SAT (Repaired)><Confirmed>]	
2	176.2	23473	154711	EST1573	176	0.20	Pass	500.0	[<Confirmed>]	
3	201.6	21498	183206	CID0173	202	0.00	Pass	500.0	[<Deleted>]	
4	212.9	30787	371212	CID0173	213	0.20	Pass	500.0	[<SAT (Repaired)><Confirmed>]	

Allele Report

Sample 17: CID0173_EST1573_CC25_A07.fsa Run date and time: 09/21/2024 - 03:00:19 -> 09/21/2024 - 03:27:31

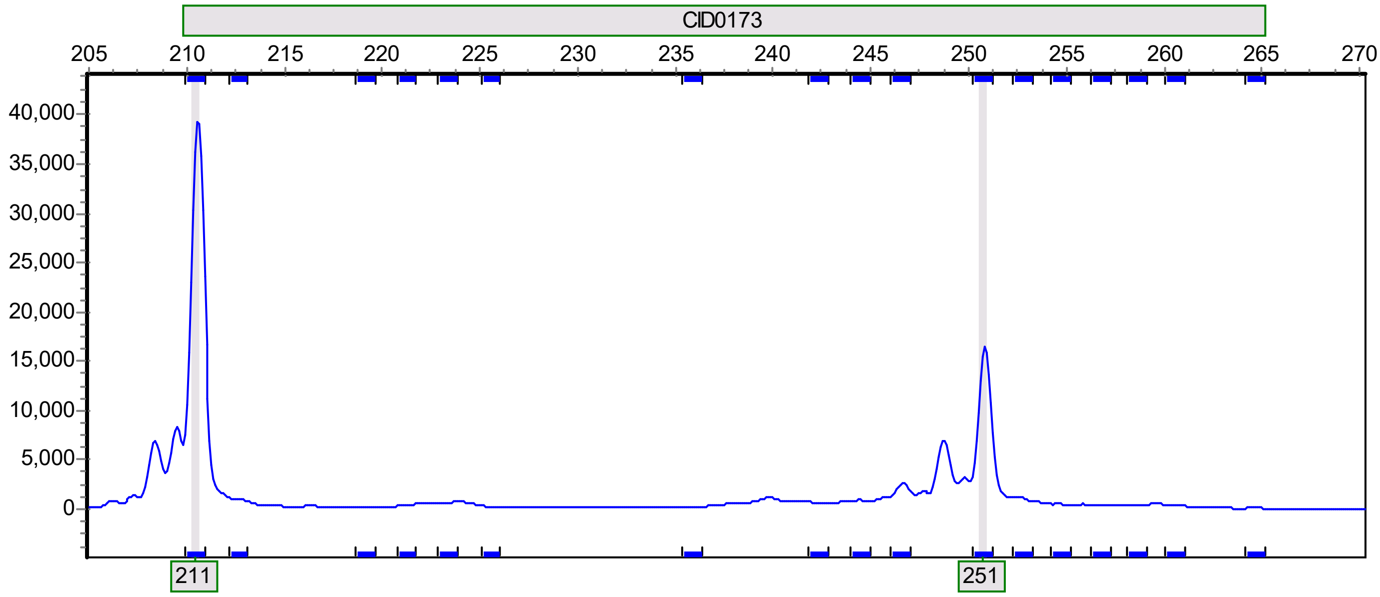

No	Size	Height	Area	Marker	Allele	Difference	Quality	Score	Allele Comments	Sample Comments
1	172.4	31885	235409	EST1573	173	0.10	Pass	500.0	[<Confirmed>]	
2	174.3	25455	182614	EST1573	174	0.10	Pass	500.0	[<Confirmed>]	
3	210.5	39283	316708	CID0173	211	0.00	Pass	500.0	[<SAT (Repaired)><Confirmed>]	
4	250.8	16494	121926	CID0173	251	0.00	Pass	500.0	[<Confirmed>]	

Sample 18: CID0173_EST1573_CC26_C07.fsa Run date and time: 09/21/2024 - 03:00:19 -> 09/21/2024 - 03:27:31

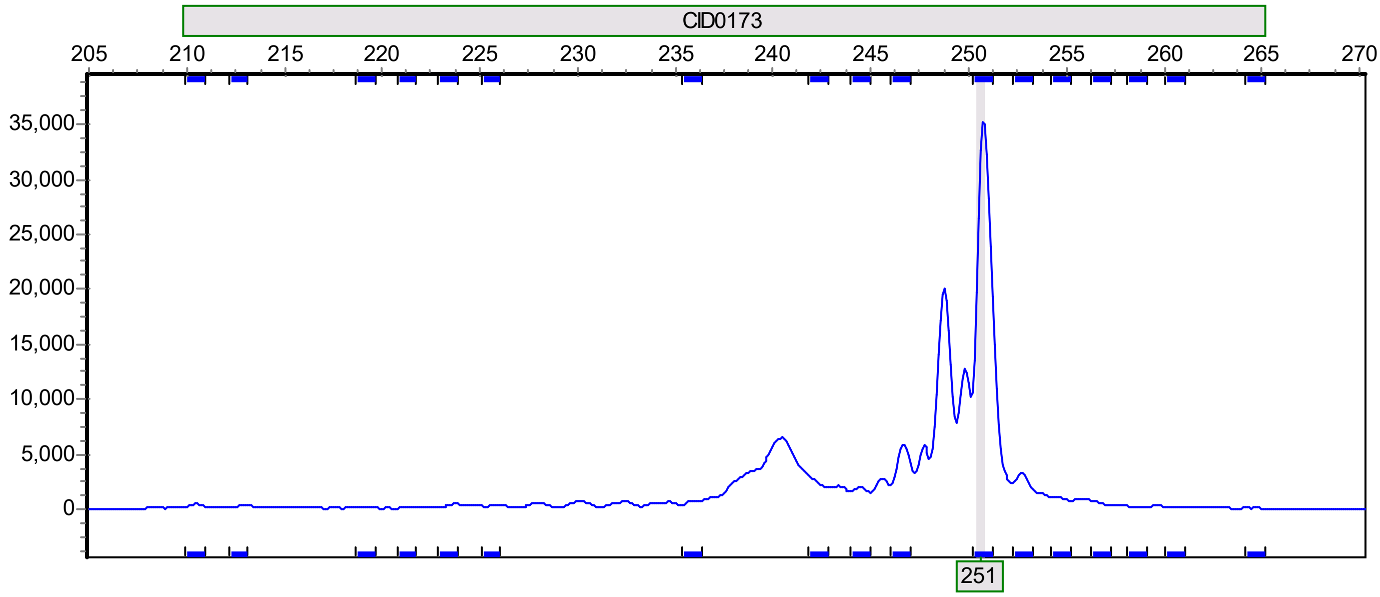

No	Size	Height	Area	Marker	Allele	Difference	Quality	Score	Allele Comments	Sample Comments
1	162.2	29842	223007	EST1573	163	0.70	Pass	500.0	[<Deleted>]	
2	170.5	36041	304051	EST1573	171	0.10	Pass	500.0	[<SAT (Repaired)><Confirmed>]	
3	249.8	12702	77529	CID0173	250	0.00	Pass	500.0	[<Deleted>]	
4	250.7	35215	297057	CID0173	251	0.10	Pass	500.0	[<SAT (Repaired)><Confirmed>]	

Sample 19: CID0173_EST1573_CC27_E07.fsa Run date and time: 09/21/2024 - 03:00:19 -> 09/21/2024 - 03:27:31

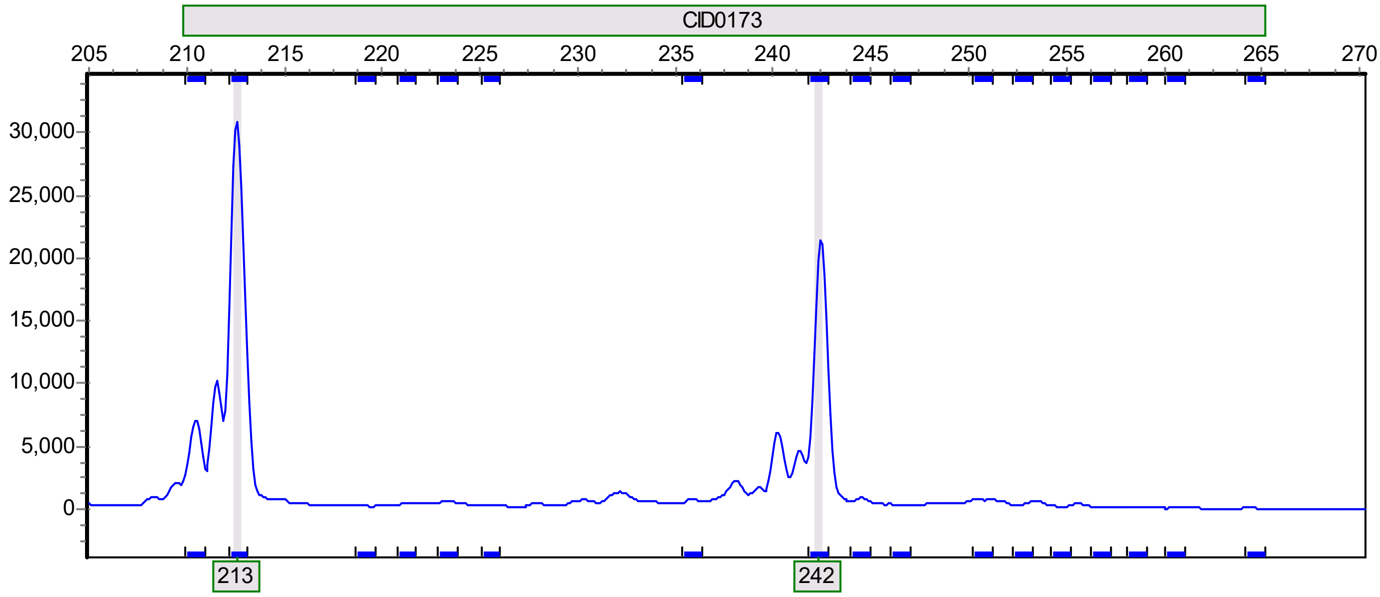

No	Size	Height	Area	Marker	Allele	Difference	Quality	Score	Allele Comments	Sample Comments
1	170.5	34389	272457	EST1573	171	0.10	Pass	500.0	[<SAT (Repaired)><Confirmed>]	
2	176.4	31715	234805	EST1573	176	0.00	Pass	500.0	[<Confirmed>]	
3	211.6	10175	67796	CID0173	212	0.00	Undetermined	500.0	[<Deleted>]	
4	212.6	30853	250050	CID0173	213	0.10	Pass	500.0	[<SAT (Repaired)><Confirmed>]	
5	242.4	21368	162653	CID0173	242	0.00	Pass	500.0	[<Confirmed>]	

Sample 20: CID0173_EST1573_CC28_G07.fsa Run date and time: 09/21/2024 - 03:00:19 -> 09/21/2024 - 03:27:31

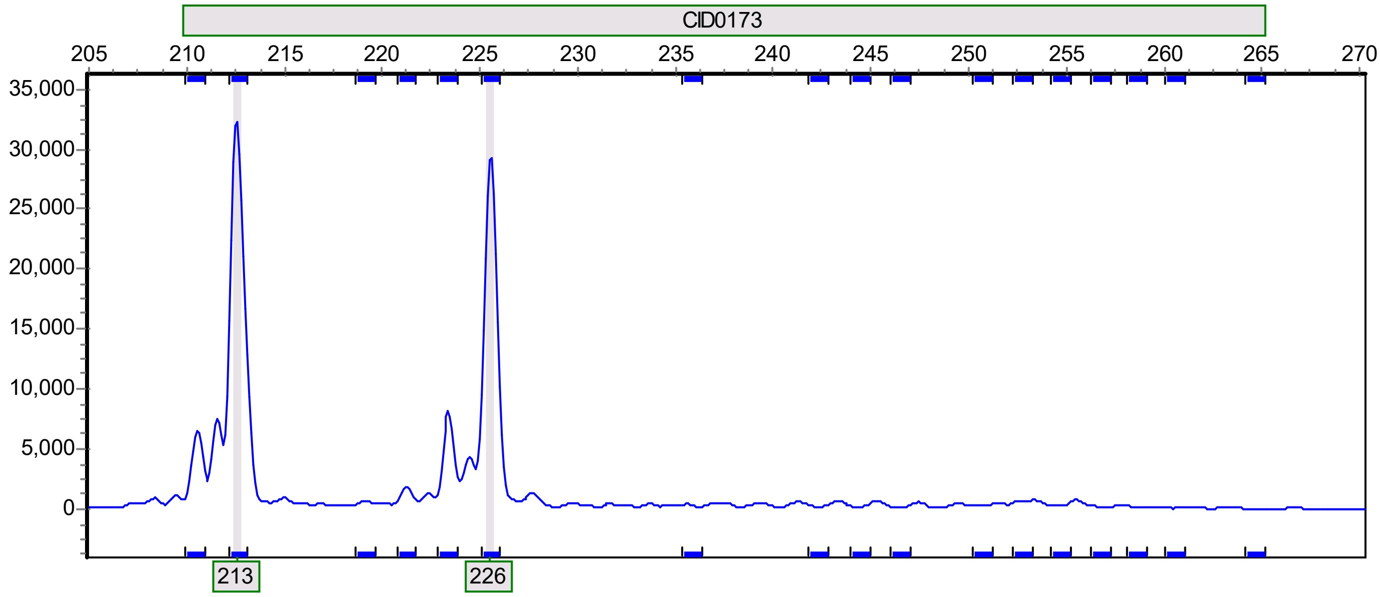

No	Size	Height	Area	Marker	Allele	Difference	Quality	Score	Allele Comments	Sample Comments
1	178.2	32099	230979	EST1573	178	0.00	Pass	500.0	[<Confirmed>]	
2	180.1	25045	162165	EST1573	180	0.10	Pass	500.0	[<Confirmed>]	
3	212.6	32266	261218	CID0173	213	0.10	Pass	500.0	[<SAT (Repaired)><Confirmed>]	
4	225.6	29191	209543	CID0173	226	0.00	Pass	500.0	[<Confirmed>]	

Sample 21: CID0173_EST1573_CC29_I07.fsa Run date and time: 09/21/2024 - 03:00:19 -> 09/21/2024 - 03:27:31

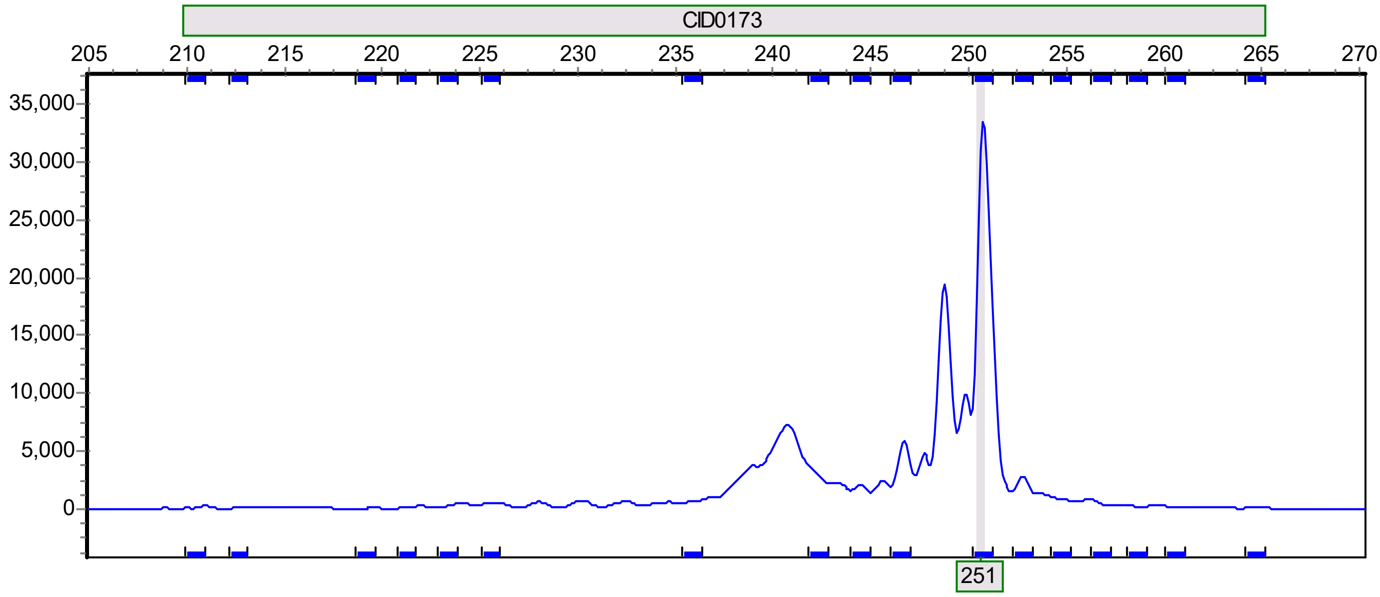

No	Size	Height	Area	Marker	Allele	Difference	Quality	Score	Allele Comments	Sample Comments
1	162.2	30873	227607	EST1573	163	0.70	Check	500.0	[<Deleted>]	
2	170.7	48761	404279	EST1573	171	0.10	Pass	500.0	[<SAT (Repaired)><Confirmed>]	
3	250.7	33471	270352	CID0173	251	0.10	Pass	500.0	[<SAT (Repaired)><Confirmed>]	

Sample 22: CID0173_EST1573_CC2_C01.fsa Run date and time: 09/21/2024 - 03:00:19 -> 09/21/2024 - 03:27:31

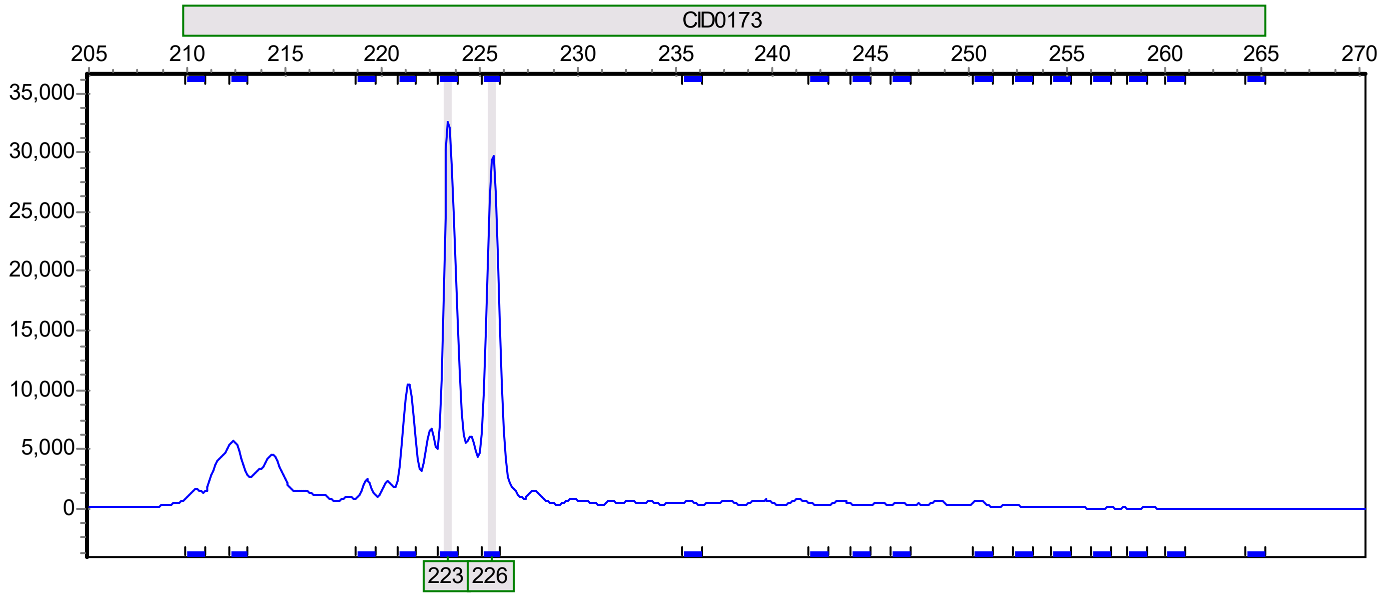

No	Size	Height	Area	Marker	Allele	Difference	Quality	Score	Allele Comments	Sample Comments
1	170.5	28362	226038	EST1573	171	0.10	Pass	500.0	[<SAT (Repaired)><Confirmed>]	
2	174.4	26038	166996	EST1573	174	0.00	Pass	500.0	[<Confirmed>]	
3	223.4	32569	258924	CID0173	223	0.00	Pass	500.0	[<SAT (Repaired)><Confirmed>]	
4	225.7	29649	213750	CID0173	226	0.10	Pass	500.0	[<Confirmed>]	

Allele Report

Sample 23: CID0173_EST1573_CC30_K07.fsa Run date and time: 09/21/2024 - 03:00:19 -> 09/21/2024 - 03:27:31

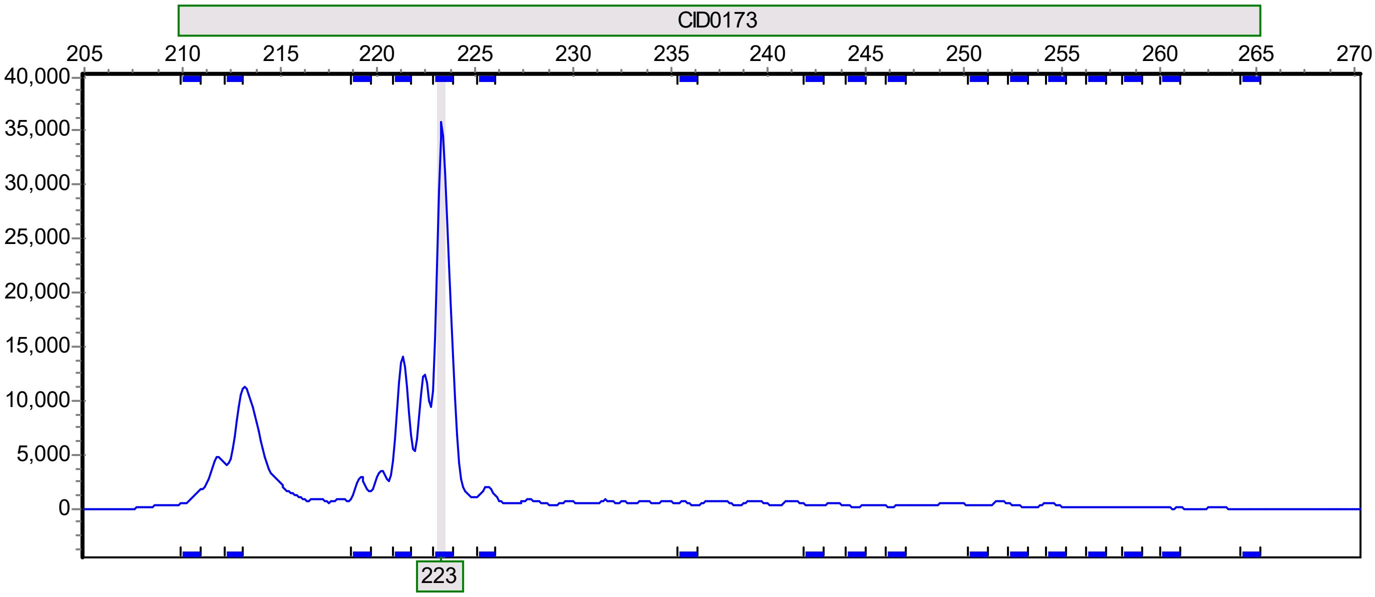

No	Size	Height	Area	Marker	Allele	Difference	Quality	Score	Allele Comments	Sample Comments
1	163.9	23520	165224	EST1573	164	0.10	Pass	500.0	[<Deleted>]	
2	172.3	30714	306855	EST1573	173	0.20	Pass	500.0	[<SAT (Repaired)><Confirmed>]	
3	213.2	11265	148968	CID0173	213	0.50	Undetermined	411.0	[<Deleted>]	
4	222.4	12522	87665	CID0173	222	0.00	Pass	500.0	[<Deleted>]	
5	223.3	35827	308657	CID0173	223	0.10	Pass	500.0	[<SAT (Repaired)><Confirmed>]	

Sample 24: CID0173_EST1573_CC31_M07.fsa Run date and time: 09/21/2024 - 03:00:19 -> 09/21/2024 - 03:27:31

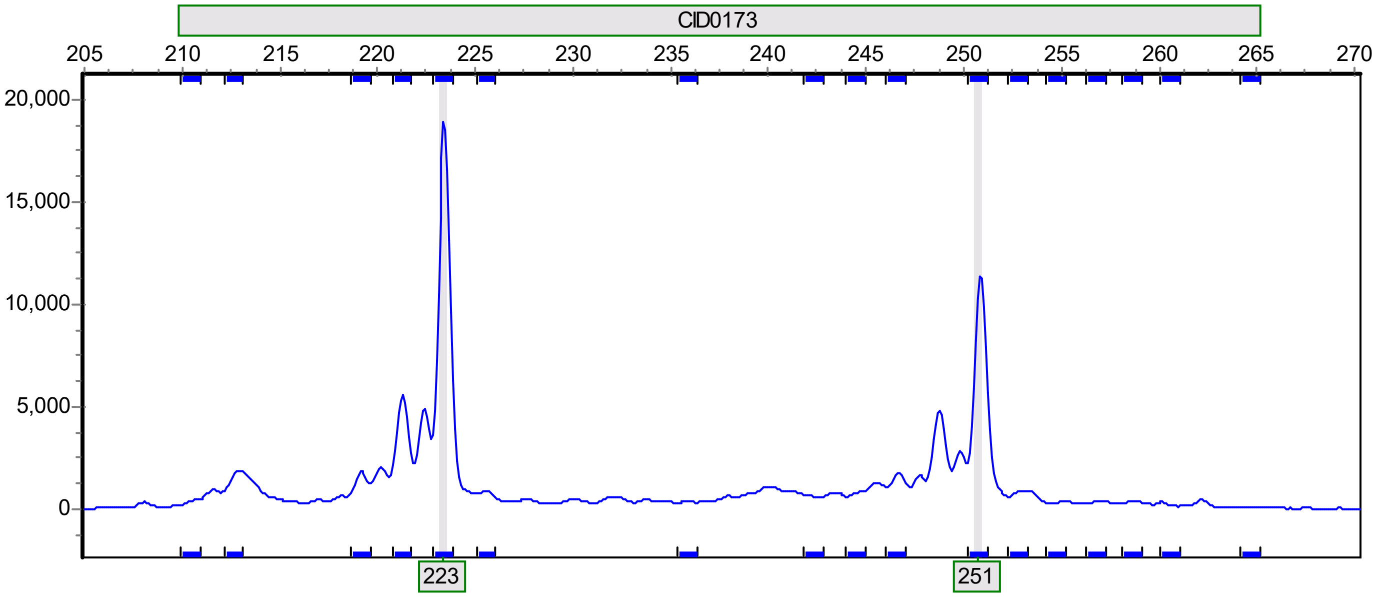

No	Size	Height	Area	Marker	Allele	Difference	Quality	Score	Allele Comments	Sample Comments
1	172.5	28912	192548	EST1573	173	0.00	Pass	500.0	[<Confirmed>]	
2	176.2	15469	101930	EST1573	176	0.20	Pass	500.0	[<Confirmed>]	
3	223.4	18925	140874	CID0173	223	0.00	Pass	500.0	[<Confirmed>]	
4	250.8	11405	80383	CID0173	251	0.00	Pass	500.0	[<Confirmed>]	

Sample 25: CID0173_EST1573_CC32_O07.fsa Run date and time: 09/21/2024 - 03:00:19 -> 09/21/2024 - 03:27:31

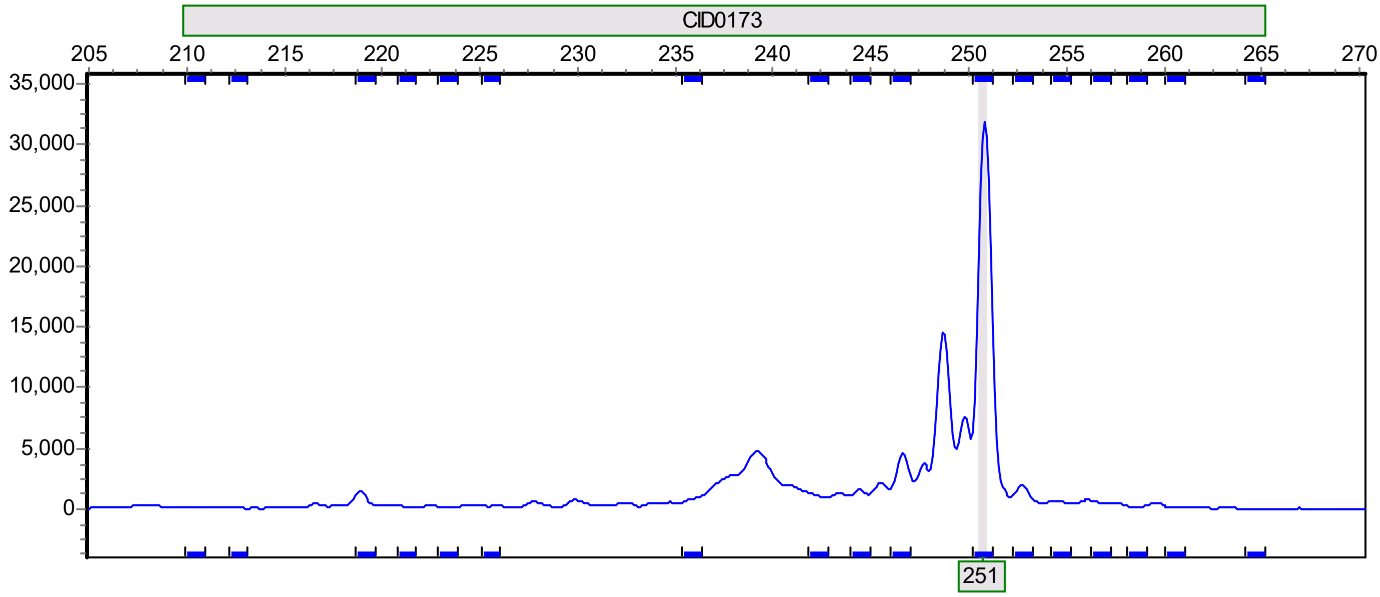

No	Size	Height	Area	Marker	Allele	Difference	Quality	Score	Allele Comments	Sample Comments
1	161.6	22071	154451	EST1573	161	0.30	Pass	500.0	[<Deleted>]	
2	170.6	42319	327762	EST1573	171	0.00	Pass	500.0	[<SAT (Repaired)><Confirmed>]	
3	250.8	31857	241679	CID0173	251	0.00	Pass	500.0	[<Confirmed>]	

Sample 26: CID0173_EST1573_CC33_A09.fsa Run date and time: 09/21/2024 - 03:00:19 -> 09/21/2024 - 03:27:31

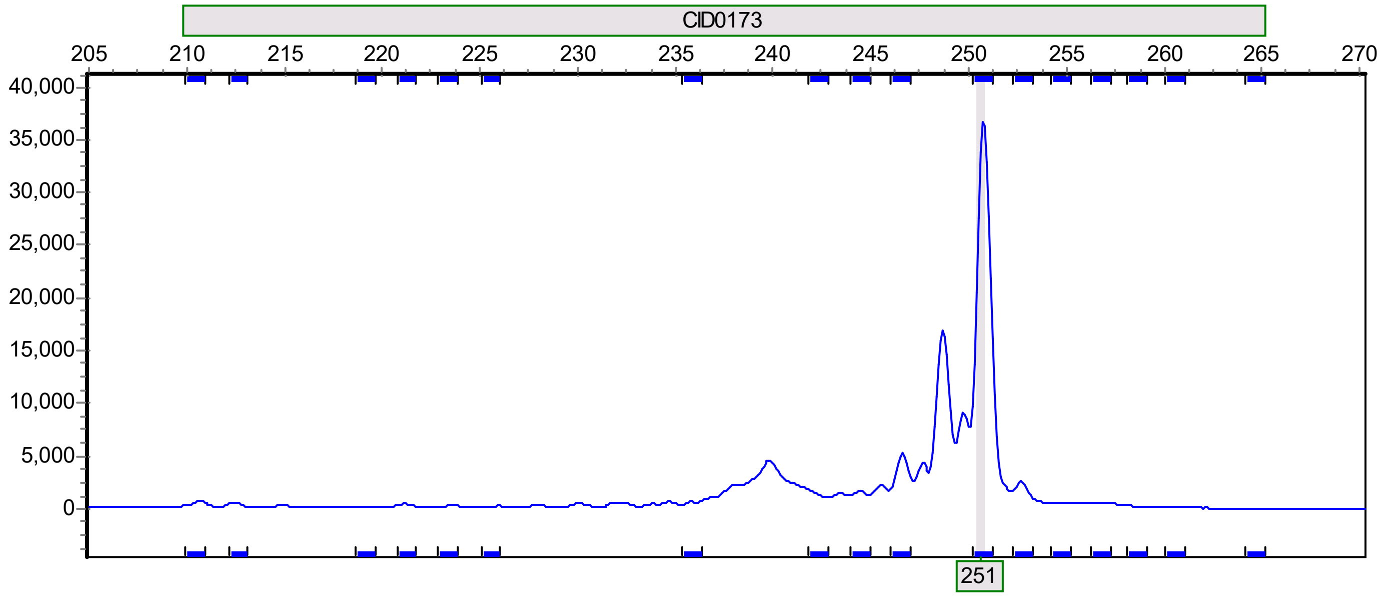

No	Size	Height	Area	Marker	Allele	Difference	Quality	Score	Allele Comments	Sample Comments
1	163.7	17449	135852	EST1573	164	0.30	Pass	500.0	[<Deleted>]	
2	172.5	40769	350115	EST1573	173	0.00	Pass	500.0	[<SAT (Repaired)><Confirmed>]	
3	250.7	36676	279625	CID0173	251	0.10	Pass	500.0	[<SAT (Repaired)><Confirmed>]	

Allele Report

Sample 27: CID0173_EST1573_CC34_C09.fsa Run date and time: 09/21/2024 - 03:00:19 -> 09/21/2024 - 03:27:31

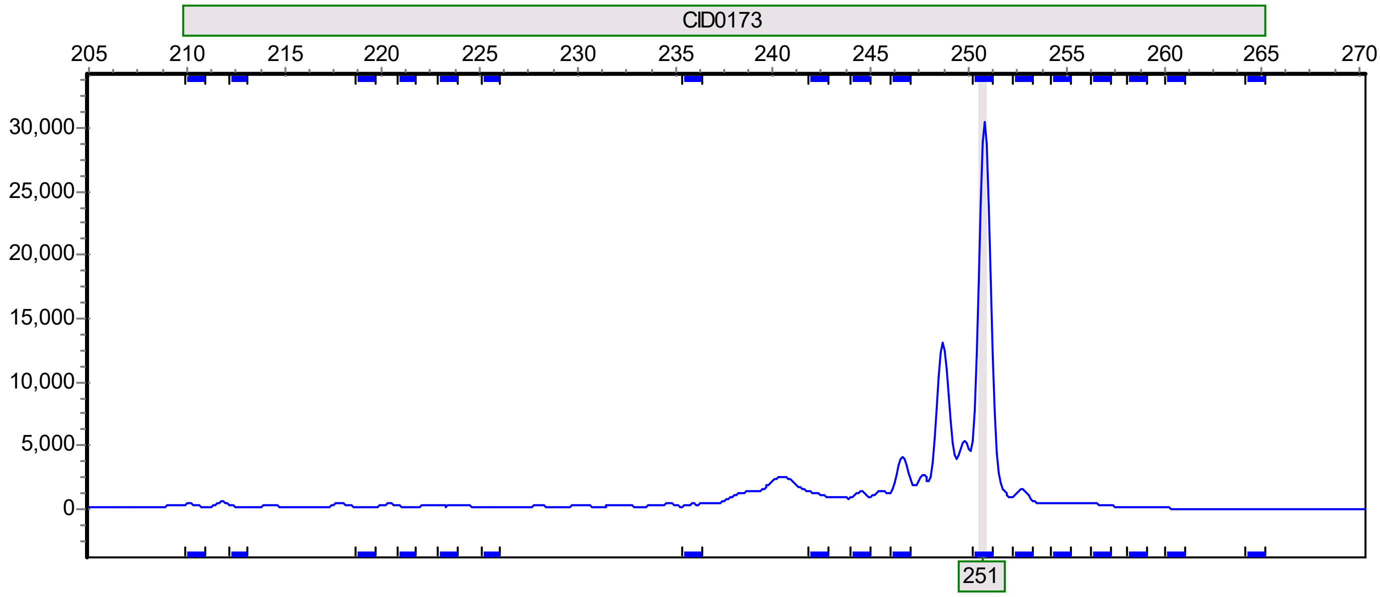

No	Size	Height	Area	Marker	Allele	Difference	Quality	Score	Allele Comments	Sample Comments
1	164.0	10932	78162	EST1573	164	0.00	Pass	500.0	[<Deleted>]	
2	172.5	31713	288691	EST1573	173	0.00	Pass	500.0	[<Confirmed>]	
3	250.8	30500	211671	CID0173	251	0.00	Pass	500.0	[<Confirmed>]	

Sample 28: CID0173_EST1573_CC35_E09.fsa Run date and time: 09/21/2024 - 03:00:19 -> 09/21/2024 - 03:27:31

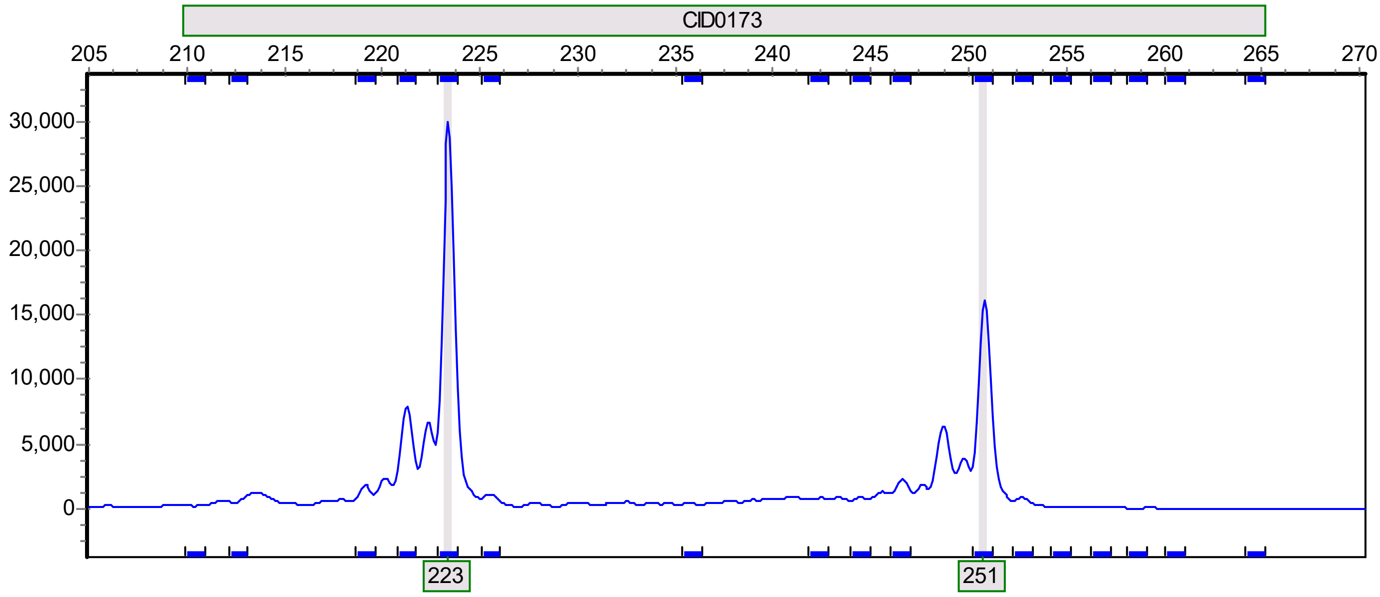

No	Size	Height	Area	Marker	Allele	Difference	Quality	Score	Allele Comments	Sample Comments
1	170.6	41220	345779	EST1573	171	0.00	Pass	500.0	[<SAT (Repaired)><Confirmed>]	
2	223.4	29936	223893	CID0173	223	0.00	Pass	500.0	[<Confirmed>]	
3	250.8	16116	116219	CID0173	251	0.00	Pass	500.0	[<Confirmed>]	

Sample 29: CID0173_EST1573_CC36_G09.fsa Run date and time: 09/21/2024 - 03:00:19 -> 09/21/2024 - 03:27:31

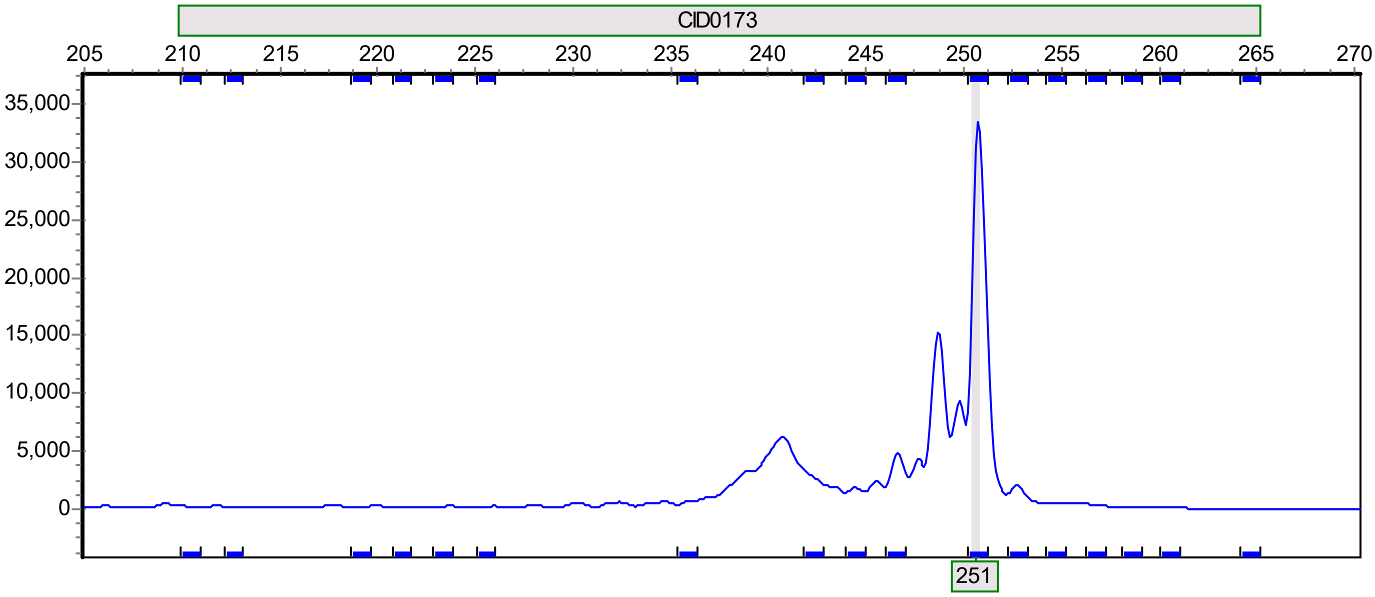

No	Size	Height	Area	Marker	Allele	Difference	Quality	Score	Allele Comments	Sample Comments
1	164.0	24139	173772	EST1573	164	0.00	Pass	500.0	[<Deleted>]	
2	172.5	40422	339522	EST1573	173	0.00	Pass	500.0	[<SAT (Repaired)><Confirmed>]	
3	250.7	33485	258302	CID0173	251	0.10	Pass	500.0	[<SAT (Repaired)><Confirmed>]	

Sample 30: CID0173_EST1573_CC37_I09.fsa Run date and time: 09/21/2024 - 03:00:19 -> 09/21/2024 - 03:27:31

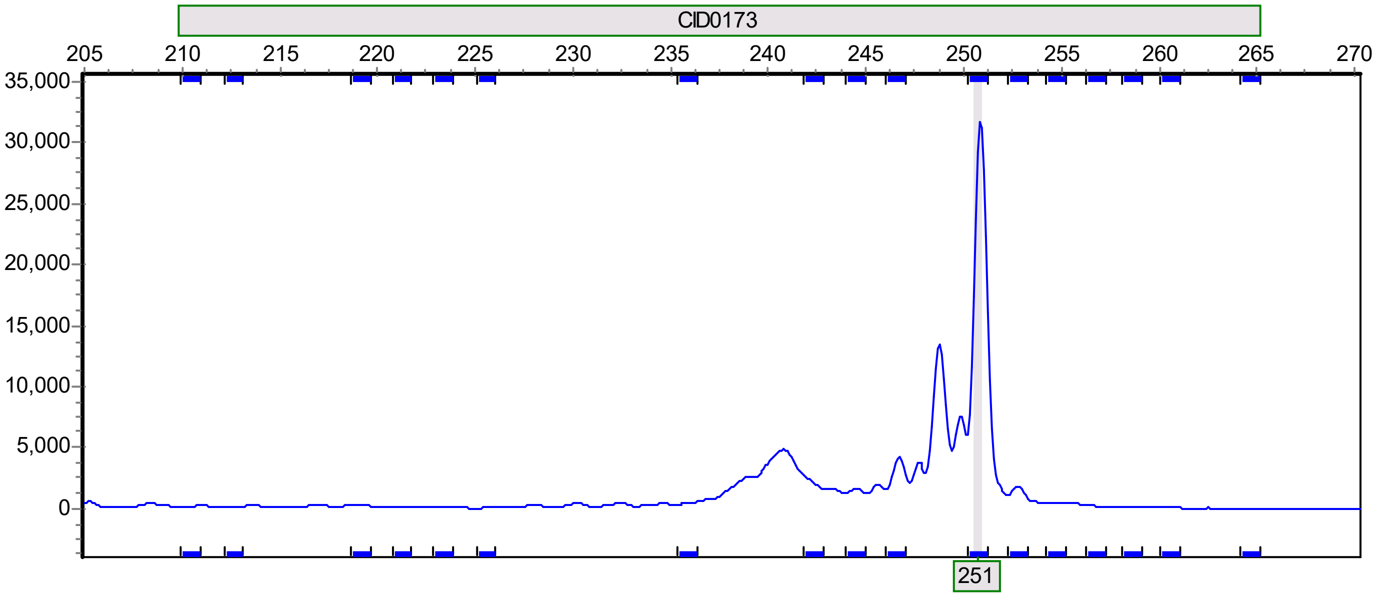

No	Size	Height	Area	Marker	Allele	Difference	Quality	Score	Allele Comments	Sample Comments
1	162.3	22426	158754	EST1573	163	0.60	Pass	500.0	[<Deleted>]	
2	170.6	36974	289537	EST1573	171	0.00	Pass	500.0	[<SAT (Repaired)><Confirmed>]	
3	250.8	31676	234874	CID0173	251	0.00	Pass	500.0	[<Confirmed>]	

Sample 31: CID0173_EST1573_CC38_K09.fsa Run date and time: 09/21/2024 - 03:00:19 -> 09/21/2024 - 03:27:31

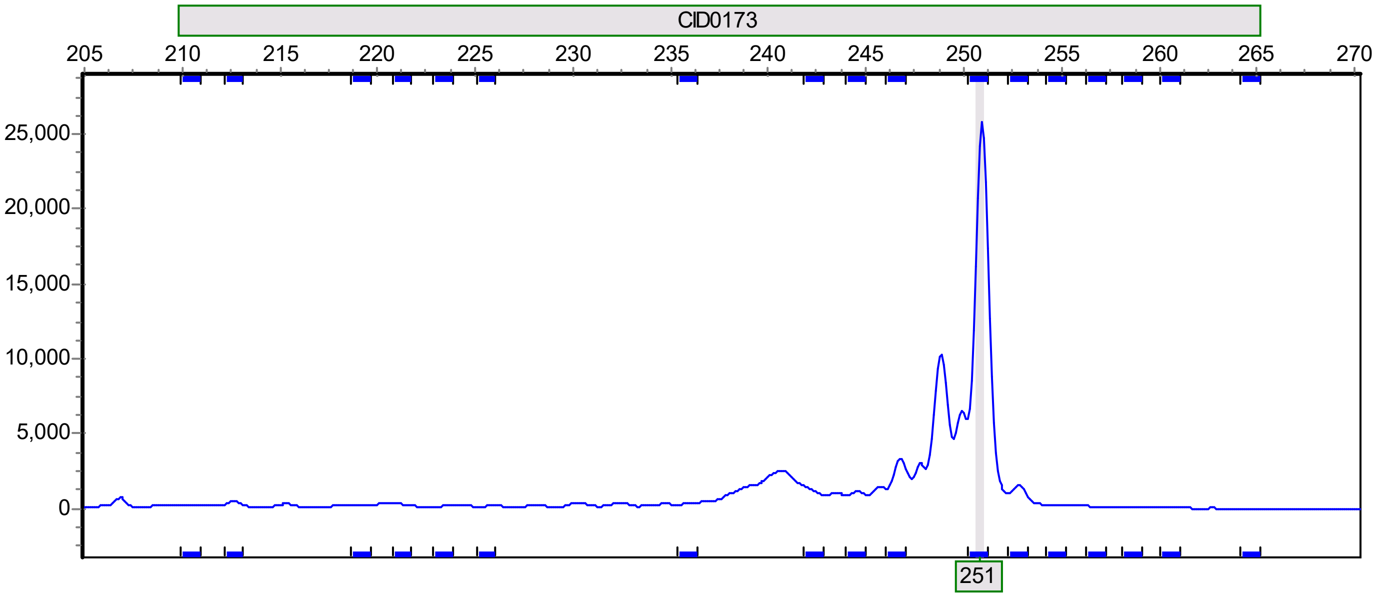

No	Size	Height	Area	Marker	Allele	Difference	Quality	Score	Allele Comments	Sample Comments
1	162.2	12080	91374	EST1573	163	0.70	Pass	500.0	[<Deleted>]	
2	170.6	37495	297516	EST1573	171	0.00	Pass	500.0	[<SAT (Repaired)><Confirmed>]	
3	250.9	25743	196247	CID0173	251	0.10	Pass	500.0	[<Confirmed>]	

Sample 32: CID0173_EST1573_CC3_E01.fsa Run date and time: 09/21/2024 - 03:00:19 -> 09/21/2024 - 03:27:31

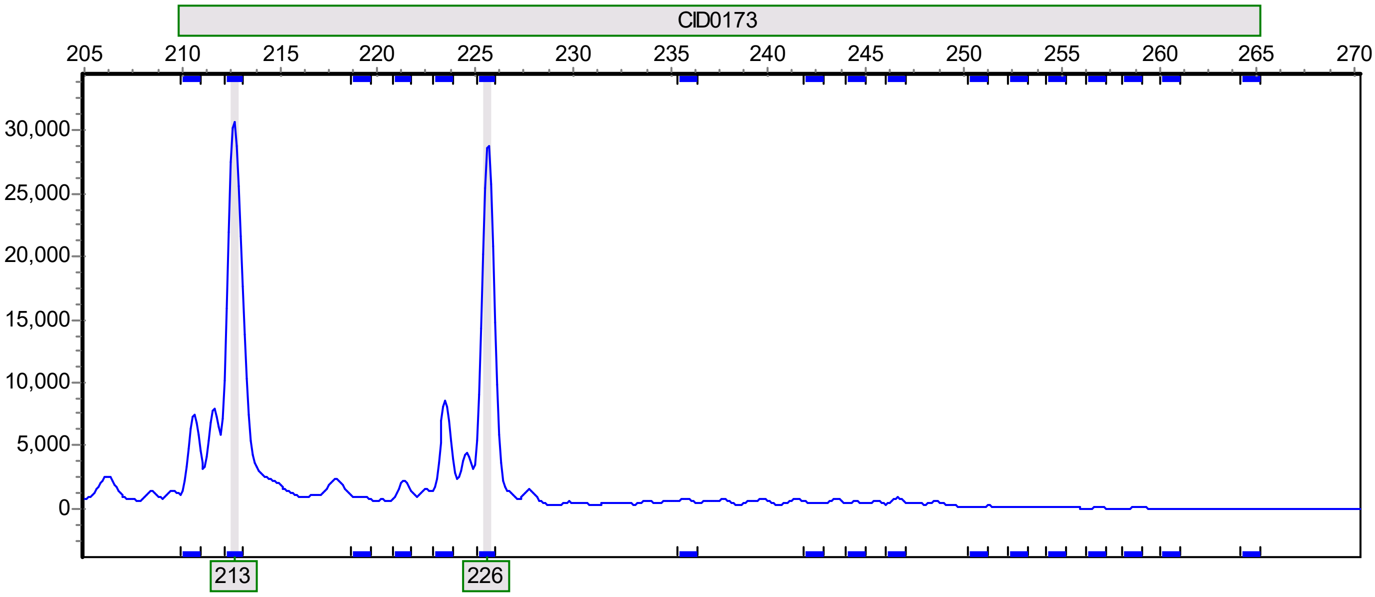

No	Size	Height	Area	Marker	Allele	Difference	Quality	Score	Allele Comments	Sample Comments
1	164.2	9154	69050	EST1573	164	0.20	Pass	500.0	[<Deleted>]	
2	174.1	29270	232450	EST1573	174	0.30	Pass	500.0	[<SAT (Repaired)><Confirmed>]	
3	212.7	30660	261912	CID0173	213	0.00	Pass	500.0	[<SAT (Repaired)><Confirmed>]	
4	225.7	28793	202823	CID0173	226	0.10	Pass	500.0	[<Confirmed>]	

Allele Report

Sample 33: CID0173_EST1573_CC4_G01.fsa Run date and time: 09/21/2024 - 03:00:19 -> 09/21/2024 - 03:27:31

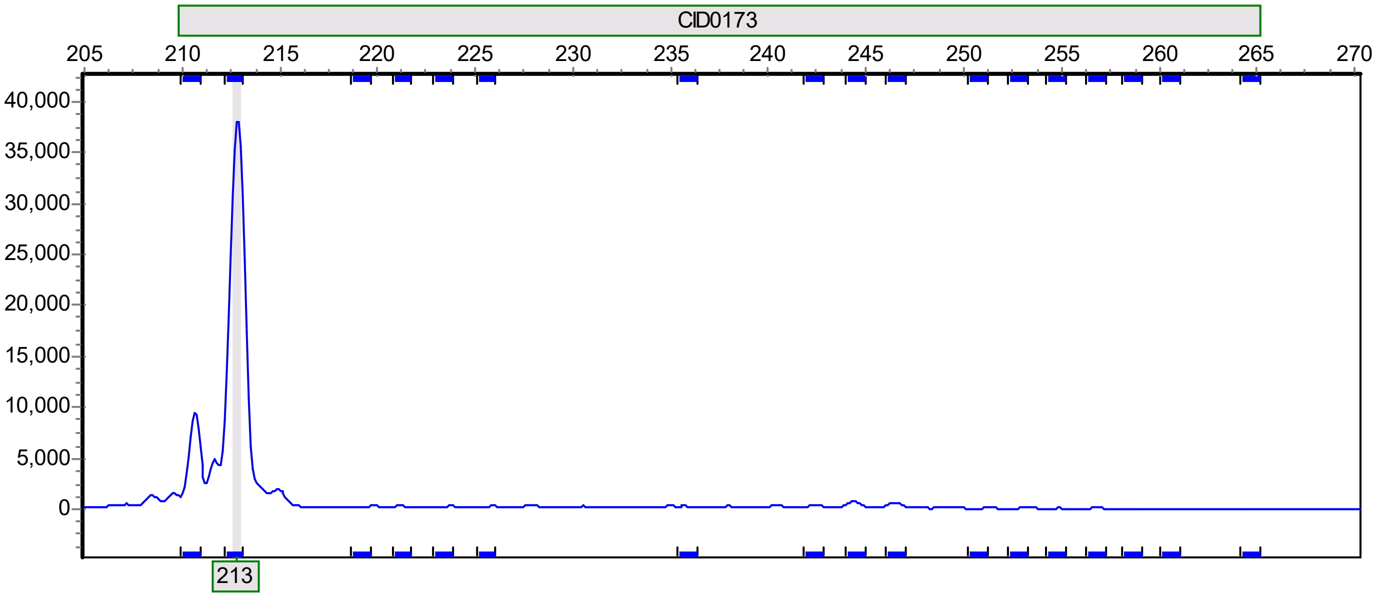

No	Size	Height	Area	Marker	Allele	Difference	Quality	Score	Allele Comments	Sample Comments
1	174.5	31198	215666	EST1573	174	0.10	Pass	500.0	[<Confirmed>]	
2	212.8	38011	326419	CID0173	213	0.10	Pass	500.0	[<SAT (Repaired)><Confirmed>]	

Sample 34: CID0173_EST1573_CC5_I01.fsa Run date and time: 09/21/2024 - 03:00:19 -> 09/21/2024 - 03:27:31

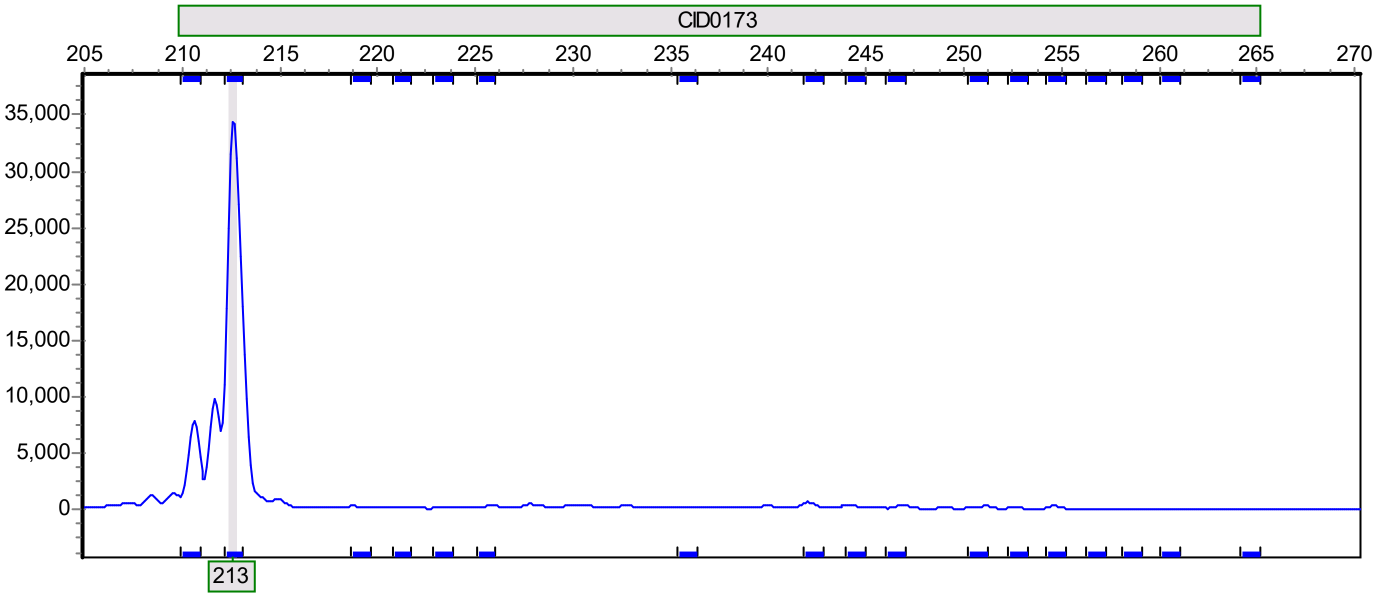

No	Size	Height	Area	Marker	Allele	Difference	Quality	Score	Allele Comments	Sample Comments
1	176.6	33087	240464	EST1573	176	0.20	Pass	500.0	[<SAT (Repaired)><Confirmed>]	
2	212.6	34373	277259	CID0173	213	0.10	Pass	500.0	[<SAT (Repaired)><Confirmed>]	

Allele Report

Sample 35: CID0173_EST1573_CC6_K01.fsa Run date and time: 09/21/2024 - 03:00:19 -> 09/21/2024 - 03:27:31

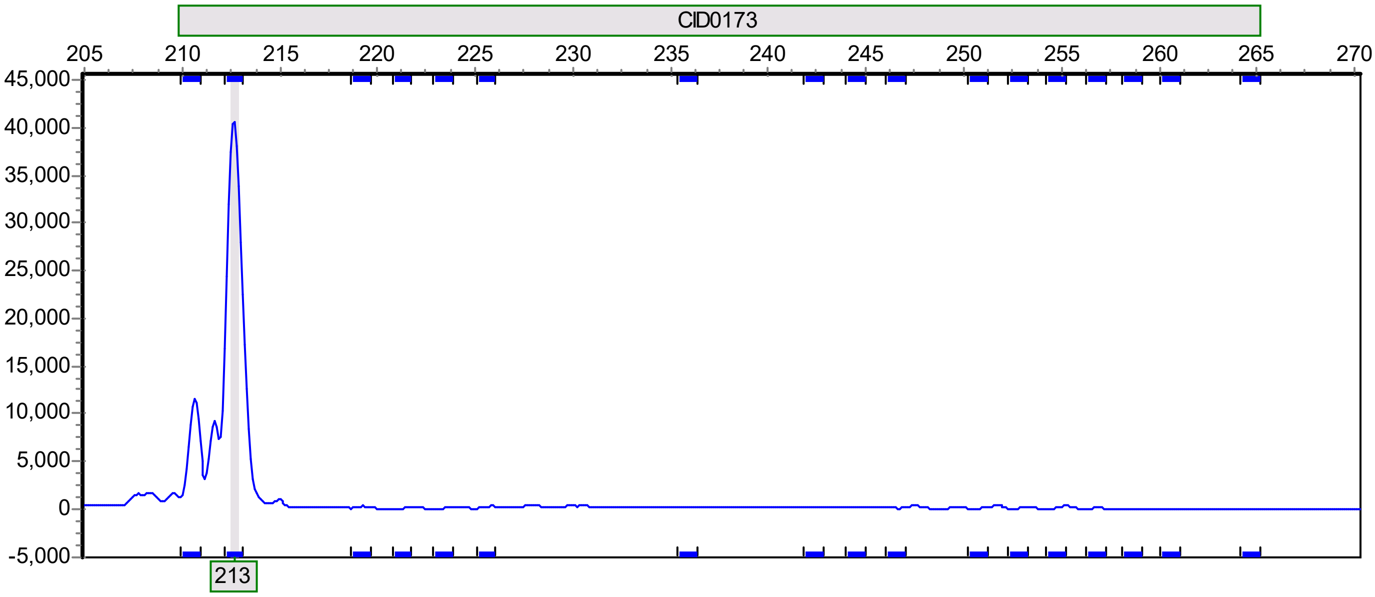

No	Size	Height	Area	Marker	Allele	Difference	Quality	Score	Allele Comments	Sample Comments
1	160.4	13789	95397	EST1573	160	0.10	Pass	500.0	[<Deleted>]	
2	170.7	33855	275274	EST1573	171	0.10	Pass	500.0	[<SAT (Repaired)><Confirmed>]	
3	212.7	40635	361489	CID0173	213	0.00	Pass	500.0	[<SAT (Repaired)><Confirmed>]	

Sample 36: CID0173_EST1573_CC7_M01.fsa Run date and time: 09/21/2024 - 03:00:19 -> 09/21/2024 - 03:27:31

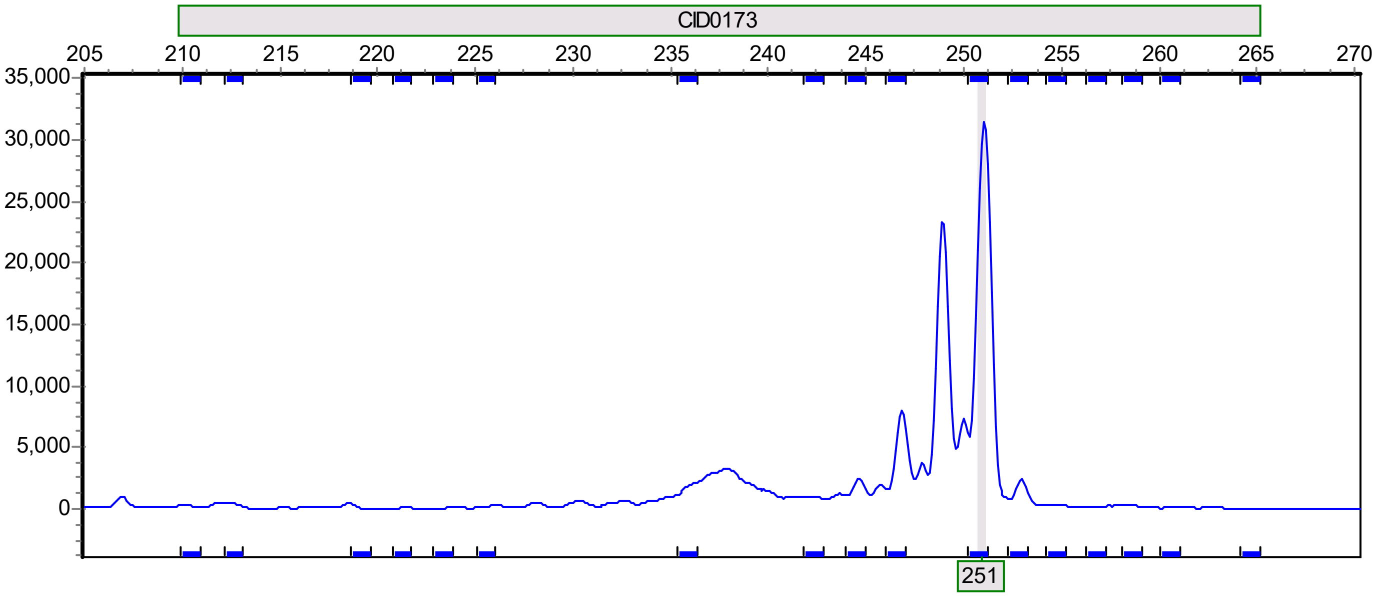

No	Size	Height	Area	Marker	Allele	Difference	Quality	Score	Allele Comments	Sample Comments
1	160.4	19743	143138	EST1573	160	0.10	Pass	500.0	[<Deleted>]	
2	170.8	47079	381093	EST1573	171	0.20	Pass	500.0	[<SAT (Repaired)><Confirmed>]	
3	251.0	31479	257510	CID0173	251	0.20	Pass	500.0	[<SAT (Repaired)><Confirmed>]	

Sample 37: CID0173_EST1573_CC8_O01.fsa Run date and time: 09/21/2024 - 03:00:19 -> 09/21/2024 - 03:27:31

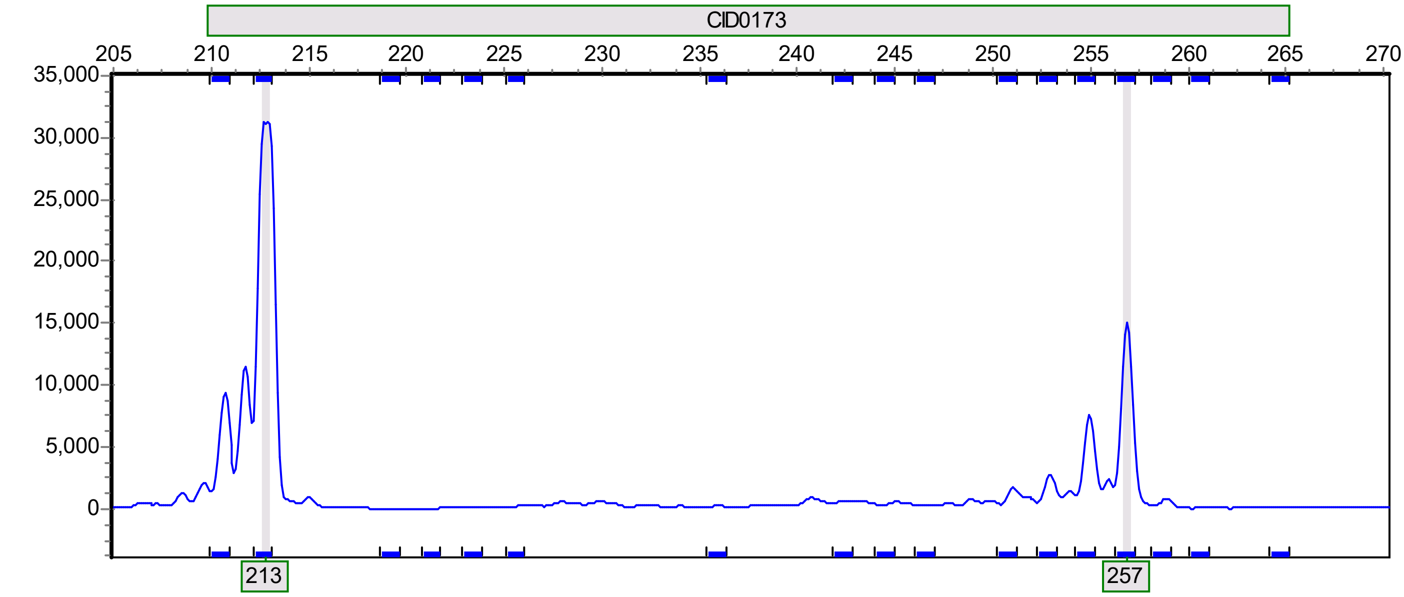

No	Size	Height	Area	Marker	Allele	Difference	Quality	Score	Allele Comments	Sample Comments
1	160.3	20509	129745	EST1573	160	0.00	Pass	500.0	[<Deleted>]	
2	170.8	43169	332967	EST1573	171	0.20	Pass	500.0	[<SAT (Repaired)><Confirmed>]	
3	211.8	11558	72788	CID0173	212	0.20	Undetermined	500.0	[<Deleted>]	
4	212.8	31026	295585	CID0173	213	0.10	Pass	500.0	[<SAT (Repaired)><Confirmed>]	
5	256.9	15106	96457	CID0173	257	0.10	Pass	500.0	[<Confirmed>]	

Sample 38: CID0173_EST1573_CC9_A03.fsa Run date and time: 09/21/2024 - 03:00:19 -> 09/21/2024 - 03:27:31

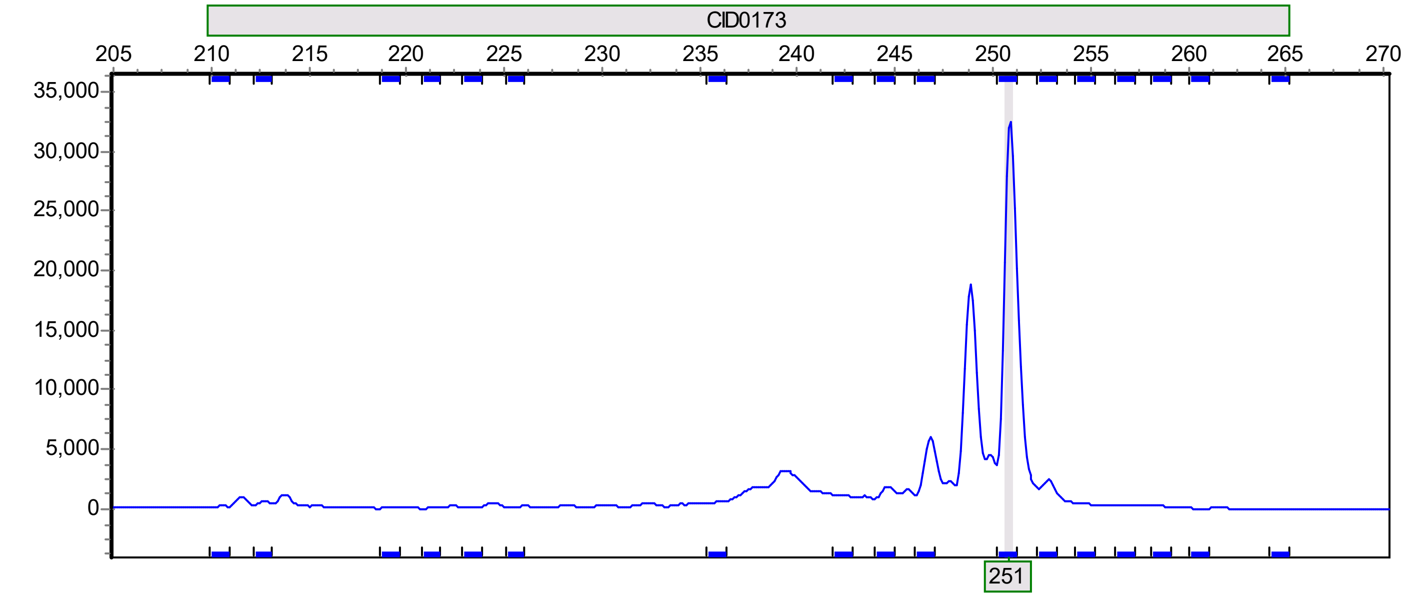

No	Size	Height	Area	Marker	Allele	Difference	Quality	Score	Allele Comments	Sample Comments
1	163.7	13676	105603	EST1573	164	0.30	Pass	500.0	[<Deleted>]	
2	172.7	39965	344818	EST1573	173	0.20	Pass	500.0	[<SAT (Repaired)><Confirmed>]	
3	250.9	32463	234416	CID0173	251	0.10	Pass	500.0	[<SAT (Repaired)><Confirmed>]	

Sample 39: CID0173_EST1573_CY10_O11.fsa Run date and time: 09/21/2024 - 03:00:19 -> 09/21/2024 - 03:27:31

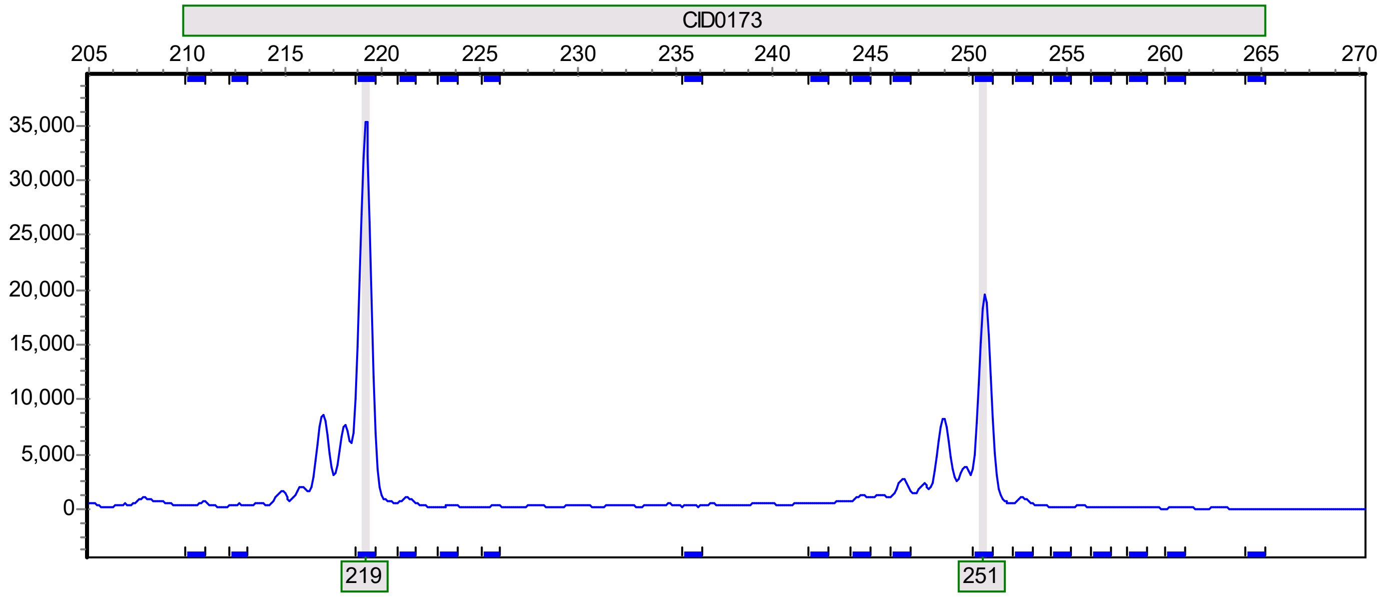

No	Size	Height	Area	Marker	Allele	Difference	Quality	Score	Allele Comments	Sample Comments
1	172.5	41262	291651	EST1573	173	0.00	Pass	500.0	[<SAT (Repaired)><Confirmed>]	
2	182.0	19744	129509	EST1573	182	0.00	Pass	500.0	[<Confirmed>]	
3	219.2	35338	271574	CID0173	219	0.00	Pass	500.0	[<SAT (Repaired)><Confirmed>]	
4	250.8	19580	138685	CID0173	251	0.00	Pass	500.0	[<Confirmed>]	

Sample 40: CID0173_EST1573_CY11_A13.fsa Run date and time: 09/21/2024 - 03:00:19 -> 09/21/2024 - 03:27:31

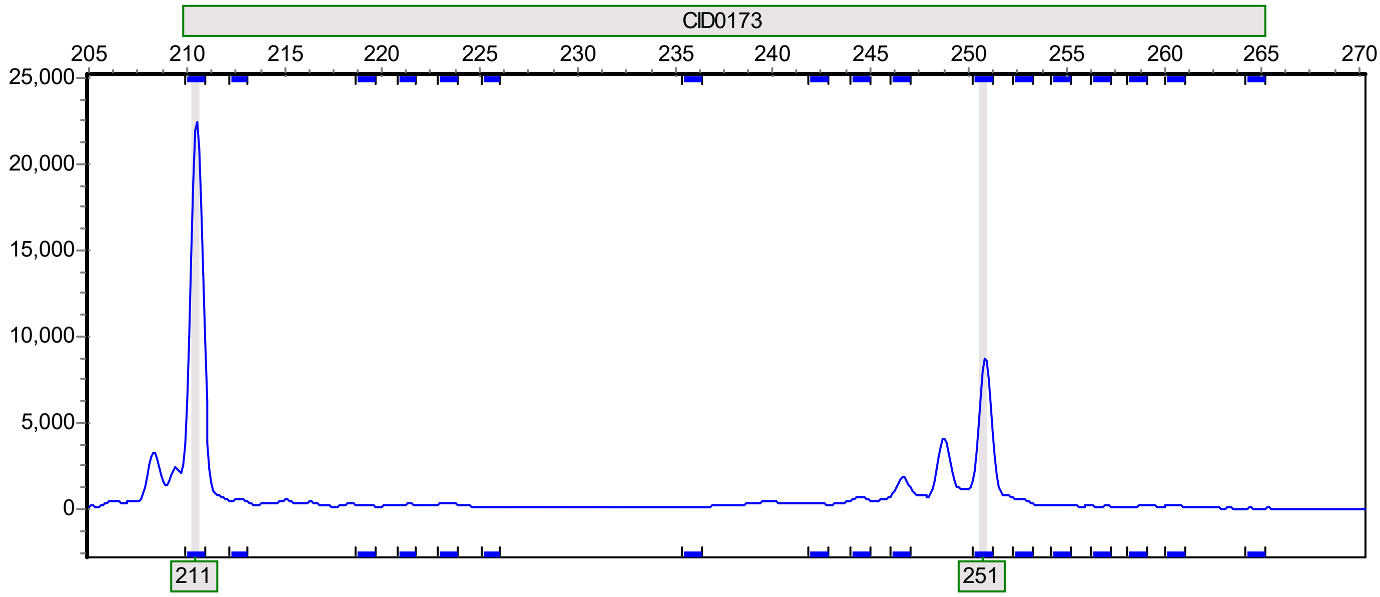

No	Size	Height	Area	Marker	Allele	Difference	Quality	Score	Allele Comments	Sample Comments
1	174.4	13647	95869	EST1573	174	0.00	Pass	500.0	[<Confirmed>]	
2	182.1	8044	53509	EST1573	182	0.10	Pass	500.0	[<Confirmed>]	
3	210.5	22412	163644	CID0173	211	0.00	Pass	500.0	[<Confirmed>]	
4	250.8	8682	65183	CID0173	251	0.00	Pass	500.0	[<Confirmed>]	

Sample 41: CID0173_EST1573_CY12_C13.fsa Run date and time: 09/21/2024 - 03:00:19 -> 09/21/2024 - 03:27:31

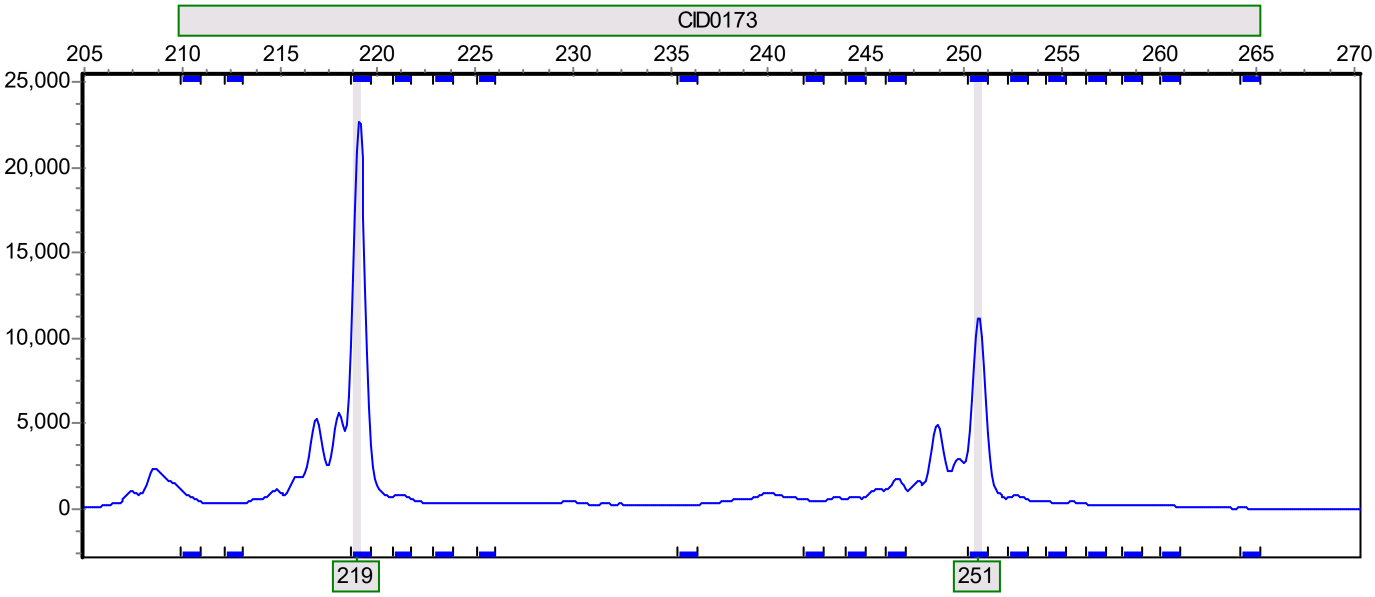

No	Size	Height	Area	Marker	Allele	Difference	Quality	Score	Allele Comments	Sample Comments
1	172.5	29770	200685	EST1573	173	0.00	Pass	500.0	[<Confirmed>]	
2	181.9	11685	83486	EST1573	182	0.10	Pass	500.0	[<Confirmed>]	
3	219.0	22694	178956	CID0173	219	0.20	Pass	500.0	[<Confirmed>]	
4	250.8	11210	87740	CID0173	251	0.00	Pass	500.0	[<Confirmed>]	

Sample 42: CID0173_EST1573_CY13_E13.fsa Run date and time: 09/21/2024 - 03:00:19 -> 09/21/2024 - 03:27:31

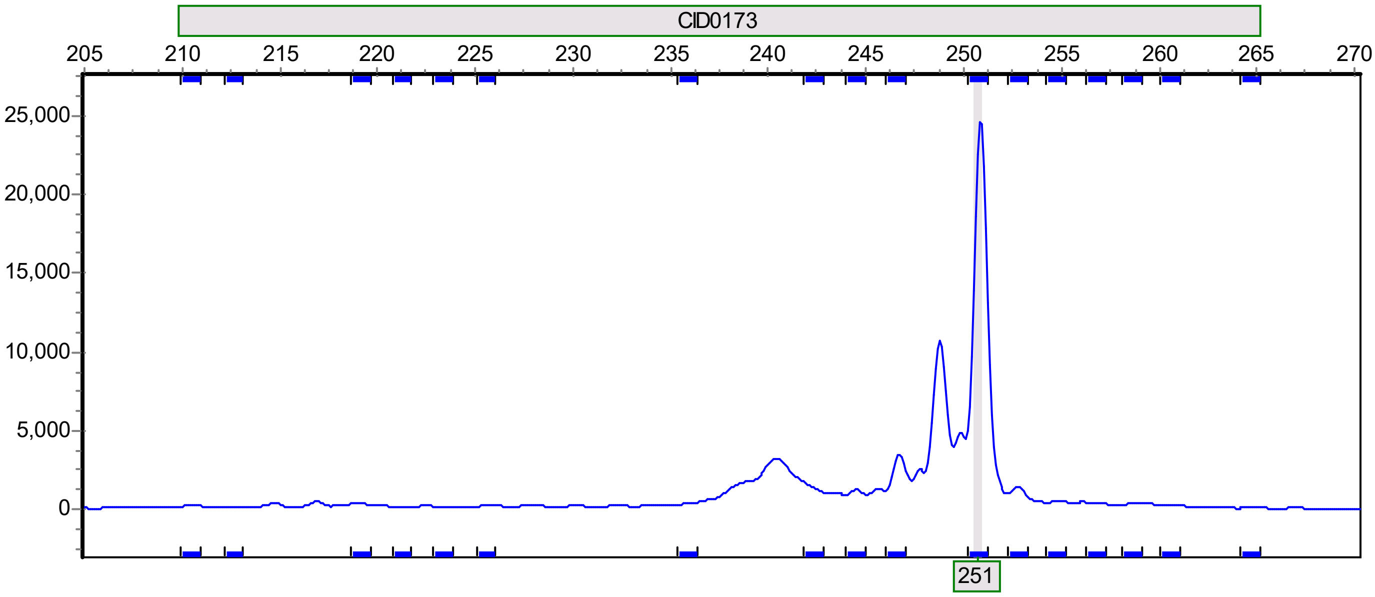

No	Size	Height	Area	Marker	Allele	Difference	Quality	Score	Allele Comments	Sample Comments
1	174.5	19943	139970	EST1573	174	0.10	Pass	500.0	[<Confirmed>]	
2	182.1	12931	90998	EST1573	182	0.10	Pass	500.0	[<Confirmed>]	
3	250.8	24625	187992	CID0173	251	0.00	Pass	500.0	[<Confirmed>]	

Sample 43: CID0173_EST1573_CY14_G13.fsa Run date and time: 09/21/2024 - 03:00:19 -> 09/21/2024 - 03:27:31

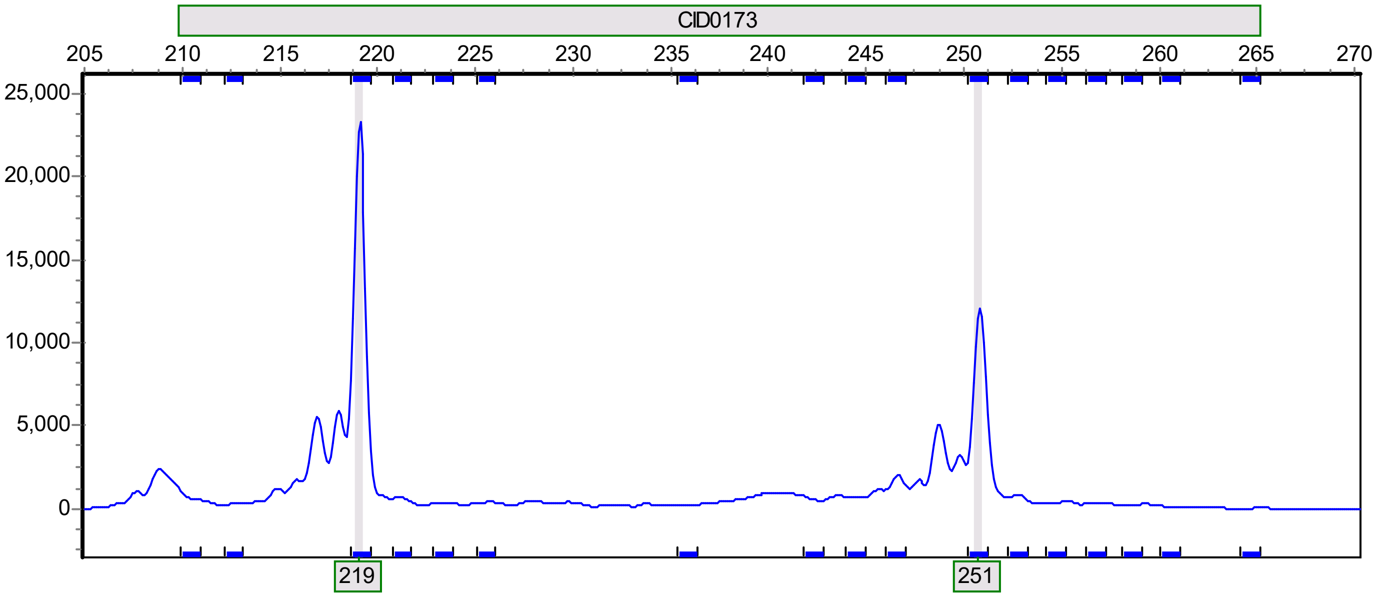

No	Size	Height	Area	Marker	Allele	Difference	Quality	Score	Allele Comments	Sample Comments
1	174.3	21997	152913	EST1573	174	0.10	Pass	500.0	[<Confirmed>]	
2	178.1	15898	110217	EST1573	178	0.10	Pass	500.0	[<Confirmed>]	
3	219.1	23270	167175	CID0173	219	0.10	Pass	500.0	[<Confirmed>]	
4	250.8	12104	90738	CID0173	251	0.00	Pass	500.0	[<Confirmed>]	

Sample 44: CID0173_EST1573_CY15_I13.fsa Run date and time: 09/21/2024 - 03:00:19 -> 09/21/2024 - 03:27:31

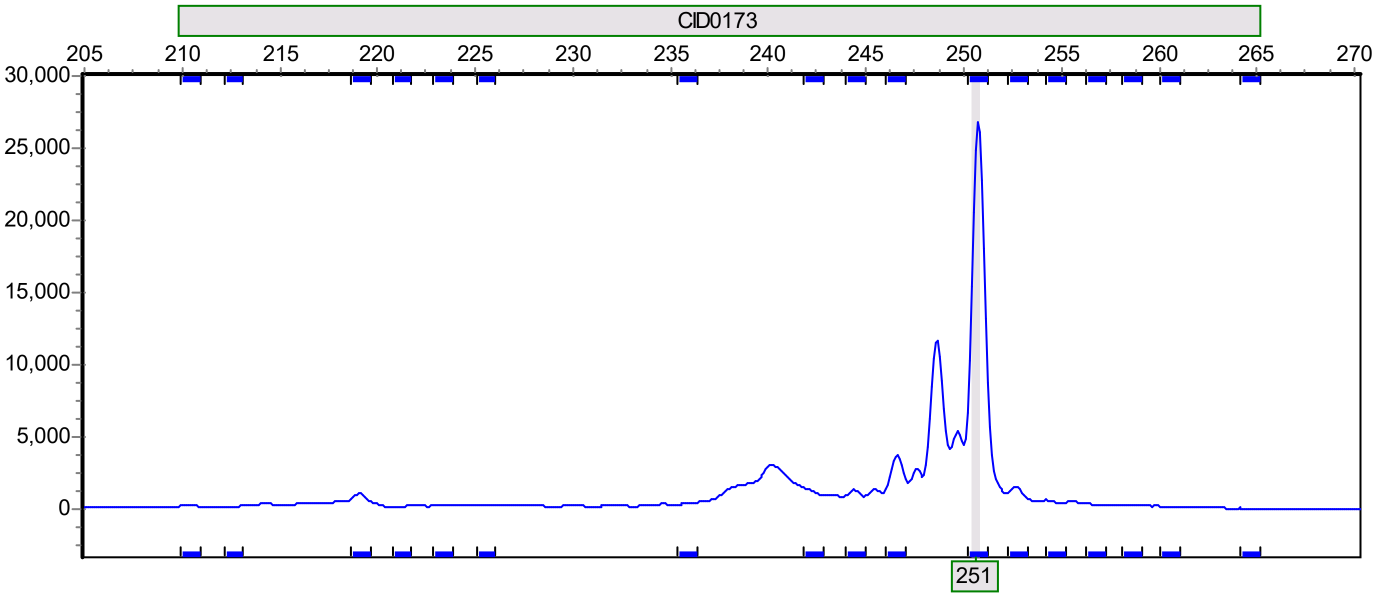

No	Size	Height	Area	Marker	Allele	Difference	Quality	Score	Allele Comments	Sample Comments
1	172.5	31187	213864	EST1573	173	0.00	Pass	500.0	[<Confirmed>]	
2	178.2	16196	110934	EST1573	178	0.00	Pass	500.0	[<Confirmed>]	
3	250.7	26825	198159	CID0173	251	0.10	Pass	500.0	[<Confirmed>]	

Sample 45: CID0173_EST1573_CY16_K13.fsa Run date and time: 09/21/2024 - 03:00:19 -> 09/21/2024 - 03:27:31

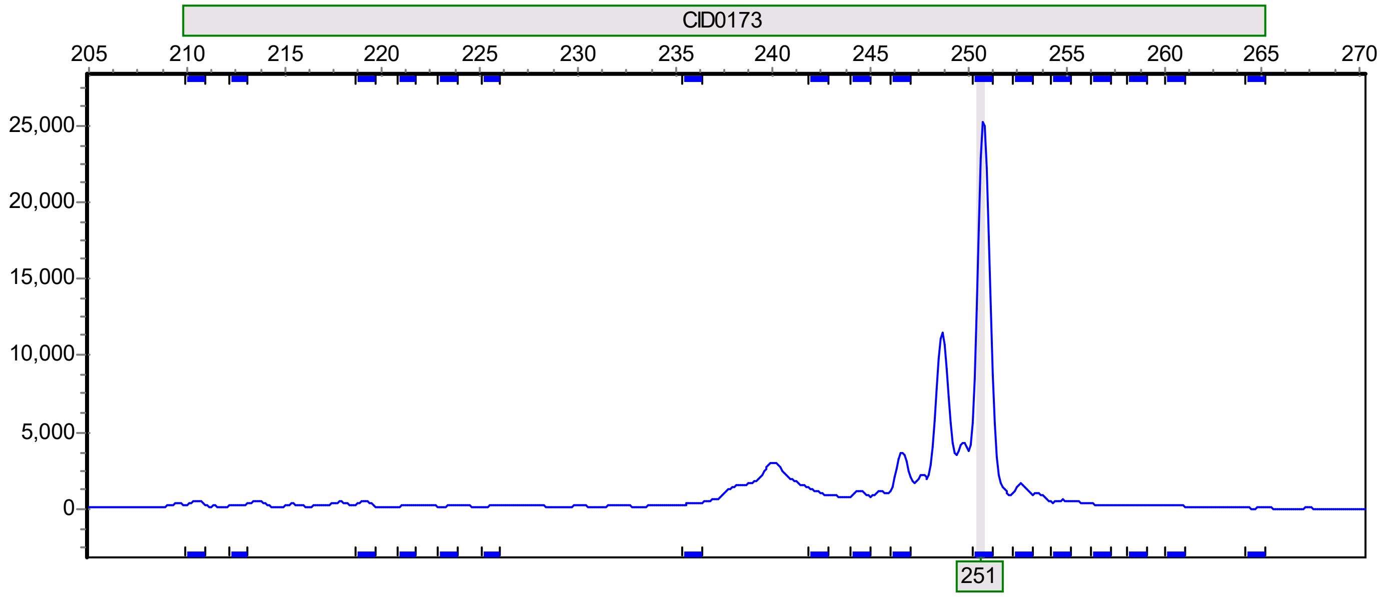

No	Size	Height	Area	Marker	Allele	Difference	Quality	Score	Allele Comments	Sample Comments
1	172.5	30335	211782	EST1573	173	0.00	Pass	500.0	[<Confirmed>]	
2	182.0	12737	86982	EST1573	182	0.00	Pass	500.0	[<Confirmed>]	
3	250.7	25231	178149	CID0173	251	0.10	Pass	500.0	[<Confirmed>]	

Sample 46: CID0173_EST1573_CY17_M13.fsa Run date and time: 09/21/2024 - 03:00:19 -> 09/21/2024 - 03:27:31

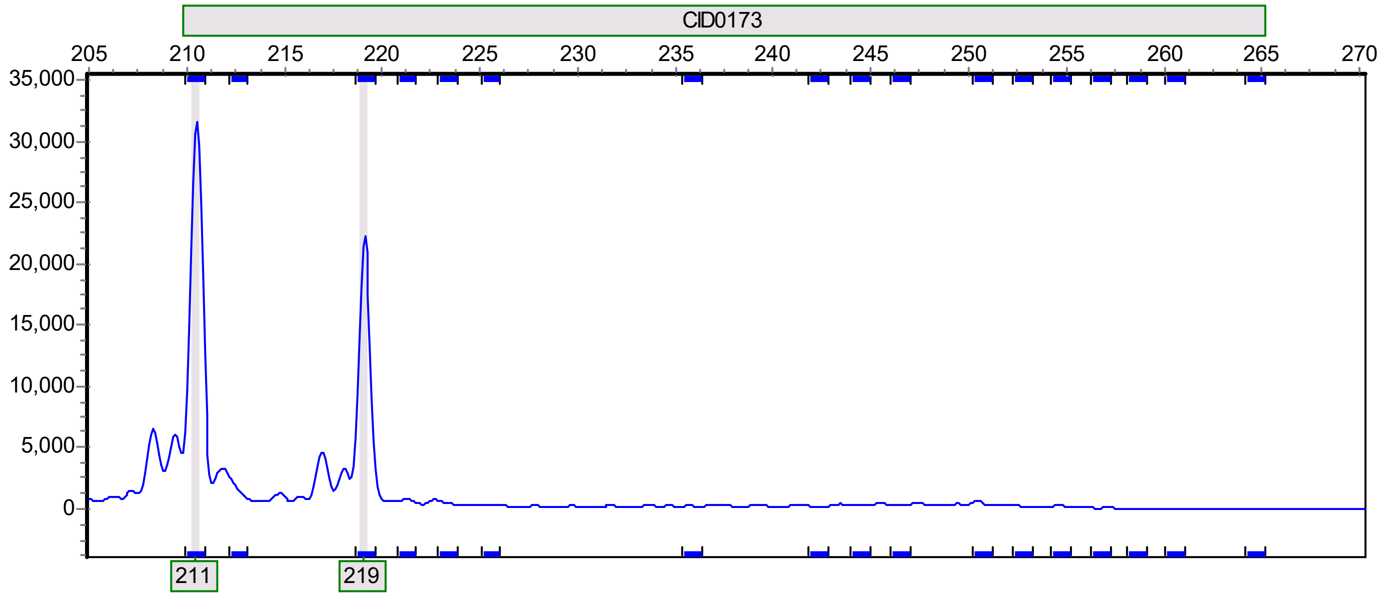

No	Size	Height	Area	Marker	Allele	Difference	Quality	Score	Allele Comments	Sample Comments
1	174.4	18563	126146	EST1573	174	0.00	Pass	500.0	[<Confirmed>]	
2	182.0	10243	67904	EST1573	182	0.00	Pass	500.0	[<Confirmed>]	
3	210.5	31556	230206	CID0173	211	0.00	Pass	500.0	[<Confirmed>]	
4	219.1	22223	156782	CID0173	219	0.10	Pass	500.0	[<Confirmed>]	

Allele Report

Sample 47: CID0173_EST1573_CY18_O13.fsa Run date and time: 09/21/2024 - 03:00:19 -> 09/21/2024 - 03:27:31

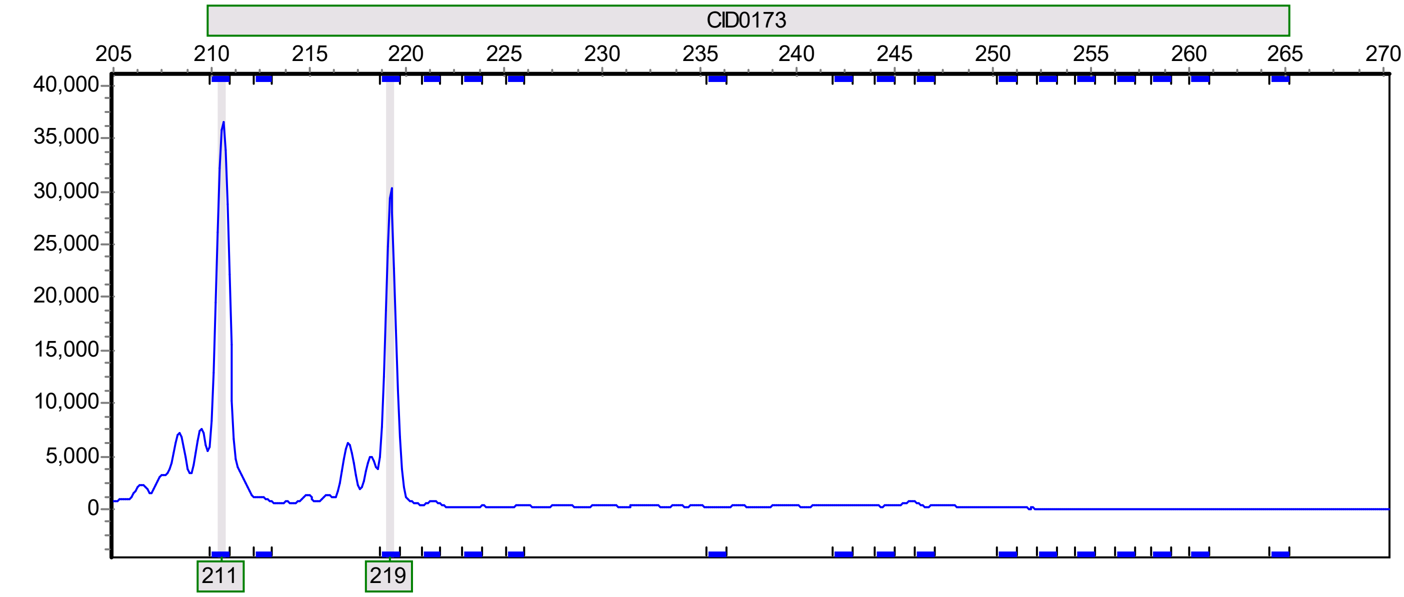

No	Size	Height	Area	Marker	Allele	Difference	Quality	Score	Allele Comments	Sample Comments
1	172.7	31682	225818	EST1573	173	0.20	Pass	500.0	[<Confirmed>]	
2	178.3	16200	104260	EST1573	178	0.10	Pass	500.0	[<Confirmed>]	
3	210.6	36573	283555	CID0173	211	0.10	Pass	500.0	[<SAT (Repaired)><Confirmed>]	
4	219.2	30240	207103	CID0173	219	0.00	Pass	500.0	[<Confirmed>]	

Sample 48: CID0173_EST1573_CY19_A15.fsa Run date and time: 09/21/2024 - 03:00:19 -> 09/21/2024 - 03:27:31

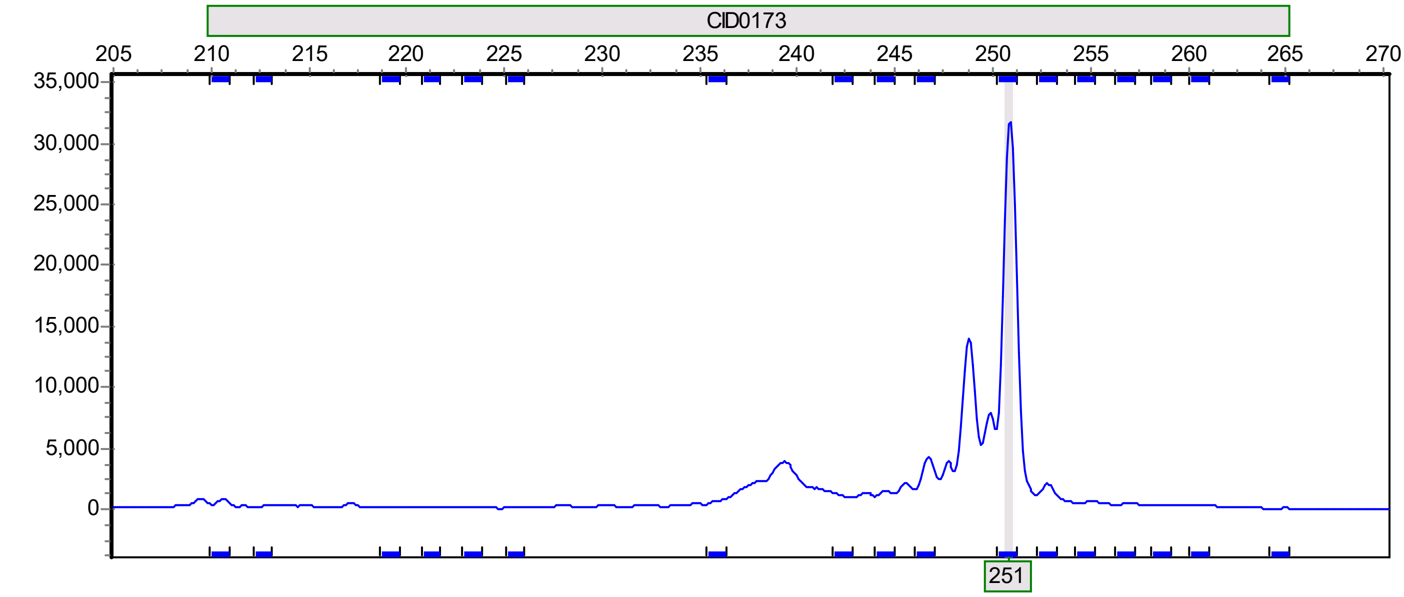

No	Size	Height	Area	Marker	Allele	Difference	Quality	Score	Allele Comments	Sample Comments
1	174.4	30144	198808	EST1573	174	0.00	Pass	500.0	[<Confirmed>]	
2	178.2	23056	151679	EST1573	178	0.00	Pass	500.0	[<Confirmed>]	
3	250.9	31740	246780	CID0173	251	0.10	Pass	500.0	[<Confirmed>]	

Allele Report

Sample 49: CID0173_EST1573_CY1_M09.fsa Run date and time: 09/21/2024 - 03:00:19 -> 09/21/2024 - 03:27:31

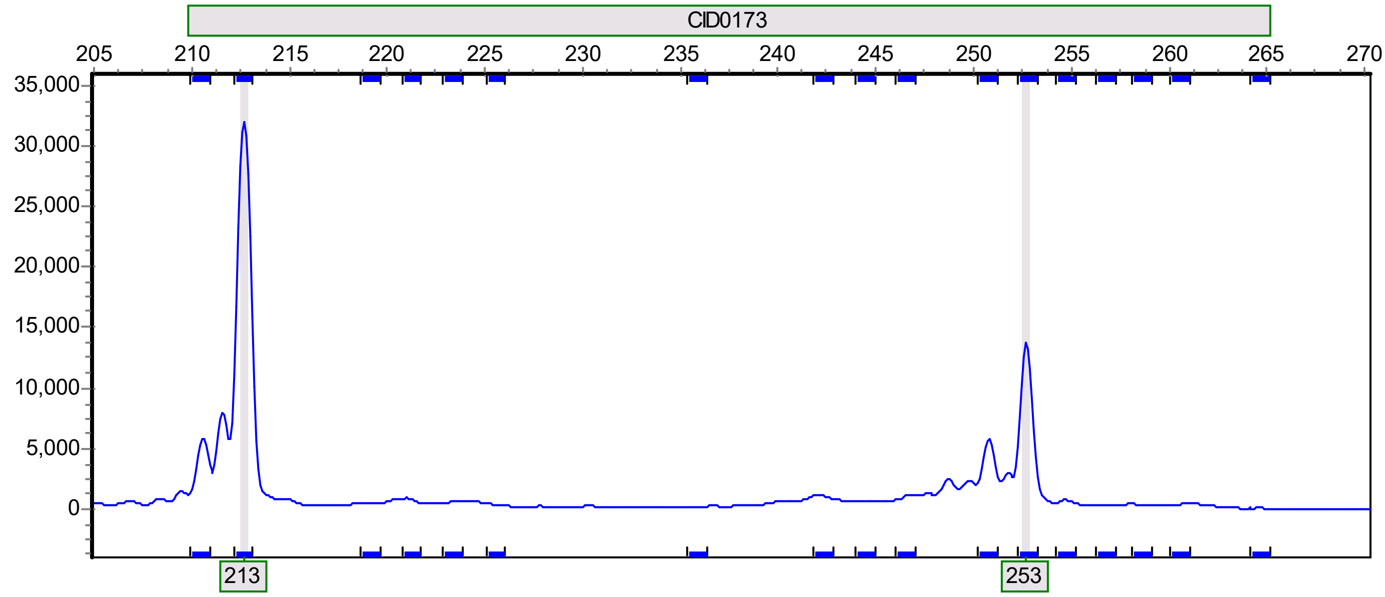

No	Size	Height	Area	Marker	Allele	Difference	Quality	Score	Allele Comments	Sample Comments
1	170.6	42467	333078	EST1573	171	0.00	Pass	500.0	[<SAT (Repaired)><Confirmed>]	
2	212.7	31955	257796	CID0173	213	0.00	Pass	500.0	[<Confirmed>]	
3	252.7	13656	99784	CID0173	253	0.10	Pass	500.0	[<Confirmed>]	

Sample 50: CID0173_EST1573_CY20_C15.fsa Run date and time: 09/21/2024 - 03:00:19 -> 09/21/2024 - 03:27:31

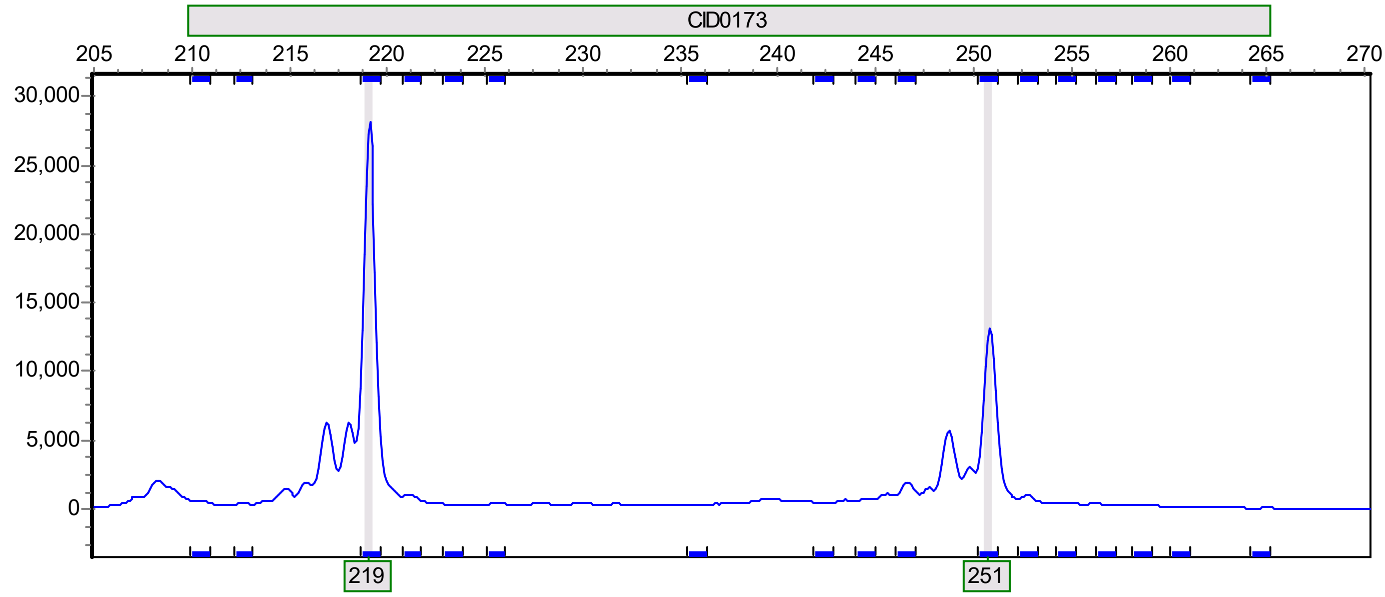

No	Size	Height	Area	Marker	Allele	Difference	Quality	Score	Allele Comments	Sample Comments
1	172.6	32351	236949	EST1573	173	0.10	Pass	500.0	[<Confirmed>]	
2	182.0	13879	97720	EST1573	182	0.00	Pass	500.0	[<Confirmed>]	
3	219.1	28141	206158	CID0173	219	0.10	Pass	500.0	[<Confirmed>]	
4	250.8	13114	97921	CID0173	251	0.00	Pass	500.0	[<Confirmed>]	

Allele Report

Sample 51: CID0173_EST1573_CY21_E15.fsa Run date and time: 09/21/2024 - 03:00:19 -> 09/21/2024 - 03:27:31

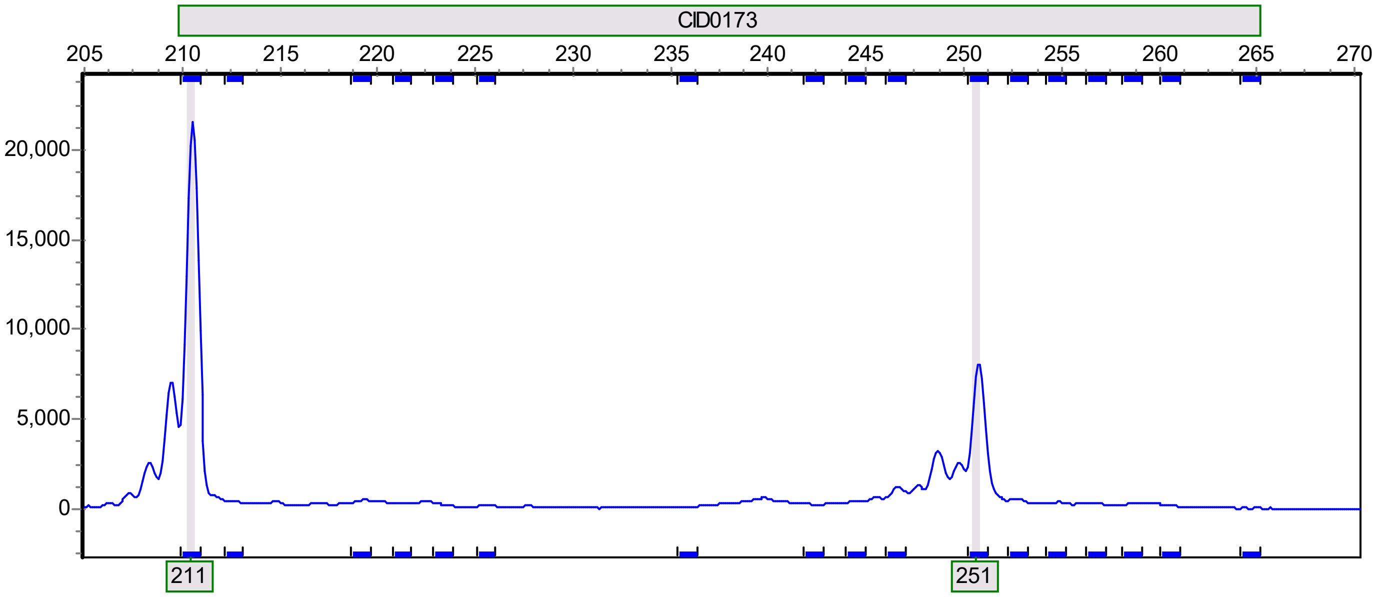

No	Size	Height	Area	Marker	Allele	Difference	Quality	Score	Allele Comments	Sample Comments
1	174.3	16447	114761	EST1573	174	0.10	Pass	500.0	[<Confirmed>]	
2	182.0	10047	69532	EST1573	182	0.00	Pass	500.0	[<Confirmed>]	
3	209.4	7011	48069	CID0173	210	0.10	Undetermined	500.0	[<Deleted>]	
4	210.5	21532	156167	CID0173	211	0.00	Pass	500.0	[<Confirmed>]	
5	250.7	8065	60149	CID0173	251	0.10	Pass	500.0	[<Confirmed>]	

Sample 52: CID0173_EST1573_CY22_G15.fsa Run date and time: 09/21/2024 - 03:00:19 -> 09/21/2024 - 03:27:31

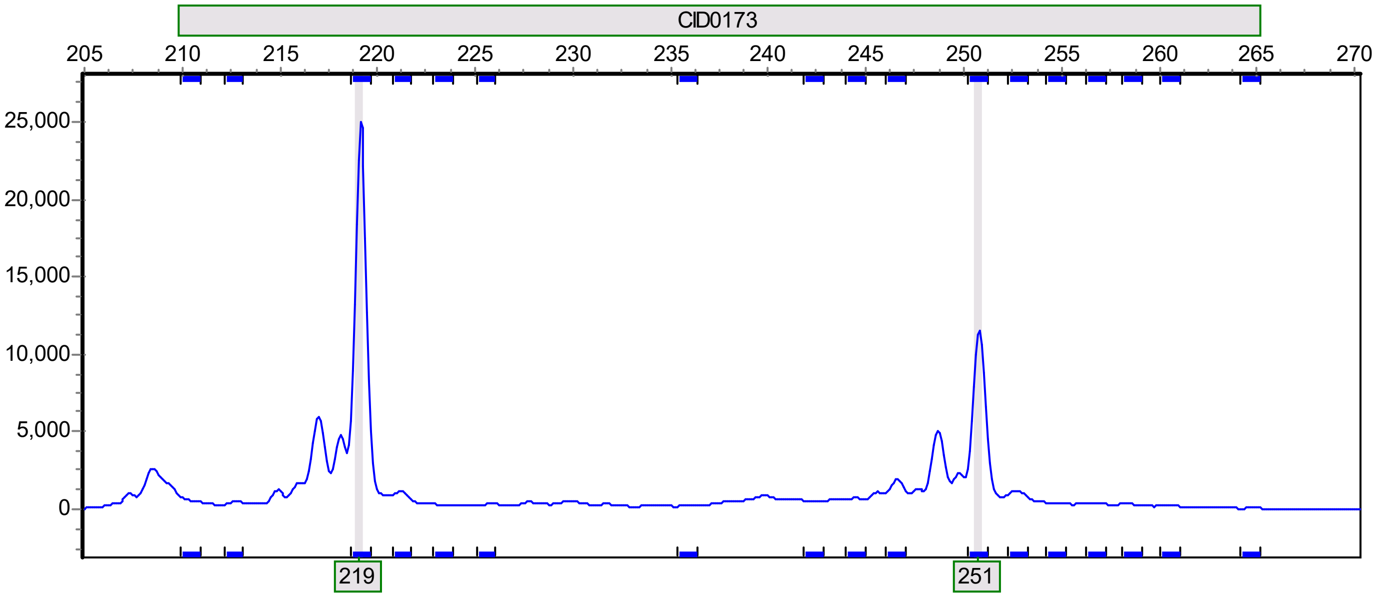

No	Size	Height	Area	Marker	Allele	Difference	Quality	Score	Allele Comments	Sample Comments
1	172.4	30327	205906	EST1573	173	0.10	Pass	500.0	[<Confirmed>]	
2	181.9	11458	79358	EST1573	182	0.10	Pass	500.0	[<Confirmed>]	
3	219.1	25034	181378	CID0173	219	0.10	Pass	500.0	[<Confirmed>]	
4	250.8	11548	87077	CID0173	251	0.00	Pass	500.0	[<Confirmed>]	

Allele Report

Sample 53: CID0173_EST1573_CY23_I15.fsa Run date and time: 09/21/2024 - 03:00:19 -> 09/21/2024 - 03:27:31

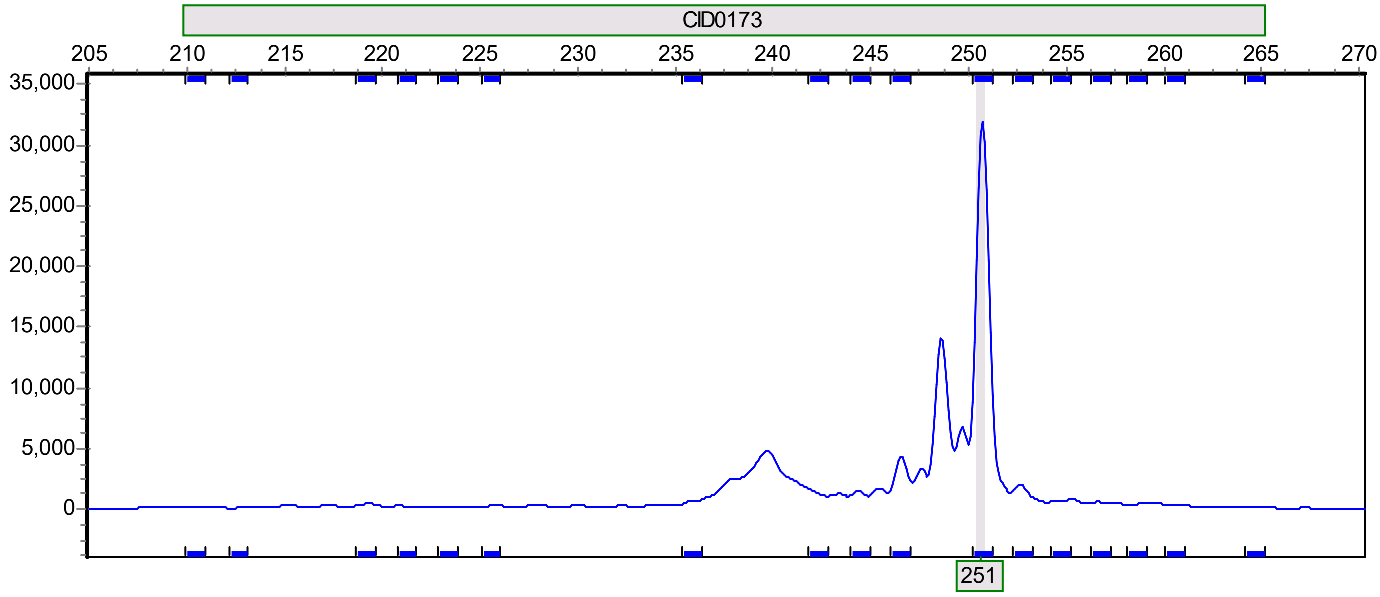

No	Size	Height	Area	Marker	Allele	Difference	Quality	Score	Allele Comments	Sample Comments
1	174.3	26621	192573	EST1573	174	0.10	Pass	500.0	[<Confirmed>]	
2	181.9	17090	118440	EST1573	182	0.10	Pass	500.0	[<Confirmed>]	
3	250.7	31907	237191	CID0173	251	0.10	Pass	500.0	[<Confirmed>]	

Sample 54: CID0173_EST1573_CY24_K15.fsa Run date and time: 09/21/2024 - 03:00:19 -> 09/21/2024 - 03:27:31

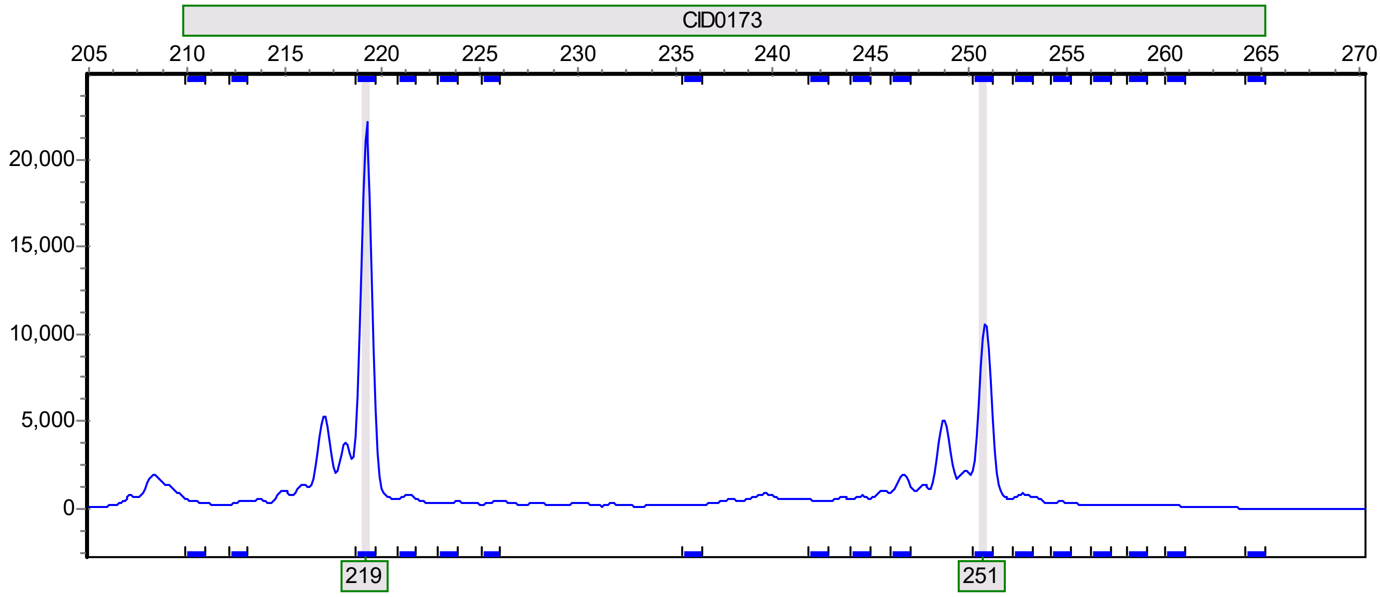

No	Size	Height	Area	Marker	Allele	Difference	Quality	Score	Allele Comments	Sample Comments
1	174.4	19661	132286	EST1573	174	0.00	Pass	500.0	[<Confirmed>]	
2	178.3	14736	98632	EST1573	178	0.10	Pass	500.0	[<Confirmed>]	
3	219.2	22126	160049	CID0173	219	0.00	Pass	500.0	[<Confirmed>]	
4	250.8	10595	78702	CID0173	251	0.00	Pass	500.0	[<Confirmed>]	

Allele Report

Sample 55: CID0173_EST1573_CY25_M15.fsa Run date and time: 09/21/2024 - 03:00:19 -> 09/21/2024 - 03:27:31

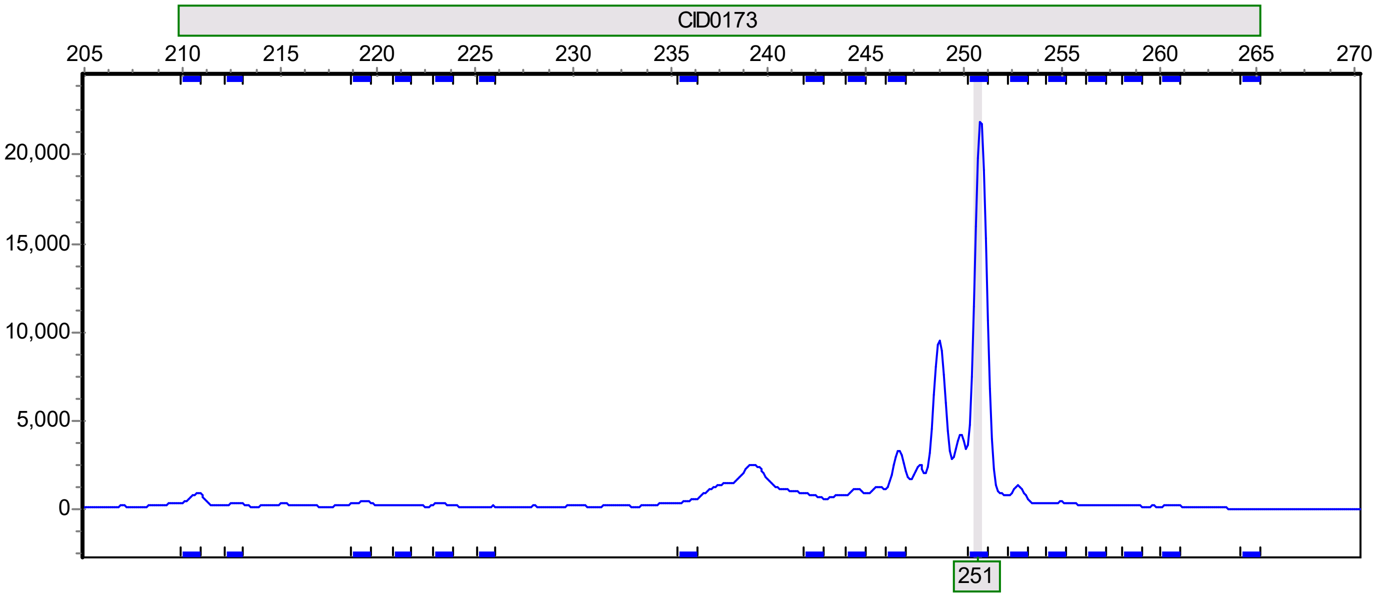

No	Size	Height	Area	Marker	Allele	Difference	Quality	Score	Allele Comments	Sample Comments
1	172.4	24960	170532	EST1573	173	0.10	Pass	500.0	[<Confirmed>]	
2	178.1	13355	87468	EST1573	178	0.10	Pass	500.0	[<Confirmed>]	
3	250.8	21865	150705	CID0173	251	0.00	Pass	500.0	[<Confirmed>]	

Sample 56: CID0173_EST1573_CY26_O15.fsa Run date and time: 09/21/2024 - 03:00:19 -> 09/21/2024 - 03:27:31

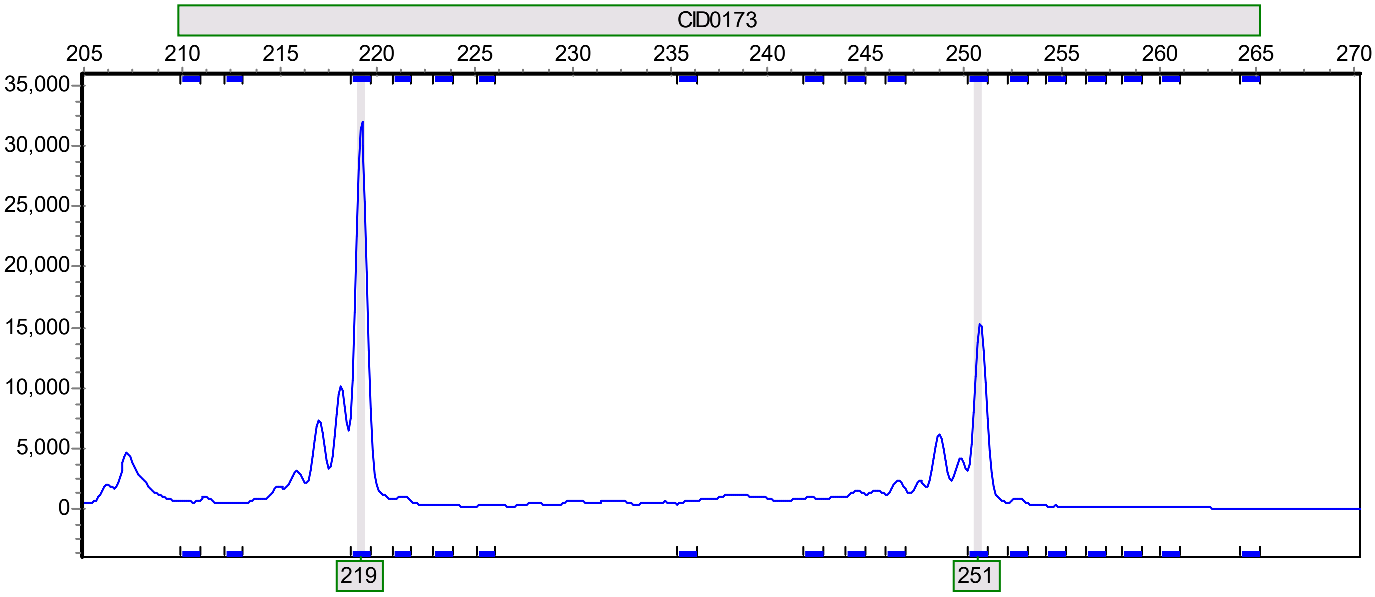

No	Size	Height	Area	Marker	Allele	Difference	Quality	Score	Allele Comments	Sample Comments
1	170.6	38768	273576	EST1573	171	0.00	Pass	500.0	[<SAT (Repaired)><Confirmed>]	
2	172.5	31552	225171	EST1573	173	0.00	Pass	500.0	[<Confirmed>]	
3	218.1	10045	69350	CID0173	218	0.00	Undetermined	500.0	[<Deleted>]	
4	219.2	32037	236218	CID0173	219	0.00	Pass	500.0	[<Confirmed>]	
5	250.8	15211	104942	CID0173	251	0.00	Pass	500.0	[<Confirmed>]	

Allele Report

Sample 57: CID0173_EST1573_CY27_A17.fsa Run date and time: 09/21/2024 - 03:00:19 -> 09/21/2024 - 03:27:31

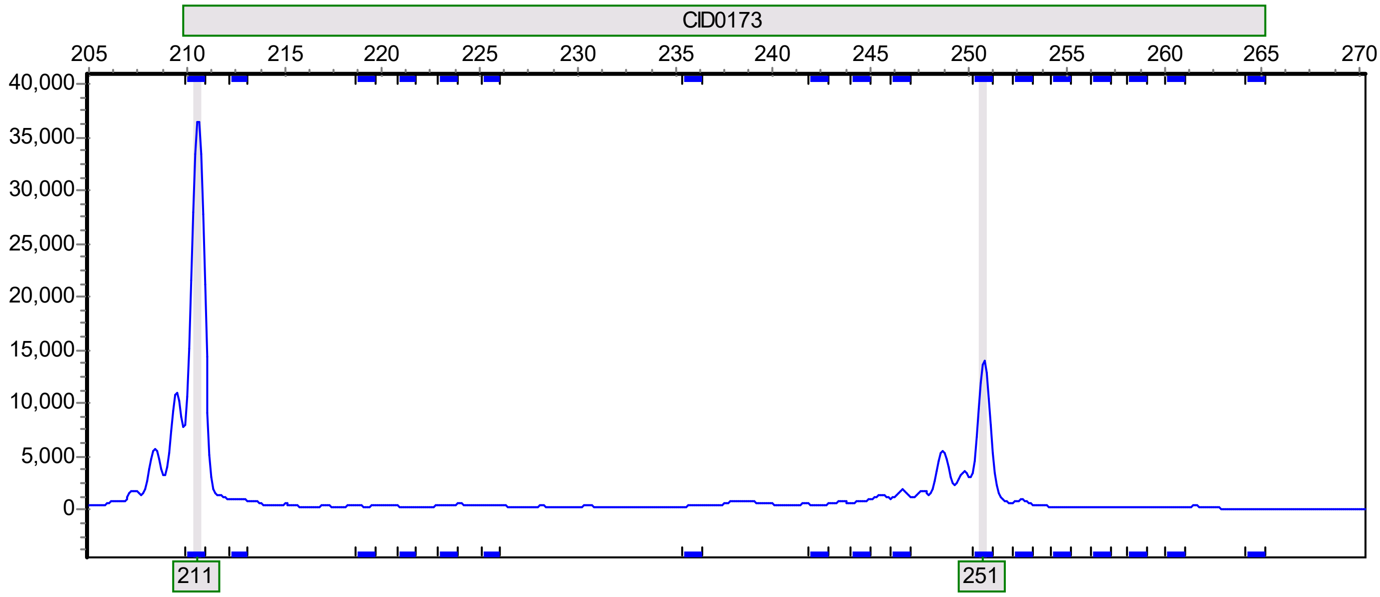

No	Size	Height	Area	Marker	Allele	Difference	Quality	Score	Allele Comments	Sample Comments
1	172.5	36526	264094	EST1573	173	0.00	Pass	500.0	[<SAT (Repaired)><Confirmed>]	
2	174.4	22838	153810	EST1573	174	0.00	Pass	500.0	[<Confirmed>]	
3	209.5	10981	74257	CID0173	210	0.00	Undetermined	500.0	[<Deleted>]	
4	210.6	36455	285577	CID0173	211	0.10	Pass	500.0	[<SAT (Repaired)><Confirmed>]	
5	250.8	13933	99687	CID0173	251	0.00	Pass	500.0	[<Confirmed>]	

Sample 58: CID0173_EST1573_CY28_C17.fsa Run date and time: 09/21/2024 - 03:00:19 -> 09/21/2024 - 03:27:31

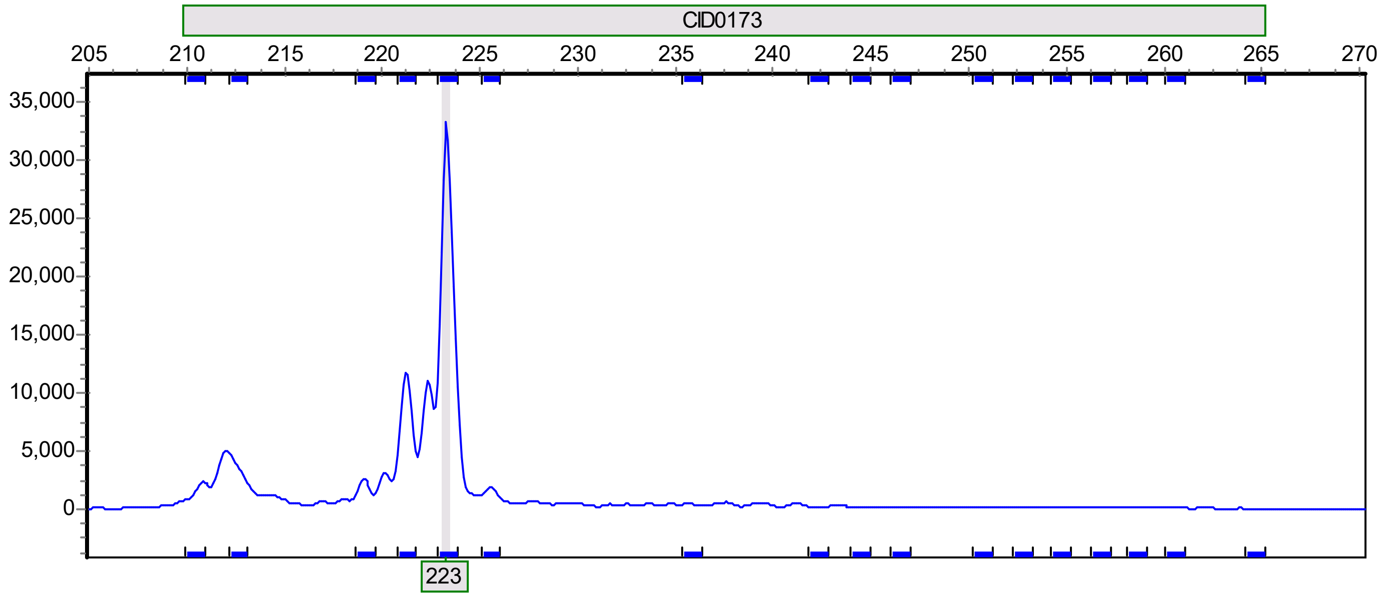

No	Size	Height	Area	Marker	Allele	Difference	Quality	Score	Allele Comments	Sample Comments
1	161.5	12731	90700	EST1573	161	0.20	Pass	500.0	[<Deleted>]	
2	170.4	37530	299558	EST1573	171	0.20	Pass	500.0	[<SAT (Repaired)><Confirmed>]	
3	222.3	10985	70216	CID0173	222	0.10	Pass	500.0	[<Deleted>]	
4	223.3	33325	275357	CID0173	223	0.10	Pass	500.0	[<SAT (Repaired)><Confirmed>]	

Allele Report

Sample 59: CID0173_EST1573_CY29_E17.fsa Run date and time: 09/21/2024 - 03:00:19 -> 09/21/2024 - 03:27:31

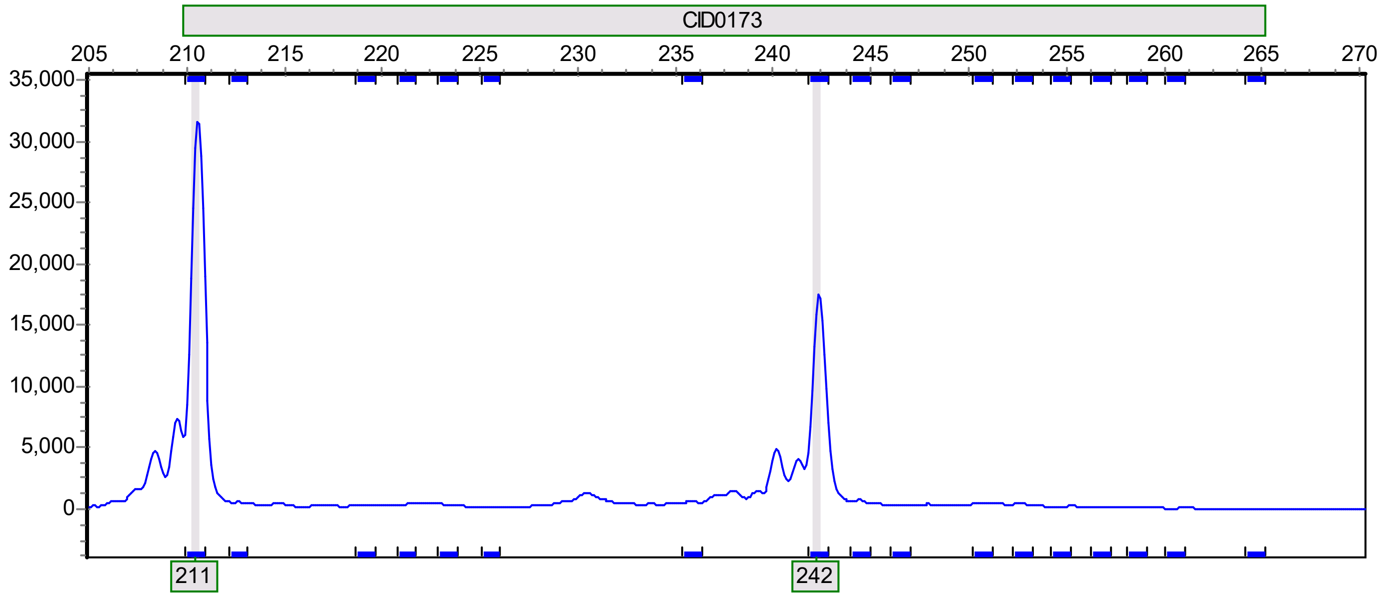

No	Size	Height	Area	Marker	Allele	Difference	Quality	Score	Allele Comments	Sample Comments
1	172.4	28640	194389	EST1573	173	0.10	Pass	500.0	[<Confirmed>]	
2	174.3	20113	138801	EST1573	174	0.10	Pass	500.0	[<Confirmed>]	
3	210.5	31625	256128	CID0173	211	0.00	Pass	500.0	[<Confirmed>]	
4	242.3	17495	135263	CID0173	242	0.10	Pass	500.0	[<Confirmed>]	

Sample 60: CID0173_EST1573_CY2_O09.fsa Run date and time: 09/21/2024 - 03:00:19 -> 09/21/2024 - 03:27:31

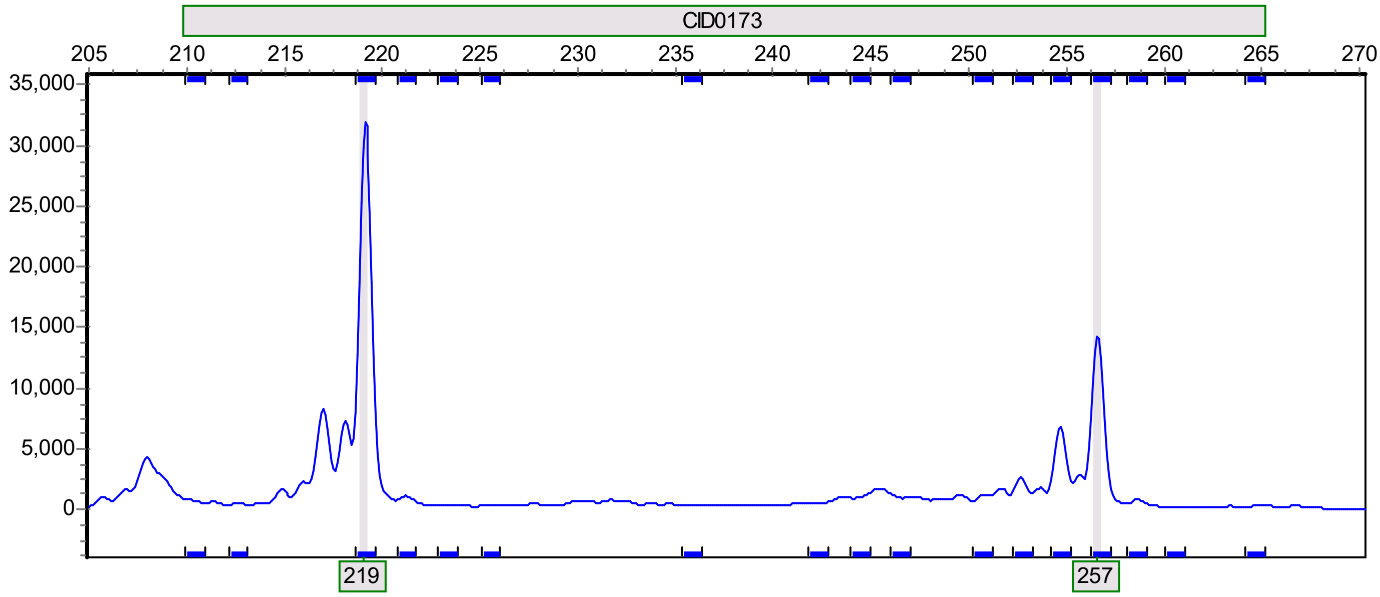

No	Size	Height	Area	Marker	Allele	Difference	Quality	Score	Allele Comments	Sample Comments
1	163.5	14795	124283	EST1573	164	0.50	Pass	500.0	[<Deleted>]	
2	172.5	40446	313173	EST1573	173	0.00	Pass	500.0	[<SAT (Repaired)><Confirmed>]	
3	219.1	31936	251510	CID0173	219	0.10	Pass	500.0	[<Confirmed>]	
4	256.6	14286	98414	CID0173	257	0.20	Pass	500.0	[<Confirmed>]	

Sample 61: CID0173_EST1573_CY30_G17.fsa Run date and time: 09/21/2024 - 03:00:19 -> 09/21/2024 - 03:27:31

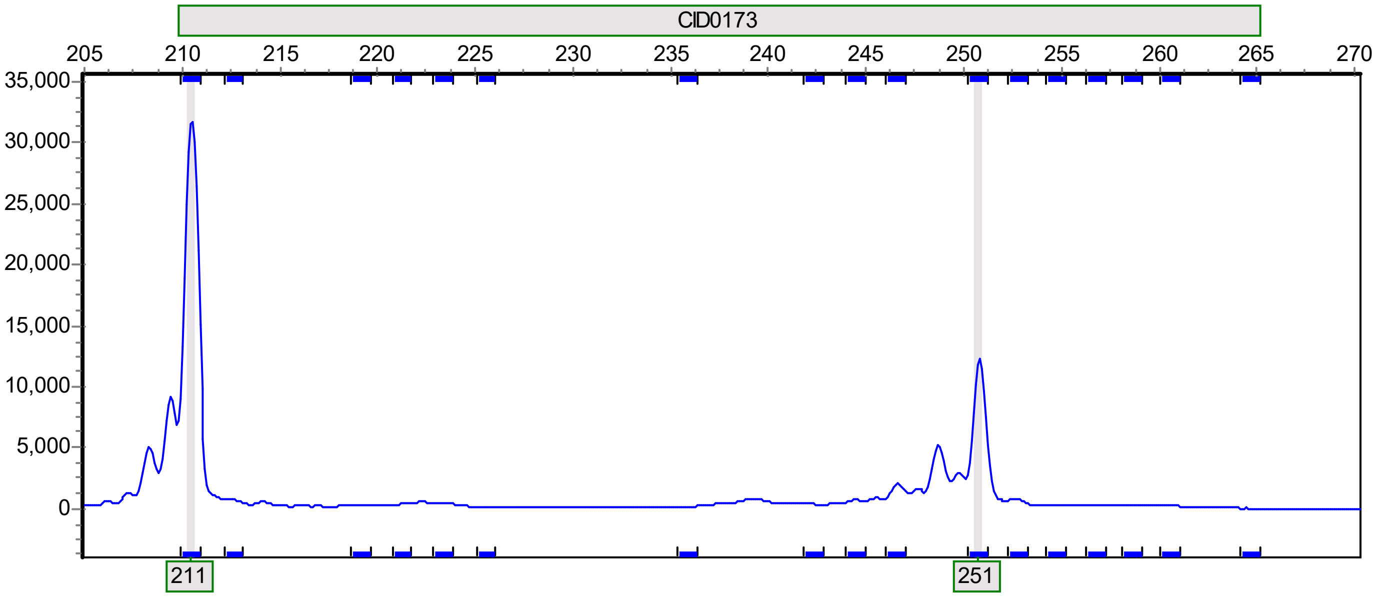

No	Size	Height	Area	Marker	Allele	Difference	Quality	Score	Allele Comments	Sample Comments
1	172.5	32629	231700	EST1573	173	0.00	Pass	500.0	[<SAT (Repaired)><Confirmed>]	
2	174.3	19765	135635	EST1573	174	0.10	Pass	500.0	[<Confirmed>]	
3	210.5	31723	262901	CID0173	211	0.00	Pass	500.0	[<Confirmed>]	
4	250.8	12280	89717	CID0173	251	0.00	Pass	500.0	[<Confirmed>]	

Sample 62: CID0173_EST1573_CY31_I17.fsa Run date and time: 09/21/2024 - 03:00:19 -> 09/21/2024 - 03:27:31

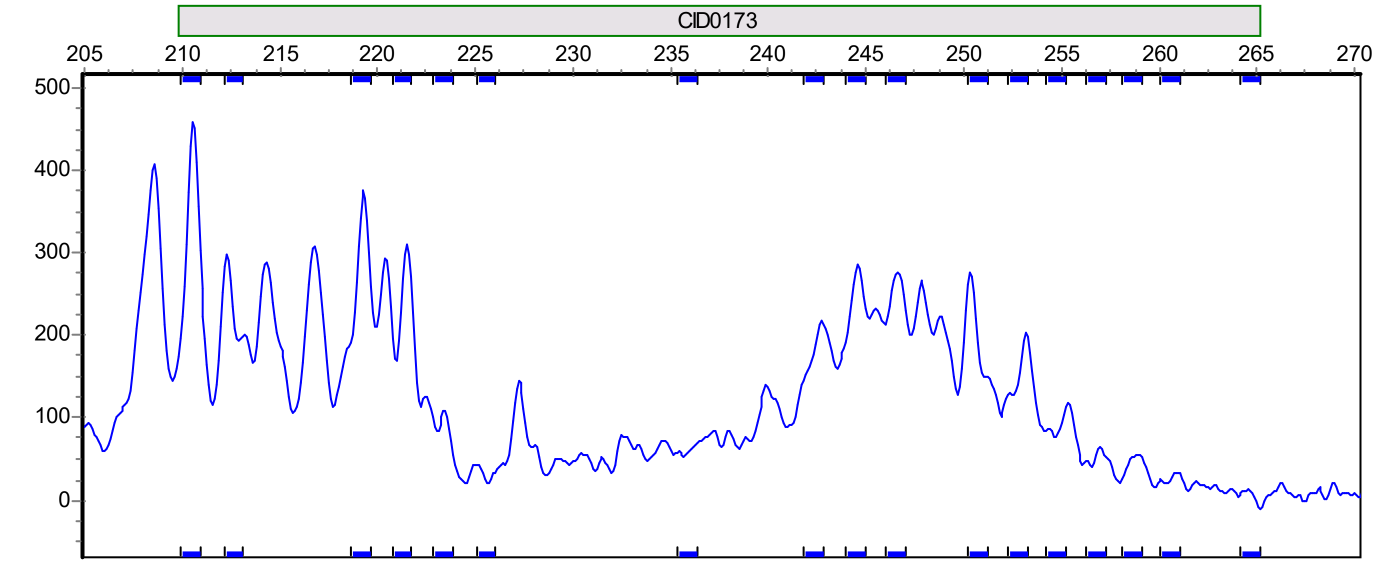

No	Size	Height	Area	Marker	Allele	Difference	Quality	Score	Allele Comments	Sample Comments
1	170.6	2729	21202	EST1573	171	0.00	Pass	427.5	[<Confirmed>]	
2	174.3	2169	16208	EST1573	174	0.10	Pass	316.7	[<Confirmed>]	
3	210.5	459	3818	CID0173	211	0.00	Pass	17.6	[<Deleted>]	
4	216.8	308	3457	CID0173	217	0.20	Undetermined	4.1	[<Deleted>]	
5	219.3	375	3573	CID0173	219	0.10	Undetermined	7.2	[<Deleted>]	
6	221.5	310	2586	CID0173	221	0.20	Pass	8.6	[<Deleted>]	

Sample 63: CID0173_EST1573_CY32_K17.fsa Run date and time: 09/21/2024 - 03:00:19 -> 09/21/2024 - 03:27:31

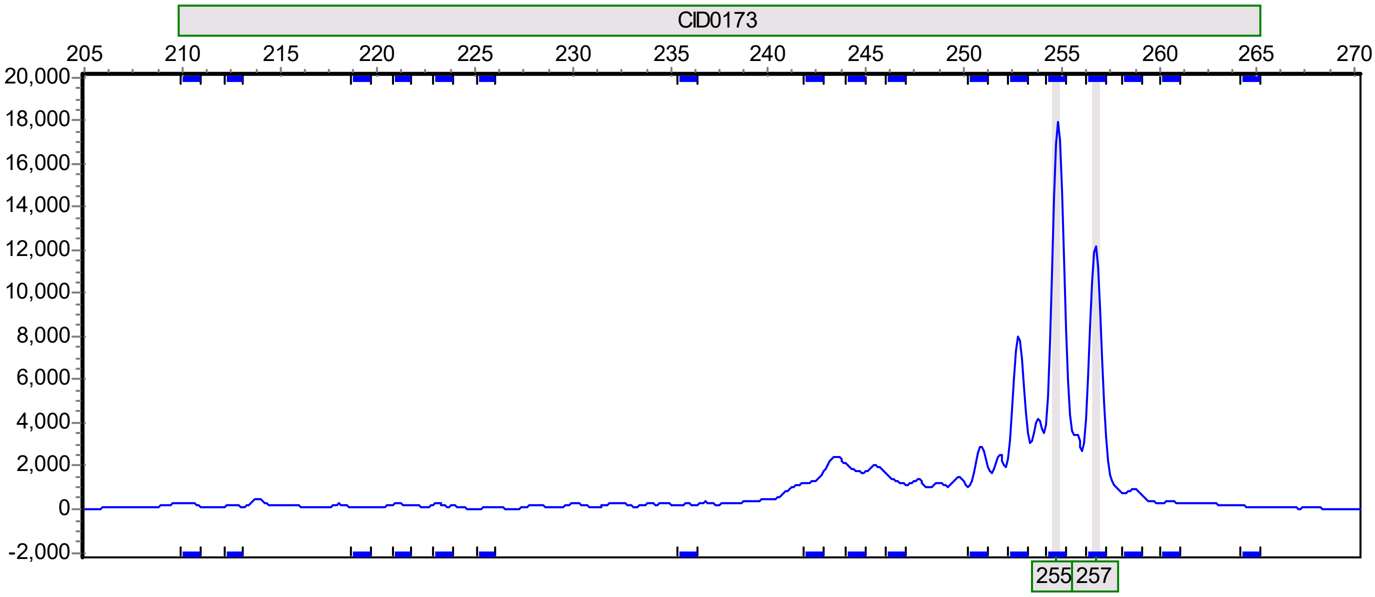

No	Size	Height	Area	Marker	Allele	Difference	Quality	Score	Allele Comments	Sample Comments
1	170.7	31452	218084	EST1573	171	0.10	Pass	500.0	[<Confirmed>]	
2	174.6	26407	176940	EST1573	174	0.20	Pass	500.0	[<Confirmed>]	
3	254.8	17922	130156	CID0173	255	0.00	Pass	500.0	[<Confirmed>]	
4	256.8	12171	89622	CID0173	257	0.00	Pass	500.0	[<Confirmed>]	

Sample 64: CID0173_EST1573_CY33_M17.fsa Run date and time: 09/21/2024 - 03:00:19 -> 09/21/2024 - 03:27:31

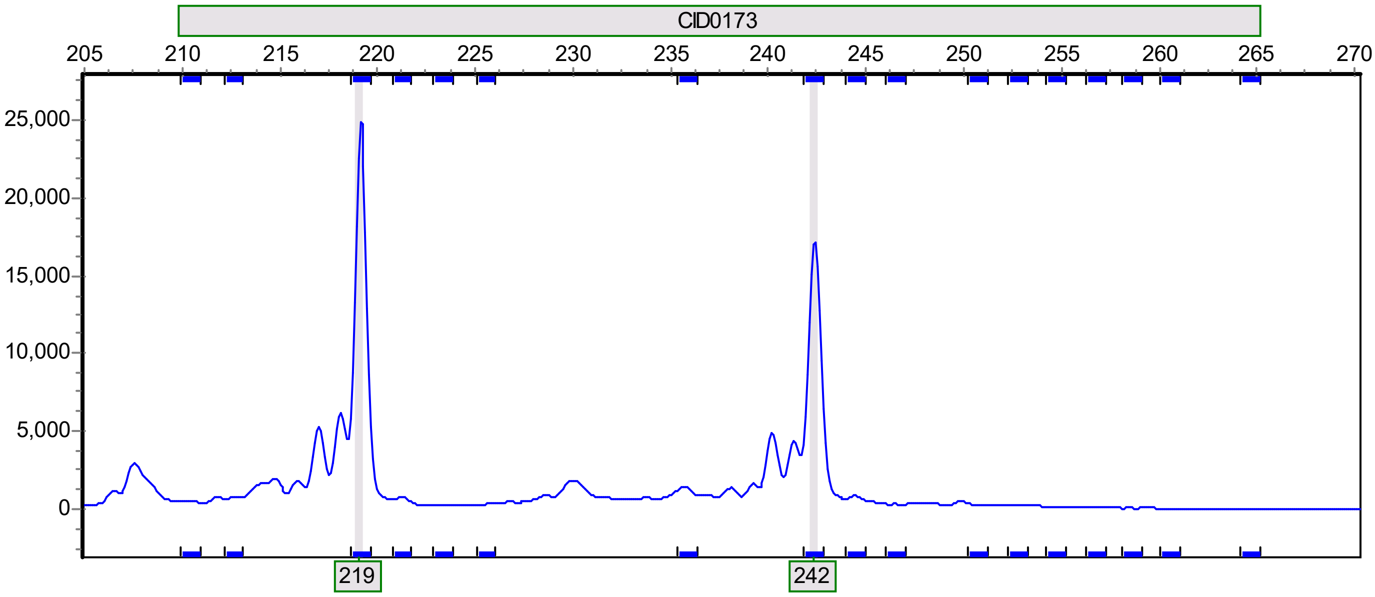

No	Size	Height	Area	Marker	Allele	Difference	Quality	Score	Allele Comments	Sample Comments
1	172.5	31795	234610	EST1573	173	0.00	Pass	500.0	[<Confirmed>]	
2	174.3	19233	127866	EST1573	174	0.10	Pass	500.0	[<Confirmed>]	
3	219.1	24916	176786	CID0173	219	0.10	Pass	500.0	[<Confirmed>]	
4	242.4	17185	130197	CID0173	242	0.00	Pass	500.0	[<Confirmed>]	

Sample 65: CID0173_EST1573_CY3_A11.fsa Run date and time: 09/21/2024 - 03:00:19 -> 09/21/2024 - 03:27:31

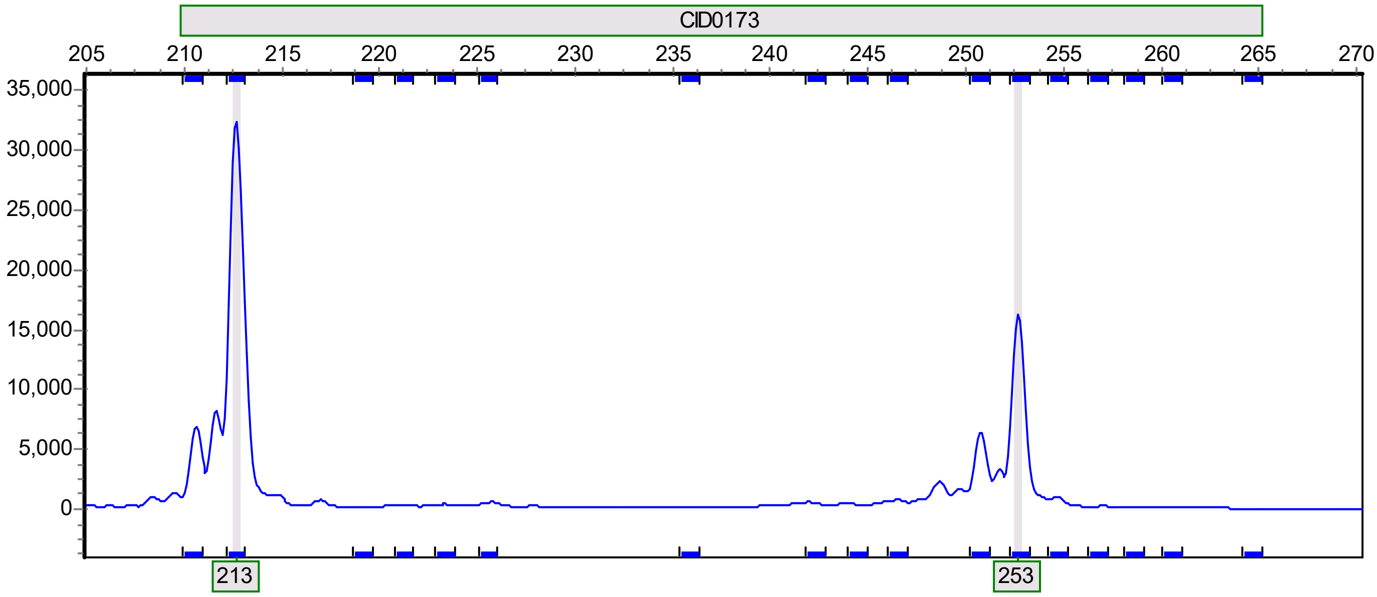

No	Size	Height	Area	Marker	Allele	Difference	Quality	Score	Allele Comments	Sample Comments
1	170.4	31057	323824	EST1573	171	0.20	Pass	500.0	[<SAT (Repaired)><Confirmed>]	
2	212.7	32379	274478	CID0173	213	0.00	Pass	500.0	[<SAT (Repaired)><Confirmed>]	
3	252.7	16317	122530	CID0173	253	0.10	Pass	500.0	[<Confirmed>]	

Sample 66: CID0173_EST1573_CY4_C11.fsa Run date and time: 09/21/2024 - 03:00:19 -> 09/21/2024 - 03:27:31

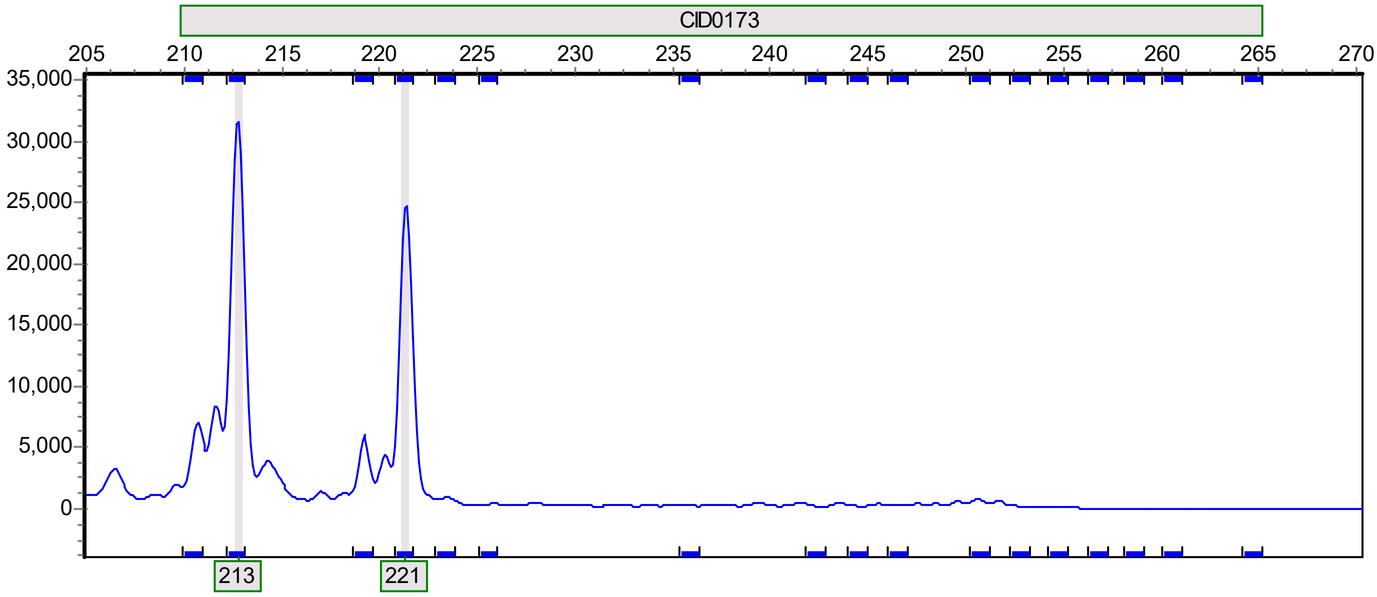

No	Size	Height	Area	Marker	Allele	Difference	Quality	Score	Allele Comments	Sample Comments
1	174.4	31865	235640	EST1573	174	0.00	Pass	500.0	[<Confirmed>]	
2	212.8	31606	248161	CID0173	213	0.10	Pass	500.0	[<SAT (Repaired)><Confirmed>]	
3	221.4	24694	183041	CID0173	221	0.10	Pass	500.0	[<Confirmed>]	

Sample 67: CID0173_EST1573_CY5_E11.fsa Run date and time: 09/21/2024 - 03:00:19 -> 09/21/2024 - 03:27:31

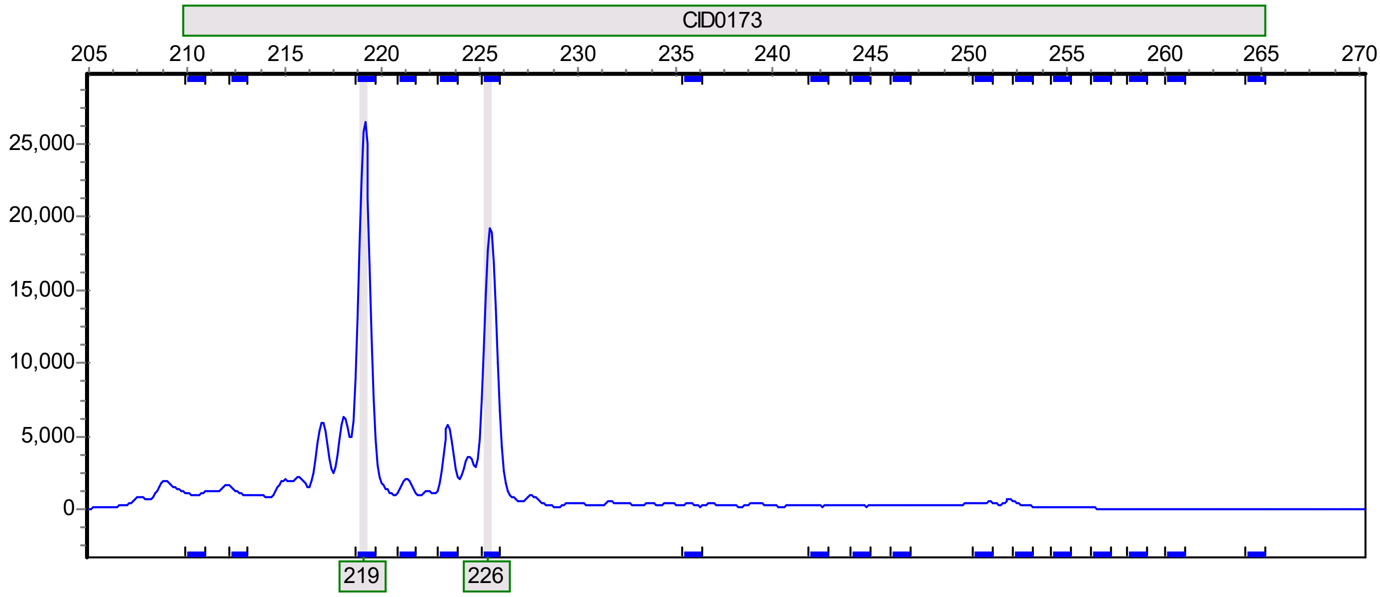

No	Size	Height	Area	Marker	Allele	Difference	Quality	Score	Allele Comments	Sample Comments
1	162.9	31823	216087	EST1573	163	0.00	Pass	500.0	[<Confirmed>]	
2	166.6	26405	166580	EST1573	167	0.10	Pass	500.0	[<Confirmed>]	
3	219.1	26520	205755	CID0173	219	0.10	Pass	500.0	[<Confirmed>]	
4	225.5	19260	144669	CID0173	226	0.10	Pass	500.0	[<Confirmed>]	

Sample 68: CID0173_EST1573_CY6_G11.fsa Run date and time: 09/21/2024 - 03:00:19 -> 09/21/2024 - 03:27:31

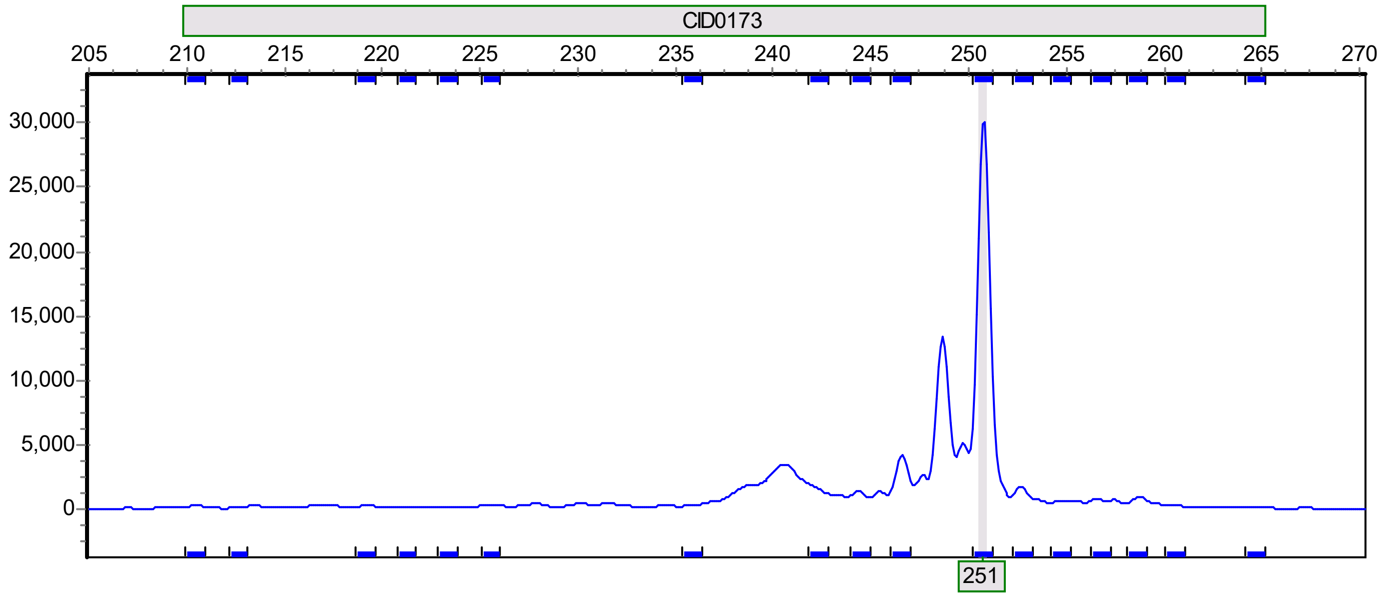

No	Size	Height	Area	Marker	Allele	Difference	Quality	Score	Allele Comments	Sample Comments
1	172.5	32083	242919	EST1573	173	0.00	Pass	500.0	[<Confirmed>]	
2	182.0	15306	104862	EST1573	182	0.00	Pass	500.0	[<Confirmed>]	
3	250.8	30060	210054	CID0173	251	0.00	Pass	500.0	[<Confirmed>]	

Allele Report

Sample 69: CID0173_EST1573_CY7_I11.fsa Run date and time: 09/21/2024 - 03:00:19 -> 09/21/2024 - 03:27:31

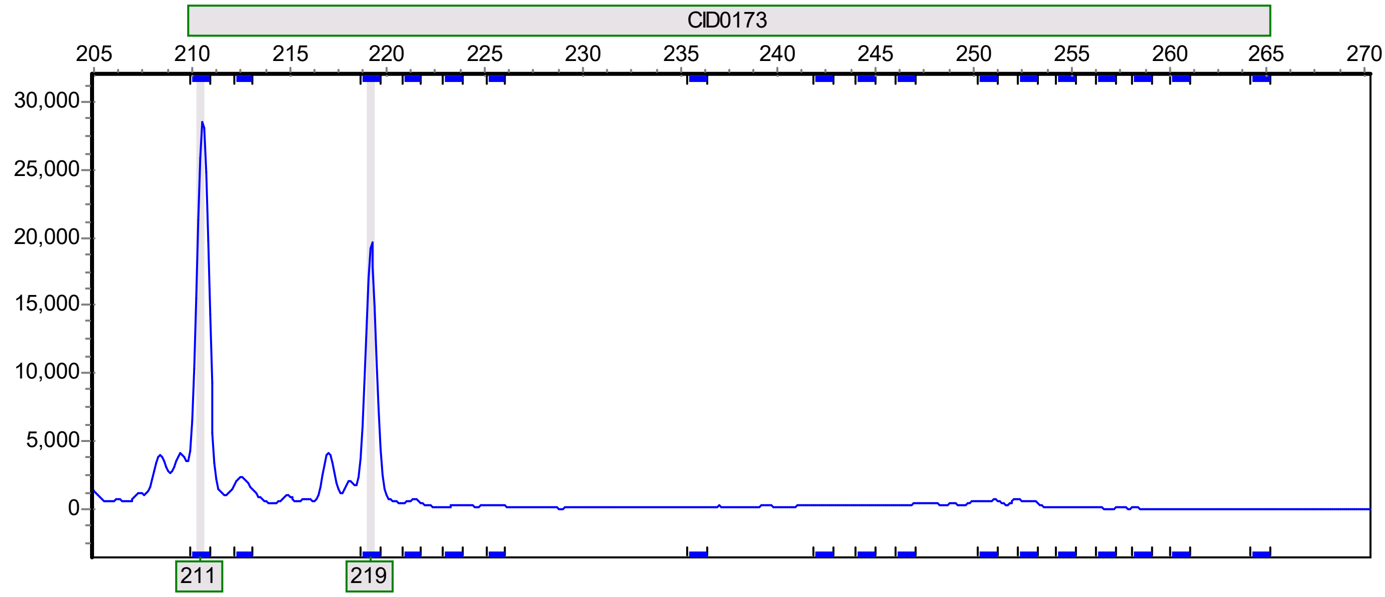

No	Size	Height	Area	Marker	Allele	Difference	Quality	Score	Allele Comments	Sample Comments
1	174.4	17388	120755	EST1573	174	0.00	Pass	500.0	[<Confirmed>]	
2	182.0	9276	61361	EST1573	182	0.00	Pass	500.0	[<Confirmed>]	
3	210.5	28501	205816	CID0173	211	0.00	Pass	500.0	[<Confirmed>]	
4	219.2	19619	140482	CID0173	219	0.00	Pass	500.0	[<Confirmed>]	

Sample 70: CID0173_EST1573_CY8_K11.fsa Run date and time: 09/21/2024 - 03:00:19 -> 09/21/2024 - 03:27:31

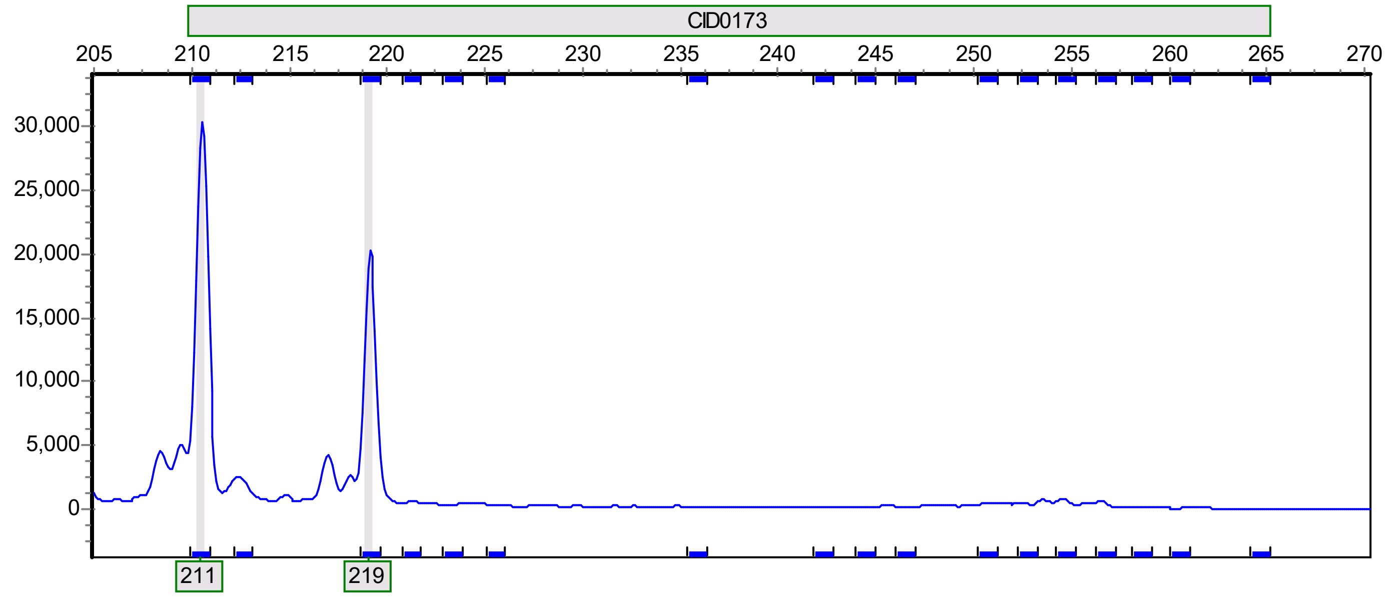

No	Size	Height	Area	Marker	Allele	Difference	Quality	Score	Allele Comments	Sample Comments
1	172.6	27764	186639	EST1573	173	0.10	Pass	500.0	[<Confirmed>]	
2	178.3	11462	77693	EST1573	178	0.10	Pass	500.0	[<Confirmed>]	
3	210.5	30356	221867	CID0173	211	0.00	Pass	500.0	[<Confirmed>]	
4	219.1	20285	148165	CID0173	219	0.10	Pass	500.0	[<Confirmed>]	

Sample 71: CID0173_EST1573_CY9_M11.fsa Run date and time: 09/21/2024 - 03:00:19 -> 09/21/2024 - 03:27:31

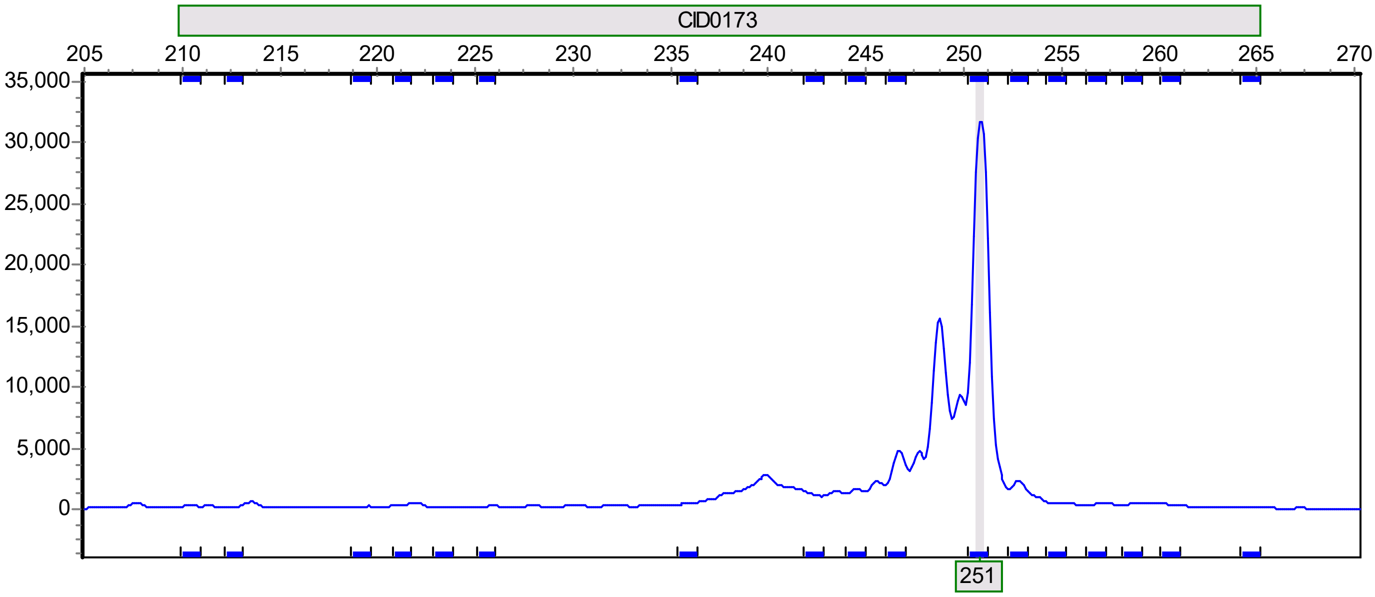

No	Size	Height	Area	Marker	Allele	Difference	Quality	Score	Allele Comments	Sample Comments
1	174.4	31757	234498	EST1573	174	0.00	Pass	500.0	[<Confirmed>]	
2	178.2	25700	182443	EST1573	178	0.00	Pass	500.0	[<Confirmed>]	
3	250.9	31725	286457	CID0173	251	0.10	Pass	500.0	[<Confirmed>]	

Sample 72: CID0173_EST1573_KC100_F19.fsa Run date and time: 09/21/2024 - 03:27:32 -> 09/21/2024 - 03:55:03

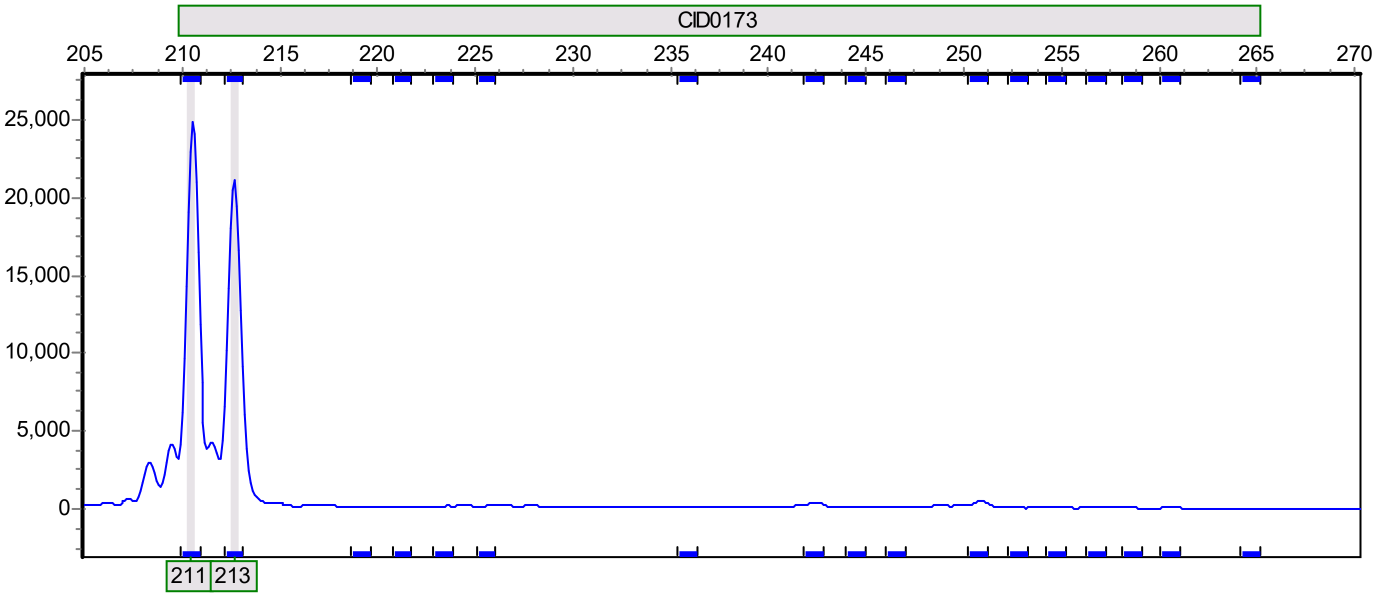

No	Size	Height	Area	Marker	Allele	Difference	Quality	Score	Allele Comments	Sample Comments
1	172.6	23188	161564	EST1573	173	0.10	Pass	500.0	[<Confirmed>]	
2	174.5	15432	107799	EST1573	174	0.10	Pass	500.0	[<Confirmed>]	
3	210.5	24866	183564	CID0173	211	0.00	Pass	500.0	[<Confirmed>]	
4	212.7	21064	158375	CID0173	213	0.00	Pass	500.0	[<Confirmed>]	

Sample 73: CID0173_EST1573_KC101_H19.fsa Run date and time: 09/21/2024 - 03:27:32 -> 09/21/2024 - 03:55:03

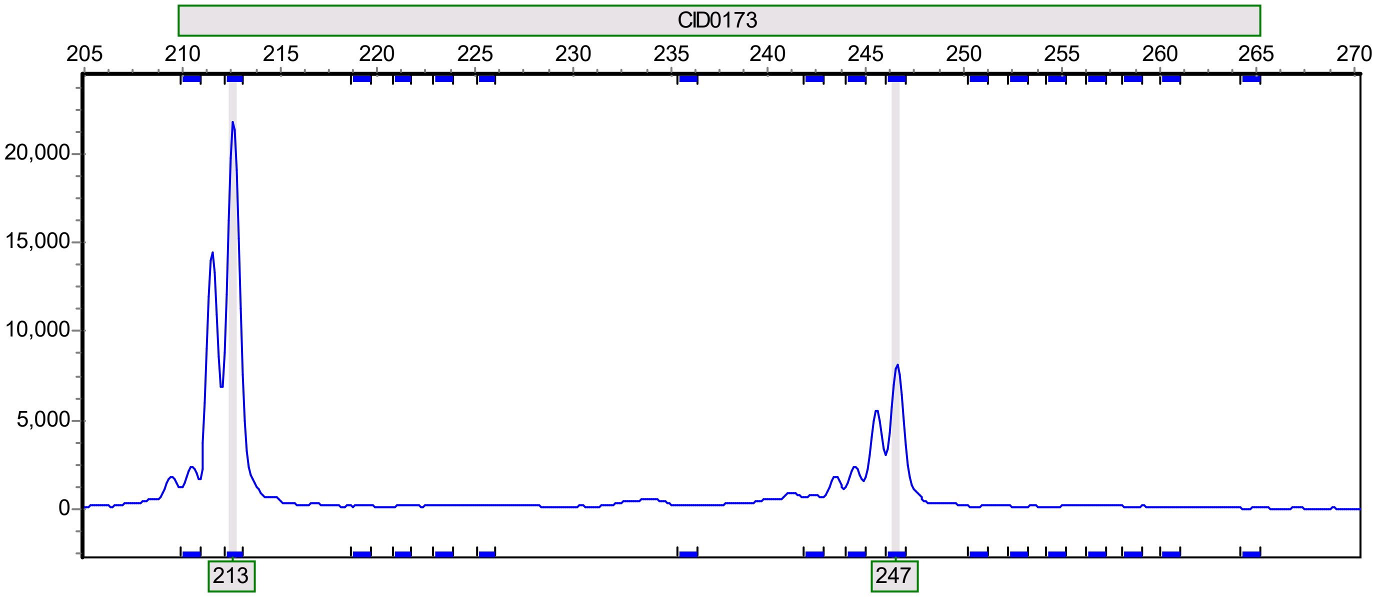

No	Size	Height	Area	Marker	Allele	Difference	Quality	Score	Allele Comments	Sample Comments
1	174.3	25039	161886	EST1573	174	0.10	Pass	500.0	[<Confirmed>]	
2	181.9	11532	76235	EST1573	182	0.10	Pass	500.0	[<Confirmed>]	
3	211.6	14476	98747	CID0173	212	0.00	Pass	500.0	[<Deleted>]	
4	212.6	21758	156685	CID0173	213	0.10	Pass	500.0	[<Confirmed>]	
5	246.6	8081	61059	CID0173	247	0.00	Pass	500.0	[<Confirmed>]	

Sample 74: CID0173_EST1573_KC102_J19.fsa Run date and time: 09/21/2024 - 03:27:32 -> 09/21/2024 - 03:55:03

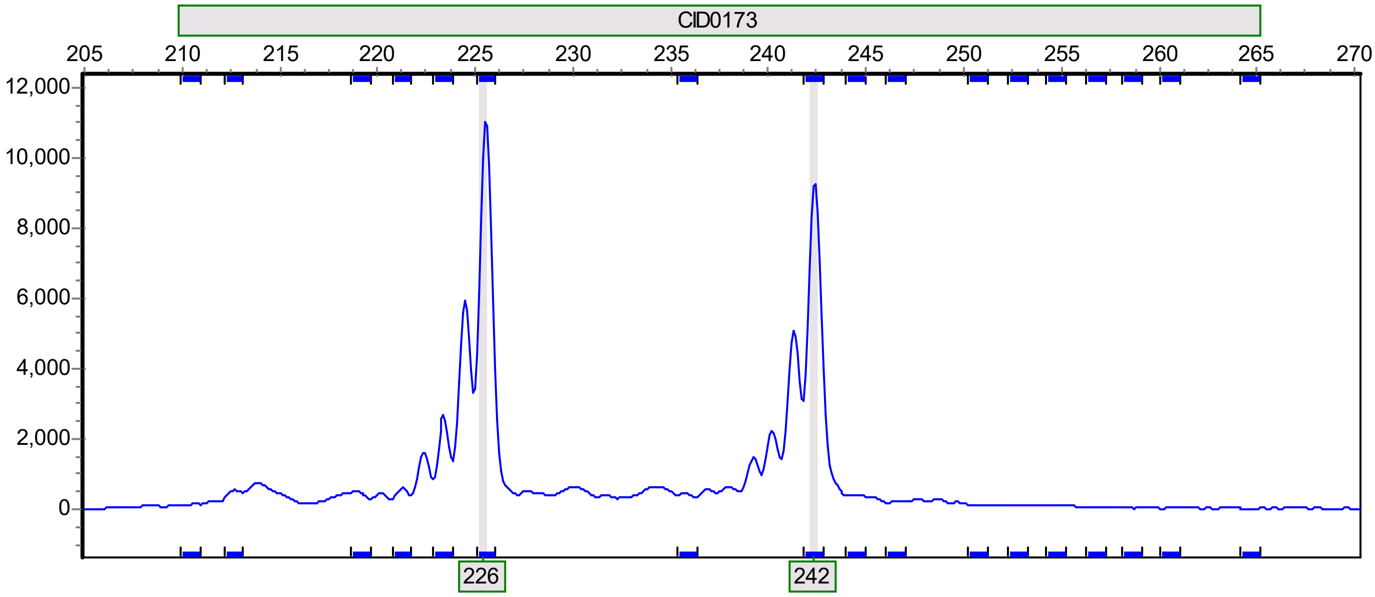

No	Size	Height	Area	Marker	Allele	Difference	Quality	Score	Allele Comments	Sample Comments
1	174.4	23031	148105	EST1573	174	0.00	Pass	500.0	[<Confirmed>]	
2	178.3	17699	118557	EST1573	178	0.10	Pass	500.0	[<Confirmed>]	
3	225.5	11051	80137	CID0173	226	0.10	Pass	500.0	[<Confirmed>]	
4	242.4	9256	71170	CID0173	242	0.00	Pass	500.0	[<Confirmed>]	

Sample 75: CID0173_EST1573_KC103_L19.fsa Run date and time: 09/21/2024 - 03:27:32 -> 09/21/2024 - 03:55:03

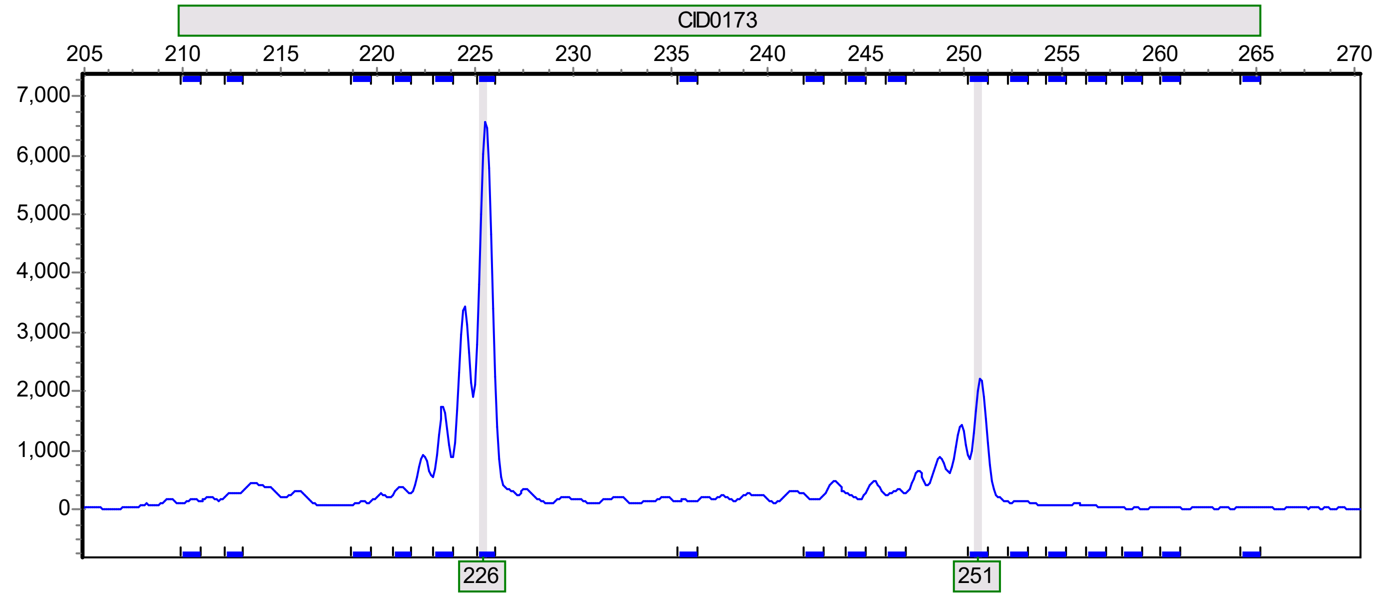

No	Size	Height	Area	Marker	Allele	Difference	Quality	Score	Allele Comments	Sample Comments
1	172.4	21788	141290	EST1573	173	0.10	Pass	500.0	[<Confirmed>]	
2	174.2	10963	72809	EST1573	174	0.20	Pass	500.0	[<Confirmed>]	
3	225.5	6561	47376	CID0173	226	0.10	Pass	500.0	[<Confirmed>]	
4	250.8	2192	15940	CID0173	251	0.00	Pass	316.3	[<Confirmed>]	

Sample 76: CID0173_EST1573_KC104_N19.fsa Run date and time: 09/21/2024 - 03:27:32 -> 09/21/2024 - 03:55:03

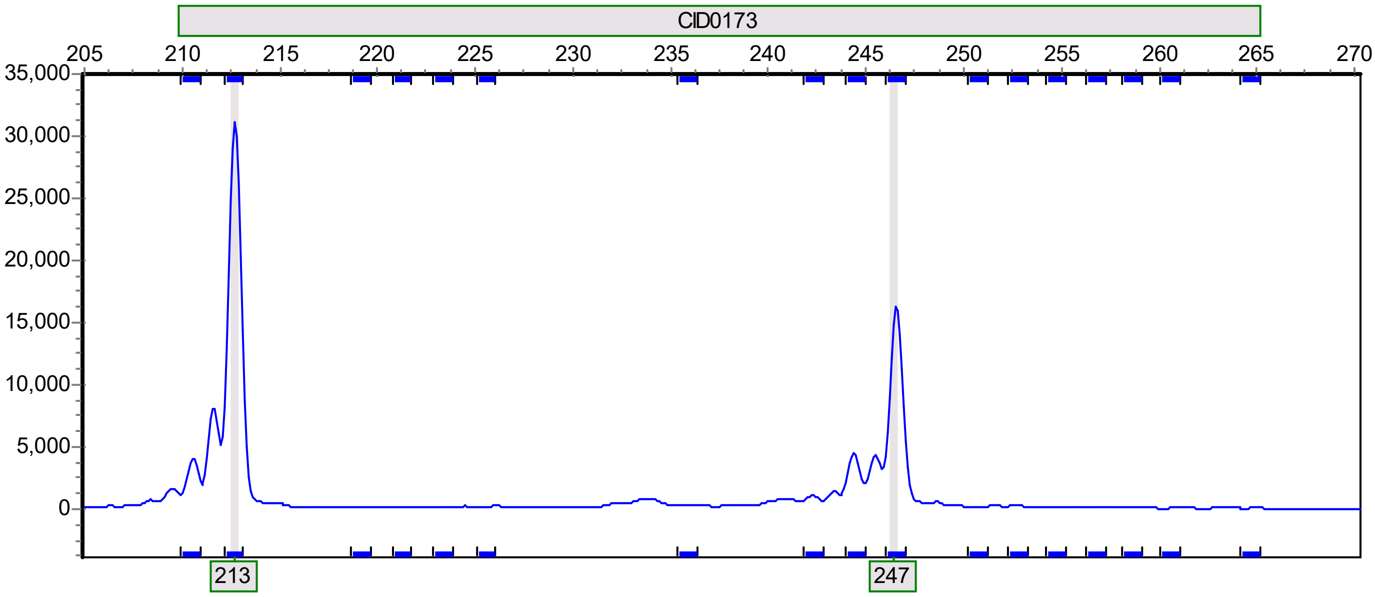

No	Size	Height	Area	Marker	Allele	Difference	Quality	Score	Allele Comments	Sample Comments
1	174.2	25692	169189	EST1573	174	0.20	Pass	500.0	[<Confirmed>]	
2	181.9	11296	74661	EST1573	182	0.10	Pass	500.0	[<Confirmed>]	
3	212.7	31131	226740	CID0173	213	0.00	Pass	500.0	[<Confirmed>]	
4	246.5	16206	118881	CID0173	247	0.10	Pass	500.0	[<Confirmed>]	

Allele Report

Sample 77: CID0173_EST1573_KC105_P19.fsa Run date and time: 09/21/2024 - 03:27:32 -> 09/21/2024 - 03:55:03

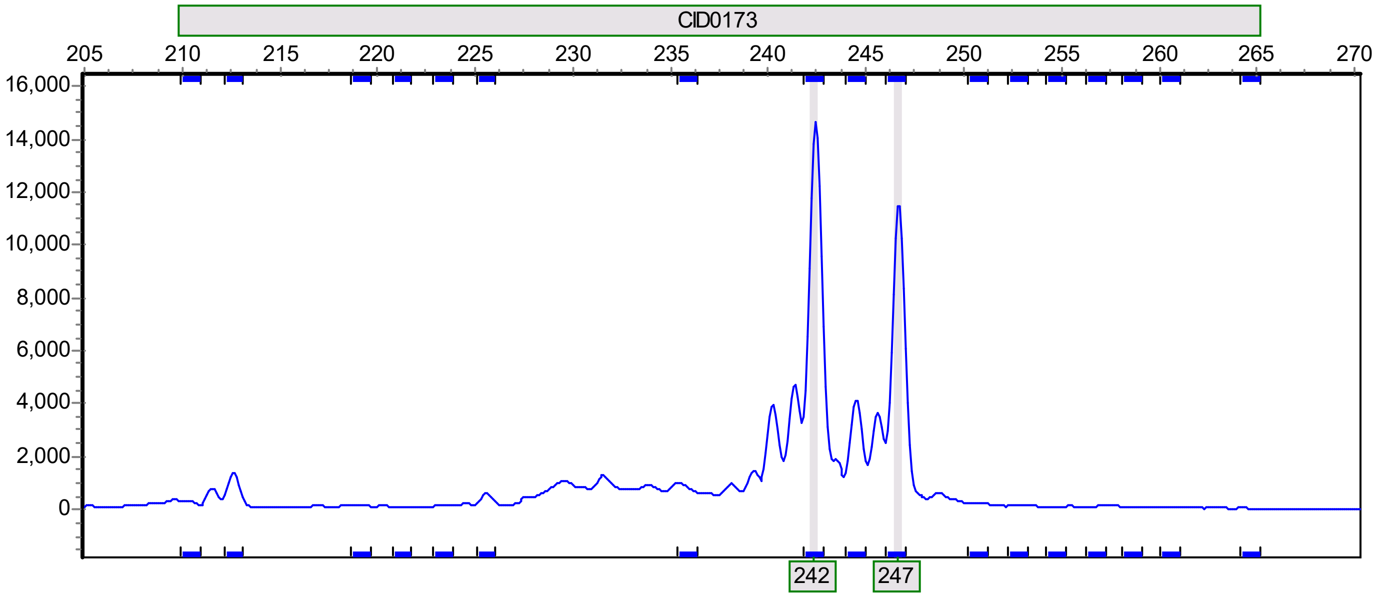

No	Size	Height	Area	Marker	Allele	Difference	Quality	Score	Allele Comments	Sample Comments
1	162.9	25171	194149	EST1573	163	0.00	Pass	500.0	[<SAT (Repaired)><Confirmed>]	
2	174.4	16463	106980	EST1573	174	0.00	Pass	500.0	[<Confirmed>]	
3	242.4	14653	109550	CID0173	242	0.00	Pass	500.0	[<Confirmed>]	
4	246.7	11491	84309	CID0173	247	0.10	Pass	500.0	[<Confirmed>]	

Sample 78: CID0173_EST1573_KC106_B21.fsa Run date and time: 09/21/2024 - 03:27:32 -> 09/21/2024 - 03:55:03

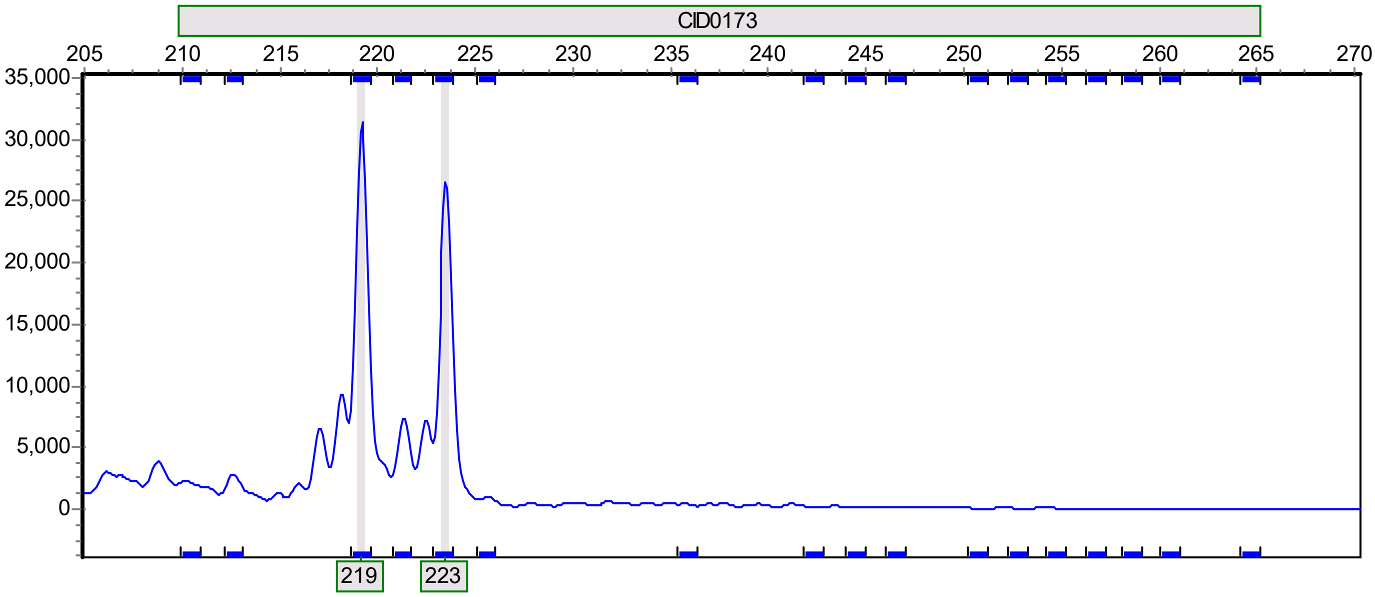

No	Size	Height	Area	Marker	Allele	Difference	Quality	Score	Allele Comments	Sample Comments
1	170.7	37813	279241	EST1573	171	0.10	Pass	500.0	[<SAT (Repaired)><Confirmed>]	
2	172.6	31386	243156	EST1573	173	0.10	Pass	500.0	[<Confirmed>]	
3	219.2	31429	262360	CID0173	219	0.00	Pass	500.0	[<Confirmed>]	
4	223.5	26588	203792	CID0173	223	0.10	Pass	500.0	[<Confirmed>]	

Sample 79: CID0173_EST1573_KC107_D21.fsa Run date and time: 09/21/2024 - 03:27:32 -> 09/21/2024 - 03:55:03

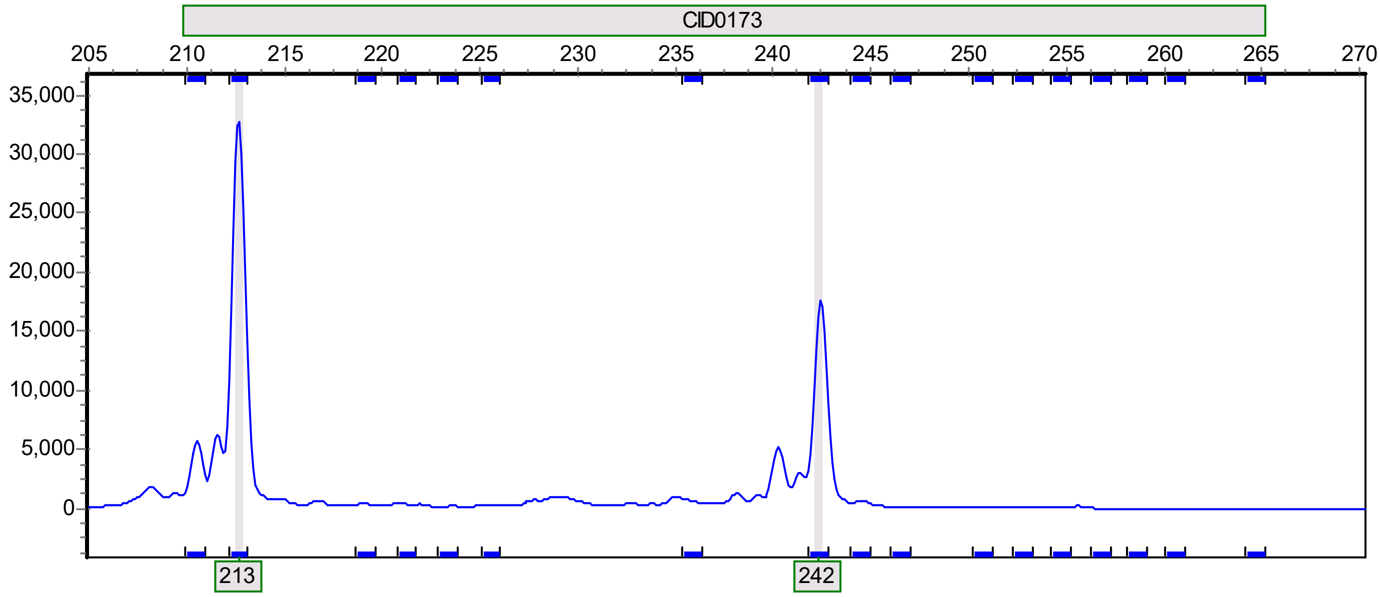

No	Size	Height	Area	Marker	Allele	Difference	Quality	Score	Allele Comments	Sample Comments
1	170.7	35449	248652	EST1573	171	0.10	Pass	500.0	[<SAT (Repaired)><Confirmed>]	
2	172.7	29379	198054	EST1573	173	0.20	Pass	500.0	[<Confirmed>]	
3	212.7	32710	249691	CID0173	213	0.00	Pass	500.0	[<SAT (Repaired)><Confirmed>]	
4	242.4	17594	131962	CID0173	242	0.00	Pass	500.0	[<Confirmed>]	

Sample 80: CID0173_EST1573_KC108_F21.fsa Run date and time: 09/21/2024 - 03:27:32 -> 09/21/2024 - 03:55:03

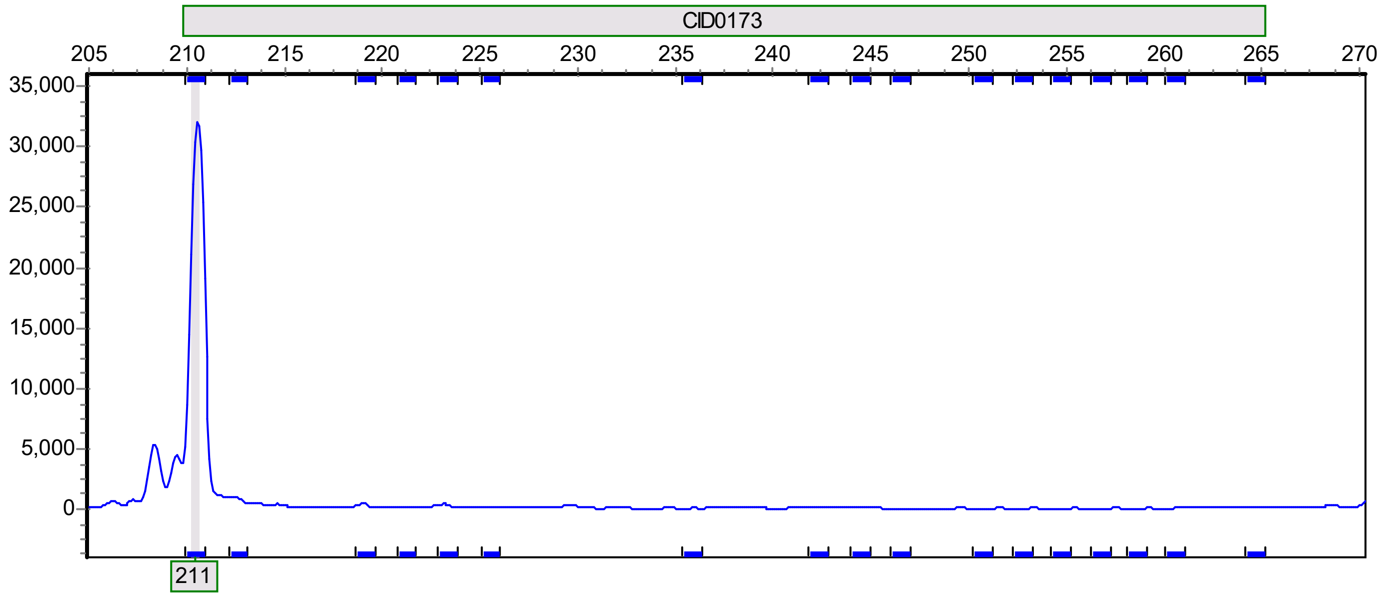

No	Size	Height	Area	Marker	Allele	Difference	Quality	Score	Allele Comments	Sample Comments
1	170.6	31705	218523	EST1573	171	0.00	Pass	500.0	[<Confirmed>]	
2	172.5	25893	172324	EST1573	173	0.00	Pass	500.0	[<Confirmed>]	
3	210.5	32061	258509	CID0173	211	0.00	Pass	500.0	[<Confirmed>]	

Sample 81: CID0173_EST1573_KC109_H21.fsa Run date and time: 09/21/2024 - 03:27:32 -> 09/21/2024 - 03:55:03

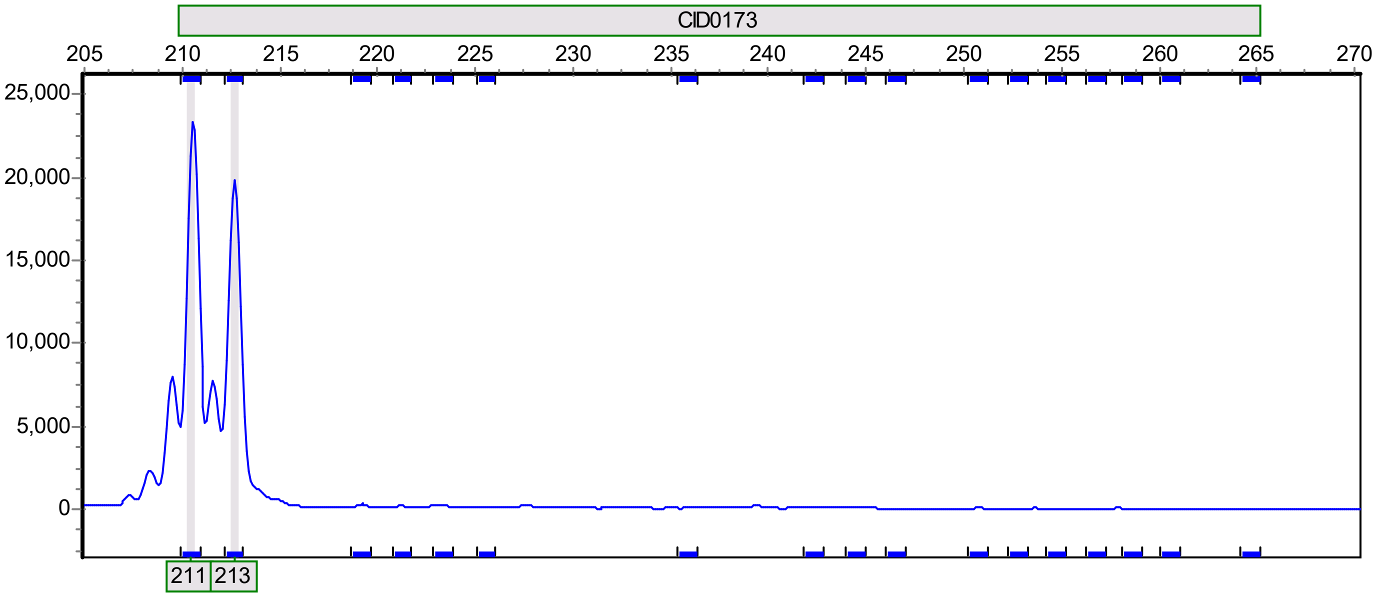

No	Size	Height	Area	Marker	Allele	Difference	Quality	Score	Allele Comments	Sample Comments
1	170.7	28194	183896	EST1573	171	0.10	Pass	500.0	[<Confirmed>]	
2	174.4	12590	86182	EST1573	174	0.00	Pass	500.0	[<Confirmed>]	
3	209.5	7940	51807	CID0173	210	0.00	Undetermined	500.0	[<Deleted>]	
4	210.5	23355	168790	CID0173	211	0.00	Pass	500.0	[<Confirmed>]	
5	211.6	7672	50423	CID0173	212	0.00	Undetermined	500.0	[<Deleted>]	
6	212.7	19841	144369	CID0173	213	0.00	Pass	500.0	[<Confirmed>]	

Sample 82: CID0173_EST1573_KC10_A21.fsa Run date and time: 09/21/2024 - 03:00:19 -> 09/21/2024 - 03:27:31

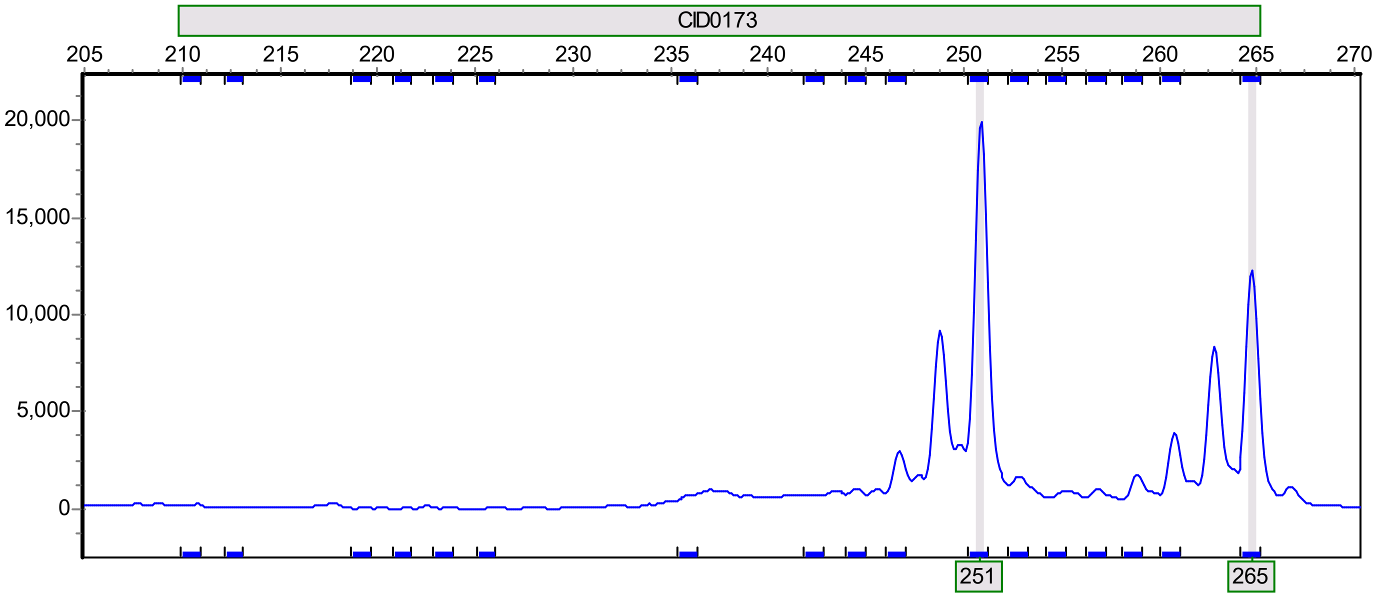

No	Size	Height	Area	Marker	Allele	Difference	Quality	Score	Allele Comments	Sample Comments
1	182.1	17993	130172	EST1573	182	0.10	Pass	500.0	[<Confirmed>]	
2	190.1	16689	114453	EST1573	190	0.10	Pass	500.0	[<Confirmed>]	
3	250.9	19944	151234	CID0173	251	0.10	Pass	500.0	[<Confirmed>]	
4	264.8	12251	93332	CID0173	265	0.10	Pass	500.0	[<Confirmed>]	

Sample 83: CID0173_EST1573_KC110_J21.fsa Run date and time: 09/21/2024 - 03:27:32 -> 09/21/2024 - 03:55:03

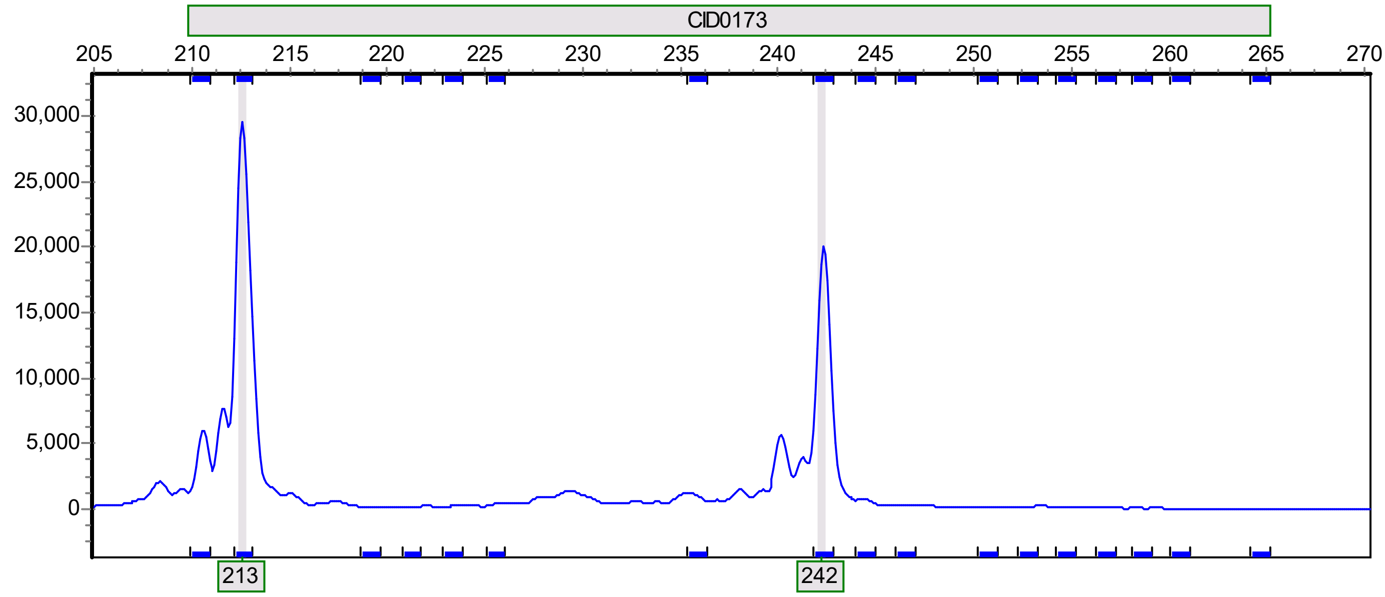

No	Size	Height	Area	Marker	Allele	Difference	Quality	Score	Allele Comments	Sample Comments
1	170.7	36550	268115	EST1573	171	0.10	Pass	500.0	[<SAT (Repaired)><Confirmed>]	
2	172.7	31389	218870	EST1573	173	0.20	Pass	500.0	[<Confirmed>]	
3	212.6	29519	252847	CID0173	213	0.10	Pass	500.0	[<SAT (Repaired)><Confirmed>]	
4	242.3	19984	155111	CID0173	242	0.10	Pass	500.0	[<Confirmed>]	

Sample 84: CID0173_EST1573_KC111_L21.fsa Run date and time: 09/21/2024 - 03:27:32 -> 09/21/2024 - 03:55:03

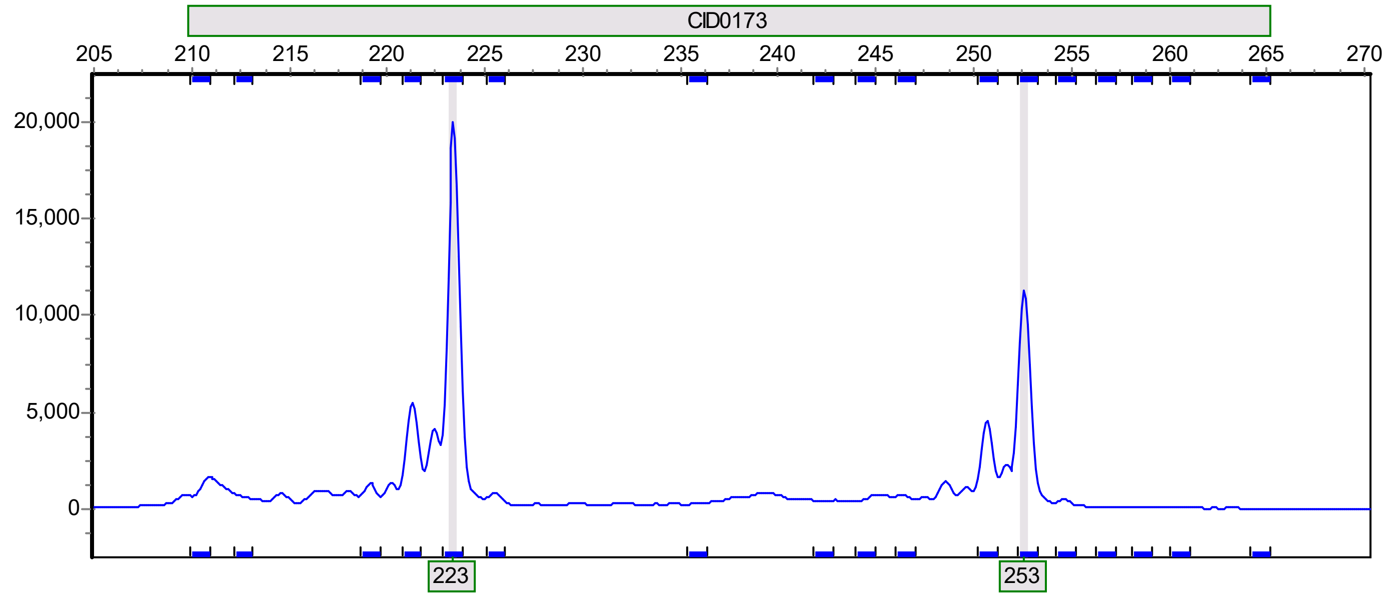

No	Size	Height	Area	Marker	Allele	Difference	Quality	Score	Allele Comments	Sample Comments
1	162.9	27333	178661	EST1573	163	0.00	Pass	500.0	[<Confirmed>]	
2	170.7	26482	177549	EST1573	171	0.10	Pass	500.0	[<Confirmed>]	
3	223.4	19978	147219	CID0173	223	0.00	Pass	500.0	[<Confirmed>]	
4	252.6	11245	81749	CID0173	253	0.20	Pass	500.0	[<Confirmed>]	

Allele Report

Sample 85: CID0173_EST1573_KC112_N21.fsa Run date and time: 09/21/2024 - 03:27:32 -> 09/21/2024 - 03:55:03

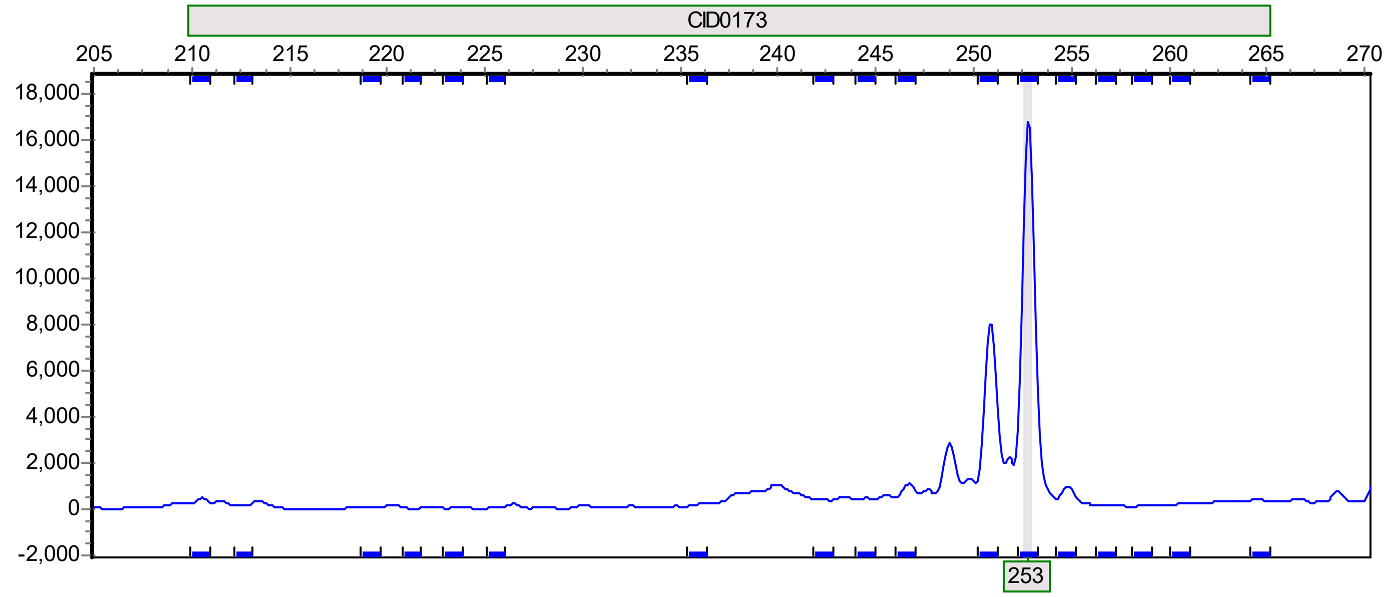

No	Size	Height	Area	Marker	Allele	Difference	Quality	Score	Allele Comments	Sample Comments
1	170.7	31454	230662	EST1573	171	0.10	Pass	500.0	[<Confirmed>]	
2	178.2	18655	123305	EST1573	178	0.00	Pass	500.0	[<Confirmed>]	
3	252.8	16800	117228	CID0173	253	0.00	Pass	500.0	[<Confirmed>]	

Sample 86: CID0173_EST1573_KC113_P21.fsa Run date and time: 09/21/2024 - 03:27:32 -> 09/21/2024 - 03:55:03

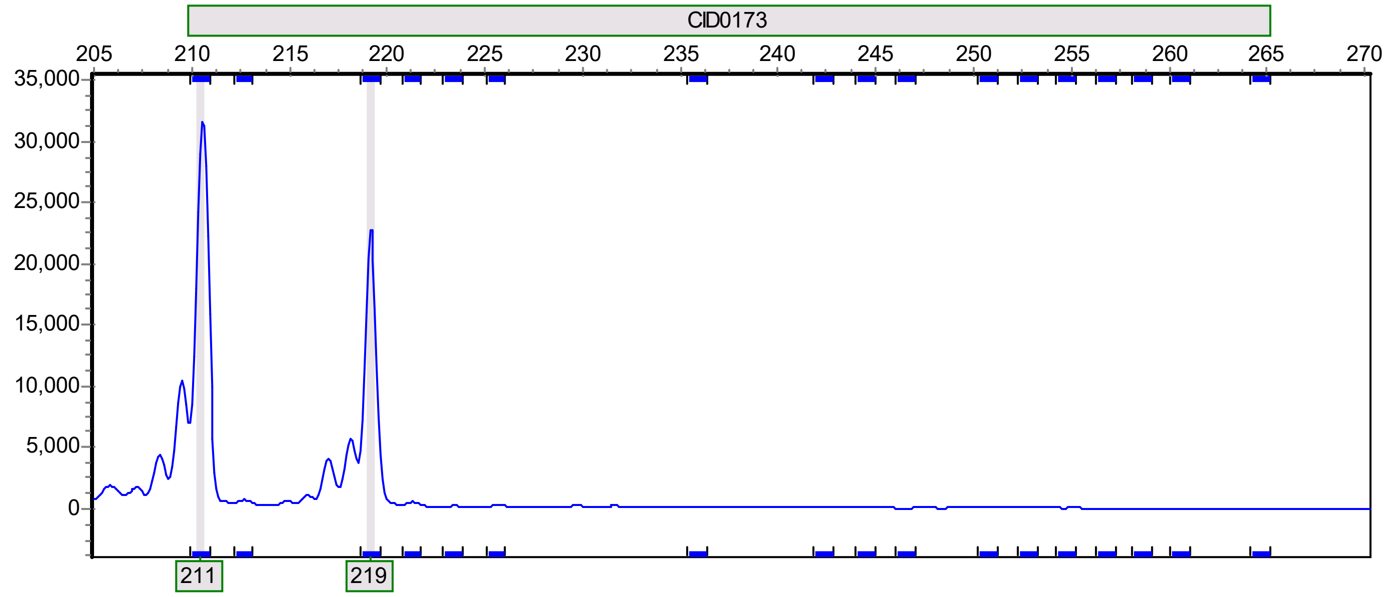

No	Size	Height	Area	Marker	Allele	Difference	Quality	Score	Allele Comments	Sample Comments
1	172.6	31822	225149	EST1573	173	0.10	Pass	500.0	[<Confirmed>]	
2	182.0	11650	76383	EST1573	182	0.00	Pass	500.0	[<Confirmed>]	
3	209.5	10424	69003	CID0173	210	0.00	Undetermined	500.0	[<Deleted>]	
4	210.5	31625	232748	CID0173	211	0.00	Pass	500.0	[<Confirmed>]	
5	219.2	22750	161798	CID0173	219	0.00	Pass	500.0	[<Confirmed>]	

Sample 87: CID0173_EST1573_KC114_B23.fsa Run date and time: 09/21/2024 - 03:27:32 -> 09/21/2024 - 03:55:03

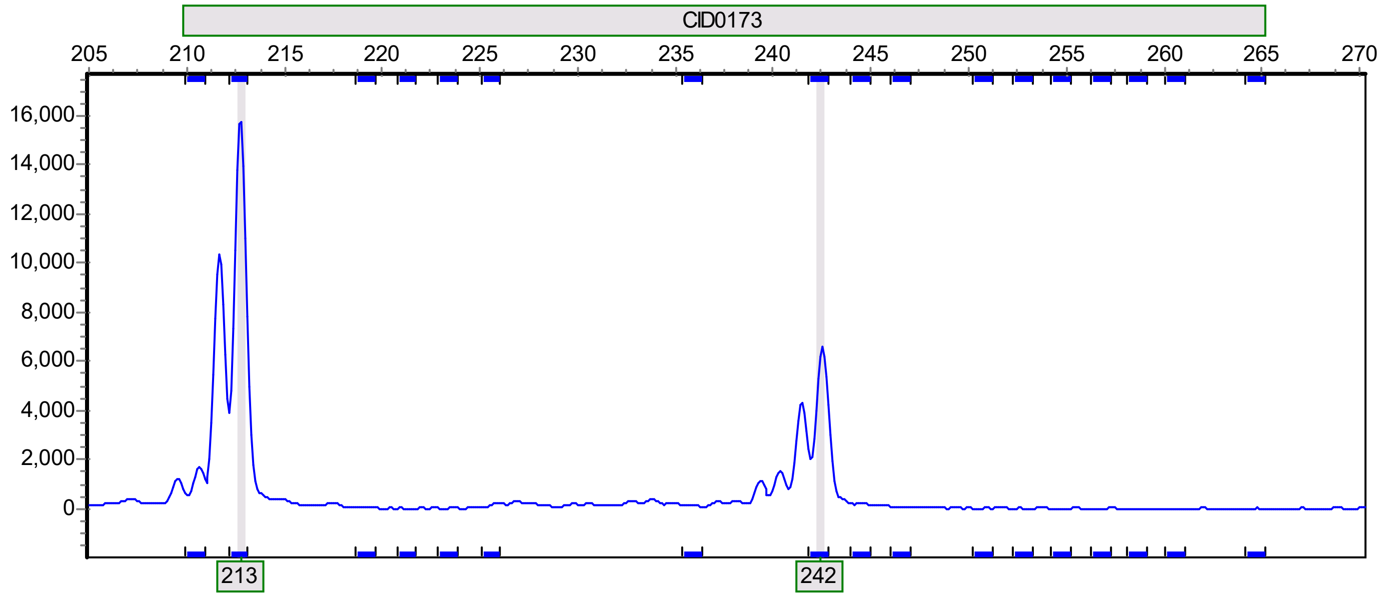

No	Size	Height	Area	Marker	Allele	Difference	Quality	Score	Allele Comments	Sample Comments
1	170.8	27074	166015	EST1573	171	0.20	Pass	500.0	[<Confirmed>]	
2	172.7	20249	122853	EST1573	173	0.20	Pass	500.0	[<Confirmed>]	
3	211.7	10341	66205	CID0173	212	0.10	Pass	500.0	[<Deleted>]	
4	212.8	15727	106567	CID0173	213	0.10	Pass	500.0	[<Confirmed>]	
5	242.5	6555	47789	CID0173	242	0.10	Pass	500.0	[<Confirmed>]	

Sample 88: CID0173_EST1573_KC115_D23.fsa Run date and time: 09/21/2024 - 03:27:32 -> 09/21/2024 - 03:55:03

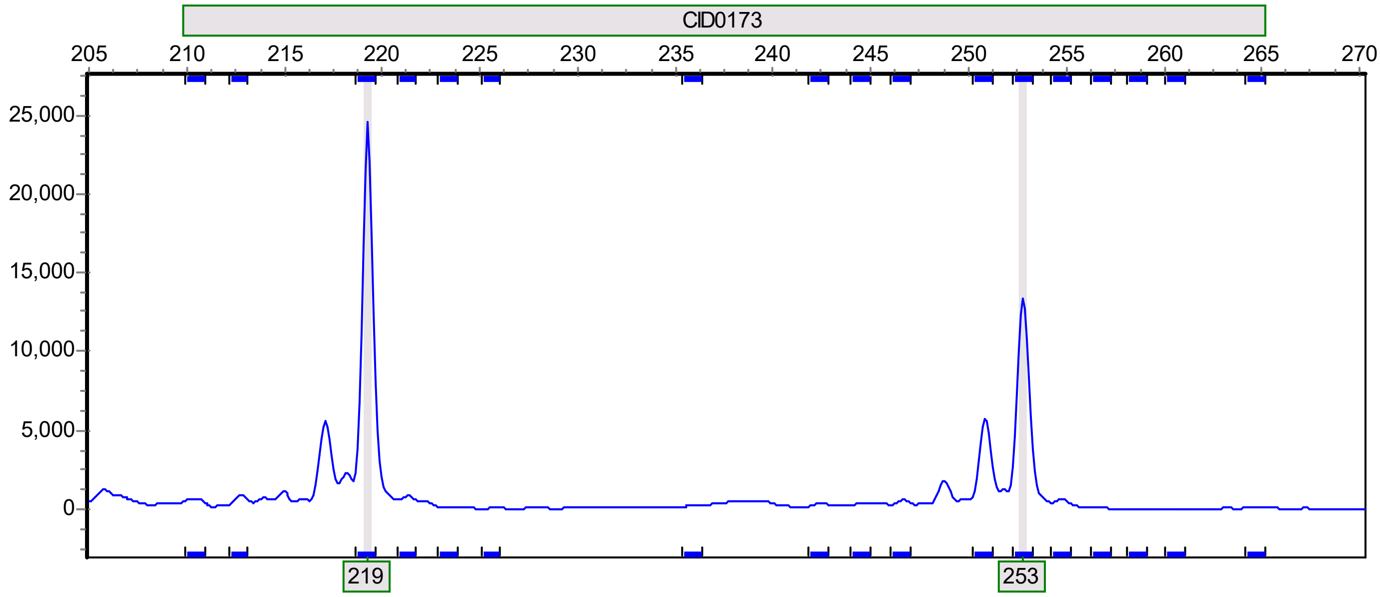

No	Size	Height	Area	Marker	Allele	Difference	Quality	Score	Allele Comments	Sample Comments
1	170.4	18018	119392	EST1573	171	0.20	Pass	500.0	[<Confirmed>]	
2	178.2	15228	99164	EST1573	178	0.00	Pass	500.0	[<Confirmed>]	
3	219.3	24601	164749	CID0173	219	0.10	Pass	500.0	[<Confirmed>]	
4	252.8	13321	91071	CID0173	253	0.00	Pass	500.0	[<Confirmed>]	

Allele Report

Sample 89: CID0173_EST1573_KC116_F23.fsa Run date and time: 09/21/2024 - 03:27:32 -> 09/21/2024 - 03:55:03

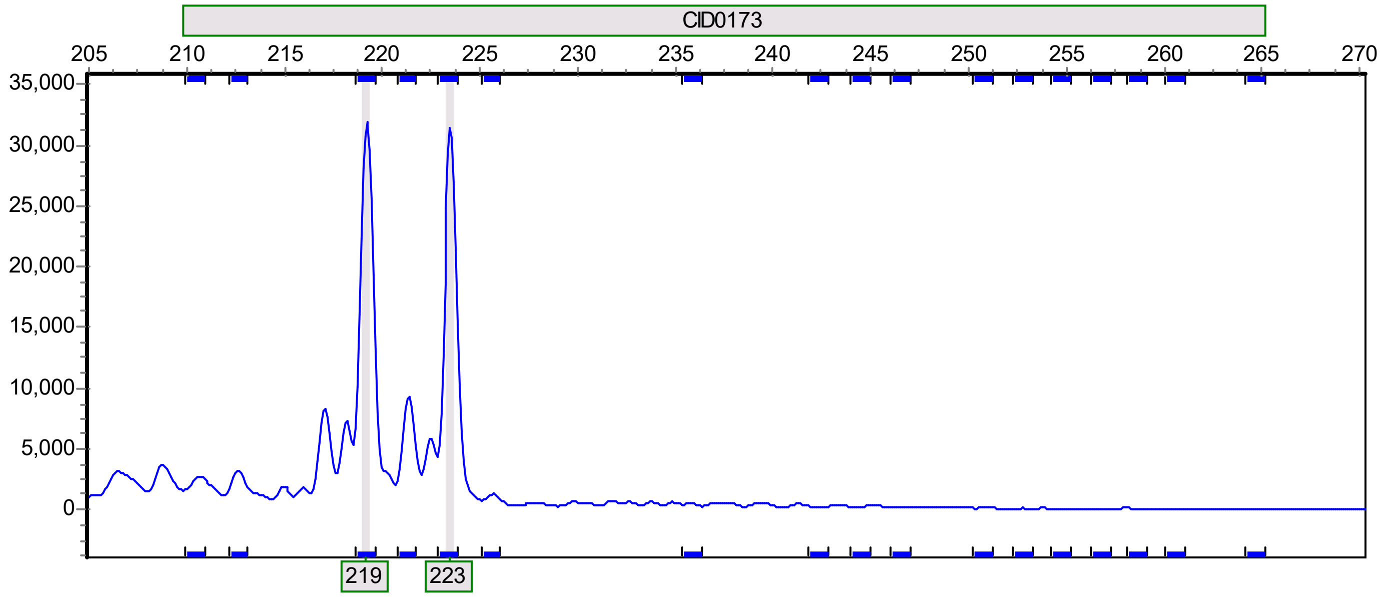

No	Size	Height	Area	Marker	Allele	Difference	Quality	Score	Allele Comments	Sample Comments
1	172.7	38452	268745	EST1573	173	0.20	Pass	500.0	[<SAT (Repaired)><Confirmed>]	
2	219.2	31925	265696	CID0173	219	0.00	Pass	500.0	[<Confirmed>]	
3	223.5	31424	232048	CID0173	223	0.10	Pass	500.0	[<Confirmed>]	

Sample 90: CID0173_EST1573_KC117_H23.fsa Run date and time: 09/21/2024 - 03:27:32 -> 09/21/2024 - 03:55:03

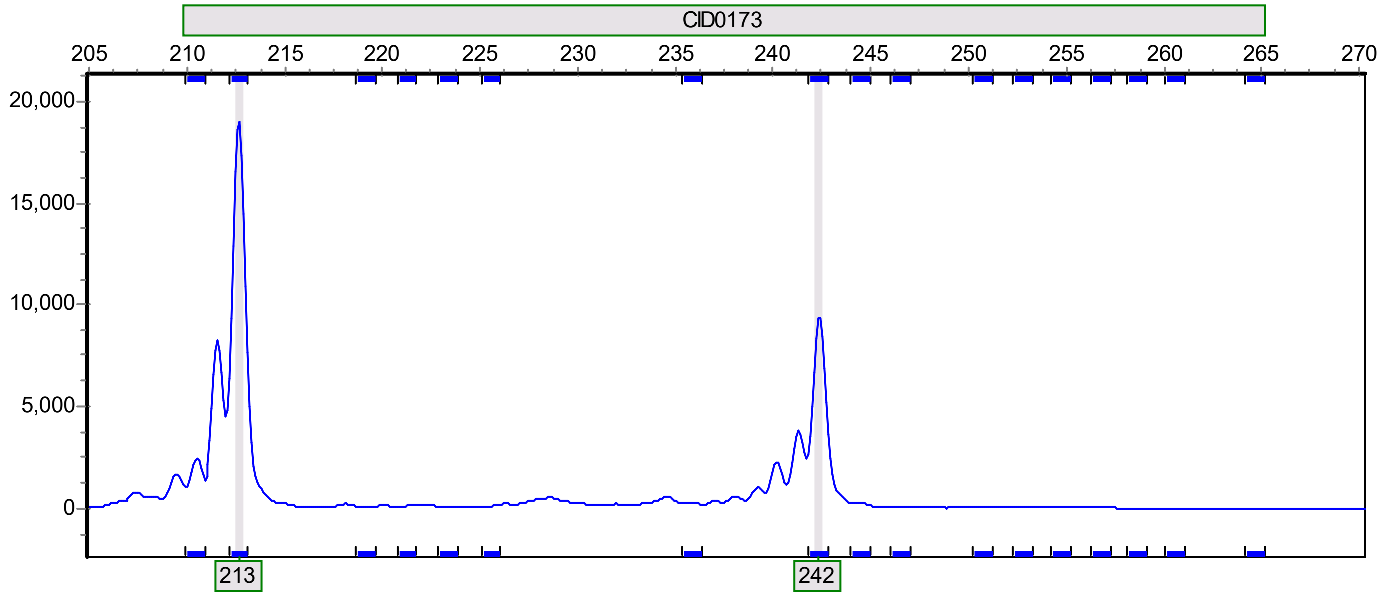

No	Size	Height	Area	Marker	Allele	Difference	Quality	Score	Allele Comments	Sample Comments
1	170.6	26175	167660	EST1573	171	0.00	Pass	500.0	[<Confirmed>]	
2	172.5	19796	130607	EST1573	173	0.00	Pass	500.0	[<Confirmed>]	
3	211.6	8293	58095	CID0173	212	0.00	Undetermined	500.0	[<Deleted>]	
4	212.7	19023	138460	CID0173	213	0.00	Pass	500.0	[<Confirmed>]	
5	242.4	9324	71762	CID0173	242	0.00	Pass	500.0	[<Confirmed>]	

Sample 91: CID0173_EST1573_KC118_J23.fsa Run date and time: 09/21/2024 - 03:27:32 -> 09/21/2024 - 03:55:03

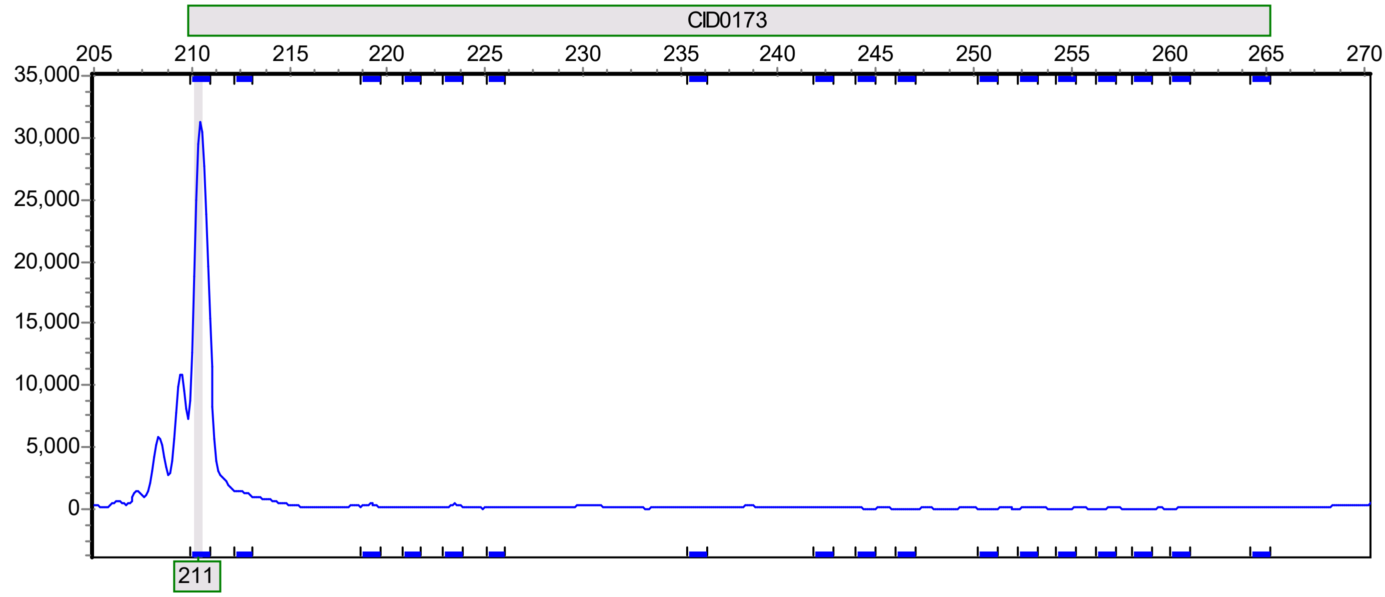

No	Size	Height	Area	Marker	Allele	Difference	Quality	Score	Allele Comments	Sample Comments
1	170.7	38905	269656	EST1573	171	0.10	Pass	500.0	[<SAT (Repaired)><Confirmed>]	
2	172.6	31805	224045	EST1573	173	0.10	Pass	500.0	[<Confirmed>]	
3	209.4	10844	73795	CID0173	210	0.10	Pass	500.0	[<Deleted>]	
4	210.4	31296	263522	CID0173	211	0.10	Pass	500.0	[<SAT (Repaired)><Confirmed>]	

Sample 92: CID0173_EST1573_KC119_L23.fsa Run date and time: 09/21/2024 - 03:27:32 -> 09/21/2024 - 03:55:03

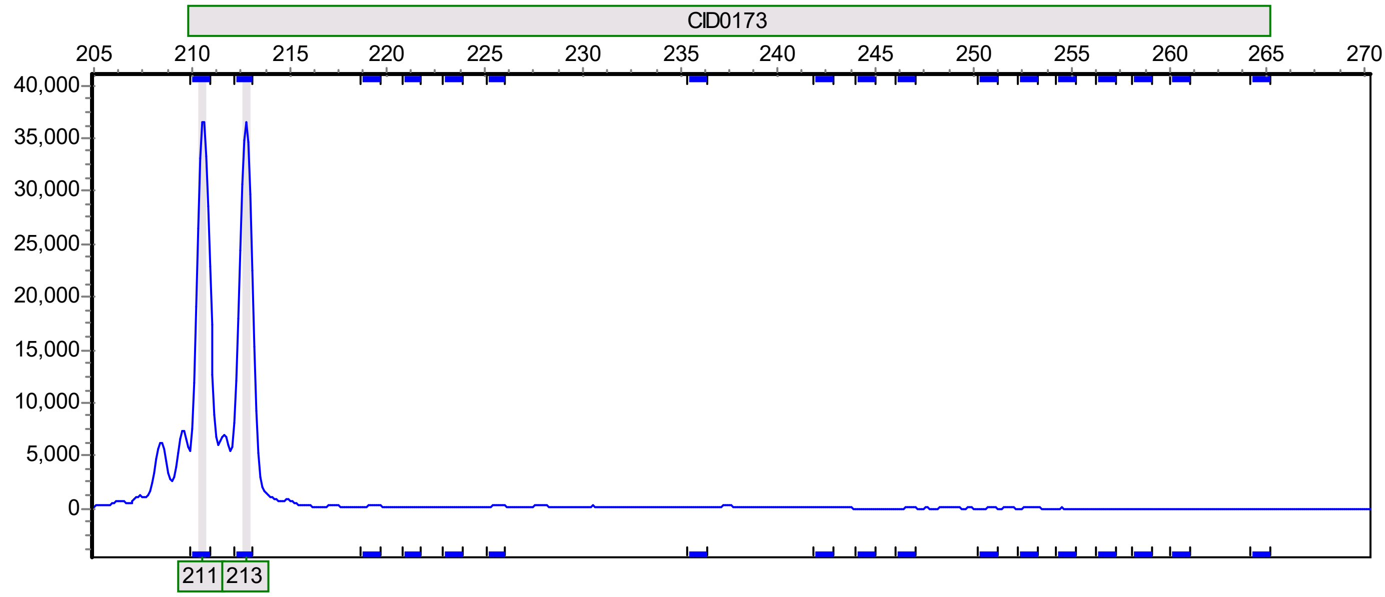

No	Size	Height	Area	Marker	Allele	Difference	Quality	Score	Allele Comments	Sample Comments
1	170.7	36189	255989	EST1573	171	0.10	Pass	500.0	[<SAT (Repaired)><Confirmed>]	
2	174.5	18663	123635	EST1573	174	0.10	Pass	500.0	[<Confirmed>]	
3	210.6	36533	282259	CID0173	211	0.10	Pass	500.0	[<SAT (Repaired)><Confirmed>]	
4	212.8	36531	273595	CID0173	213	0.10	Pass	500.0	[<SAT (Repaired)><Confirmed>]	

Allele Report

Sample 93: CID0173_EST1573_KC11_C21.fsa Run date and time: 09/21/2024 - 03:00:19 -> 09/21/2024 - 03:27:31

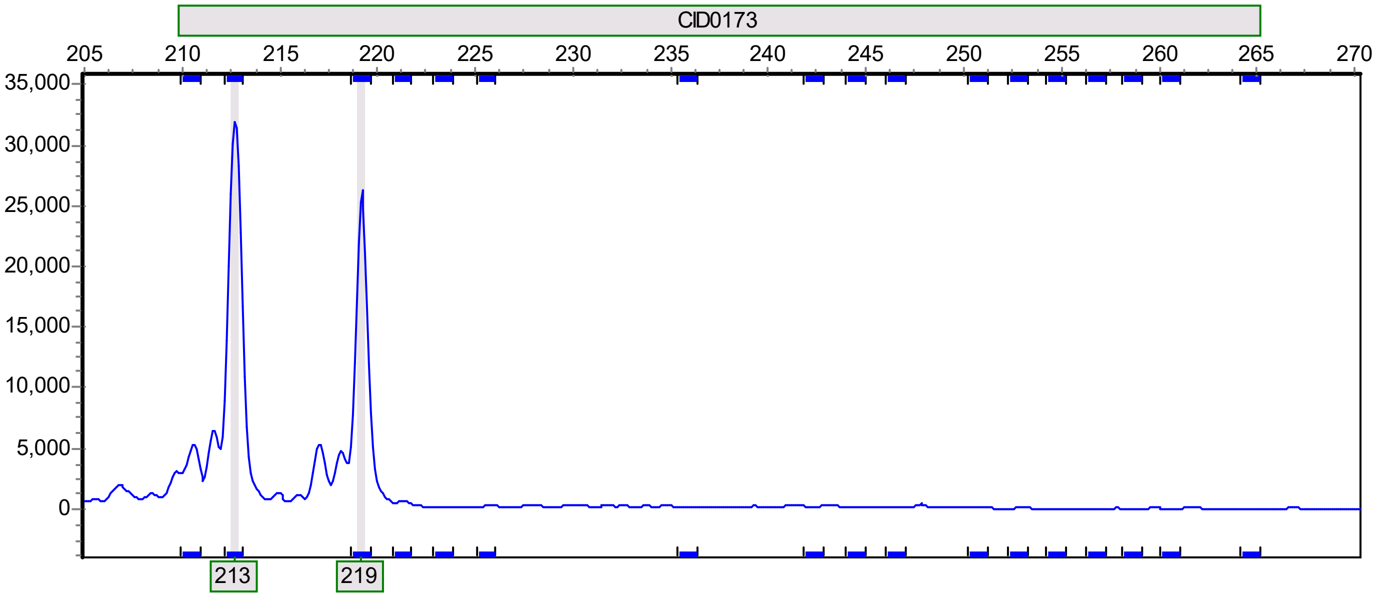

No	Size	Height	Area	Marker	Allele	Difference	Quality	Score	Allele Comments	Sample Comments
1	163.0	31902	233422	EST1573	163	0.10	Pass	500.0	[<Confirmed>]	
2	174.5	18068	116450	EST1573	174	0.10	Pass	500.0	[<Confirmed>]	
3	212.7	31921	247203	CID0173	213	0.00	Pass	500.0	[<Confirmed>]	
4	219.2	26316	197260	CID0173	219	0.00	Pass	500.0	[<Confirmed>]	

Sample 94: CID0173_EST1573_KC120_N23.fsa Run date and time: 09/21/2024 - 03:27:32 -> 09/21/2024 - 03:55:03

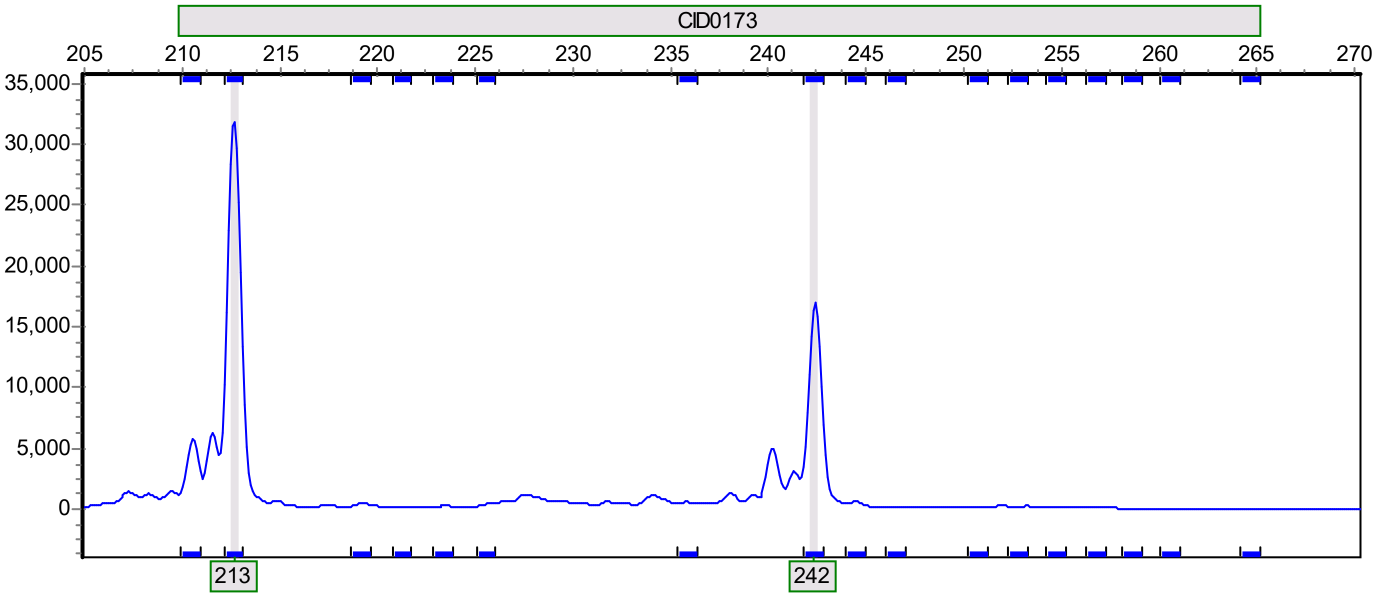

No	Size	Height	Area	Marker	Allele	Difference	Quality	Score	Allele Comments	Sample Comments
1	170.6	31281	218309	EST1573	171	0.00	Pass	500.0	[<SAT (Repaired)><Confirmed>]	
2	172.6	27774	179959	EST1573	173	0.10	Pass	500.0	[<Confirmed>]	
3	212.7	31787	239884	CID0173	213	0.00	Pass	500.0	[<Confirmed>]	
4	242.4	16925	121238	CID0173	242	0.00	Pass	500.0	[<Confirmed>]	

Allele Report

Sample 95: CID0173_EST1573_KC121_P23.fsa Run date and time: 09/21/2024 - 03:27:32 -> 09/21/2024 - 03:55:03

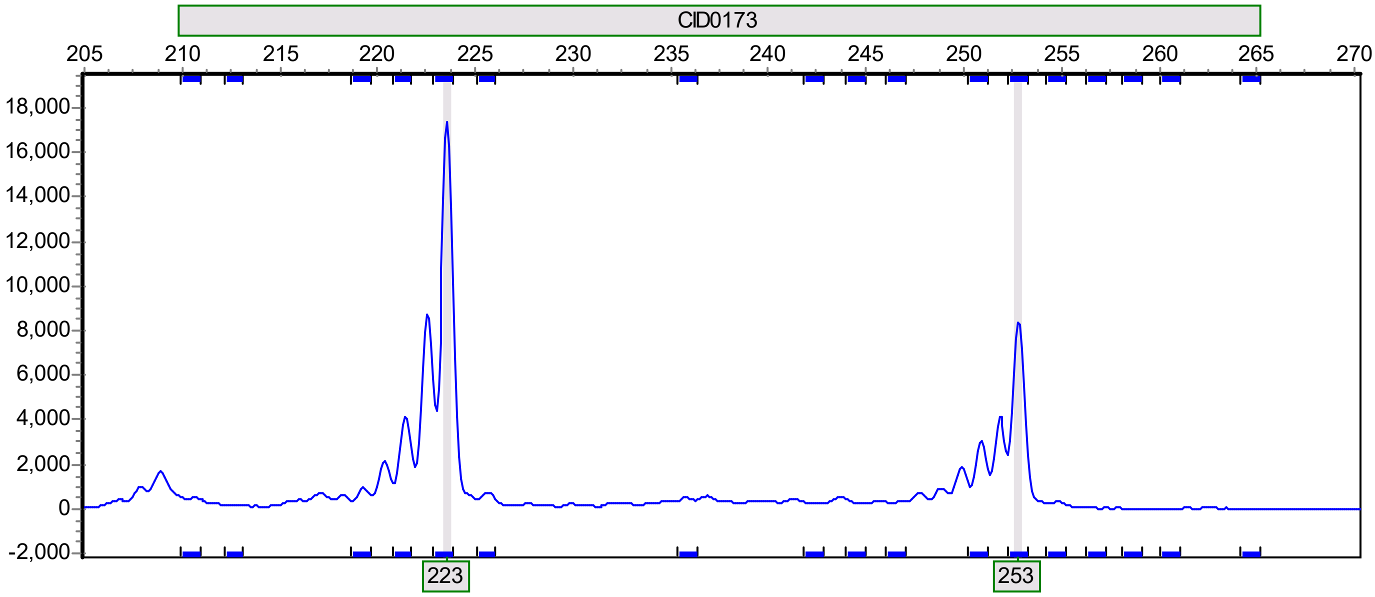

No	Size	Height	Area	Marker	Allele	Difference	Quality	Score	Allele Comments	Sample Comments
1	162.8	31667	218068	EST1573	163	0.10	Pass	500.0	[<Confirmed>]	
2	170.6	31529	206028	EST1573	171	0.00	Pass	500.0	[<Confirmed>]	
3	222.5	8705	56495	CID0173	222	0.10	Pass	500.0	[<Deleted>]	
4	223.6	17365	121540	CID0173	223	0.20	Pass	500.0	[<Confirmed>]	
5	252.8	8401	56835	CID0173	253	0.00	Pass	500.0	[<Confirmed>]	

Sample 96: CID0173_EST1573_KC122_A18.fsa Run date and time: 09/21/2024 - 03:55:04 -> 09/21/2024 - 04:22:25

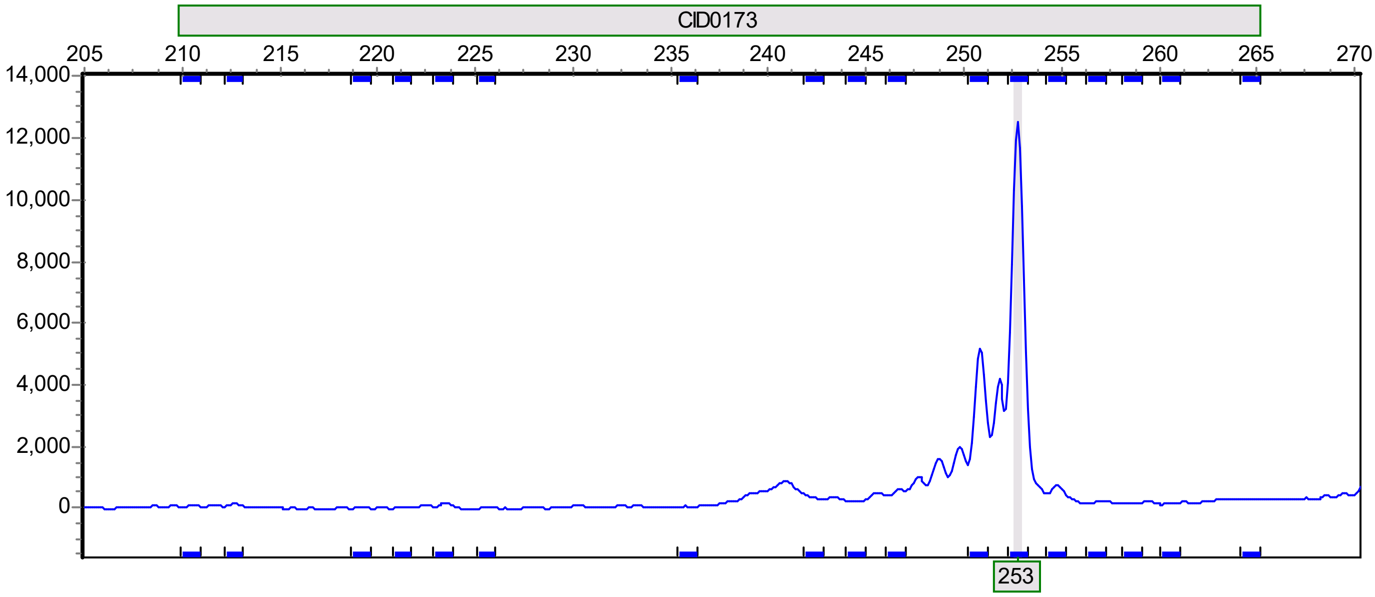

No	Size	Height	Area	Marker	Allele	Difference	Quality	Score	Allele Comments	Sample Comments
1	170.7	31742	233917	EST1573	171	0.10	Pass	500.0	[<Confirmed>]	
2	178.2	17492	117143	EST1573	178	0.00	Pass	500.0	[<Confirmed>]	
3	252.8	12533	89374	CID0173	253	0.00	Pass	500.0	[<Confirmed>]	

Allele Report

Sample 97: CID0173_EST1573_KC123_C18.fsa Run date and time: 09/21/2024 - 03:55:04 -> 09/21/2024 - 04:22:25

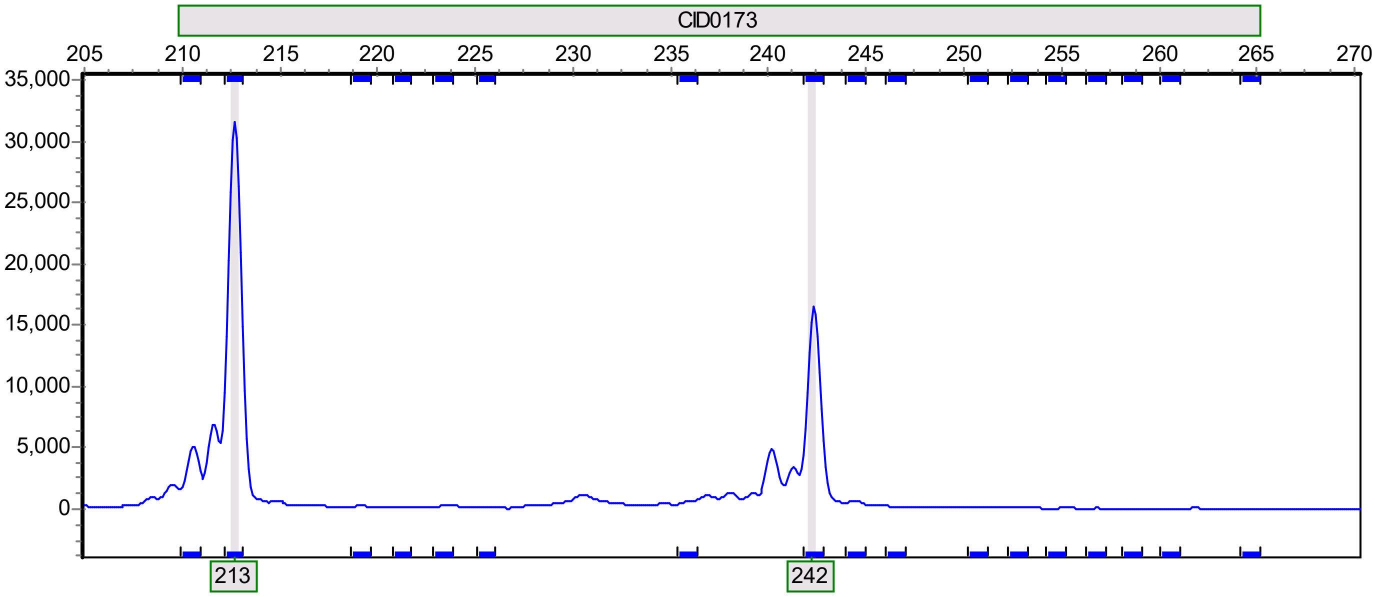

No	Size	Height	Area	Marker	Allele	Difference	Quality	Score	Allele Comments	Sample Comment
1	170.7	31963	221582	EST1573	171	0.10	Pass	500.0	[<Confirmed>]	
2	172.6	27291	179292	EST1573	173	0.10	Pass	500.0	[<Confirmed>]	
3	212.7	31630	235032	CID0173	213	0.00	Pass	500.0	[<Confirmed>]	
4	242.3	16474	123115	CID0173	242	0.10	Pass	500.0	[<Confirmed>]	

Sample 98: CID0173_EST1573_KC124_E18.fsa Run date and time: 09/21/2024 - 03:55:04 -> 09/21/2024 - 04:22:25

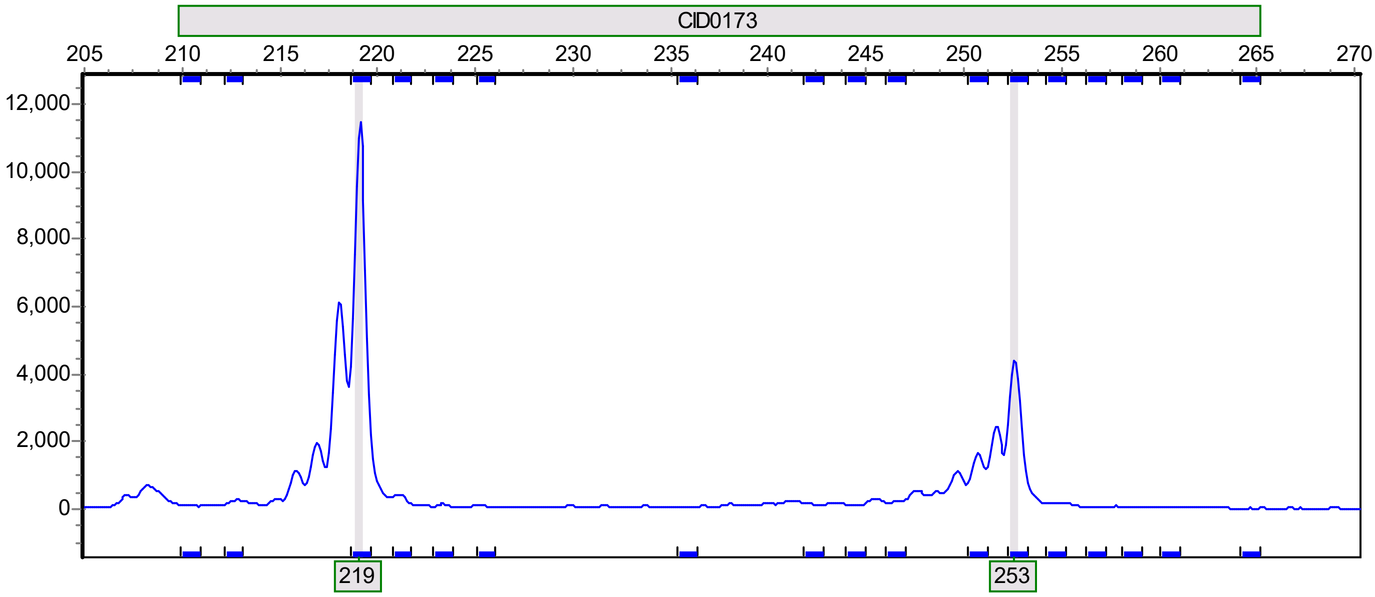

No	Size	Height	Area	Marker	Allele	Difference	Quality	Score	Allele Comments	Sample Comment
1	170.3	13363	91111	EST1573	171	0.30	Pass	500.0	[<Confirmed>]	
2	178.1	10938	73378	EST1573	178	0.10	Pass	500.0	[<Confirmed>]	
3	218.0	6101	41688	CID0173	218	0.10	Pass	500.0	[<Deleted>]	
4	219.1	11479	85631	CID0173	219	0.10	Pass	500.0	[<Confirmed>]	
5	252.6	4372	32652	CID0173	253	0.20	Pass	500.0	[<Confirmed>]	

Sample 99: CID0173_EST1573_KC125_G18.fsa Run date and time: 09/21/2024 - 03:55:04 -> 09/21/2024 - 04:22:25

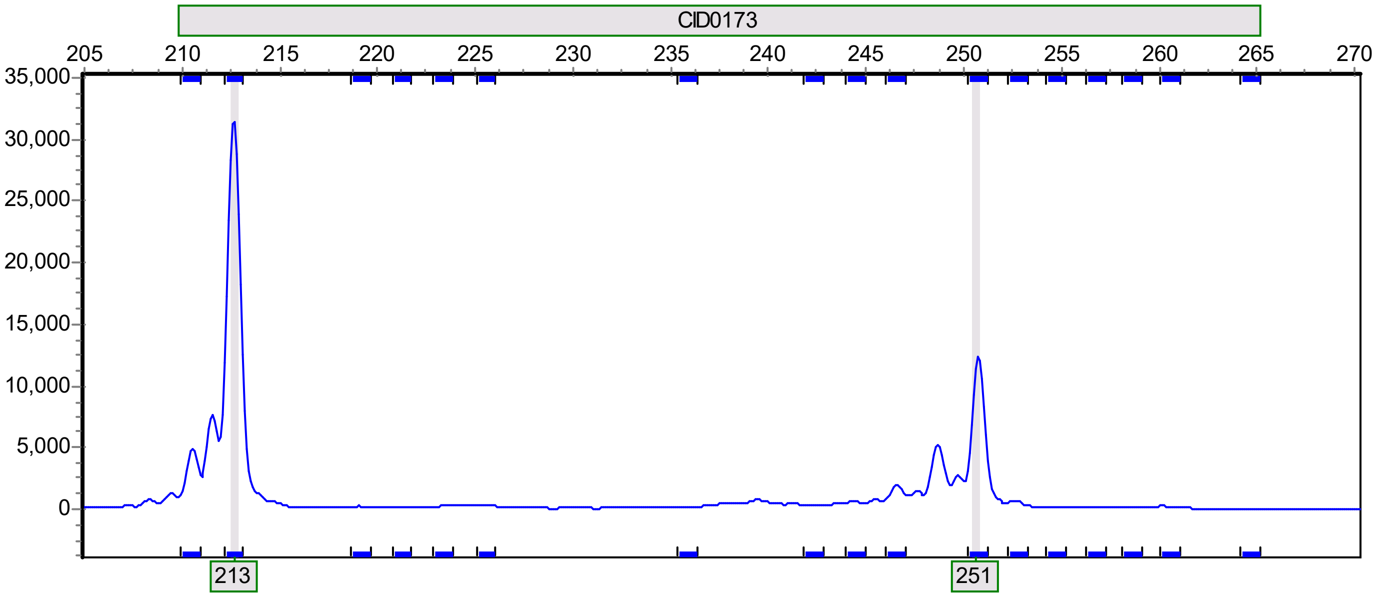

No	Size	Height	Area	Marker	Allele	Difference	Quality	Score	Allele Comments	Sample Comments
1	172.6	32053	234344	EST1573	173	0.10	Pass	500.0	[<Confirmed>]	
2	174.4	19082	128813	EST1573	174	0.00	Pass	500.0	[<Confirmed>]	
3	212.7	31434	244524	CID0173	213	0.00	Pass	500.0	[<Confirmed>]	
4	250.7	12390	86150	CID0173	251	0.10	Pass	500.0	[<Confirmed>]	

Sample 100: CID0173_EST1573_KC12_E21.fsa Run date and time: 09/21/2024 - 03:00:19 -> 09/21/2024 - 03:27:31

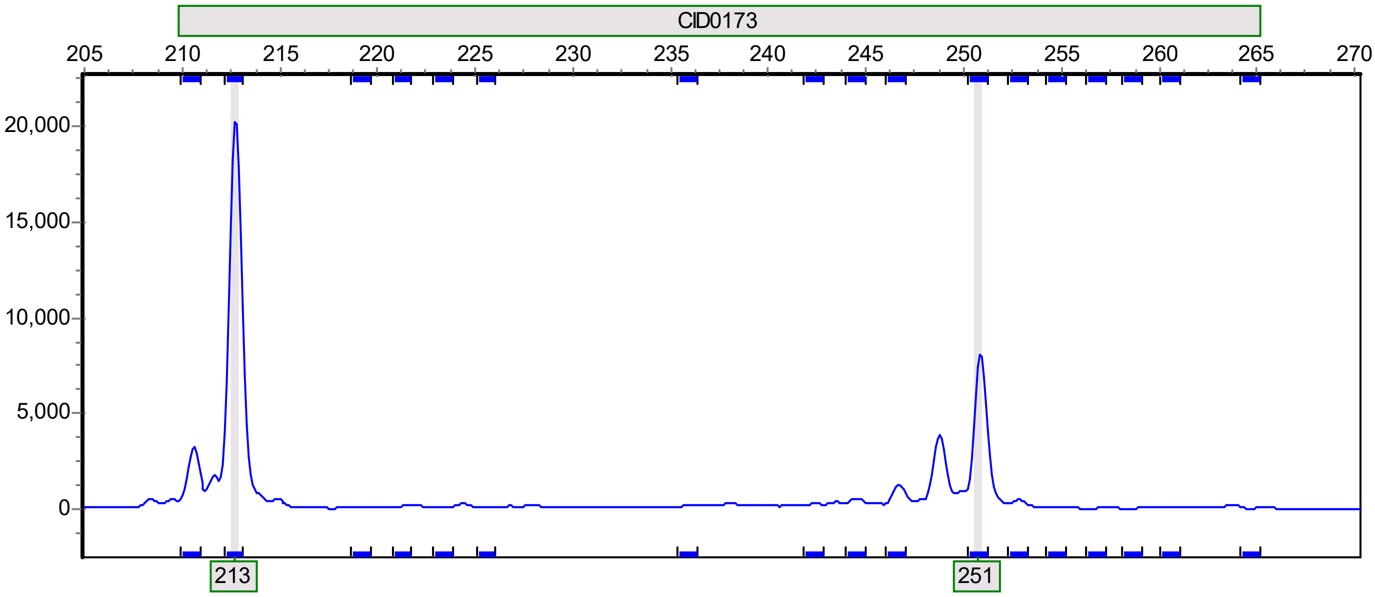

No	Size	Height	Area	Marker	Allele	Difference	Quality	Score	Allele Comments	Sample Comments
1	170.6	25029	166247	EST1573	171	0.00	Pass	500.0	[<Confirmed>]	
2	182.0	5768	40218	EST1573	182	0.00	Pass	500.0	[<Confirmed><Inserted>]	
3	212.7	20240	145310	CID0173	213	0.00	Pass	500.0	[<Confirmed>]	
4	250.8	8089	57866	CID0173	251	0.00	Pass	500.0	[<Confirmed>]	

Sample 101: CID0173_EST1573_KC13_G21.fsa Run date and time: 09/21/2024 - 03:00:19 -> 09/21/2024 - 03:27:31

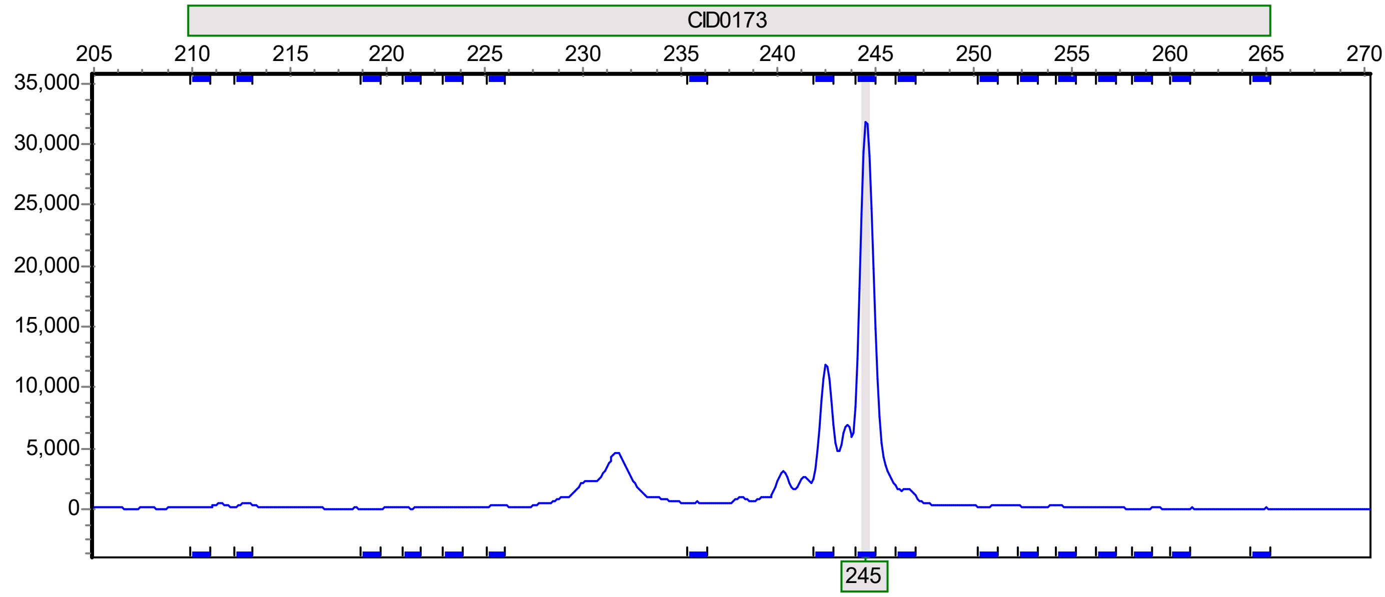

No	Size	Height	Area	Marker	Allele	Difference	Quality	Score	Allele Comments	Sample Comments
1	162.8	31611	225068	EST1573	163	0.10	Pass	500.0	[<Confirmed>]	
2	176.1	17610	119851	EST1573	176	0.30	Pass	500.0	[<Confirmed>]	
3	244.5	31846	265884	CID0173	245	0.00	Pass	500.0	[<SAT (Repaired)><Confirmed>]	

Sample 102: CID0173_EST1573_KC14_I21.fsa Run date and time: 09/21/2024 - 03:00:19 -> 09/21/2024 - 03:27:31

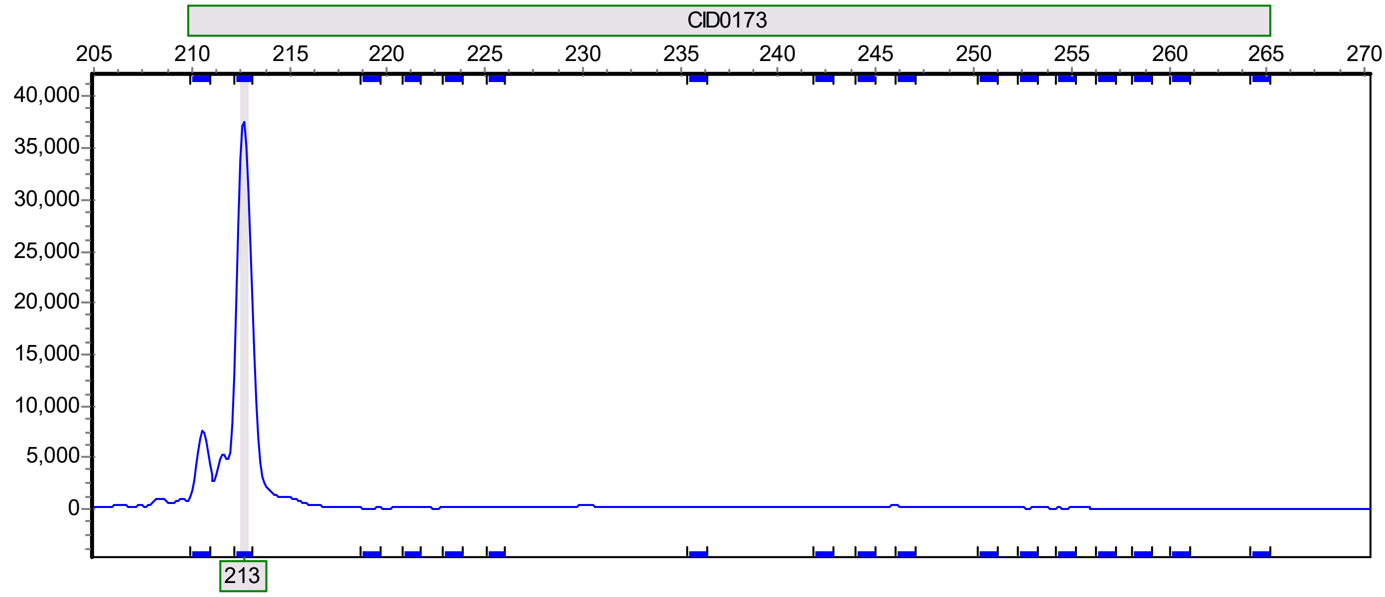

No	Size	Height	Area	Marker	Allele	Difference	Quality	Score	Allele Comments	Sample Comments
1	162.9	32384	223427	EST1573	163	0.00	Pass	500.0	[<Confirmed>]	
2	190.1	5705	41346	EST1573	190	0.10	Pass	500.0	[<Confirmed><Inserted>]	
3	212.7	37561	315374	CID0173	213	0.00	Pass	500.0	[<SAT (Repaired)><Confirmed>]	

Sample 103: CID0173_EST1573_KC15_K21.fsa Run date and time: 09/21/2024 - 03:00:19 -> 09/21/2024 - 03:27:31

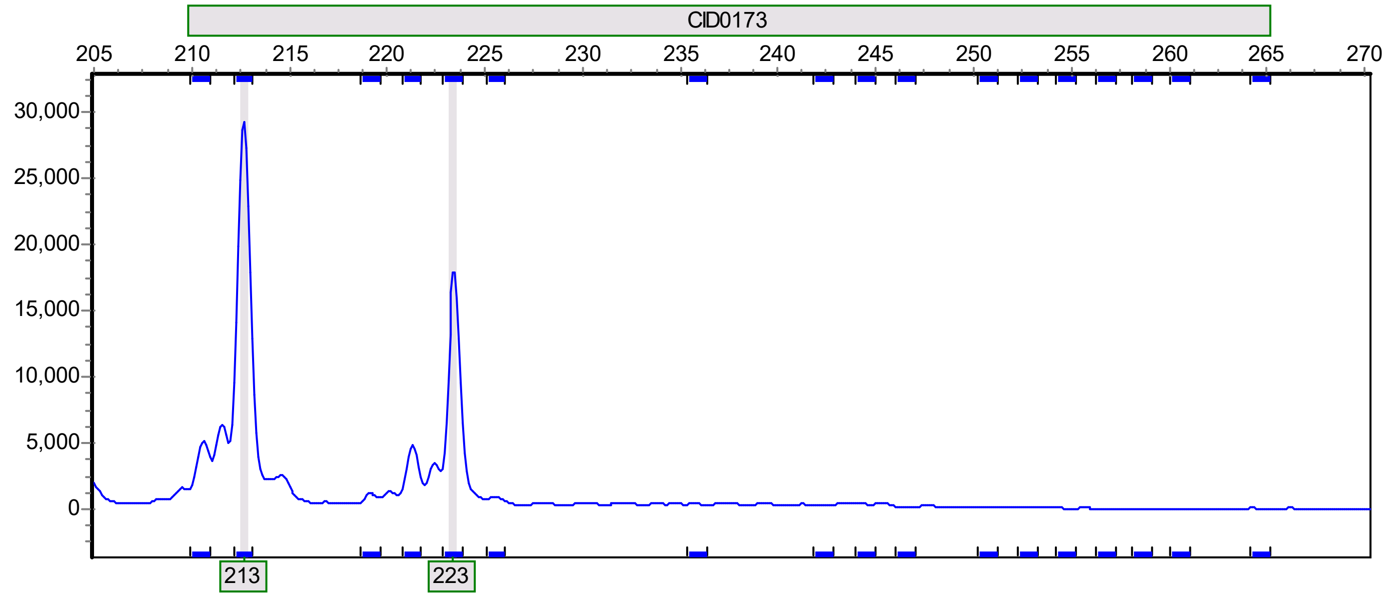

No	Size	Height	Area	Marker	Allele	Difference	Quality	Score	Allele Comments	Sample Comments
1	172.6	26544	177538	EST1573	173	0.10	Pass	500.0	[<Confirmed>]	
2	212.7	29290	224421	CID0173	213	0.00	Pass	500.0	[<Confirmed>]	
3	223.4	17940	129729	CID0173	223	0.00	Pass	500.0	[<Confirmed>]	

Sample 104: CID0173_EST1573_KC16_M21.fsa Run date and time: 09/21/2024 - 03:00:19 -> 09/21/2024 - 03:27:31

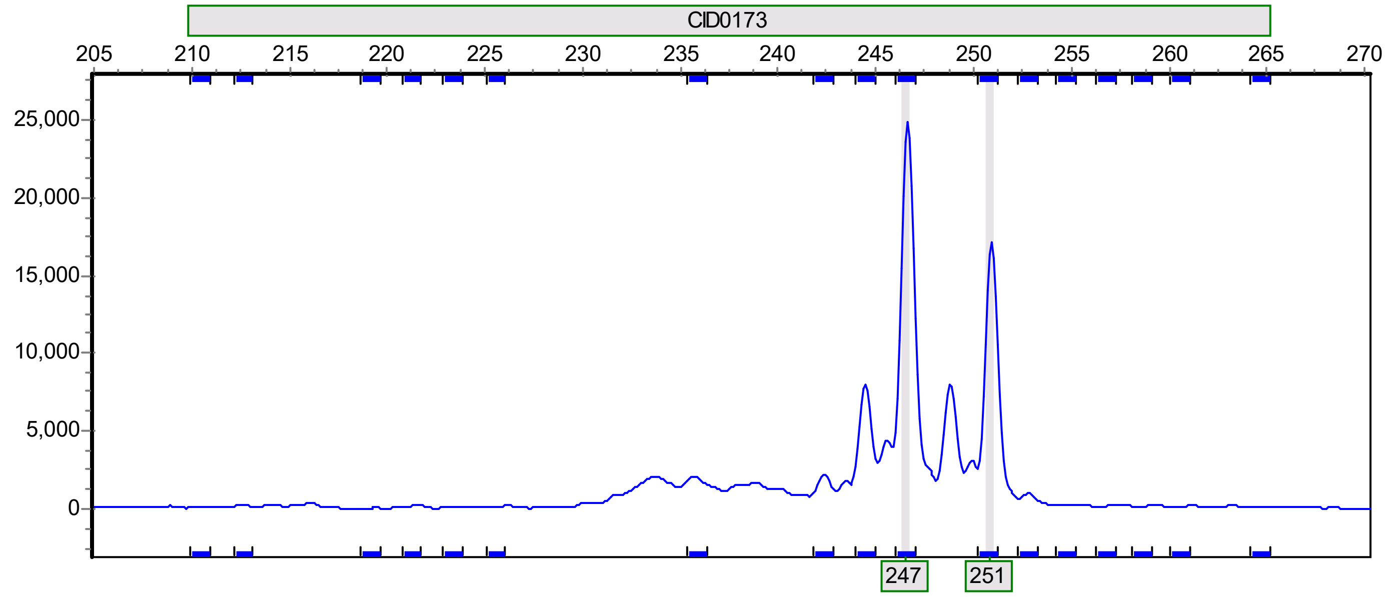

No	Size	Height	Area	Marker	Allele	Difference	Quality	Score	Allele Comments	Sample Comments
1	176.4	28832	225934	EST1573	176	0.00	Pass	500.0	[<SAT (Repaired)><Confirmed>]	
2	181.9	13444	89415	EST1573	182	0.10	Pass	500.0	[<Confirmed>]	
3	246.6	24841	192072	CID0173	247	0.00	Pass	500.0	[<Confirmed>]	
4	250.9	17147	124022	CID0173	251	0.10	Pass	500.0	[<Confirmed>]	

Sample 105: CID0173_EST1573_KC17_O21.fsa Run date and time: 09/21/2024 - 03:00:19 -> 09/21/2024 - 03:27:31

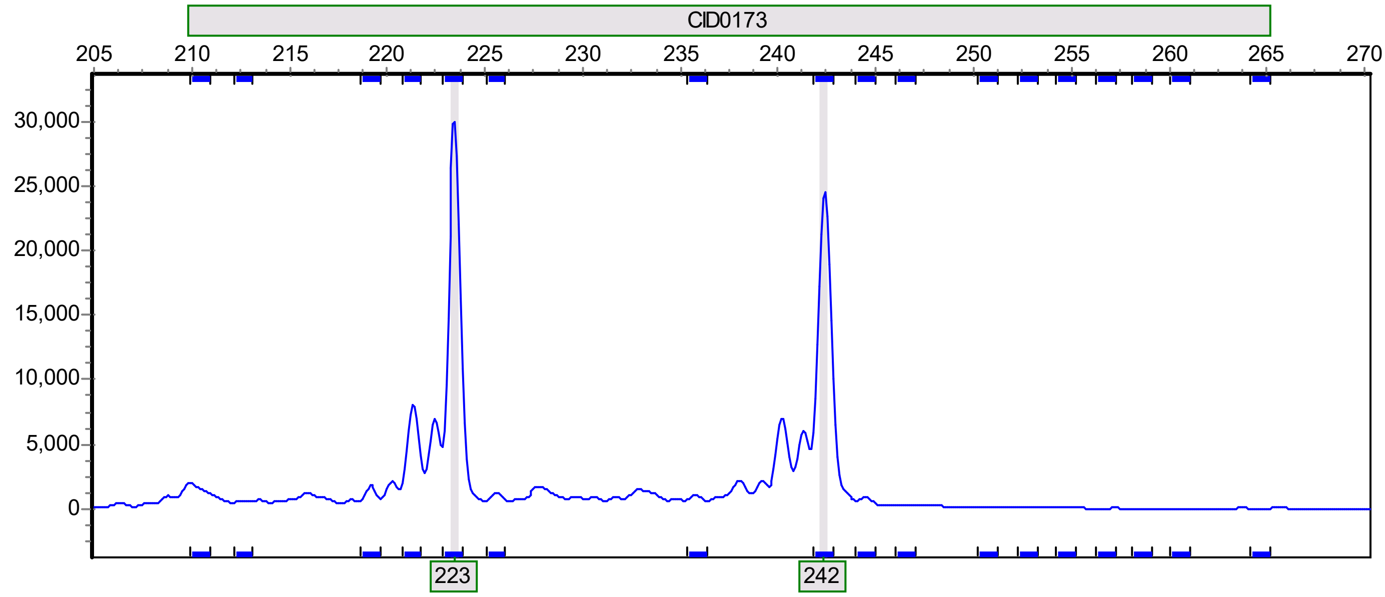

No	Size	Height	Area	Marker	Allele	Difference	Quality	Score	Allele Comments	Sample Comments
1	160.1	18365	127628	EST1573	160	0.20	Pass	500.0	[<Deleted>]	
2	170.6	44060	356167	EST1573	171	0.00	Pass	500.0	[<SAT (Repaired)><Confirmed>]	
3	223.5	29955	216482	CID0173	223	0.10	Pass	500.0	[<Confirmed>]	
4	242.4	24549	188648	CID0173	242	0.00	Pass	500.0	[<Confirmed>]	

Sample 106: CID0173_EST1573_KC18_A23.fsa Run date and time: 09/21/2024 - 03:00:19 -> 09/21/2024 - 03:27:31

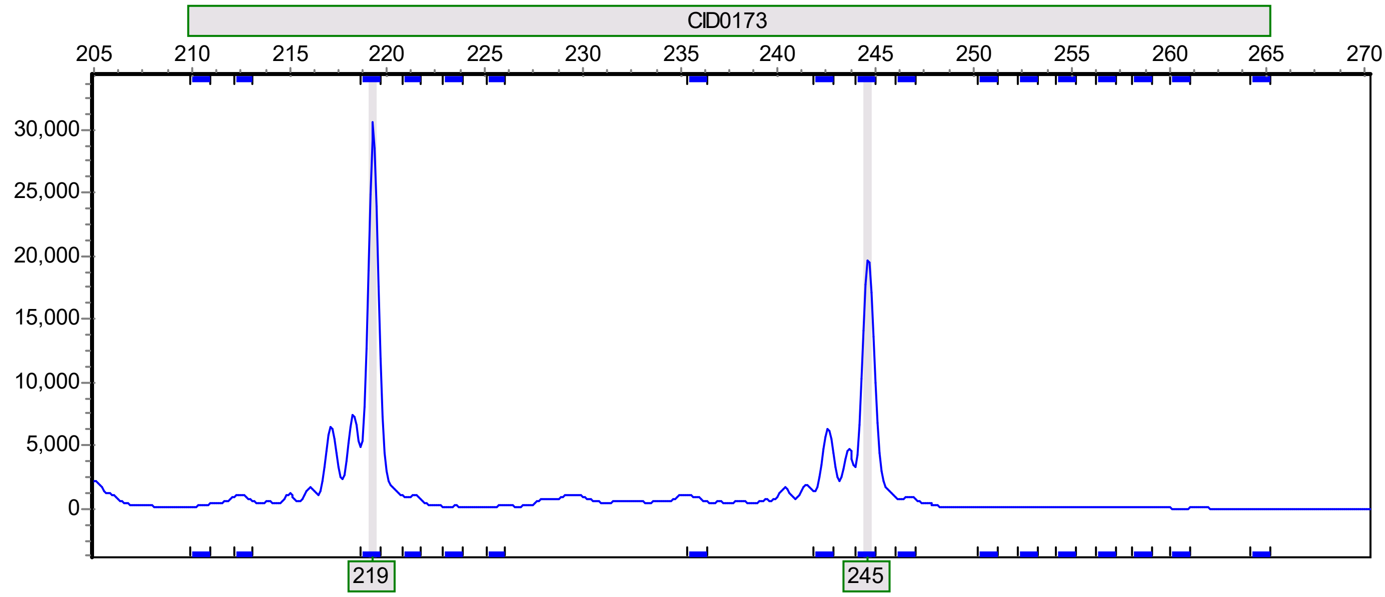

No	Size	Height	Area	Marker	Allele	Difference	Quality	Score	Allele Comments	Sample Comments
1	172.5	28819	218149	EST1573	173	0.00	Pass	500.0	[<SAT (Repaired)><Confirmed>]	
2	174.5	23634	153814	EST1573	174	0.10	Pass	500.0	[<Confirmed>]	
3	219.3	30582	213669	CID0173	219	0.10	Pass	500.0	[<Confirmed>]	
4	244.6	19609	138463	CID0173	245	0.10	Pass	500.0	[<Confirmed>]	

Sample 107: CID0173_EST1573_KC19_C23.fsa Run date and time: 09/21/2024 - 03:00:19 -> 09/21/2024 - 03:27:31

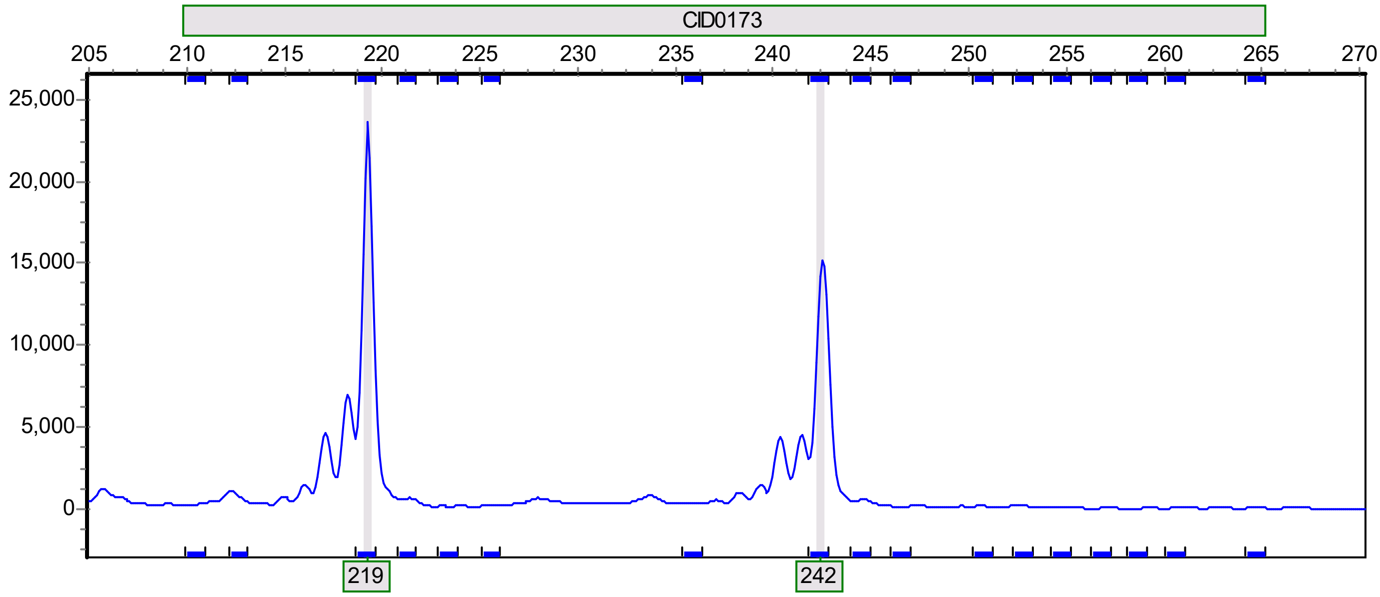

No	Size	Height	Area	Marker	Allele	Difference	Quality	Score	Allele Comments	Sample Comments
1	164.8	31349	205129	EST1573	165	0.00	Pass	500.0	[<Confirmed>]	
2	174.5	26924	171502	EST1573	174	0.10	Pass	500.0	[<Confirmed>]	
3	219.3	23637	163891	CID0173	219	0.10	Pass	500.0	[<Confirmed>]	
4	242.5	15208	112636	CID0173	242	0.10	Pass	500.0	[<Confirmed>]	

Sample 108: CID0173_EST1573_KC1_O17.fsa Run date and time: 09/21/2024 - 03:00:19 -> 09/21/2024 - 03:27:31

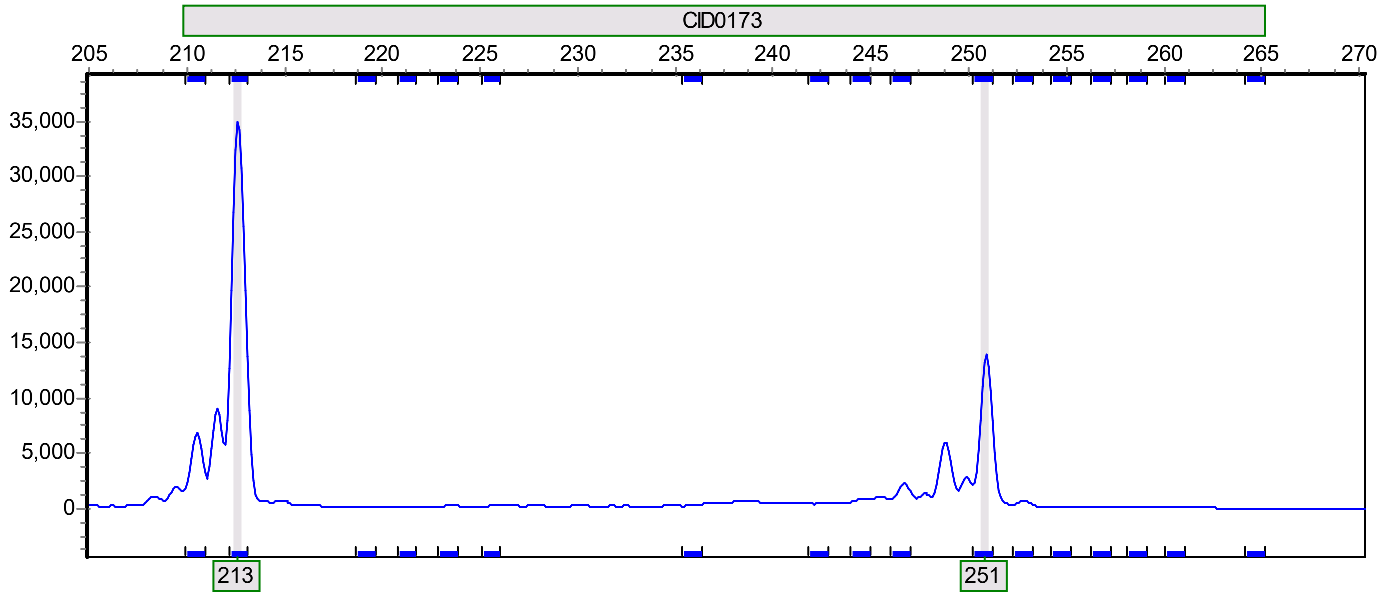

No	Size	Height	Area	Marker	Allele	Difference	Quality	Score	Allele Comments	Sample Comments
1	162.8	38052	253000	EST1573	163	0.10	Pass	500.0	[<SAT (Repaired)><Confirmed>]	
2	164.8	31334	207200	EST1573	165	0.00	Pass	500.0	[<Confirmed>]	
3	212.6	34926	265930	CID0173	213	0.10	Pass	500.0	[<SAT (Repaired)><Confirmed>]	
4	250.9	13984	93294	CID0173	251	0.10	Pass	500.0	[<Confirmed>]	

Sample 109: CID0173_EST1573_KC20_E23.fsa Run date and time: 09/21/2024 - 03:00:19 -> 09/21/2024 - 03:27:31

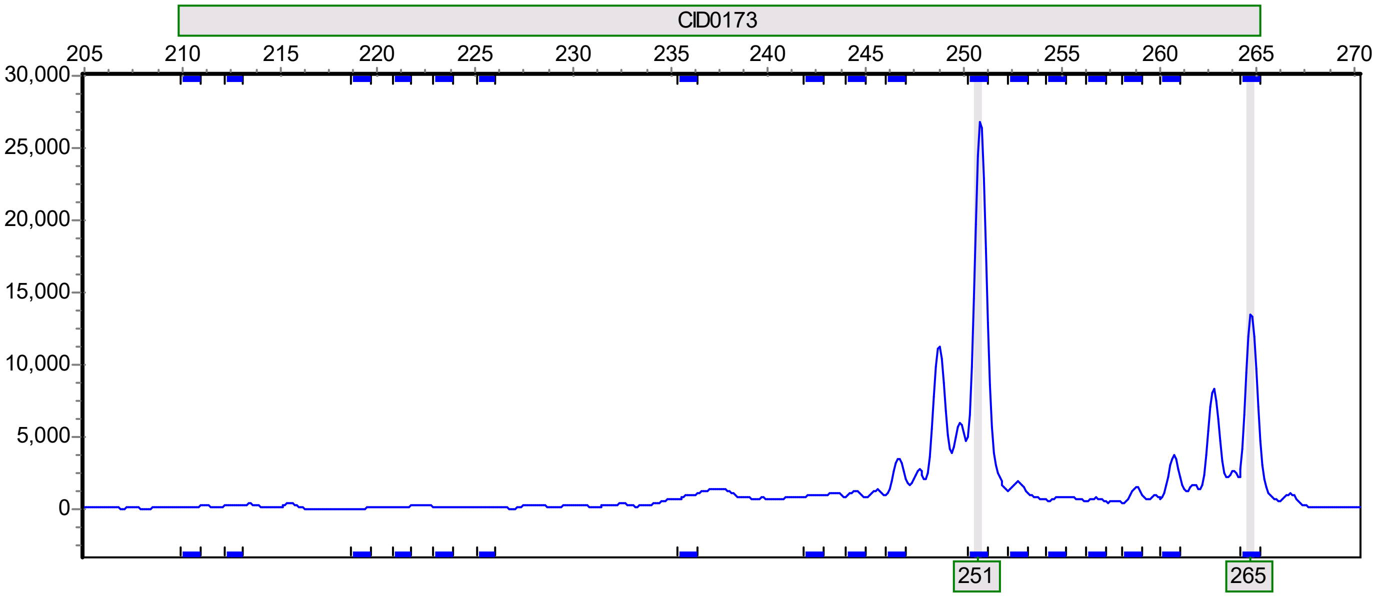

No	Size	Height	Area	Marker	Allele	Difference	Quality	Score	Allele Comments	Sample Comments
1	162.9	34301	263457	EST1573	163	0.00	Pass	500.0	[<SAT (Repaired)><Confirmed>]	
2	170.6	29409	235983	EST1573	171	0.00	Pass	500.0	[<SAT (Repaired)><Confirmed>]	
3	250.8	26835	196596	CID0173	251	0.00	Pass	500.0	[<Confirmed>]	
4	264.7	13468	94704	CID0173	265	0.00	Pass	500.0	[<Confirmed>]	

Sample 110: CID0173_EST1573_KC21_G23.fsa Run date and time: 09/21/2024 - 03:00:19 -> 09/21/2024 - 03:27:31

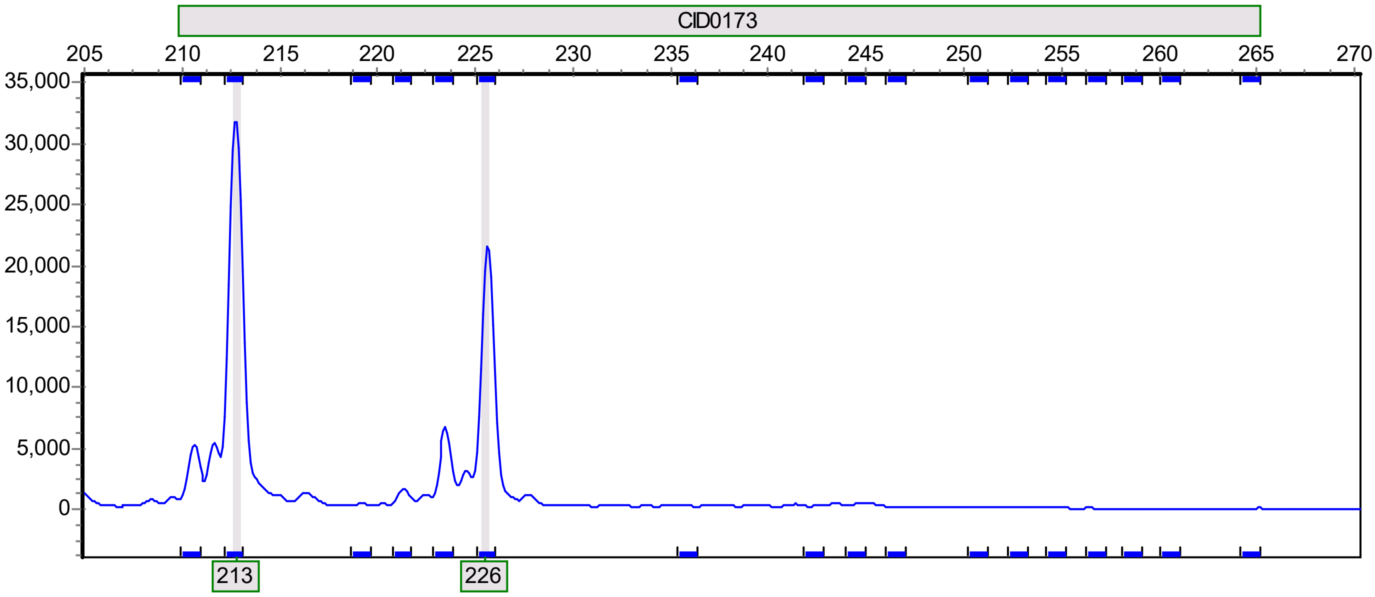

No	Size	Height	Area	Marker	Allele	Difference	Quality	Score	Allele Comments	Sample Comments
1	172.6	31826	245624	EST1573	173	0.10	Pass	500.0	[<Confirmed>]	
2	174.4	19422	131797	EST1573	174	0.00	Pass	500.0	[<Confirmed>]	
3	212.8	31783	254495	CID0173	213	0.10	Pass	500.0	[<Confirmed>]	
4	225.6	21643	155663	CID0173	226	0.00	Pass	500.0	[<Confirmed>]	

Sample 111: CID0173_EST1573_KC22_I23.fsa Run date and time: 09/21/2024 - 03:00:19 -> 09/21/2024 - 03:27:31

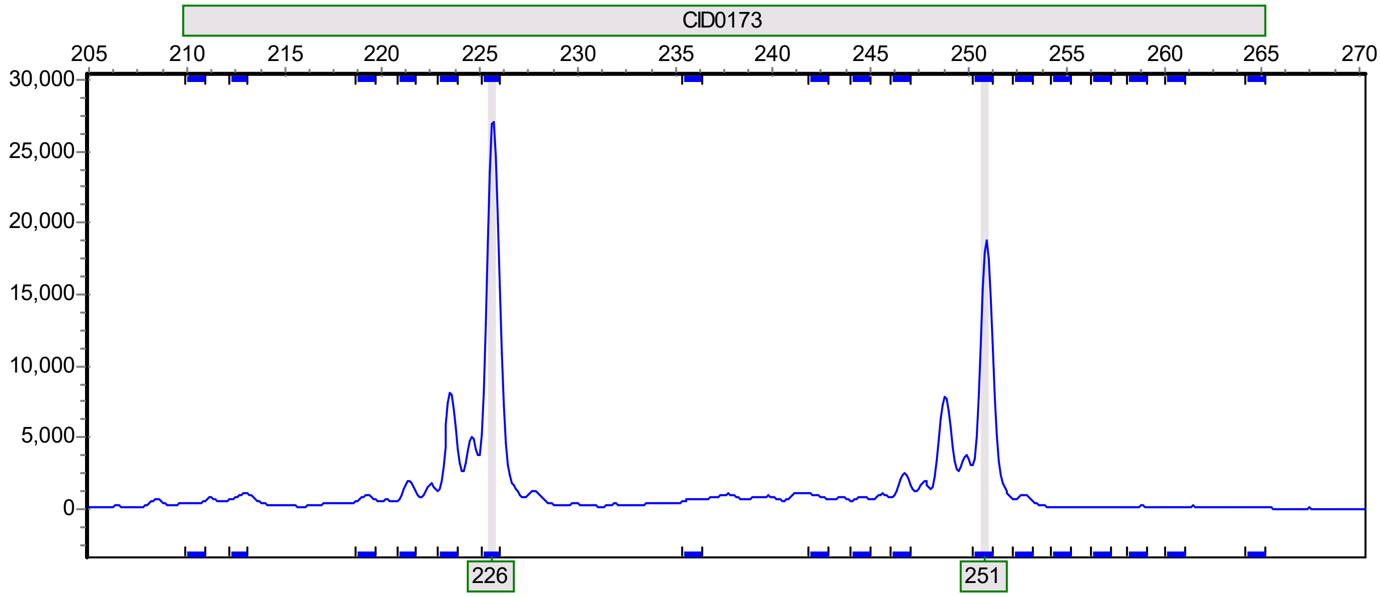

No	Size	Height	Area	Marker	Allele	Difference	Quality	Score	Allele Comments	Sample Comments
1	174.3	31575	218071	EST1573	174	0.10	Pass	500.0	[<Confirmed>]	
2	182.0	15071	102405	EST1573	182	0.00	Pass	500.0	[<Confirmed>]	
3	225.7	27032	198288	CID0173	226	0.10	Pass	500.0	[<Confirmed>]	
4	250.9	18788	135404	CID0173	251	0.10	Pass	500.0	[<Confirmed>]	

Sample 112: CID0173_EST1573_KC23_K23.fsa Run date and time: 09/21/2024 - 03:00:19 -> 09/21/2024 - 03:27:31

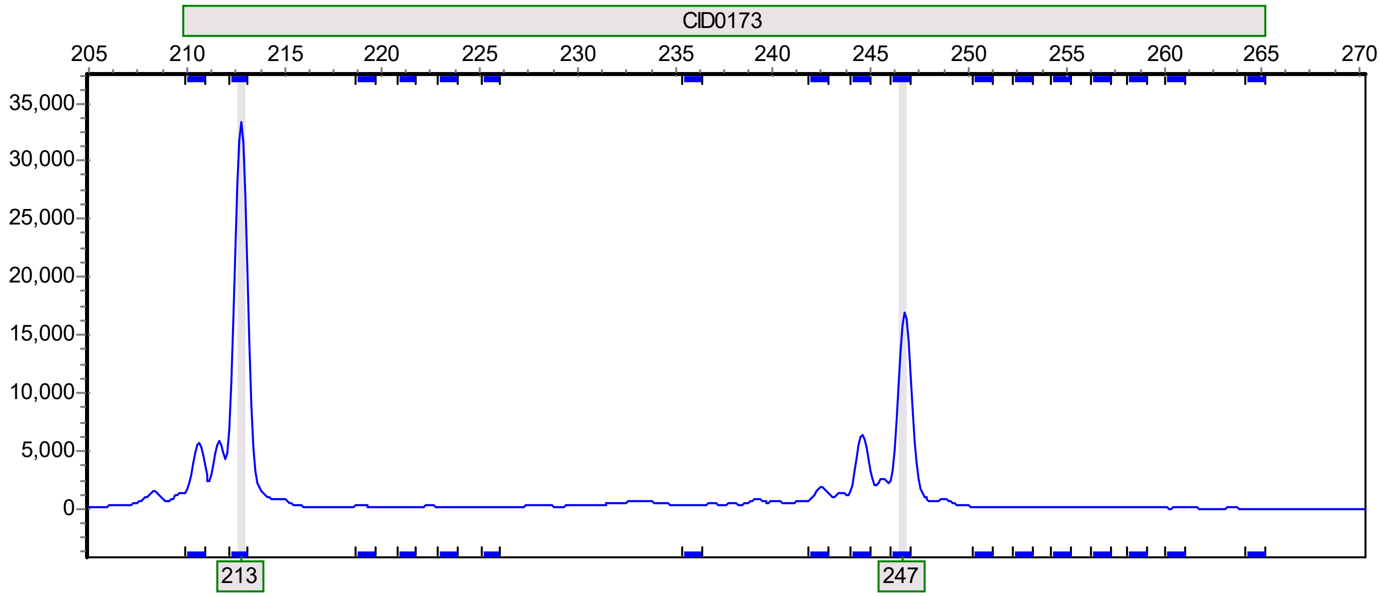

No	Size	Height	Area	Marker	Allele	Difference	Quality	Score	Allele Comments	Sample Comments
1	174.4	31735	248793	EST1573	174	0.00	Pass	500.0	[<SAT (Repaired)><Confirmed>]	
2	212.8	33357	250208	CID0173	213	0.10	Pass	500.0	[<SAT (Repaired)><Confirmed>]	
3	246.7	16940	132030	CID0173	247	0.10	Pass	500.0	[<Confirmed>]	

Allele Report

Sample 113: CID0173_EST1573_KC24_M23.fsa Run date and time: 09/21/2024 - 03:00:19 -> 09/21/2024 - 03:27:31

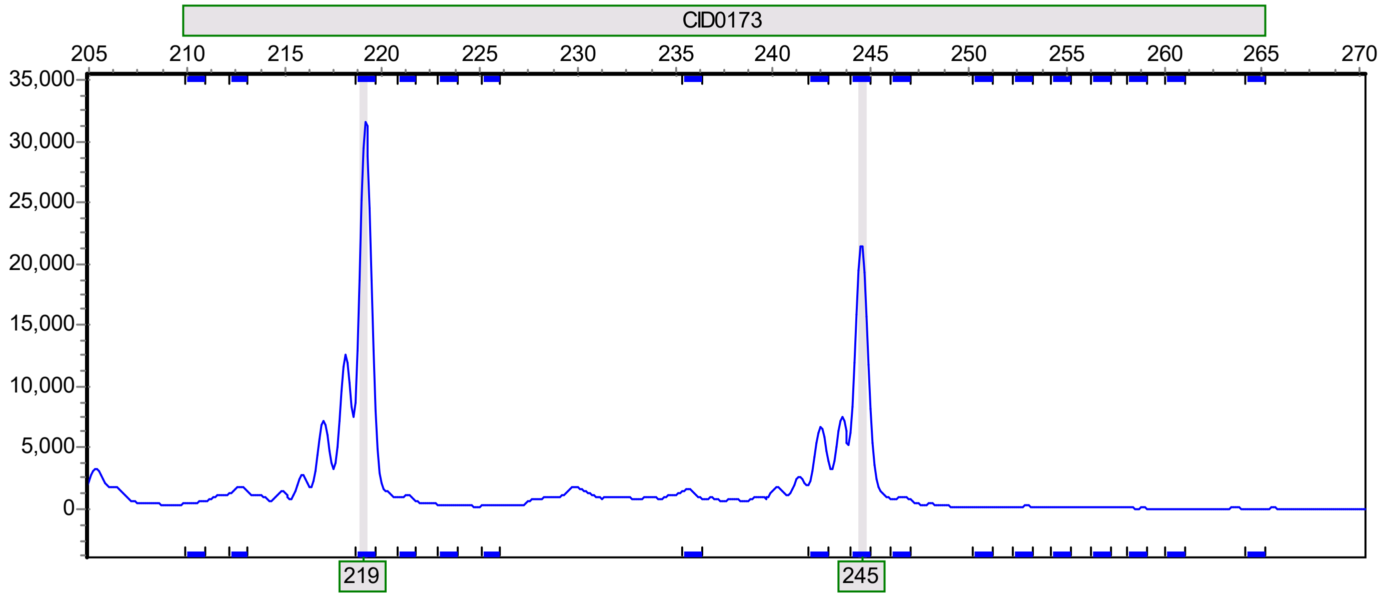

No	Size	Height	Area	Marker	Allele	Difference	Quality	Score	Allele Comments	Sample Comments
1	164.8	33857	240808	EST1573	165	0.00	Pass	500.0	[<SAT (Repaired)><Confirmed>]	
2	218.1	12540	75960	CID0173	218	0.00	Undetermined	500.0	[<Deleted>]	
3	219.1	31559	250112	CID0173	219	0.10	Pass	500.0	[<Confirmed>]	
4	244.6	21483	164829	CID0173	245	0.10	Pass	500.0	[<Confirmed>]	

Sample 114: CID0173_EST1573_KC25_O23.fsa Run date and time: 09/21/2024 - 03:00:19 -> 09/21/2024 - 03:27:31

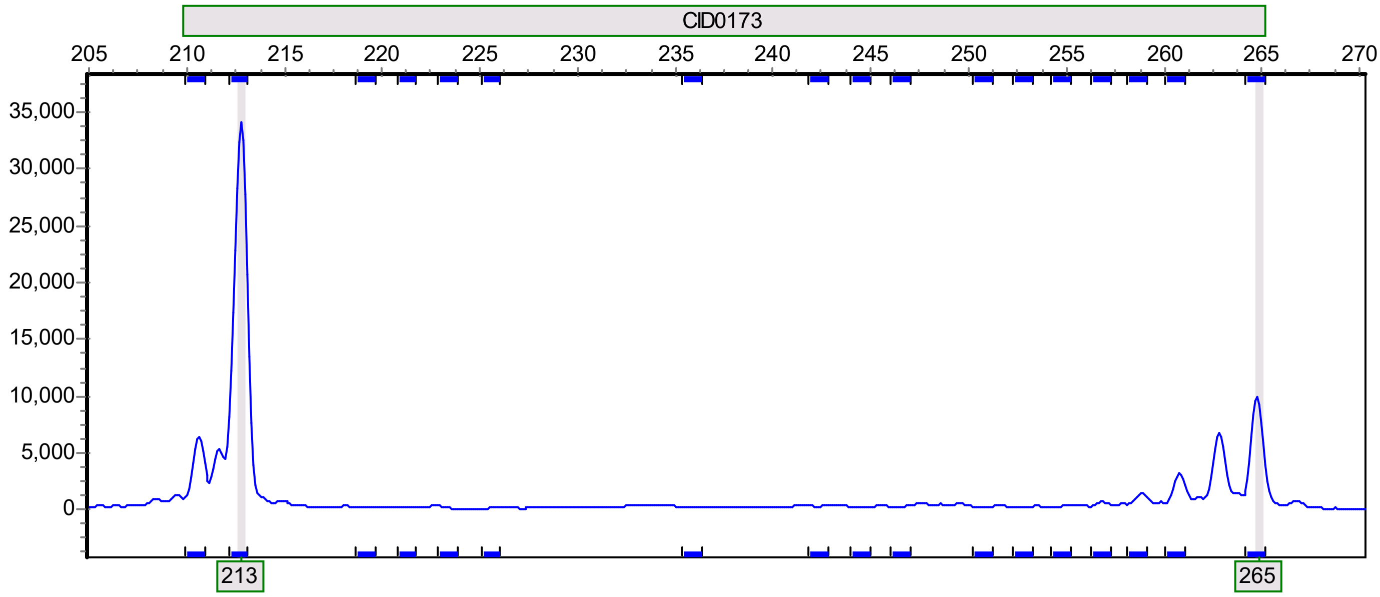

No	Size	Height	Area	Marker	Allele	Difference	Quality	Score	Allele Comments	Sample Comments
1	182.0	14949	102695	EST1573	182	0.00	Pass	500.0	[<Confirmed>]	
2	190.0	12912	87289	EST1573	190	0.00	Pass	500.0	[<Confirmed>]	
3	212.8	34114	254794	CID0173	213	0.10	Pass	500.0	[<SAT (Repaired)><Confirmed>]	
4	264.9	9251	68335	CID0173	265	0.20	Pass	500.0	[<Confirmed><Inserted>]	

Sample 115: CID0173_EST1573_KC26_B01.fsa Run date and time: 09/21/2024 - 03:27:32 -> 09/21/2024 - 03:55:03

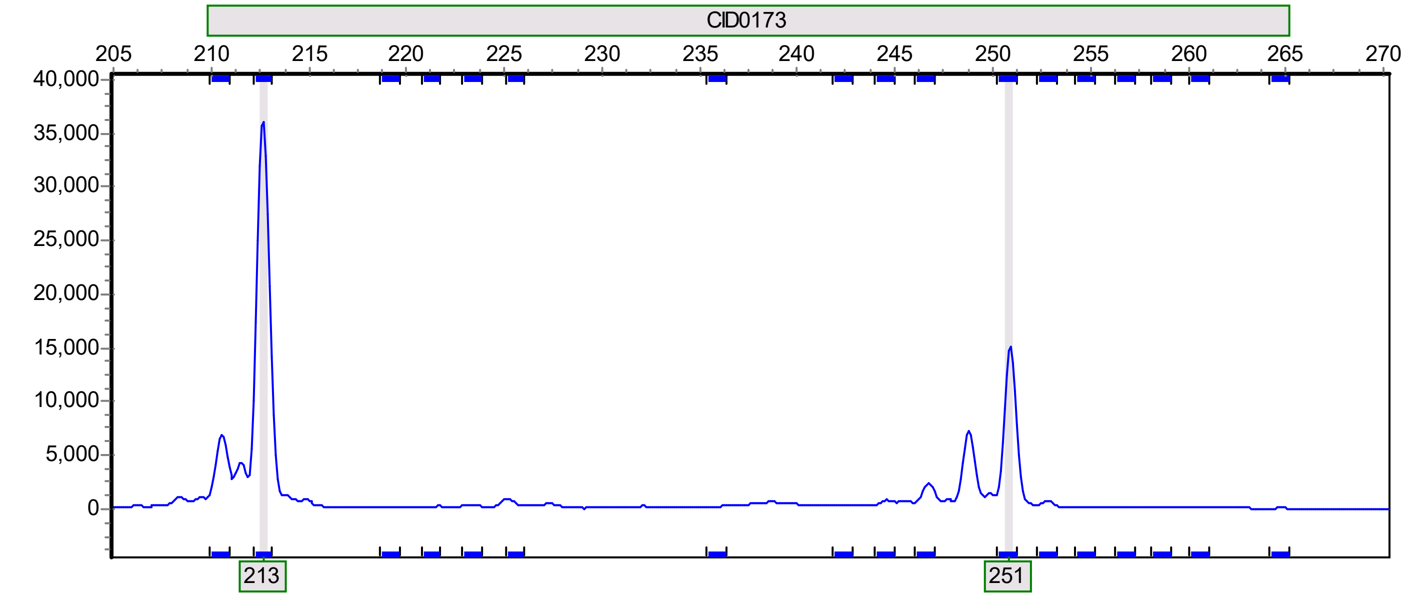

No	Size	Height	Area	Marker	Allele	Difference	Quality	Score	Allele Comments	Sample Comments
1	162.9	31363	213165	EST1573	163	0.00	Pass	500.0	[<Confirmed>]	
2	170.6	31536	212131	EST1573	171	0.00	Pass	500.0	[<Confirmed>]	
3	212.7	36056	259242	CID0173	213	0.00	Pass	500.0	[<SAT (Repaired)><Confirmed>]	
4	250.9	15151	99453	CID0173	251	0.10	Pass	500.0	[<Confirmed>]	

Sample 116: CID0173_EST1573_KC27_D01.fsa Run date and time: 09/21/2024 - 03:27:32 -> 09/21/2024 - 03:55:03

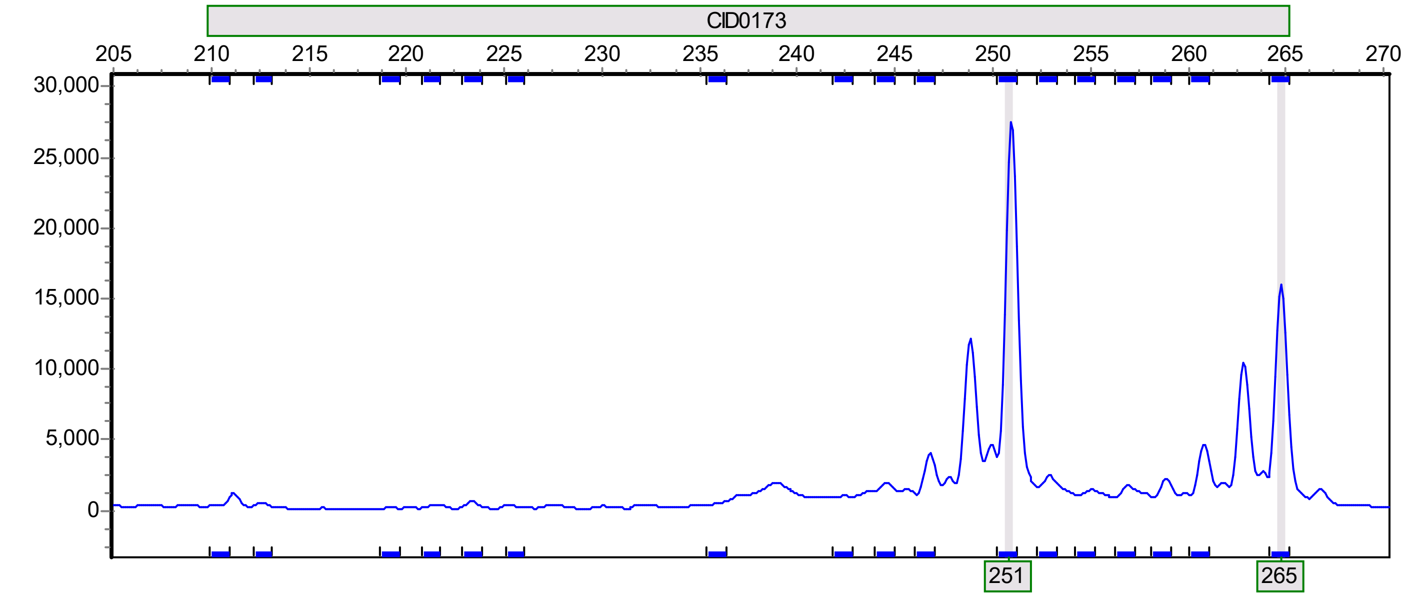

No	Size	Height	Area	Marker	Allele	Difference	Quality	Score	Allele Comments	Sample Comments
1	162.8	31116	247516	EST1573	163	0.10	Pass	500.0	[<SAT (Repaired)><Confirmed>]	
2	190.0	18656	125745	EST1573	190	0.00	Pass	500.0	[<Confirmed>]	
3	250.9	27485	189970	CID0173	251	0.10	Pass	500.0	[<Confirmed>]	
4	264.8	15950	113179	CID0173	265	0.10	Pass	500.0	[<Confirmed>]	

Sample 117: CID0173_EST1573_KC28_F01.fsa Run date and time: 09/21/2024 - 03:27:32 -> 09/21/2024 - 03:55:03

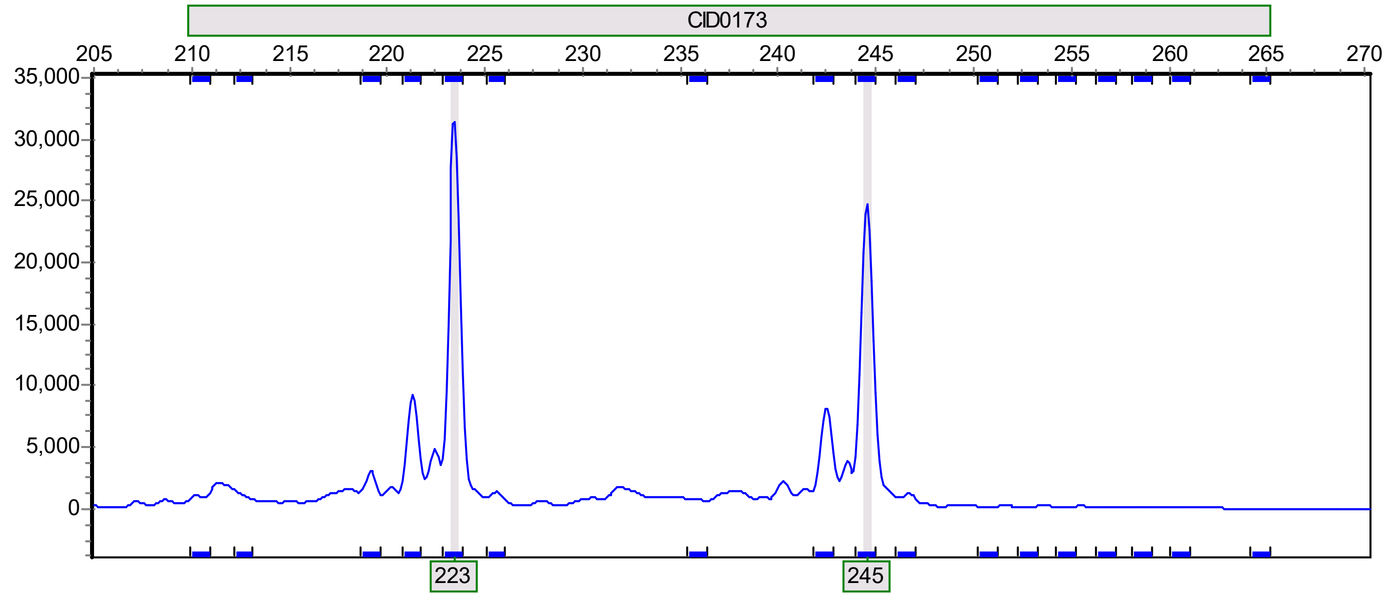

No	Size	Height	Area	Marker	Allele	Difference	Quality	Score	Allele Comments	Sample Comments
1	172.6	40445	277477	EST1573	173	0.10	Pass	500.0	[<SAT (Repaired)><Confirmed>]	
2	187.9	14001	92618	EST1573	188	0.10	Pass	500.0	[<Confirmed>]	
3	223.5	31389	222905	CID0173	223	0.10	Pass	500.0	[<Confirmed>]	
4	244.6	24744	173679	CID0173	245	0.10	Pass	500.0	[<Confirmed>]	

Sample 118: CID0173_EST1573_KC29_H01.fsa Run date and time: 09/21/2024 - 03:27:32 -> 09/21/2024 - 03:55:03

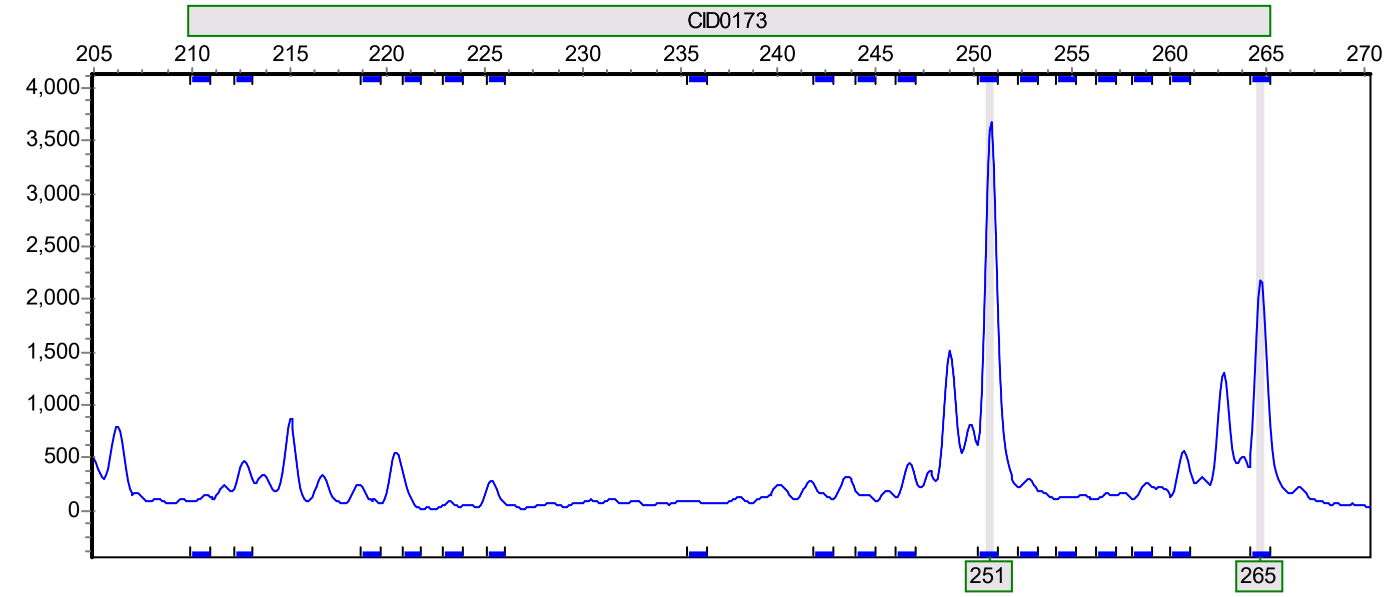

No	Size	Height	Area	Marker	Allele	Difference	Quality	Score	Allele Comments	Sample Comments
1	170.8	10091	66754	EST1573	171	0.20	Pass	500.0	[<Confirmed>]	
2	182.1	3428	21846	EST1573	182	0.10	Pass	500.0	[<Confirmed>]	
3	250.9	3676	25102	CID0173	251	0.10	Pass	500.0	[<Confirmed>]	
4	264.7	2185	15940	CID0173	265	0.00	Pass	354.5	[<Confirmed>]	

Allele Report

Sample 119: CID0173_EST1573_KC2_A19.fsa Run date and time: 09/21/2024 - 03:00:19 -> 09/21/2024 - 03:27:31

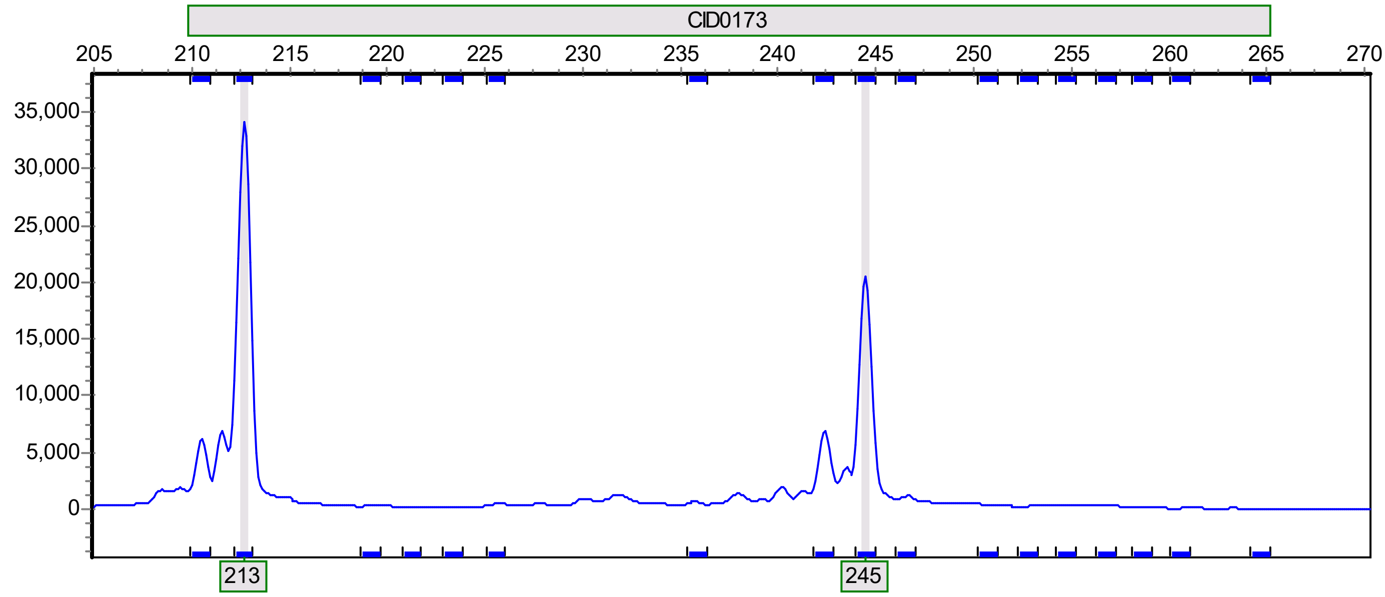

No	Size	Height	Area	Marker	Allele	Difference	Quality	Score	Allele Comments	Sample Comment
1	176.3	25436	171690	EST1573	176	0.10	Pass	500.0	[<Confirmed>]	
2	182.0	12169	81158	EST1573	182	0.00	Pass	500.0	[<Confirmed>]	
3	212.7	34146	256457	CID0173	213	0.00	Pass	500.0	[<SAT (Repaired)><Confirmed>]	
4	244.5	20608	149031	CID0173	245	0.00	Pass	500.0	[<Confirmed>]	

Sample 120: CID0173_EST1573_KC30_J01.fsa Run date and time: 09/21/2024 - 03:27:32 -> 09/21/2024 - 03:55:03

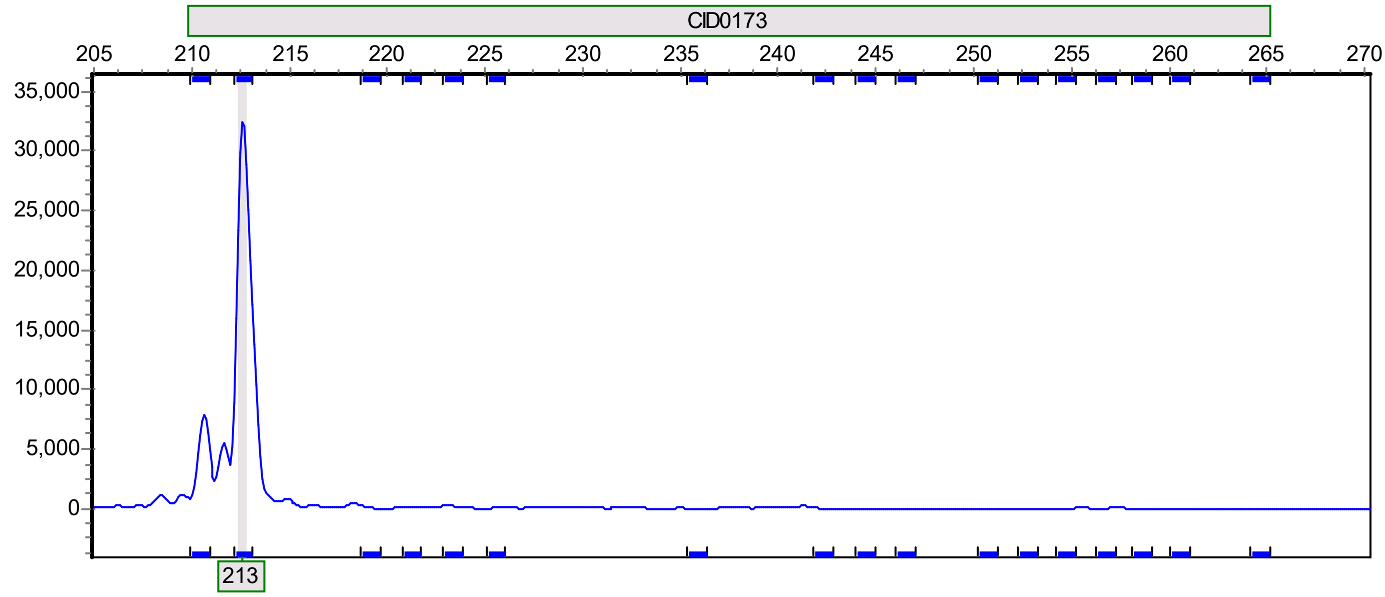

No	Size	Height	Area	Marker	Allele	Difference	Quality	Score	Allele Comments	Sample Comment
1	163.0	26128	160984	EST1573	163	0.10	Pass	500.0	[<Confirmed>]	
2	170.8	25077	156367	EST1573	171	0.20	Pass	500.0	[<Confirmed>]	
3	212.6	32425	264517	CID0173	213	0.10	Pass	500.0	[<SAT (Repaired)><Confirmed>]	

Sample 121: CID0173_EST1573_KC31_L01.fsa Run date and time: 09/21/2024 - 03:27:32 -> 09/21/2024 - 03:55:03

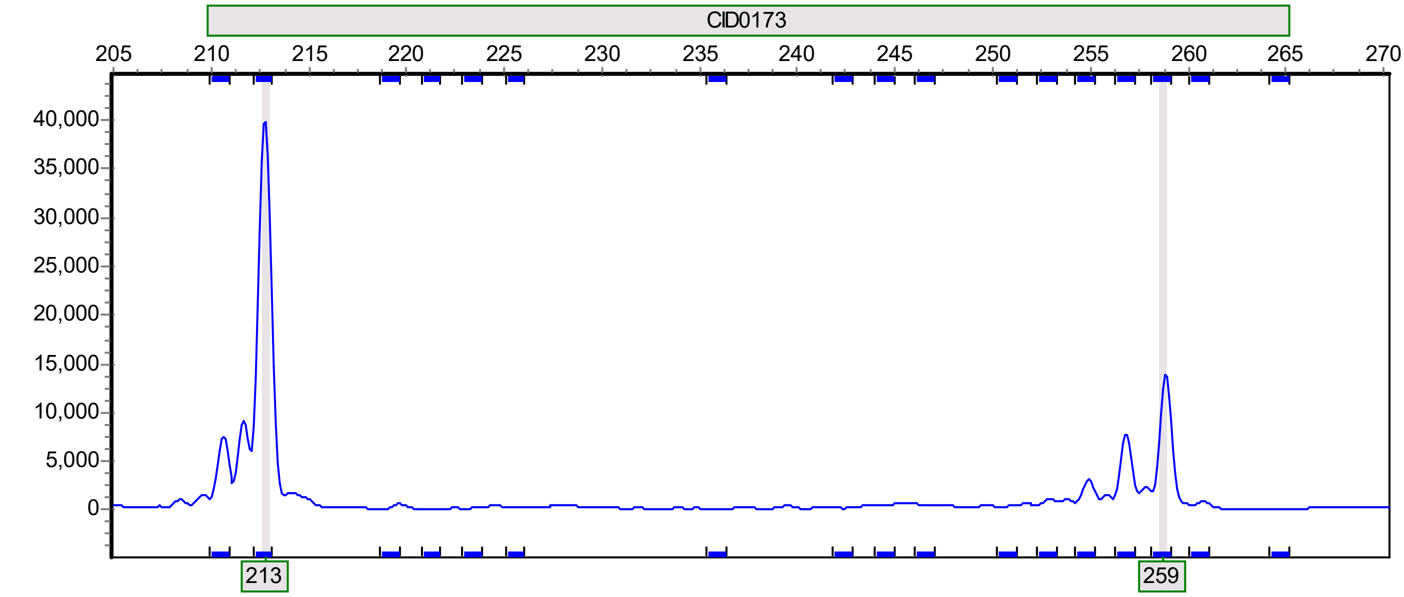

No	Size	Height	Area	Marker	Allele	Difference	Quality	Score	Allele Comments	Sample Comments
1	174.4	30978	232199	EST1573	174	0.00	Pass	500.0	[<Confirmed>]	
2	184.0	17341	111875	EST1573	184	0.00	Pass	500.0	[<Confirmed>]	
3	212.8	39795	294187	CID0173	213	0.10	Pass	500.0	[<SAT (Repaired)><Confirmed>]	
4	258.8	13826	93044	CID0173	259	0.10	Pass	500.0	[<Confirmed>]	

Sample 122: CID0173_EST1573_KC32_N01.fsa Run date and time: 09/21/2024 - 03:27:32 -> 09/21/2024 - 03:55:03

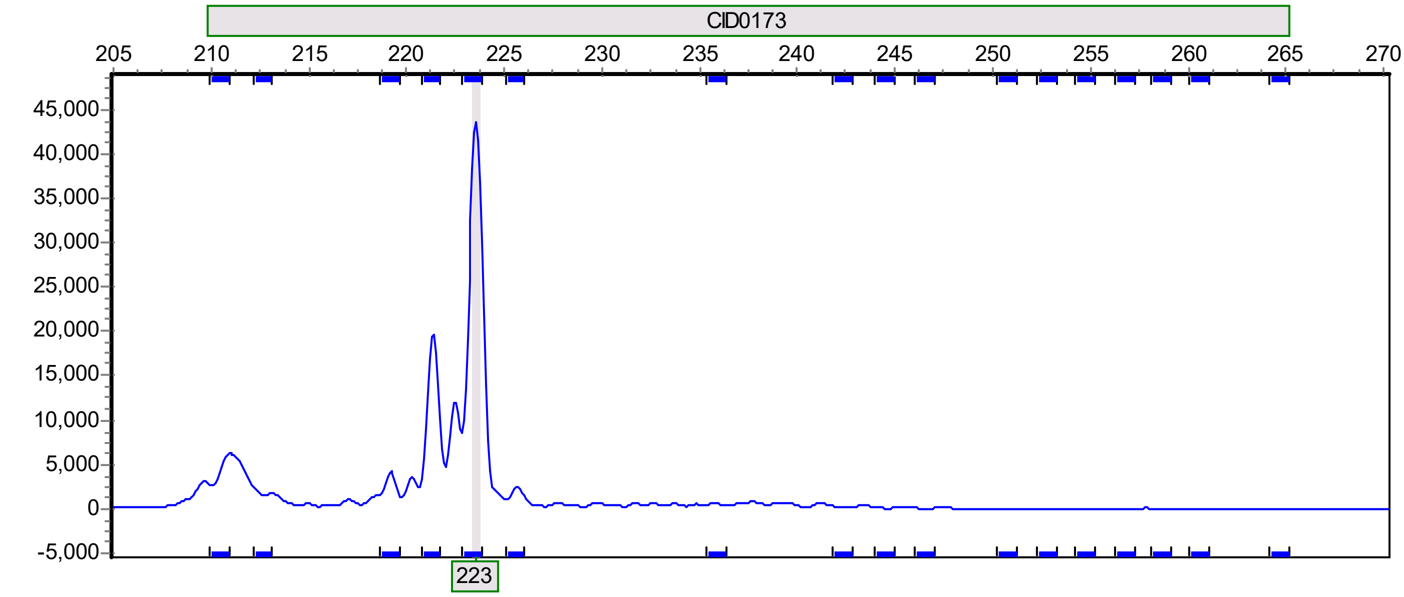

No	Size	Height	Area	Marker	Allele	Difference	Quality	Score	Allele Comments	Sample Comments
1	160.4	17600	120882	EST1573	160	0.10	Pass	500.0	[<Deleted>]	
2	170.6	47033	385477	EST1573	171	0.00	Pass	500.0	[<SAT (Repaired)><Confirmed>]	
3	223.6	43545	361020	CID0173	223	0.20	Pass	500.0	[<SAT (Repaired)><Confirmed>]	

Sample 123: CID0173_EST1573_KC33_P01.fsa Run date and time: 09/21/2024 - 03:27:32 -> 09/21/2024 - 03:55:03

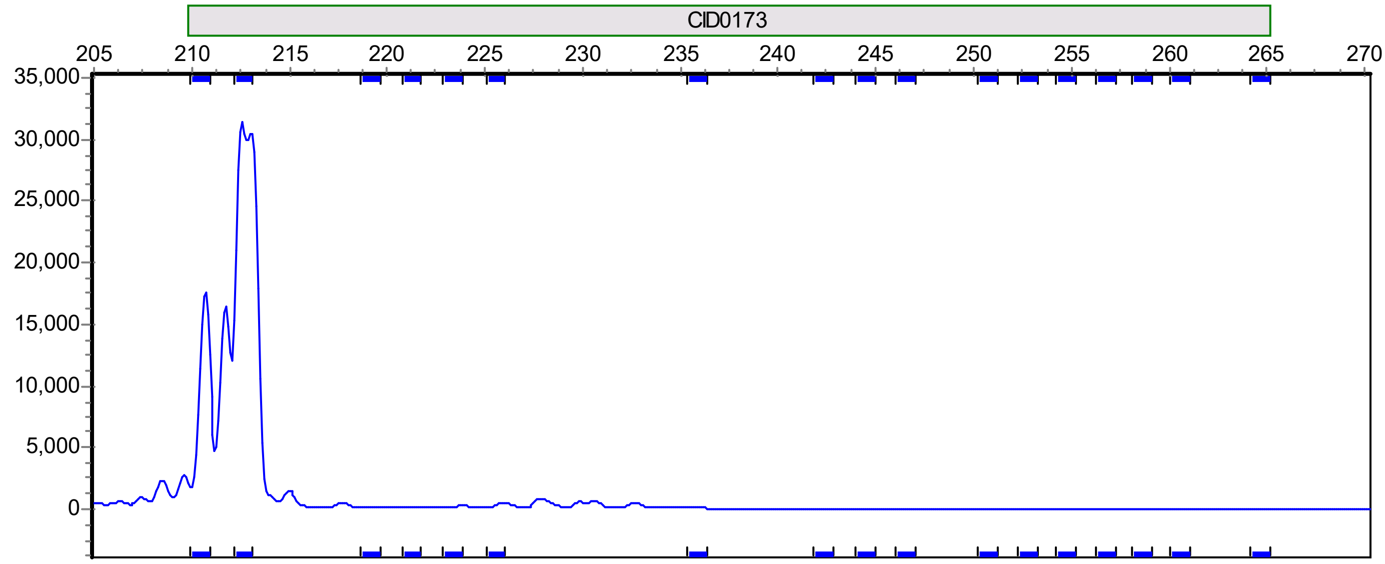

No	Size	Height	Area	Marker	Allele	Difference	Quality	Score	Allele Comments	Sample Comment
1	165.8	19977	143237	EST1573	166	0.00	Pass	500.0	[<Deleted>]	
2	176.6	35056	290495	EST1573	176	0.20	Pass	500.0	[<SAT (Repaired)><Confirmed>]	
3	199.7	18556	137703	CID0173	200	0.20	Pass	500.0	[<Deleted>]	
4	211.8	16379	108826	CID0173	212	0.20	Undetermined	500.0	[<Deleted>]	
5	212.5	30611	360120	CID0173	213	0.20	Check	500.0	[<SAT (Repaired)><Deleted>]	

Sample 124: CID0173_EST1573_KC34_B03.fsa Run date and time: 09/21/2024 - 03:27:32 -> 09/21/2024 - 03:55:03

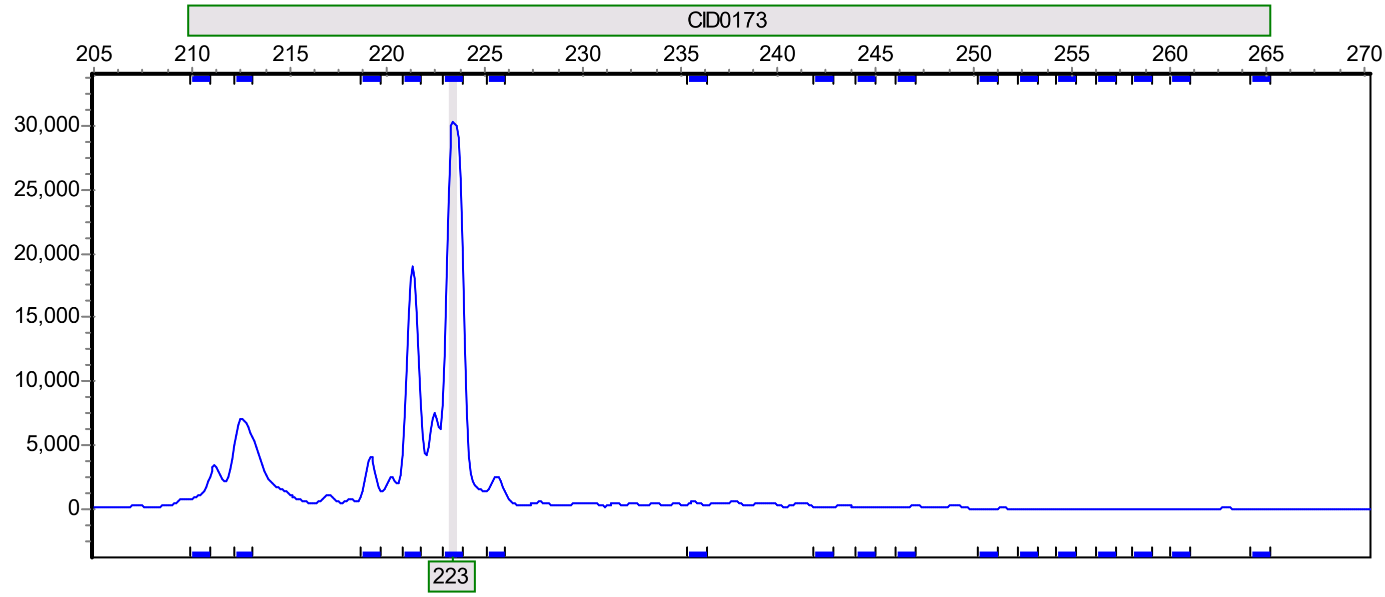

No	Size	Height	Area	Marker	Allele	Difference	Quality	Score	Allele Comments	Sample Comment
1	162.1	19250	133953	EST1573	163	0.80	Pass	500.0	[<Deleted>]	
2	170.7	42810	353399	EST1573	171	0.10	Pass	500.0	[<SAT (Repaired)><Confirmed>]	
3	223.4	30292	306693	CID0173	223	0.00	Pass	500.0	[<Confirmed>]	

Sample 125: CID0173_EST1573_KC35_D03.fsa Run date and time: 09/21/2024 - 03:27:32 -> 09/21/2024 - 03:55:03

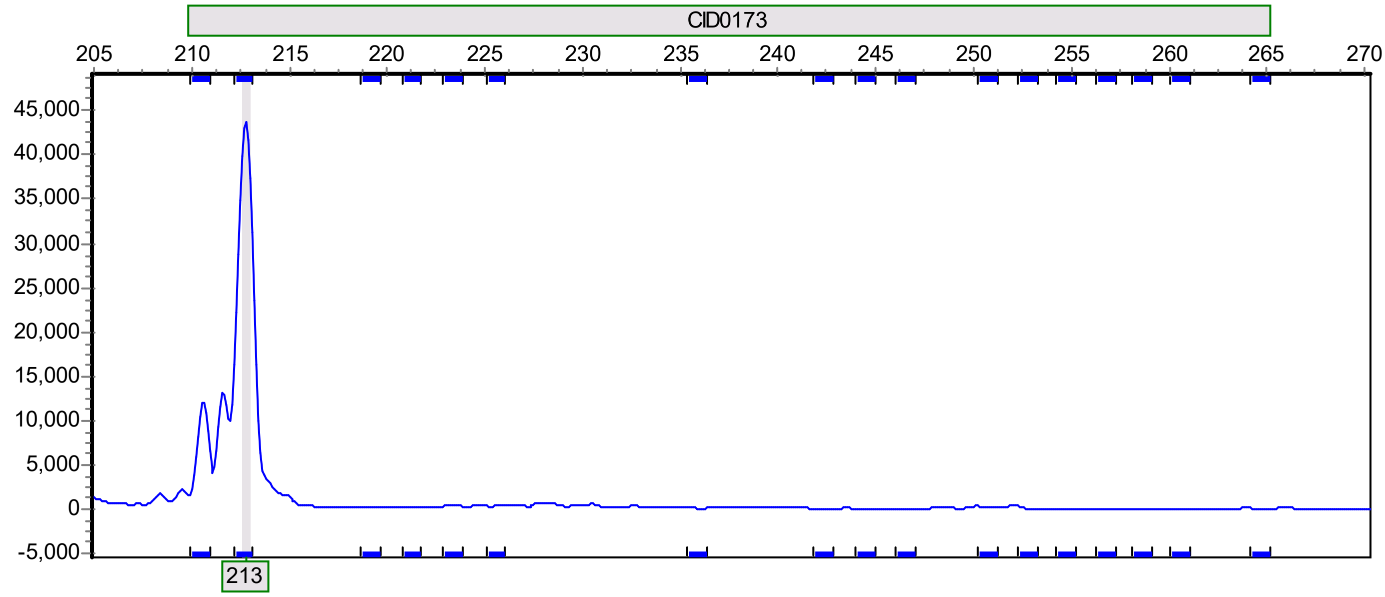

No	Size	Height	Area	Marker	Allele	Difference	Quality	Score	Allele Comments	Sample Comments
1	167.5	18909	132167	EST1573	168	0.00	Pass	500.0	[<Deleted>]	
2	176.4	38531	325755	EST1573	176	0.00	Pass	500.0	[<SAT (Repaired)><Confirmed>]	
3	202.4	20163	176584	CID0173	202	0.00	Pass	500.0	[<Deleted>]	
4	212.8	43712	398828	CID0173	213	0.10	Pass	500.0	[<SAT (Repaired)><Confirmed>]	

Sample 126: CID0173_EST1573_KC36_F03.fsa Run date and time: 09/21/2024 - 03:27:32 -> 09/21/2024 - 03:55:03

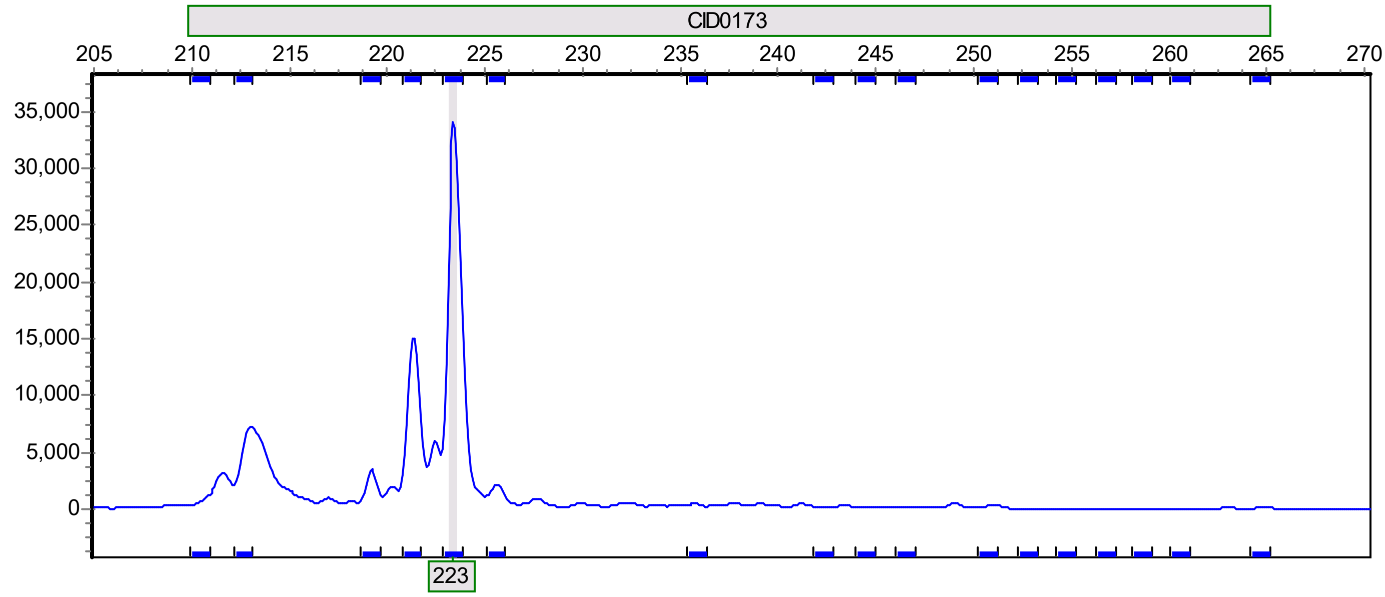

No	Size	Height	Area	Marker	Allele	Difference	Quality	Score	Allele Comments	Sample Comments
1	162.1	17798	125192	EST1573	163	0.80	Pass	500.0	[<Deleted>]	
2	170.6	31021	296562	EST1573	171	0.00	Pass	500.0	[<SAT (Repaired)><Confirmed>]	
3	223.4	34063	281288	CID0173	223	0.00	Pass	500.0	[<SAT (Repaired)><Confirmed>]	

Sample 127: CID0173_EST1573_KC37_H03.fsa Run date and time: 09/21/2024 - 03:27:32 -> 09/21/2024 - 03:55:03

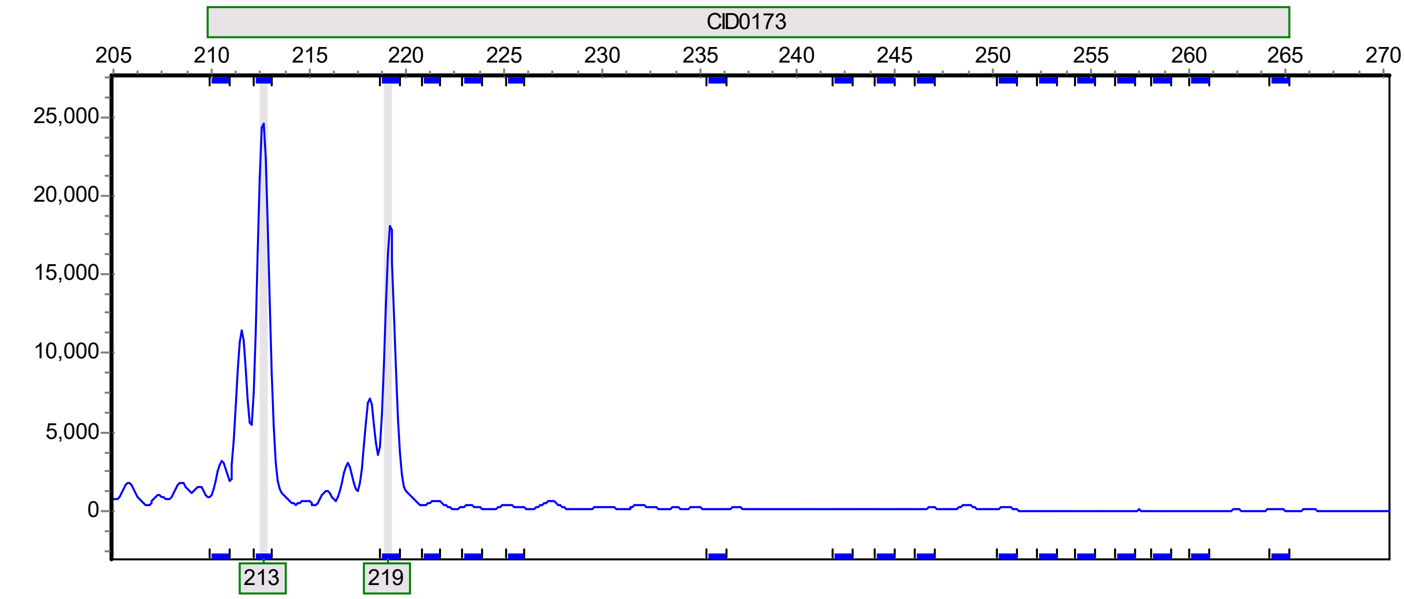

No	Size	Height	Area	Marker	Allele	Difference	Quality	Score	Allele Comments	Sample Comments
1	162.9	32242	213304	EST1573	163	0.00	Pass	500.0	[<SAT (Repaired)><Confirmed>]	
2	164.9	29298	179045	EST1573	165	0.10	Pass	500.0	[<Confirmed>]	
3	211.6	11394	77623	CID0173	212	0.00	Undetermined	500.0	[<Deleted>]	
4	212.7	24589	170807	CID0173	213	0.00	Pass	500.0	[<Confirmed>]	
5	219.1	18133	125367	CID0173	219	0.10	Pass	500.0	[<Confirmed>]	

Sample 128: CID0173_EST1573_KC38_J03.fsa Run date and time: 09/21/2024 - 03:27:32 -> 09/21/2024 - 03:55:03

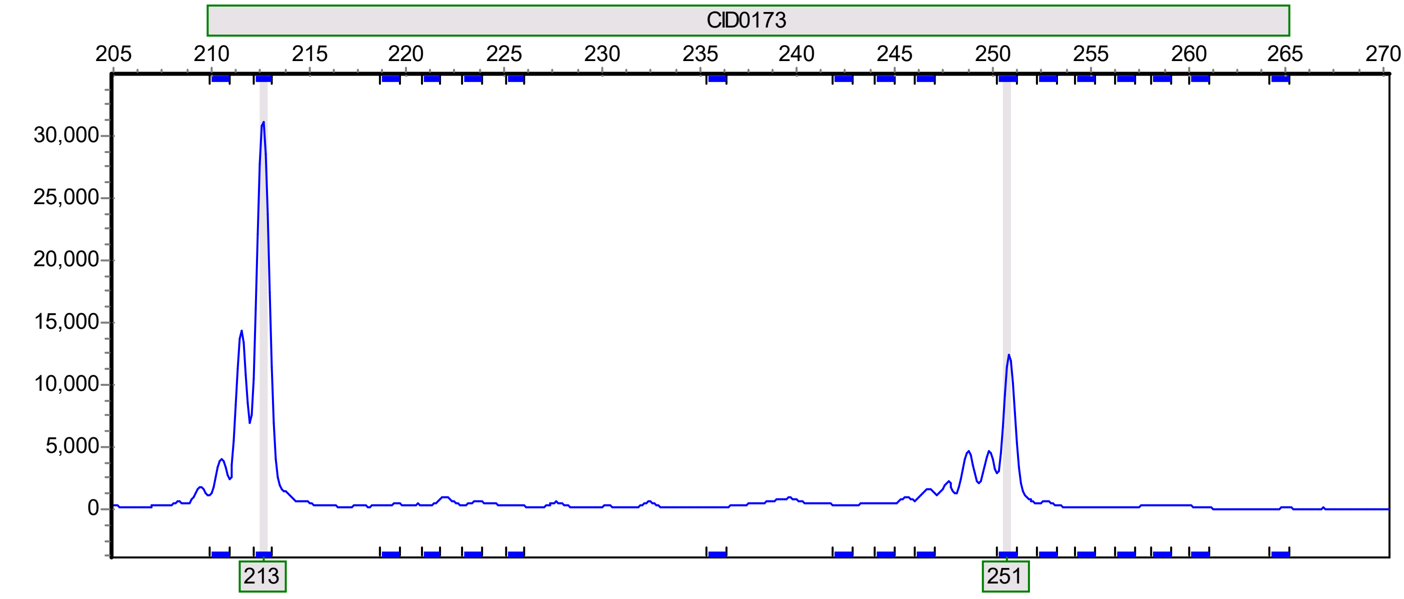

No	Size	Height	Area	Marker	Allele	Difference	Quality	Score	Allele Comments	Sample Comments
1	162.9	30465	221933	EST1573	163	0.00	Pass	500.0	[<Confirmed>]	
2	174.4	22368	141554	EST1573	174	0.00	Pass	500.0	[<Confirmed>]	
3	211.6	14247	96486	CID0173	212	0.00	Pass	500.0	[<Deleted>]	
4	212.7	31070	223275	CID0173	213	0.00	Pass	500.0	[<Confirmed>]	
5	250.8	12405	82692	CID0173	251	0.00	Pass	500.0	[<Confirmed>]	

Sample 129: CID0173_EST1573_KC39_L03.fsa Run date and time: 09/21/2024 - 03:27:32 -> 09/21/2024 - 03:55:03

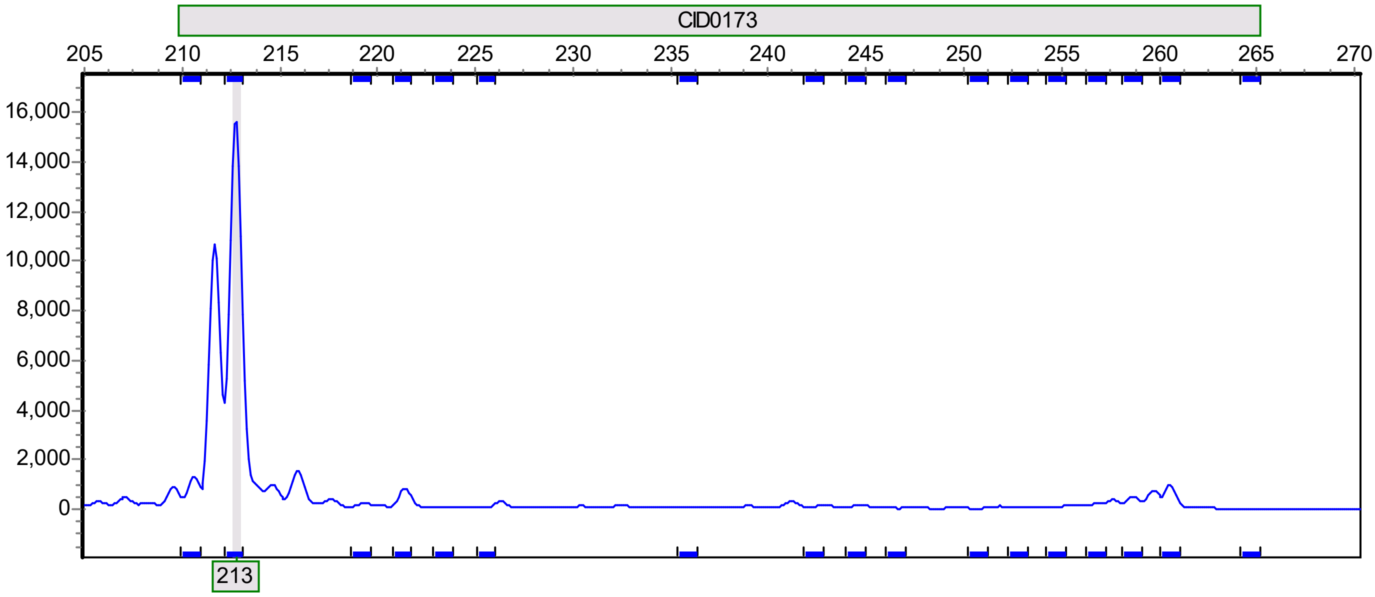

No	Size	Height	Area	Marker	Allele	Difference	Quality	Score	Allele Comments	Sample Comments
1	163.1	30145	183638	EST1573	163	0.20	Pass	500.0	[<Confirmed>]	
2	174.5	15034	93088	EST1573	174	0.10	Pass	500.0	[<Confirmed>]	
3	211.7	10688	68271	CID0173	212	0.10	Pass	500.0	[<Deleted>]	
4	212.8	15627	106213	CID0173	213	0.10	Pass	500.0	[<Confirmed>]	

Sample 130: CID0173_EST1573_KC3_C19.fsa Run date and time: 09/21/2024 - 03:00:19 -> 09/21/2024 - 03:27:31

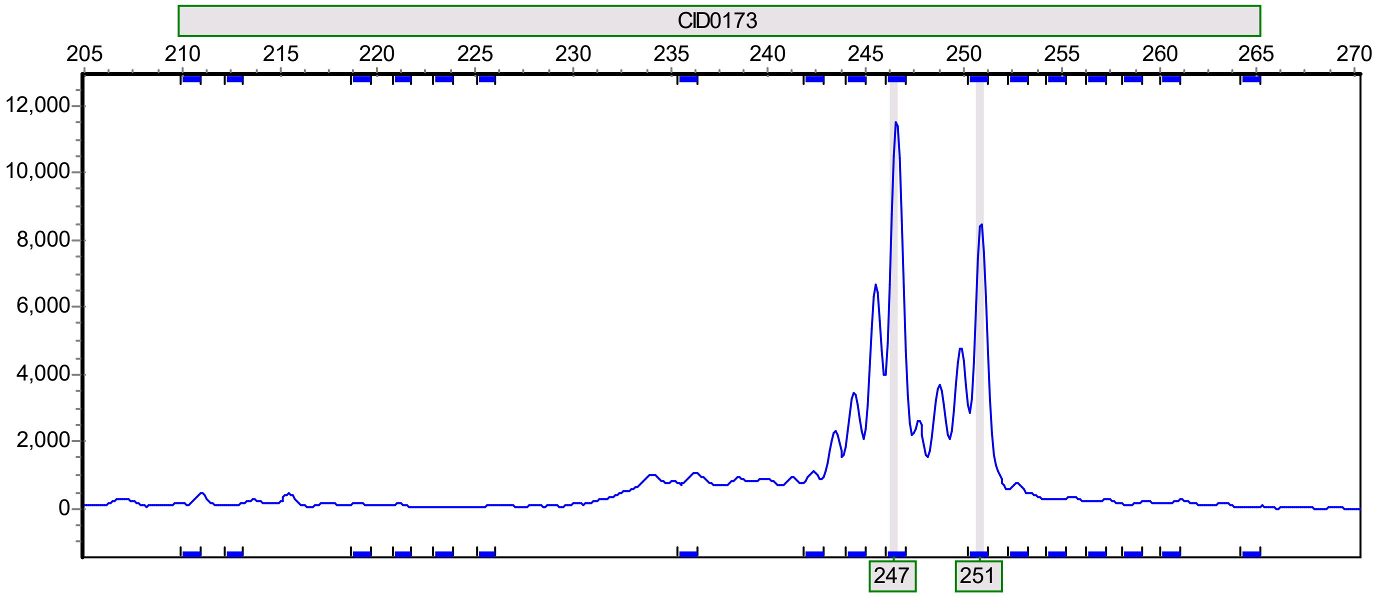

No	Size	Height	Area	Marker	Allele	Difference	Quality	Score	Allele Comments	Sample Comments
1	174.3	21560	141791	EST1573	174	0.10	Pass	500.0	[<Confirmed>]	
2	181.9	10944	71445	EST1573	182	0.10	Pass	500.0	[<Confirmed>]	
3	246.5	11495	86956	CID0173	247	0.10	Pass	500.0	[<Confirmed>]	
4	249.8	4772	33897	CID0173	250	0.00	Undetermined	500.0	[<Deleted>]	
5	250.9	8471	62500	CID0173	251	0.10	Pass	500.0	[<Confirmed>]	

Sample 131: CID0173_EST1573_KC40_N03.fsa Run date and time: 09/21/2024 - 03:27:32 -> 09/21/2024 - 03:55:03

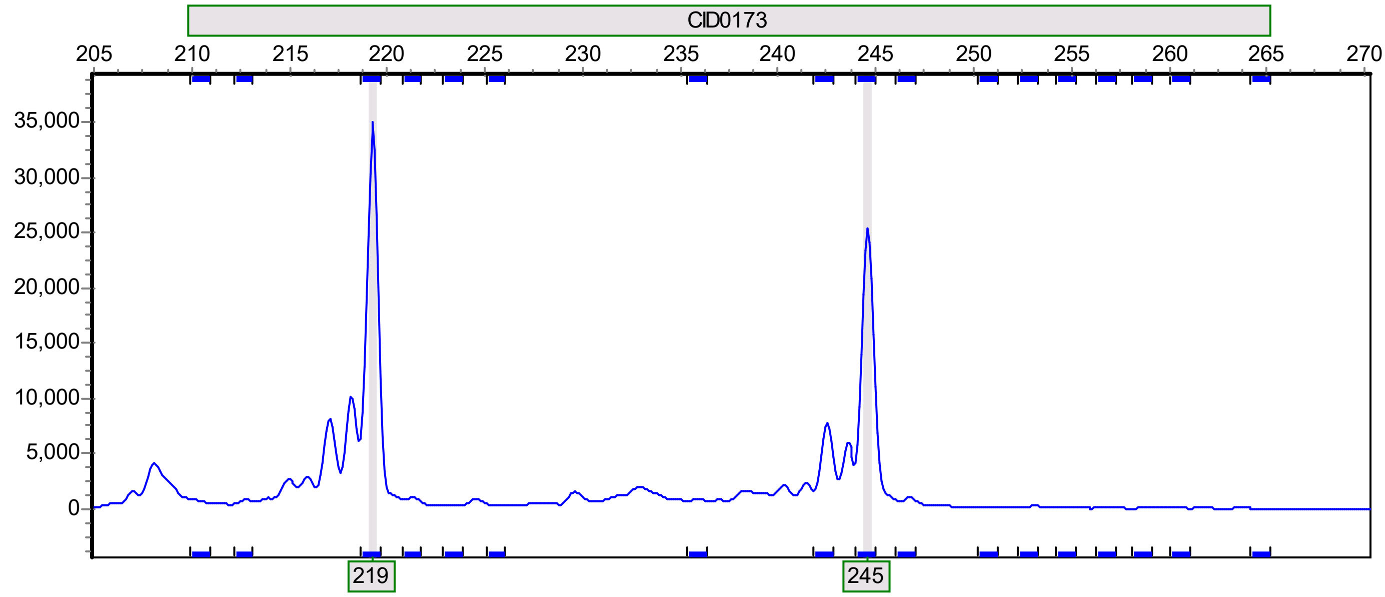

No	Size	Height	Area	Marker	Allele	Difference	Quality	Score	Allele Comments	Sample Comments
1	162.8	30883	224993	EST1573	163	0.10	Pass	500.0	[<SAT (Repaired)><Confirmed>]	
2	174.4	29641	185567	EST1573	174	0.00	Pass	500.0	[<Confirmed>]	
3	219.3	35029	258264	CID0173	219	0.10	Pass	500.0	[<SAT (Repaired)><Confirmed>]	
4	244.6	25357	175933	CID0173	245	0.10	Pass	500.0	[<Confirmed>]	

Sample 132: CID0173_EST1573_KC41_P03.fsa Run date and time: 09/21/2024 - 03:27:32 -> 09/21/2024 - 03:55:03

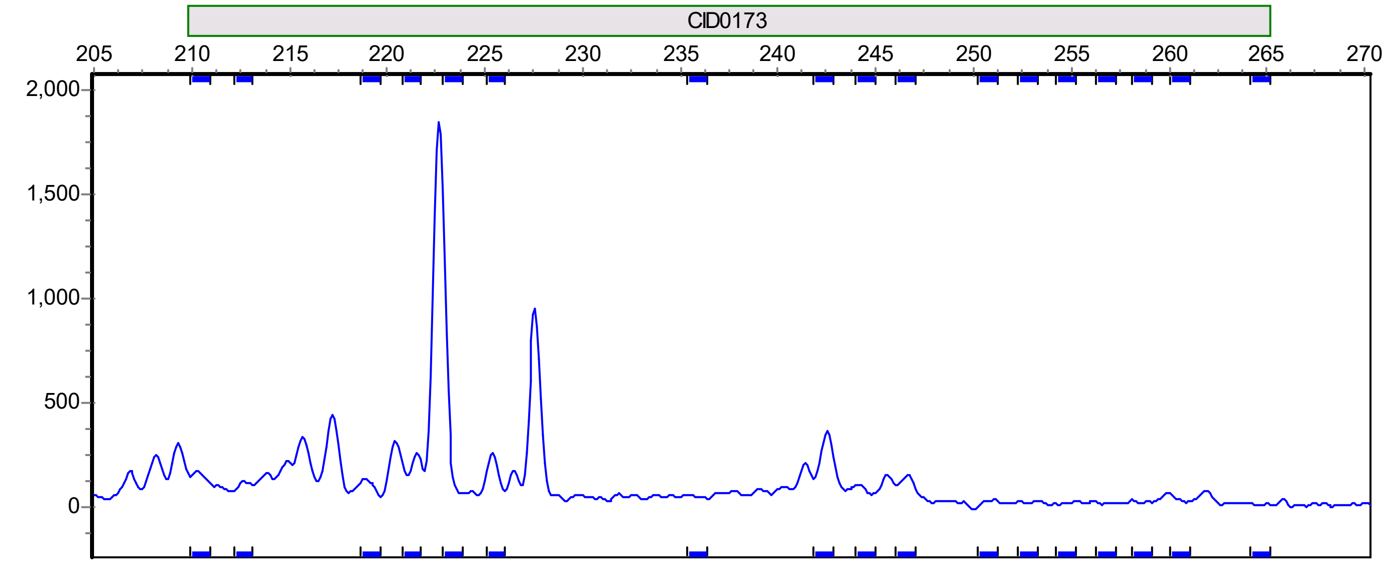

No	Size	Height	Area	Marker	Allele	Difference	Quality	Score	Allele Comments	Sample Comments
1	163.1	33497	208207	EST1573	163	0.20	Pass	500.0	[<SAT (Repaired)><Confirmed>]	
2	174.5	6214	47332	EST1573	174	0.10	Pass	500.0	[<Confirmed><Inserted>]	
3	222.6	1847	12790	CID0173	222	0.20	Pass	302.4	[<Deleted>]	

Allele Report

Sample 133: CID0173_EST1573_KC42_B05.fsa Run date and time: 09/21/2024 - 03:27:32 -> 09/21/2024 - 03:55:03

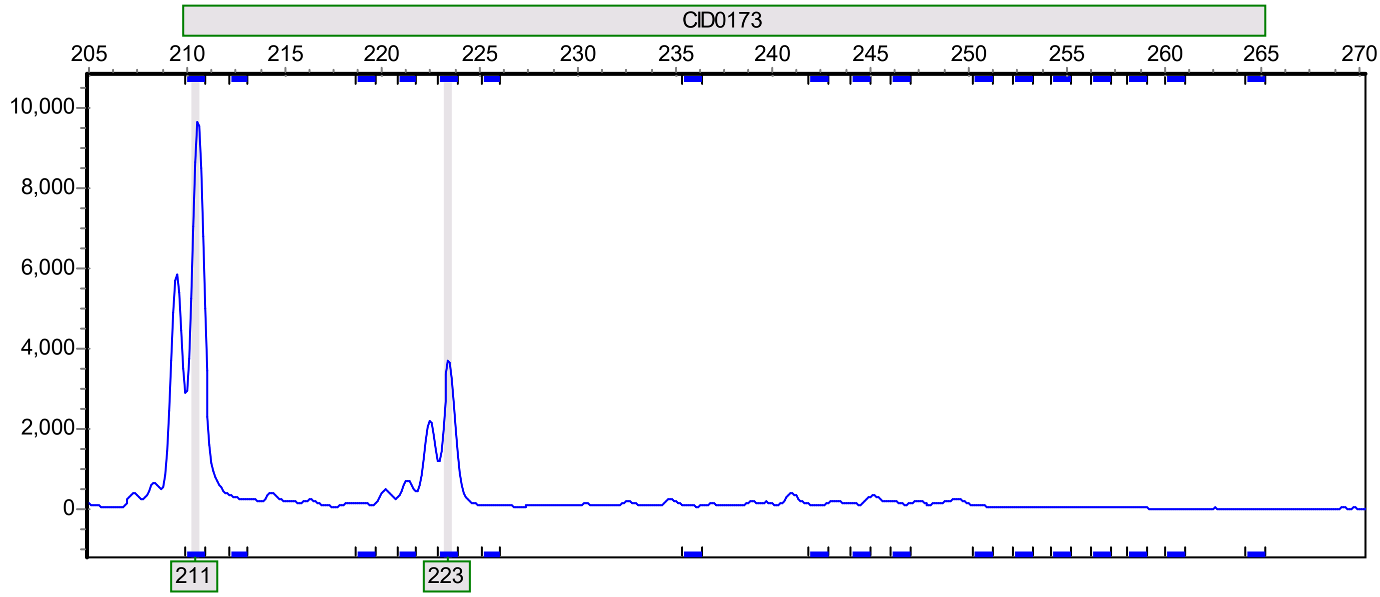

No	Size	Height	Area	Marker	Allele	Difference	Quality	Score	Allele Comments	Sample Comments
1	163.0	30485	189290	EST1573	163	0.10	Pass	500.0	[<Confirmed>]	
2	172.5	13976	89993	EST1573	173	0.00	Pass	500.0	[<Confirmed>]	
3	209.5	5847	40046	CID0173	210	0.00	Pass	500.0	[<Deleted>]	
4	210.5	9635	69553	CID0173	211	0.00	Pass	500.0	[<Confirmed>]	
5	223.4	3668	26900	CID0173	223	0.00	Pass	500.0	[<Confirmed>]	

Sample 134: CID0173_EST1573_KC43_D05.fsa Run date and time: 09/21/2024 - 03:27:32 -> 09/21/2024 - 03:55:03

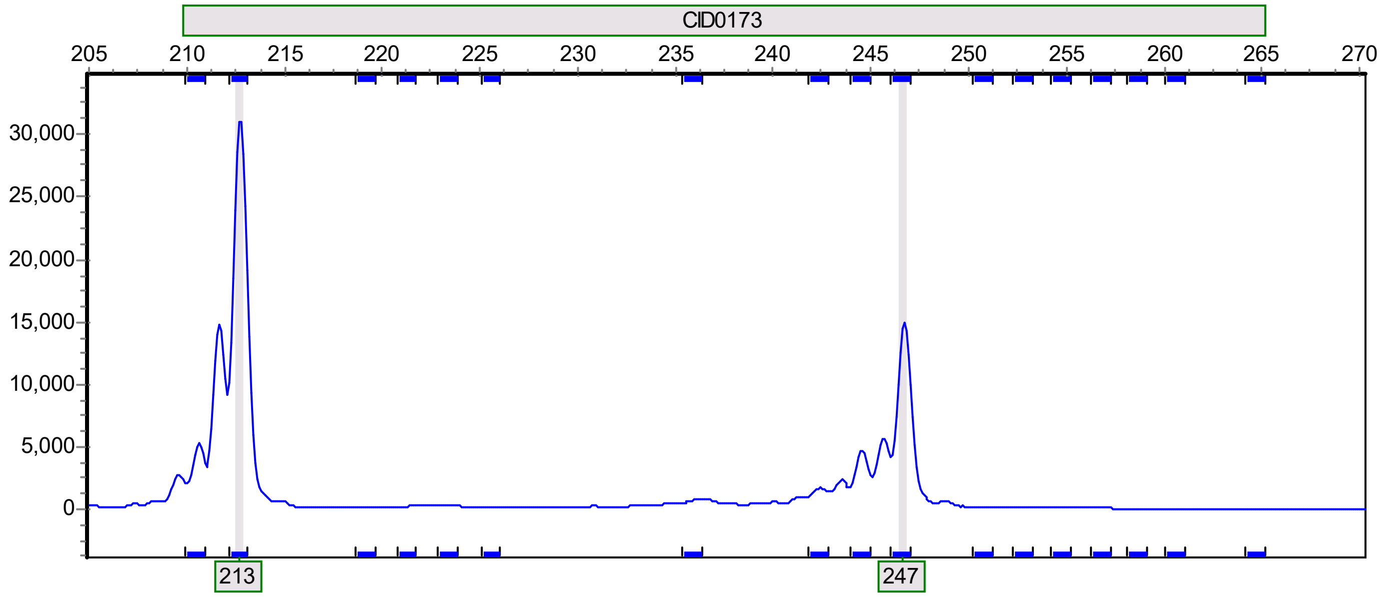

No	Size	Height	Area	Marker	Allele	Difference	Quality	Score	Allele Comments	Sample Comments
1	163.0	33308	252557	EST1573	163	0.10	Pass	500.0	[<SAT (Repaired)><Confirmed>]	
2	174.5	30394	203222	EST1573	174	0.10	Pass	500.0	[<Confirmed>]	
3	211.7	14788	106956	CID0173	212	0.10	Undetermined	500.0	[<Deleted>]	
4	212.7	31020	243110	CID0173	213	0.00	Pass	500.0	[<SAT (Repaired)><Confirmed>]	
5	246.7	14957	116580	CID0173	247	0.10	Pass	500.0	[<Confirmed>]	

Sample 135: CID0173_EST1573_KC44_F05.fsa Run date and time: 09/21/2024 - 03:27:32 -> 09/21/2024 - 03:55:03

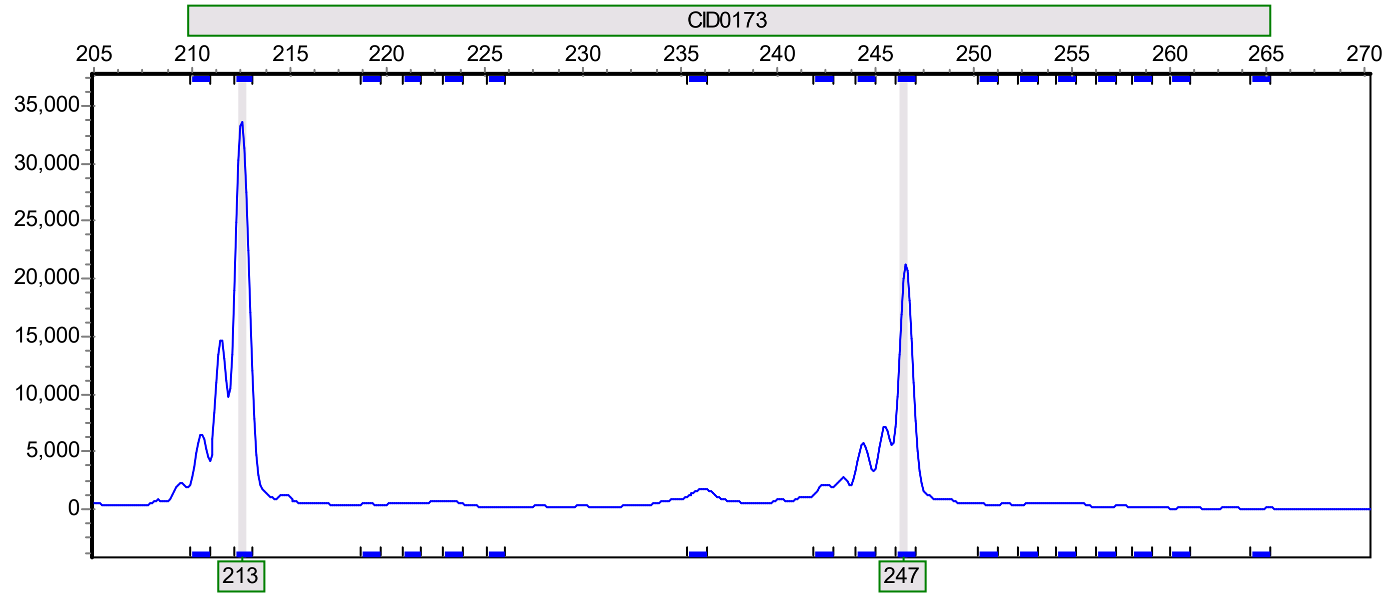

No	Size	Height	Area	Marker	Allele	Difference	Quality	Score	Allele Comments	Sample Comments
1	176.3	35620	288765	EST1573	176	0.10	Pass	500.0	[<SAT (Repaired)><Confirmed>]	
2	181.8	14686	101855	EST1573	182	0.20	Pass	500.0	[<Confirmed>]	
3	211.6	14675	106657	CID0173	212	0.00	Undetermined	500.0	[<Deleted>]	
4	212.6	33589	270514	CID0173	213	0.10	Pass	500.0	[<SAT (Repaired)><Confirmed>]	
5	246.5	21180	164653	CID0173	247	0.10	Pass	500.0	[<Confirmed>]	

Sample 136: CID0173_EST1573_KC45_H05.fsa Run date and time: 09/21/2024 - 03:27:32 -> 09/21/2024 - 03:55:03

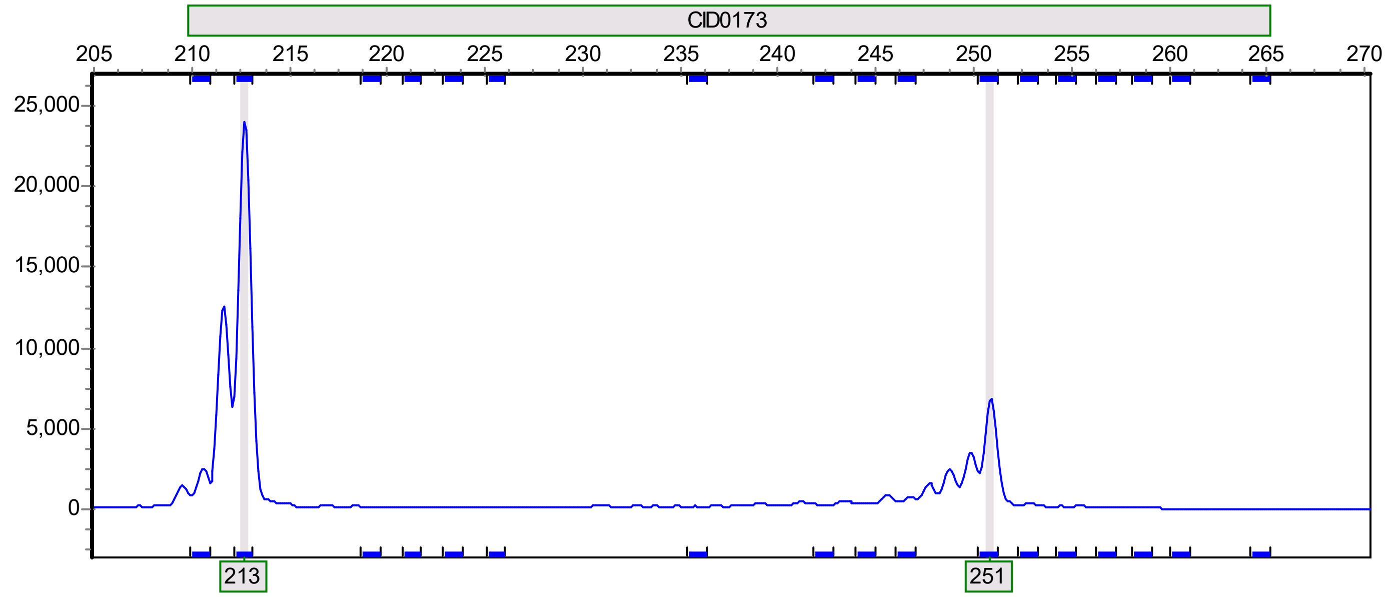

No	Size	Height	Area	Marker	Allele	Difference	Quality	Score	Allele Comments	Sample Comments
1	174.5	26918	175148	EST1573	174	0.10	Pass	500.0	[<Confirmed>]	
2	178.3	20801	135762	EST1573	178	0.10	Pass	500.0	[<Confirmed>]	
3	211.7	12544	89514	CID0173	212	0.10	Pass	500.0	[<Deleted>]	
4	212.7	24033	166502	CID0173	213	0.00	Pass	500.0	[<Confirmed>]	
5	250.9	6815	48697	CID0173	251	0.10	Pass	500.0	[<Confirmed><Inserted>]	

Allele Report

Sample 137: CID0173_EST1573_KC46_J05.fsa Run date and time: 09/21/2024 - 03:27:32 -> 09/21/2024 - 03:55:03

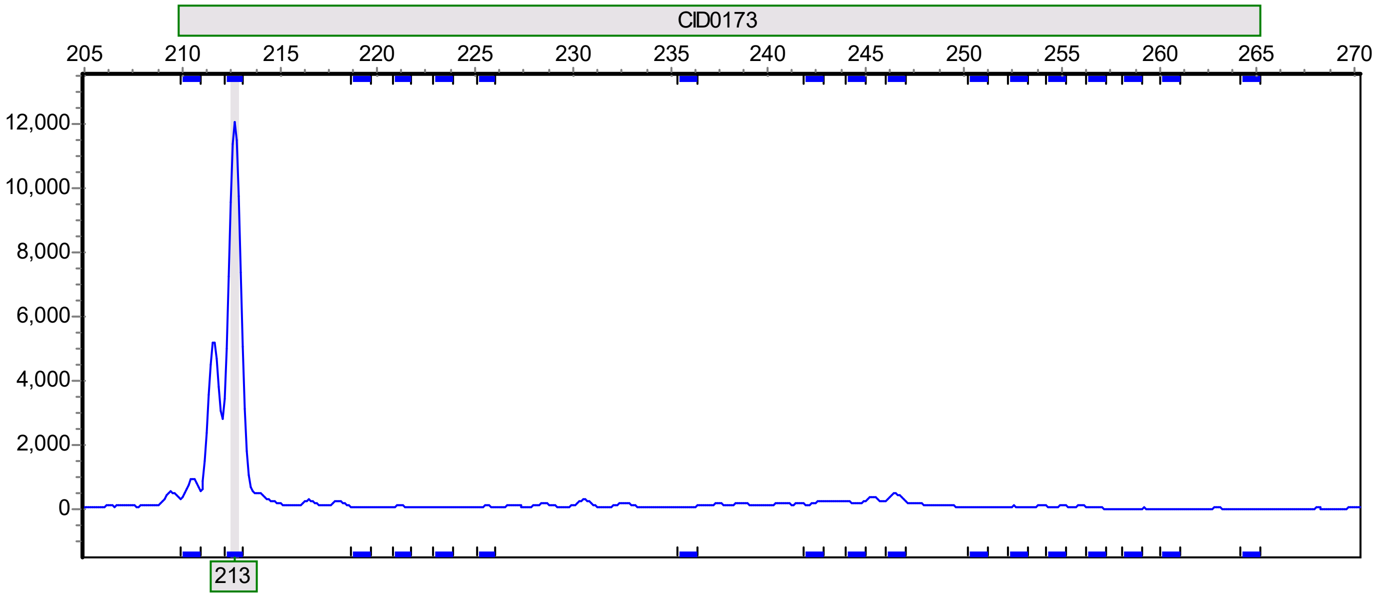

No	Size	Height	Area	Marker	Allele	Difference	Quality	Score	Allele Comments	Sample Comments
1	174.3	12334	81090	EST1573	174	0.10	Pass	500.0	[<Confirmed>]	
2	182.0	3313	21791	EST1573	182	0.00	Pass	500.0	[<Confirmed><Inserted>]	
3	211.7	5200	33968	CID0173	212	0.10	Pass	500.0	[<Deleted>]	
4	212.7	12057	85088	CID0173	213	0.00	Pass	500.0	[<Confirmed>]	

Sample 138: CID0173_EST1573_KC47_L05.fsa Run date and time: 09/21/2024 - 03:27:32 -> 09/21/2024 - 03:55:03

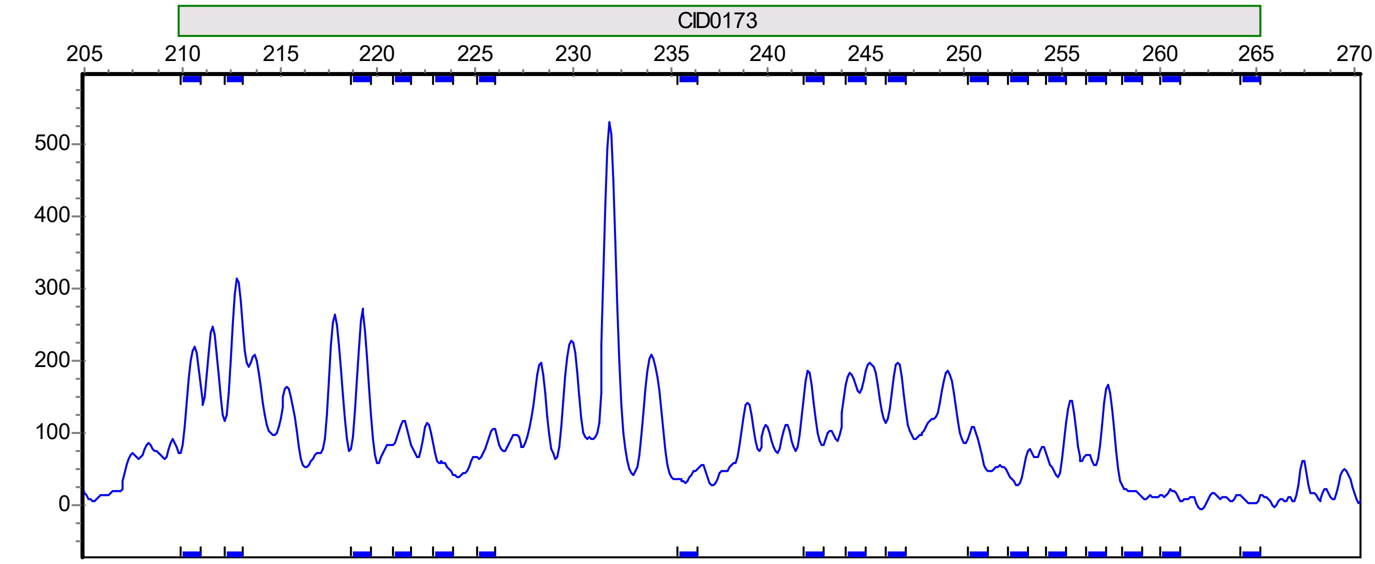

No	Size	Height	Area	Marker	Allele	Difference	Quality	Score	Allele Comments	Sample Comments
1	168.6	728	6491	EST1573	169	0.10	Pass	40.4	[<Deleted>]	
2	172.6	738	6973	EST1573	173	0.10	Undetermined	33.1	[<Deleted>]	
3	174.4	598	4645	EST1573	174	0.00	Undetermined	26.5	[<Deleted>]	
4	176.4	499	4110	EST1573	176	0.00	Undetermined	11.9	[<Deleted>]	
5	177.7	755	6651	EST1573	178	0.50	Pass	48.1	[<Deleted>]	
6	184.0	329	2659	EST1573	184	0.00	Undetermined	13.4	[<Deleted>]	
7	212.8	313	2447	CID0173	213	0.10	Pass	9.7	[<Deleted>]	

Sample 139: CID0173_EST1573_KC48_N05.fsa Run date and time: 09/21/2024 - 03:27:32 -> 09/21/2024 - 03:55:03

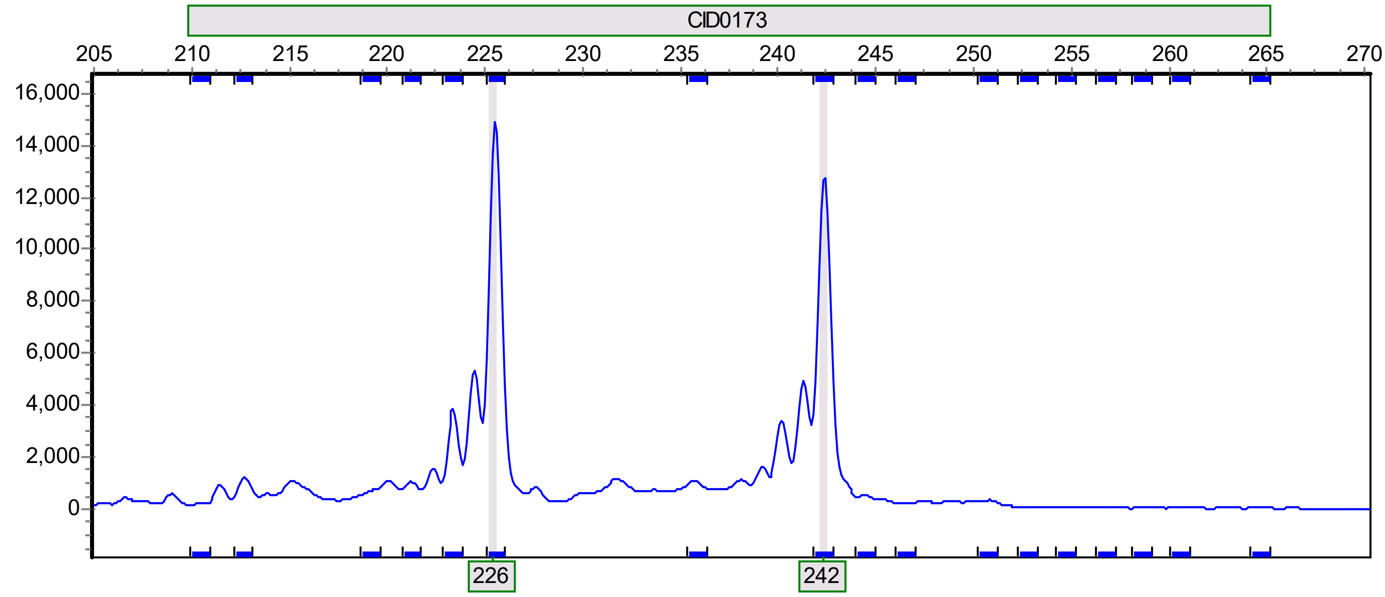

No	Size	Height	Area	Marker	Allele	Difference	Quality	Score	Allele Comments	Sample Comments
1	174.4	20312	130194	EST1573	174	0.00	Pass	500.0	[<Confirmed>]	
2	178.3	21609	141246	EST1573	178	0.10	Pass	500.0	[<Confirmed>]	
3	225.5	14897	109026	CID0173	226	0.10	Pass	500.0	[<Confirmed>]	
4	242.4	12699	97933	CID0173	242	0.00	Pass	500.0	[<Confirmed>]	

Sample 140: CID0173_EST1573_KC49_P05.fsa Run date and time: 09/21/2024 - 03:27:32 -> 09/21/2024 - 03:55:03

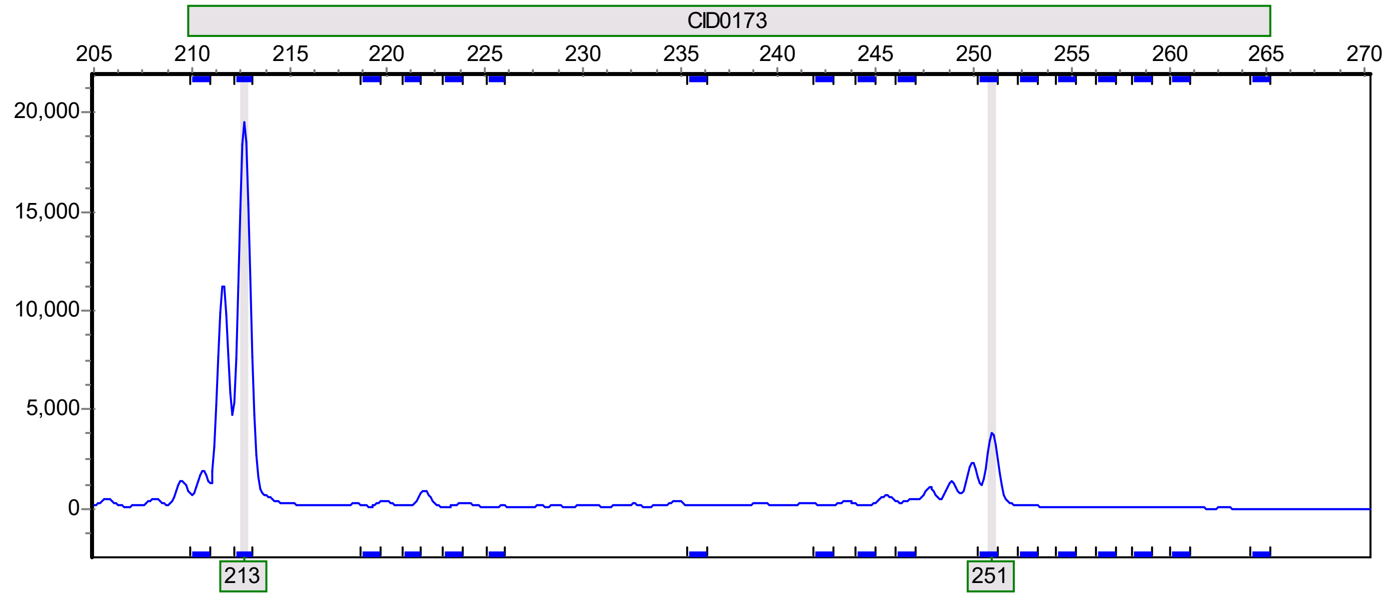

No	Size	Height	Area	Marker	Allele	Difference	Quality	Score	Allele Comments	Sample Comments
1	172.6	30275	187921	EST1573	173	0.10	Pass	500.0	[<Confirmed>]	
2	174.4	15136	96079	EST1573	174	0.00	Pass	500.0	[<Confirmed>]	
3	211.6	11248	77199	CID0173	212	0.00	Pass	500.0	[<Deleted>]	
4	212.7	19508	134538	CID0173	213	0.00	Pass	500.0	[<Confirmed>]	
5	251.0	3731	26292	CID0173	251	0.20	Pass	500.0	[<Confirmed><Inserted>]	

Allele Report

Sample 141: CID0173_EST1573_KC4_E19.fsa Run date and time: 09/21/2024 - 03:00:19 -> 09/21/2024 - 03:27:31

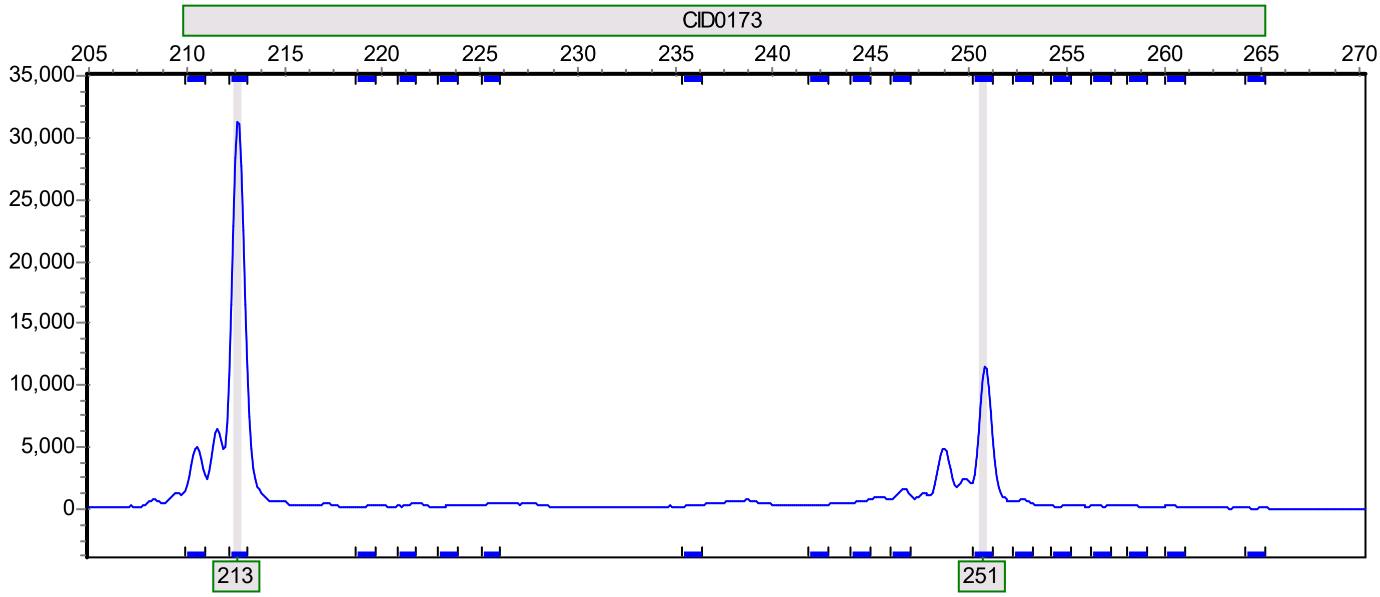

No	Size	Height	Area	Marker	Allele	Difference	Quality	Score	Allele Comments	Sample Comments
1	172.4	27105	189401	EST1573	173	0.10	Pass	500.0	[<Confirmed>]	
2	212.6	31280	225201	CID0173	213	0.10	Pass	500.0	[<Confirmed>]	
3	250.8	11575	81315	CID0173	251	0.00	Pass	500.0	[<Confirmed>]	

Sample 142: CID0173_EST1573_KC50_B07.fsa Run date and time: 09/21/2024 - 03:27:32 -> 09/21/2024 - 03:55:03

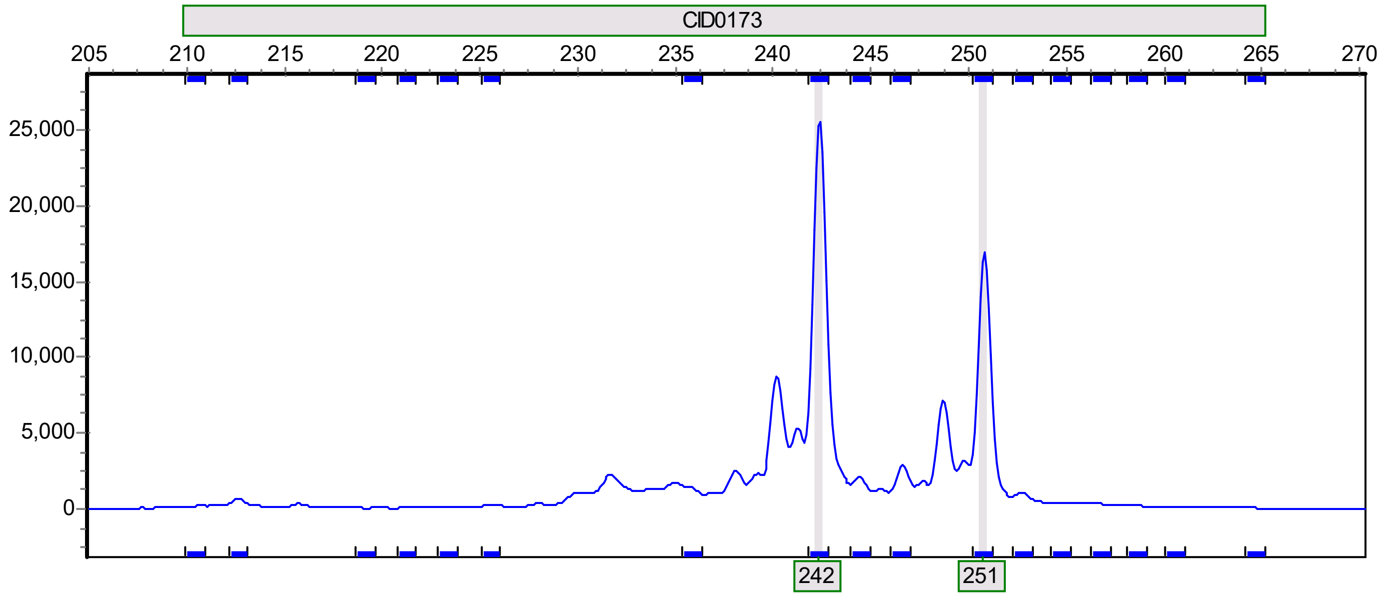

No	Size	Height	Area	Marker	Allele	Difference	Quality	Score	Allele Comments	Sample Comments
1	170.6	33476	241691	EST1573	171	0.00	Pass	500.0	[<SAT (Repaired)><Confirmed>]	
2	174.4	31114	218871	EST1573	174	0.00	Pass	500.0	[<Confirmed>]	
3	242.4	25538	198365	CID0173	242	0.00	Pass	500.0	[<Confirmed>]	
4	250.8	16882	121527	CID0173	251	0.00	Pass	500.0	[<Confirmed>]	

Sample 143: CID0173_EST1573_KC51_D07.fsa Run date and time: 09/21/2024 - 03:27:32 -> 09/21/2024 - 03:55:03

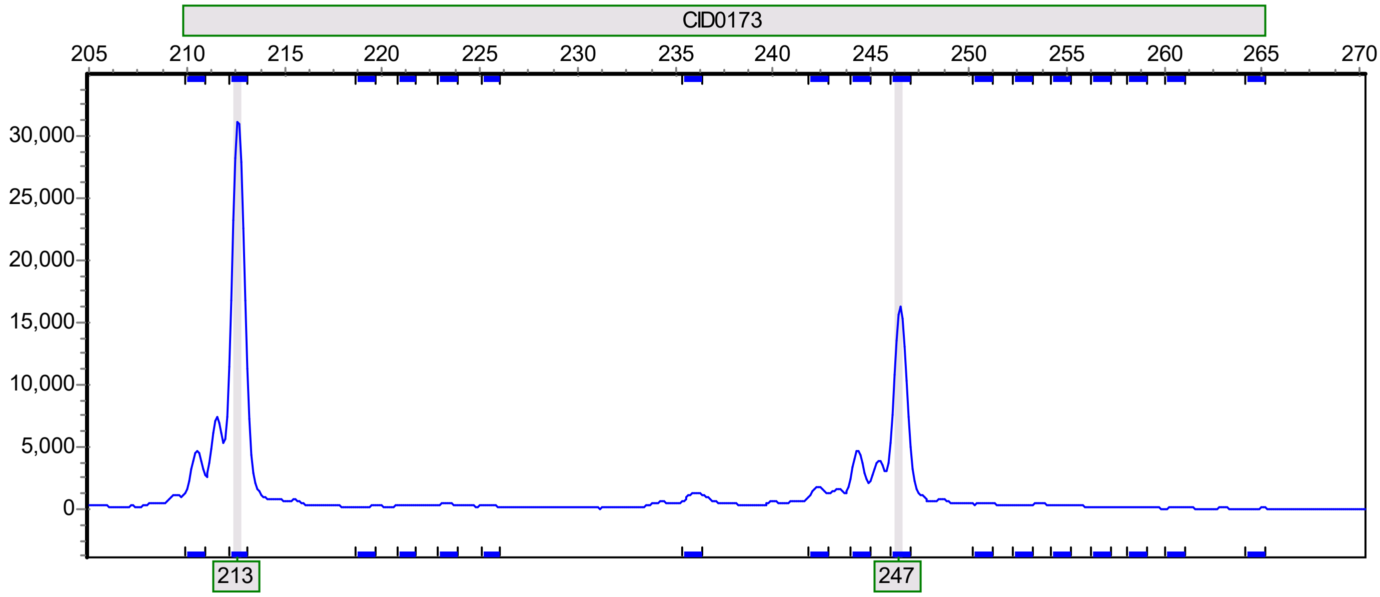

No	Size	Height	Area	Marker	Allele	Difference	Quality	Score	Allele Comments	Sample Comments
1	174.2	24843	175332	EST1573	174	0.20	Pass	500.0	[<Confirmed>]	
2	181.8	10643	70558	EST1573	182	0.20	Pass	500.0	[<Confirmed>]	
3	212.6	31099	233889	CID0173	213	0.10	Pass	500.0	[<Confirmed>]	
4	246.5	16187	121052	CID0173	247	0.10	Pass	500.0	[<Confirmed>]	

Sample 144: CID0173_EST1573_KC52_F07.fsa Run date and time: 09/21/2024 - 03:27:32 -> 09/21/2024 - 03:55:03

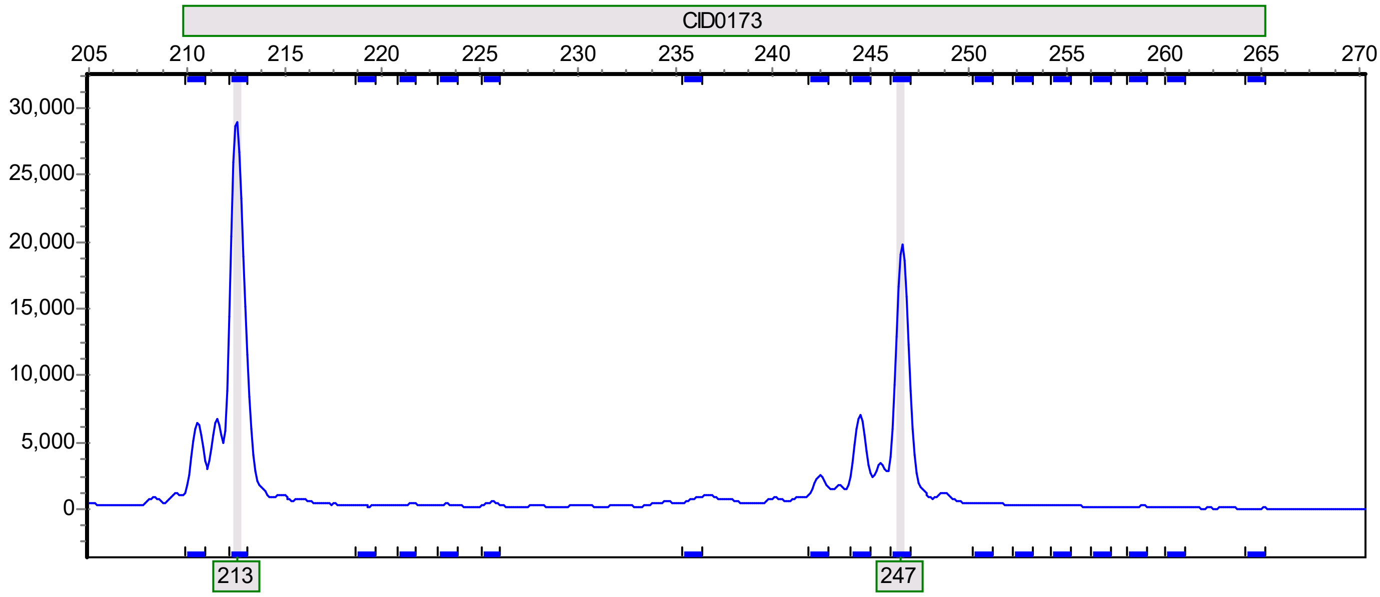

No	Size	Height	Area	Marker	Allele	Difference	Quality	Score	Allele Comments	Sample Comments
1	174.4	29983	207036	EST1573	174	0.00	Pass	500.0	[<Confirmed>]	
2	181.9	12701	85508	EST1573	182	0.10	Pass	500.0	[<Confirmed>]	
3	212.6	28949	229645	CID0173	213	0.10	Pass	500.0	[<SAT (Repaired)><Confirmed>]	
4	246.6	19833	146653	CID0173	247	0.00	Pass	500.0	[<Confirmed>]	

Allele Report

Sample 145: CID0173_EST1573_KC53_H07.fsa Run date and time: 09/21/2024 - 03:27:32 -> 09/21/2024 - 03:55:03

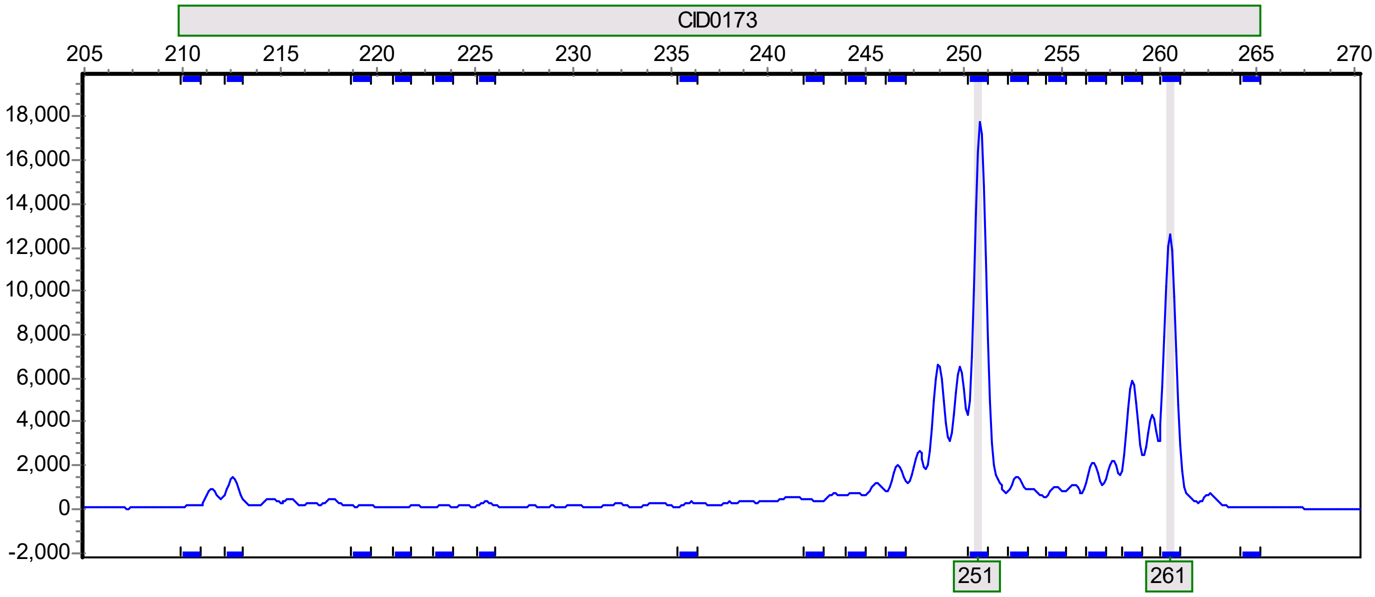

No	Size	Height	Area	Marker	Allele	Difference	Quality	Score	Allele Comments	Sample Comments
1	172.4	38455	270682	EST1573	173	0.10	Pass	500.0	[<SAT (Repaired)><Confirmed>]	
2	178.3	31739	241580	EST1573	178	0.10	Pass	500.0	[<Confirmed>]	
3	249.8	6498	42029	CID0173	250	0.00	Undetermined	500.0	[<Deleted>]	
4	250.8	17764	126309	CID0173	251	0.00	Pass	500.0	[<Confirmed>]	
5	260.6	12627	88201	CID0173	261	0.00	Pass	500.0	[<Confirmed>]	

Sample 146: CID0173_EST1573_KC54_J07.fsa Run date and time: 09/21/2024 - 03:27:32 -> 09/21/2024 - 03:55:03

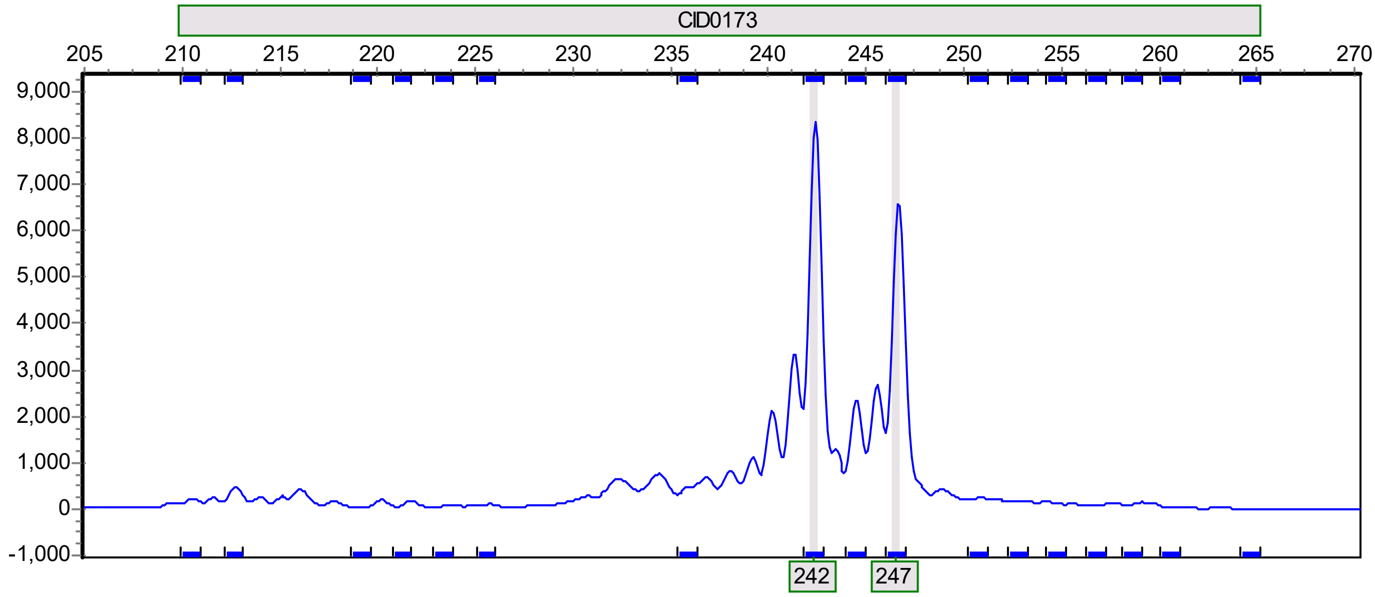

No	Size	Height	Area	Marker	Allele	Difference	Quality	Score	Allele Comments	Sample Comments
1	174.3	26500	162490	EST1573	174	0.10	Pass	500.0	[<Confirmed>]	
2	242.4	8340	61669	CID0173	242	0.00	Pass	500.0	[<Confirmed>]	
3	246.6	6562	48386	CID0173	247	0.00	Pass	500.0	[<Confirmed>]	

SoftGenetics | **Allele Report** | 2024/9/27 18:12:41

Sample 149: CID0173_EST1573_KC57_P07.fsa Run date and time: 09/21/2024 - 03:27:32 -> 09/21/2024 - 03:55:03

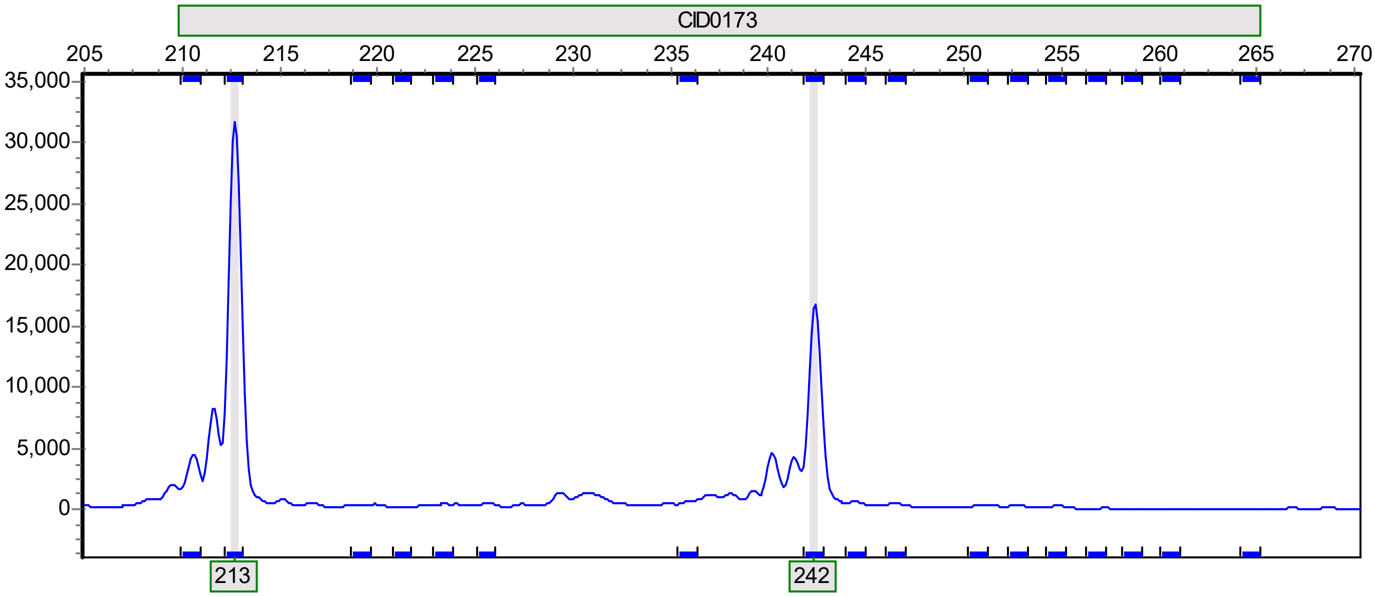

No	Size	Height	Area	Marker	Allele	Difference	Quality	Score	Allele Comments	Sample Comments
1	170.6	31474	215303	EST1573	171	0.00	Pass	500.0	[<SAT (Repaired)><Confirmed>]	
2	174.4	23922	156589	EST1573	174	0.00	Pass	500.0	[<Confirmed>]	
3	212.7	31722	230641	CID0173	213	0.00	Pass	500.0	[<Confirmed>]	
4	242.4	16823	120241	CID0173	242	0.00	Pass	500.0	[<Confirmed>]	

Sample 150: CID0173_EST1573_KC58_B09.fsa Run date and time: 09/21/2024 - 03:27:32 -> 09/21/2024 - 03:55:03

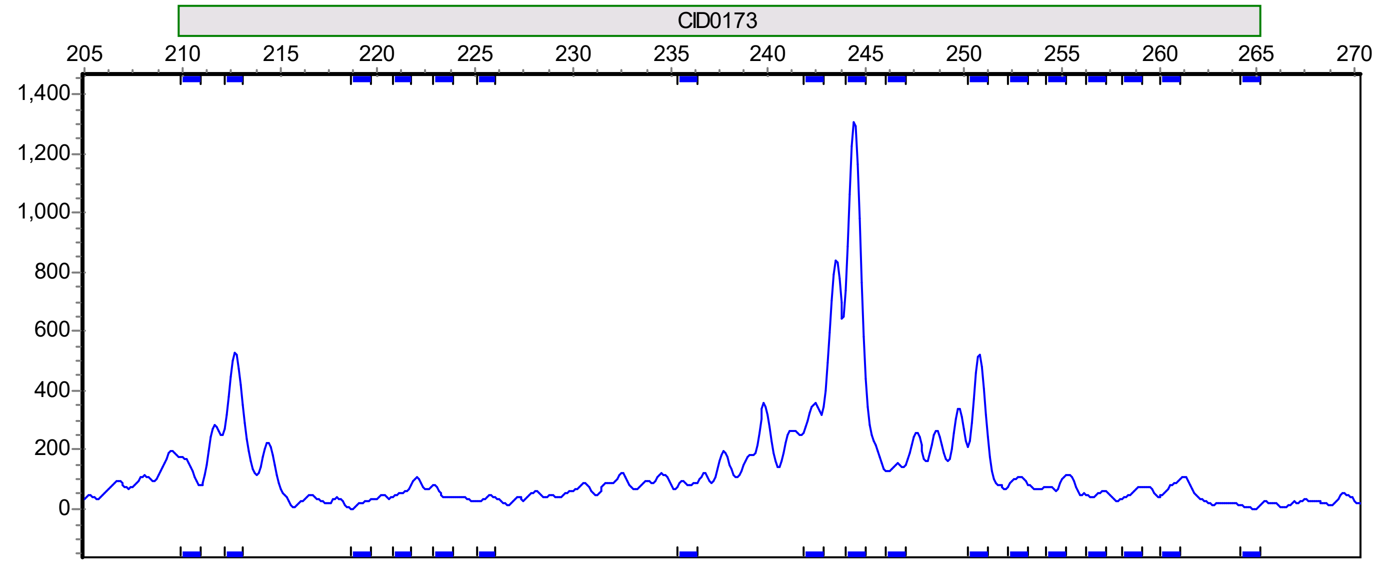

No	Size	Height	Area	Marker	Allele	Difference	Quality	Score	Allele Comments	Sample Comments
1	172.5	21631	145672	EST1573	173	0.00	Pass	500.0	[<Confirmed>]	
2	174.4	9863	66316	EST1573	174	0.00	Pass	500.0	[<Confirmed>]	
3	212.7	526	4897	CID0173	213	0.00	Undetermined	19.8	[<Deleted>]	
4	244.4	1308	10416	CID0173	245	0.10	Pass	108.2	[<Deleted>]	
5	250.8	523	3994	CID0173	251	0.00	Pass	32.3	[<Deleted>]	

Sample 151: CID0173_EST1573_KC59_D09.fsa Run date and time: 09/21/2024 - 03:27:32 -> 09/21/2024 - 03:55:03

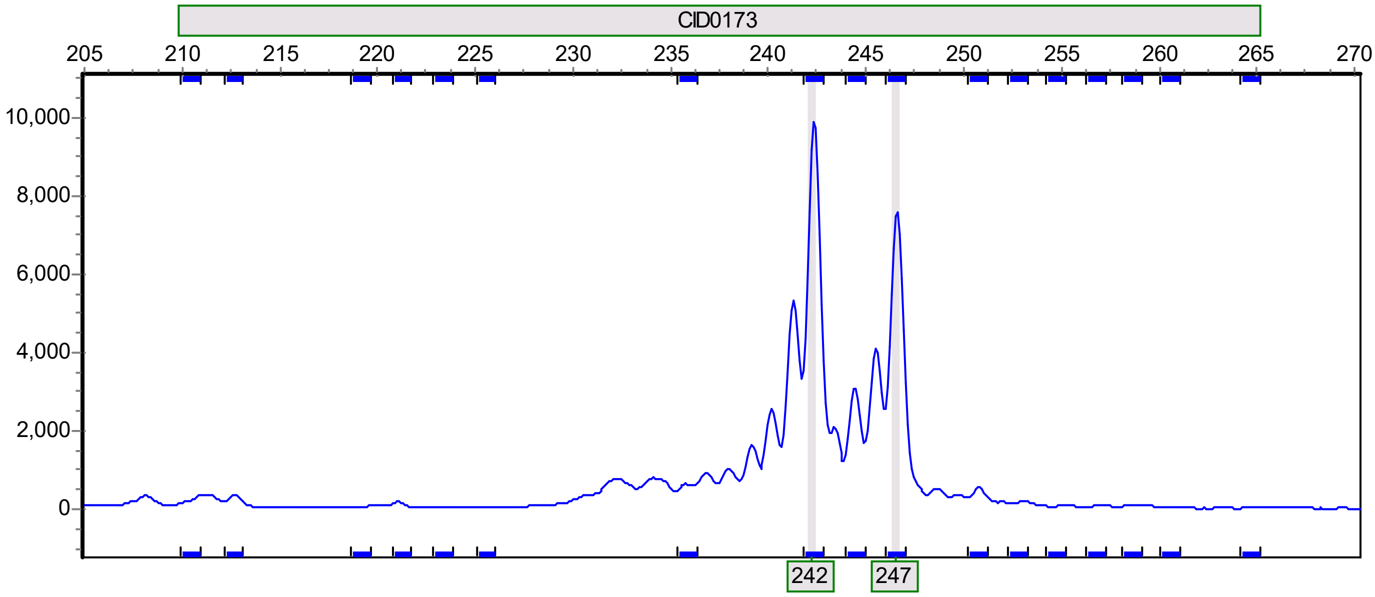

No	Size	Height	Area	Marker	Allele	Difference	Quality	Score	Allele Comments	Sample Comments
1	163.0	31897	211262	EST1573	163	0.10	Pass	500.0	[<Confirmed>]	
2	174.5	19638	124084	EST1573	174	0.10	Pass	500.0	[<Confirmed>]	
3	242.3	9892	73523	CID0173	242	0.10	Pass	500.0	[<Confirmed>]	
4	246.6	7572	57788	CID0173	247	0.00	Pass	500.0	[<Confirmed>]	

Sample 152: CID0173_EST1573_KC5_G19.fsa Run date and time: 09/21/2024 - 03:00:19 -> 09/21/2024 - 03:27:31

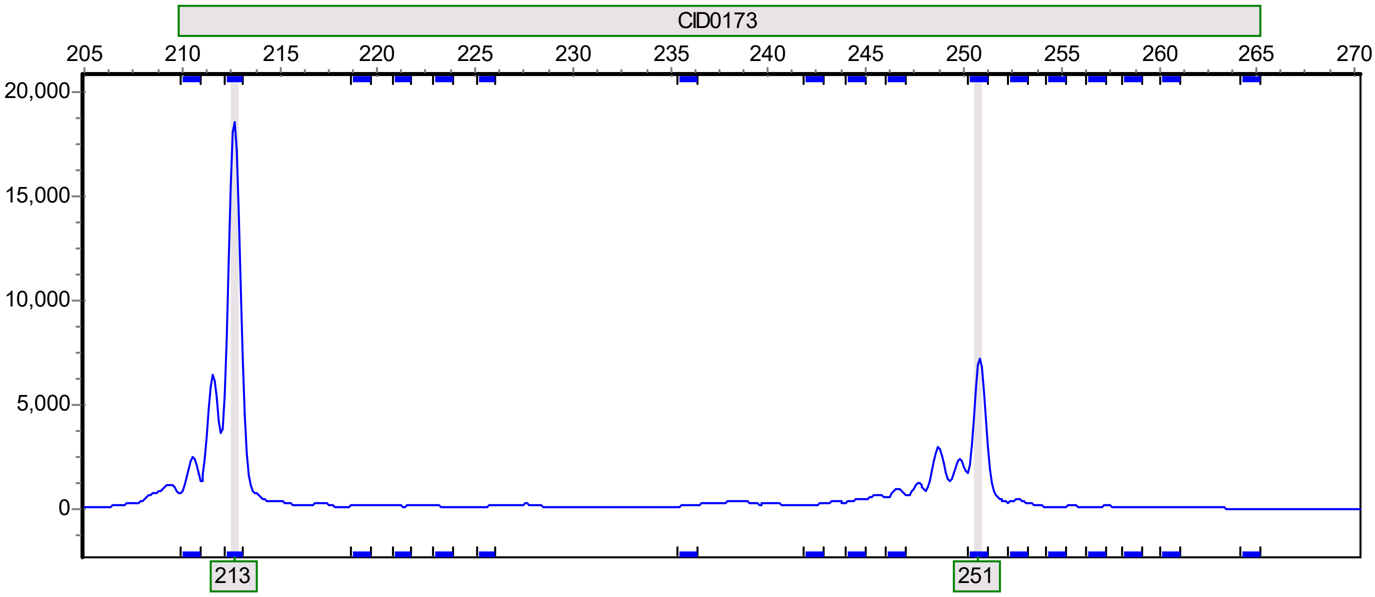

No	Size	Height	Area	Marker	Allele	Difference	Quality	Score	Allele Comments	Sample Comments
1	174.3	14750	96837	EST1573	174	0.10	Pass	500.0	[<Confirmed>]	
2	178.3	15869	104022	EST1573	178	0.10	Pass	500.0	[<Confirmed>]	
3	211.6	6463	44131	CID0173	212	0.00	Undetermined	500.0	[<Deleted>]	
4	212.7	18575	130757	CID0173	213	0.00	Pass	500.0	[<Confirmed>]	
5	250.8	7233	51116	CID0173	251	0.00	Pass	500.0	[<Confirmed>]	

Sample 153: CID0173_EST1573_KC60_F09.fsa Run date and time: 09/21/2024 - 03:27:32 -> 09/21/2024 - 03:55:03

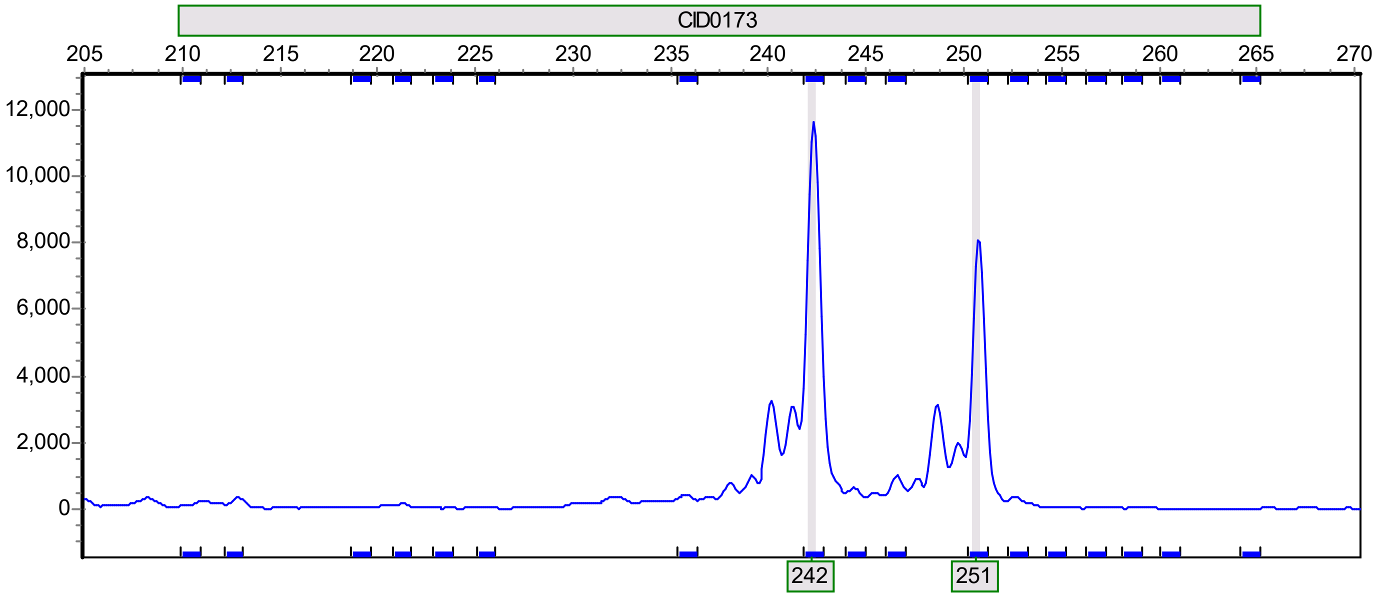

No	Size	Height	Area	Marker	Allele	Difference	Quality	Score	Allele Comments	Sample Comments
1	170.6	27710	183699	EST1573	171	0.00	Pass	500.0	[<Confirmed>]	
2	174.5	20968	134893	EST1573	174	0.10	Pass	500.0	[<Confirmed>]	
3	242.3	11643	89437	CID0173	242	0.10	Pass	500.0	[<Confirmed>]	
4	250.7	8098	56670	CID0173	251	0.10	Pass	500.0	[<Confirmed>]	

Sample 154: CID0173_EST1573_KC61_H09.fsa Run date and time: 09/21/2024 - 03:27:32 -> 09/21/2024 - 03:55:03

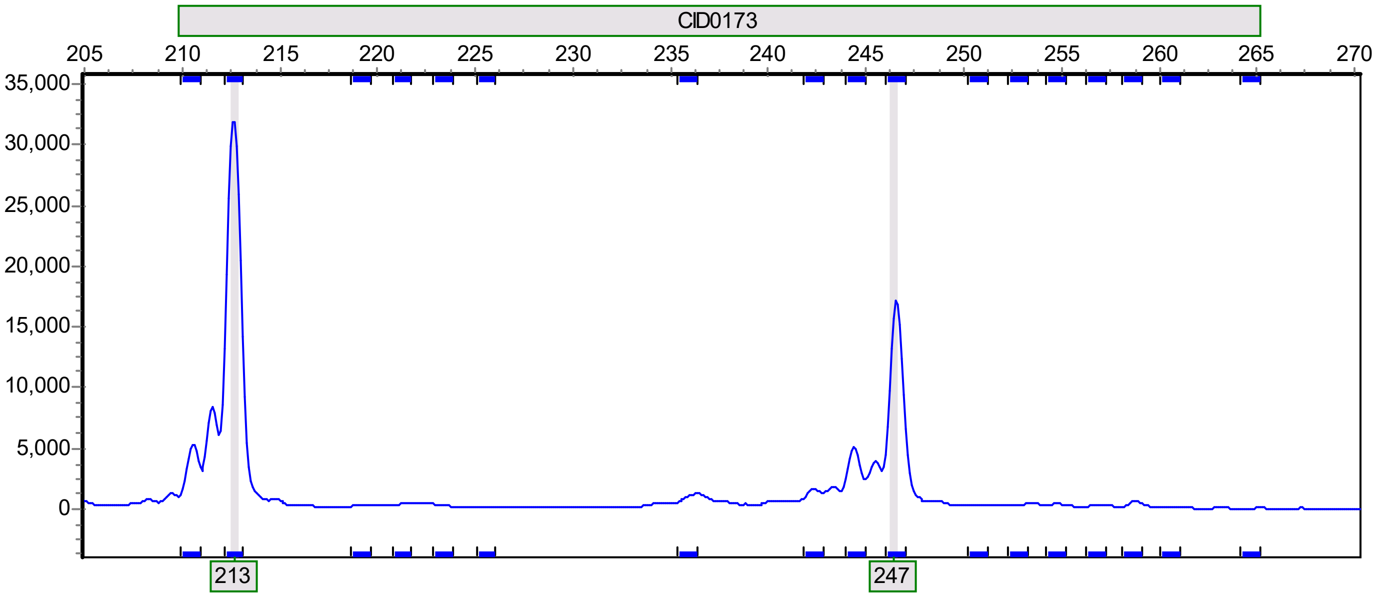

No	Size	Height	Area	Marker	Allele	Difference	Quality	Score	Allele Comments	Sample Comments
1	176.6	37087	267523	EST1573	176	0.20	Pass	500.0	[<SAT (Repaired)><Confirmed>]	
2	182.0	10637	73244	EST1573	182	0.00	Pass	500.0	[<Confirmed><Inserted>]	
3	212.7	31900	261024	CID0173	213	0.00	Pass	500.0	[<Confirmed>]	
4	246.5	17115	134119	CID0173	247	0.10	Pass	500.0	[<Confirmed>]	

Sample 155: CID0173_EST1573_KC62_J09.fsa Run date and time: 09/21/2024 - 03:27:32 -> 09/21/2024 - 03:55:03

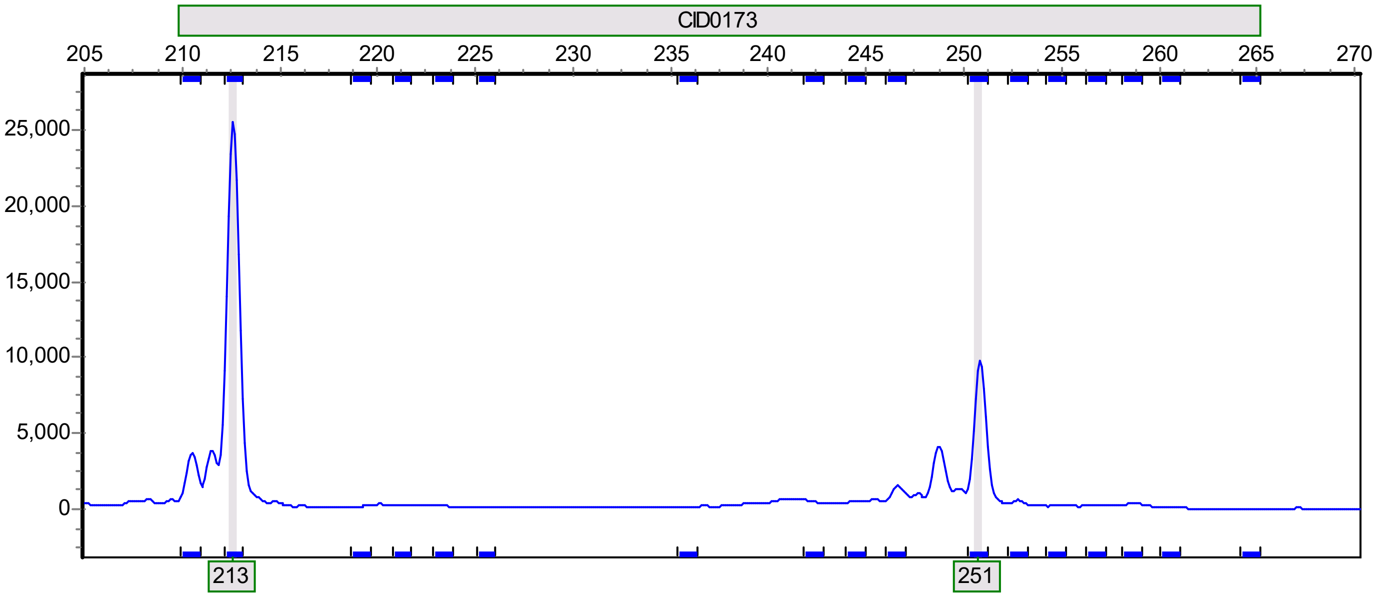

No	Size	Height	Area	Marker	Allele	Difference	Quality	Score	Allele Comments	Sample Comments
1	174.3	17918	118814	EST1573	174	0.10	Pass	500.0	[<Confirmed>]	
2	183.9	9353	60551	EST1573	184	0.10	Pass	500.0	[<Confirmed>]	
3	212.6	25556	178632	CID0173	213	0.10	Pass	500.0	[<Confirmed>]	
4	250.8	9804	65910	CID0173	251	0.00	Pass	500.0	[<Confirmed>]	

Sample 156: CID0173_EST1573_KC63_L09.fsa Run date and time: 09/21/2024 - 03:27:32 -> 09/21/2024 - 03:55:03

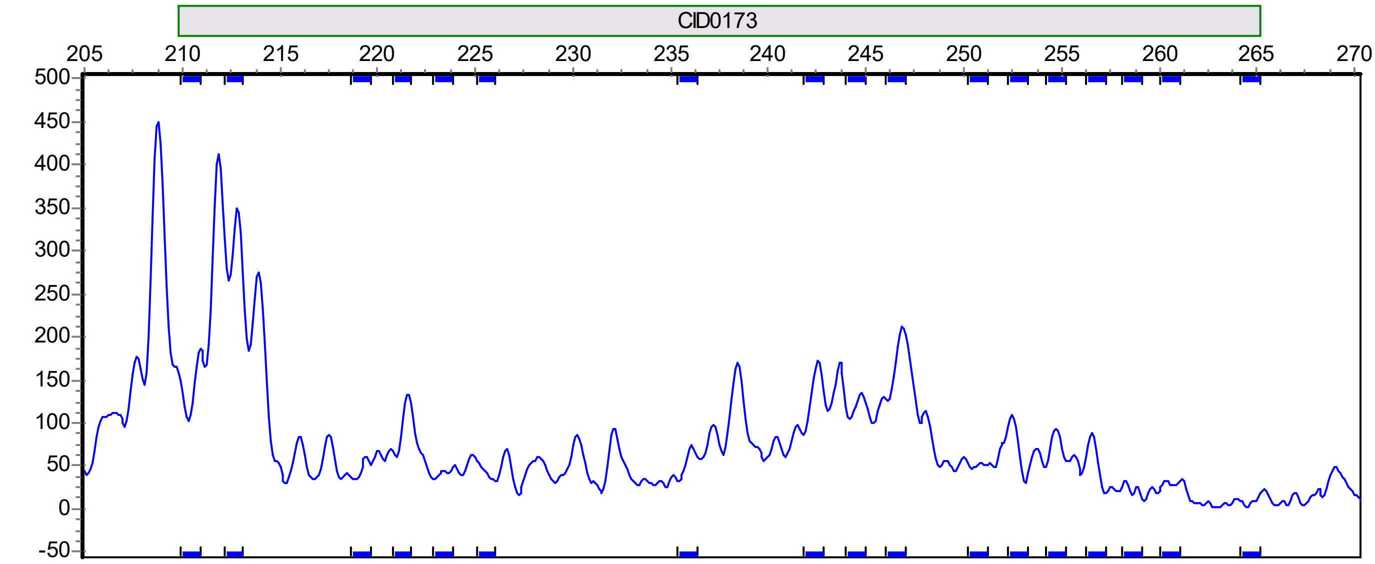

No	Size	Height	Area	Marker	Allele	Difference	Quality	Score	Allele Comments	Sample Comments
1	162.8	3917	26626	EST1573	163	0.10	Pass	500.0	[<Confirmed>]	
2	164.8	2826	19614	EST1573	165	0.00	Pass	500.0	[<Confirmed>]	
3	208.8	450	3838	CID0173	208	0.40	Pass	18.3	[<Deleted>]	
4	211.9	411	3206	CID0173	212	0.30	Pass	13.8	[<Deleted>]	
5	212.8	348	2602	CID0173	213	0.10	Undetermined	6.2	[<Deleted>]	

Allele Report

Sample 157: CID0173_EST1573_KC64_N09.fsa Run date and time: 09/21/2024 - 03:27:32 -> 09/21/2024 - 03:55:03

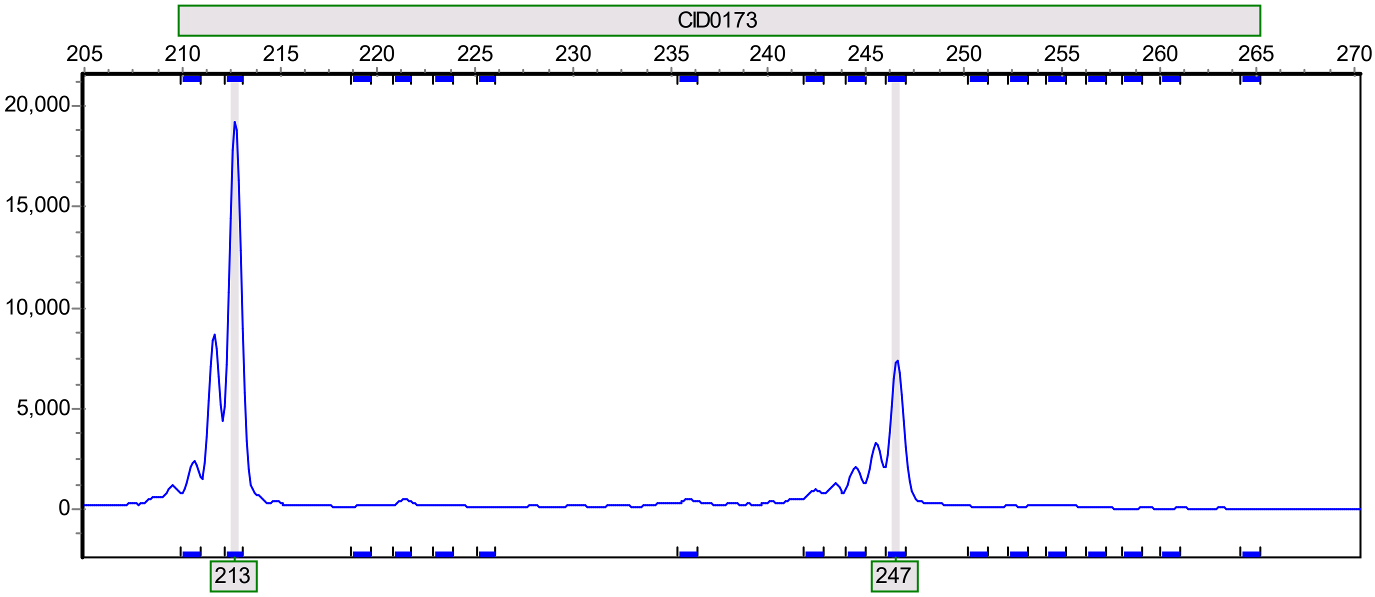

No	Size	Height	Area	Marker	Allele	Difference	Quality	Score	Allele Comments	Sample Comments
1	172.6	20870	134614	EST1573	173	0.10	Pass	500.0	[<Confirmed>]	
2	176.5	21654	140089	EST1573	176	0.10	Pass	500.0	[<Confirmed>]	
3	211.7	8653	55336	CID0173	212	0.10	Pass	500.0	[<Deleted>]	
4	212.7	19221	132336	CID0173	213	0.00	Pass	500.0	[<Confirmed>]	
5	246.6	7407	54908	CID0173	247	0.00	Pass	500.0	[<Confirmed>]	

Sample 158: CID0173_EST1573_KC65_P09.fsa Run date and time: 09/21/2024 - 03:27:32 -> 09/21/2024 - 03:55:03

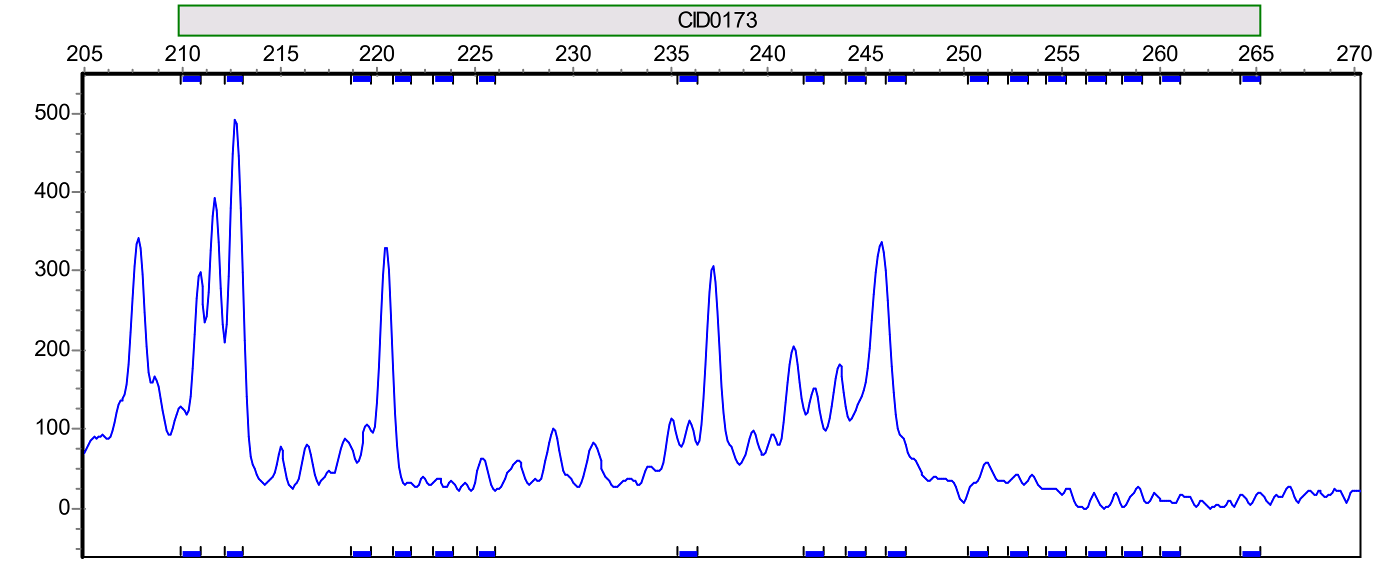

No	Size	Height	Area	Marker	Allele	Difference	Quality	Score	Allele Comments	Sample Comments
1	174.5	10396	67207	EST1573	174	0.10	Pass	500.0	[<Confirmed>]	
2	211.7	393	2820	CID0173	212	0.10	Pass	14.2	[<Deleted>]	
3	212.7	490	3822	CID0173	213	0.00	Pass	25.7	[<Deleted>]	

Sample 159: CID0173_EST1573_KC66_B11.fsa Run date and time: 09/21/2024 - 03:27:32 -> 09/21/2024 - 03:55:03

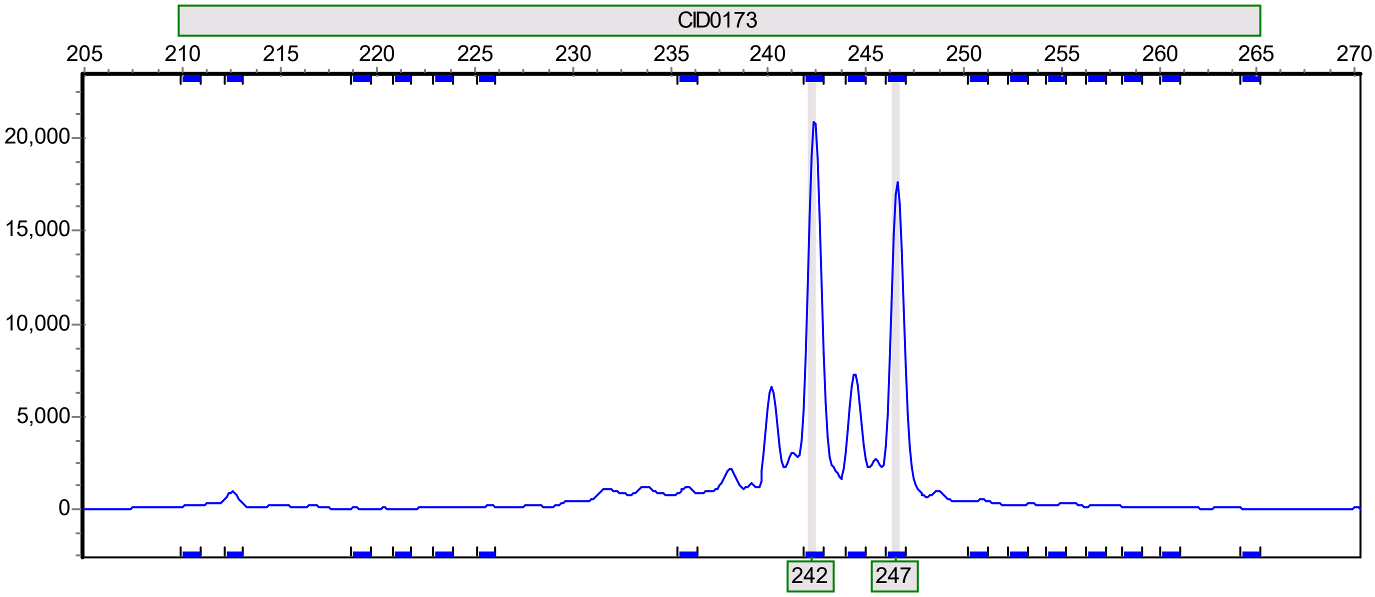

No	Size	Height	Area	Marker	Allele	Difference	Quality	Score	Allele Comments	Sample Comments
1	174.5	36687	262883	EST1573	174	0.10	Pass	500.0	[<SAT (Repaired)><Confirmed>]	
2	242.3	20897	164408	CID0173	242	0.10	Pass	500.0	[<Confirmed>]	
3	246.6	17695	130757	CID0173	247	0.00	Pass	500.0	[<Confirmed>]	

Sample 160: CID0173_EST1573_KC67_D11.fsa Run date and time: 09/21/2024 - 03:27:32 -> 09/21/2024 - 03:55:03

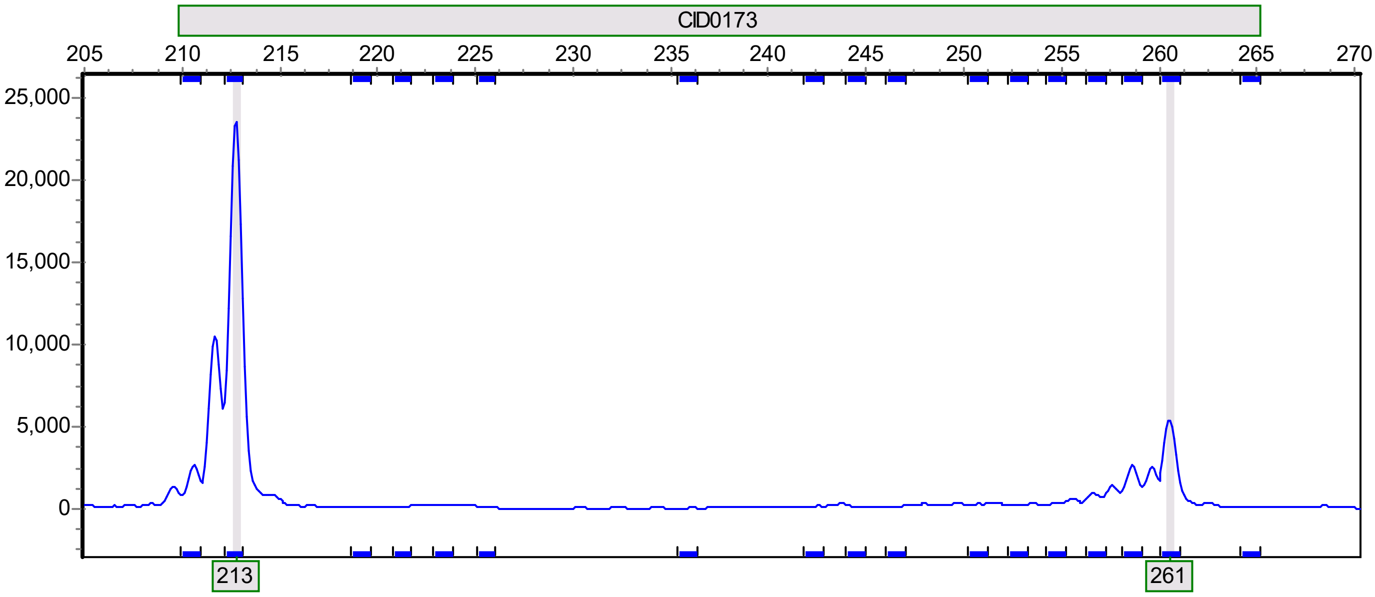

No	Size	Height	Area	Marker	Allele	Difference	Quality	Score	Allele Comments	Sample Comments
1	163.0	34708	238773	EST1573	163	0.10	Pass	500.0	[<SAT (Repaired)><Confirmed>]	
2	174.3	15254	100253	EST1573	174	0.10	Pass	500.0	[<Confirmed>]	
3	211.7	10532	73877	CID0173	212	0.10	Pass	500.0	[<Deleted>]	
4	212.8	23517	169470	CID0173	213	0.10	Pass	500.0	[<Confirmed>]	
5	260.6	5432	41131	CID0173	261	0.00	Pass	500.0	[<Confirmed><Inserted>]	

Sample 161: CID0173_EST1573_KC68_F11.fsa Run date and time: 09/21/2024 - 03:27:32 -> 09/21/2024 - 03:55:03

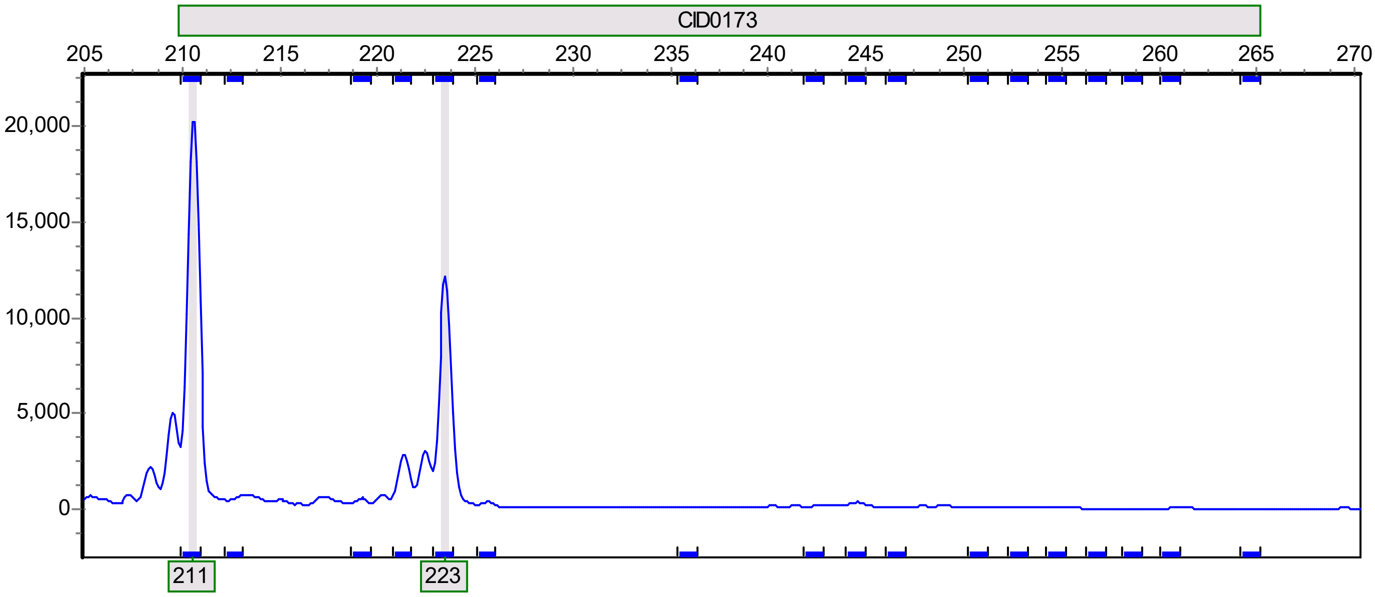

No	Size	Height	Area	Marker	Allele	Difference	Quality	Score	Allele Comments	Sample Comments
1	162.9	31669	208278	EST1573	163	0.00	Pass	500.0	[<Confirmed>]	
2	174.4	12239	79269	EST1573	174	0.00	Pass	500.0	[<Confirmed>]	
3	210.6	20244	145579	CID0173	211	0.10	Pass	500.0	[<Confirmed>]	
4	223.5	12211	88740	CID0173	223	0.10	Pass	500.0	[<Confirmed>]	

Sample 162: CID0173_EST1573_KC69_H11.fsa Run date and time: 09/21/2024 - 03:27:32 -> 09/21/2024 - 03:55:03

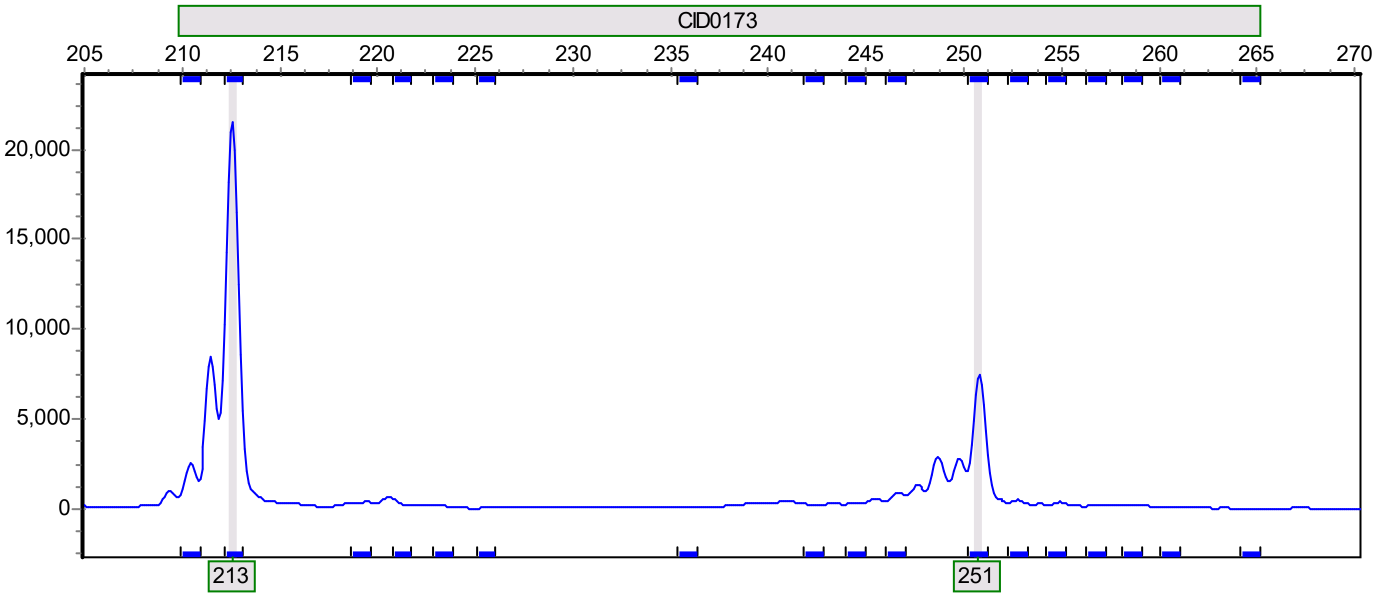

No	Size	Height	Area	Marker	Allele	Difference	Quality	Score	Allele Comments	Sample Comments
1	162.9	27808	183865	EST1573	163	0.00	Pass	500.0	[<Confirmed>]	
2	170.5	17532	115131	EST1573	171	0.10	Pass	500.0	[<Confirmed>]	
3	211.5	8417	59617	CID0173	212	0.10	Pass	500.0	[<Deleted>]	
4	212.6	21511	154822	CID0173	213	0.10	Pass	500.0	[<Confirmed>]	
5	250.8	7440	54102	CID0173	251	0.00	Pass	500.0	[<Confirmed>]	

Allele Report

Sample 163: CID0173_EST1573_KC6_I19.fsa Run date and time: 09/21/2024 - 03:00:19 -> 09/21/2024 - 03:27:31

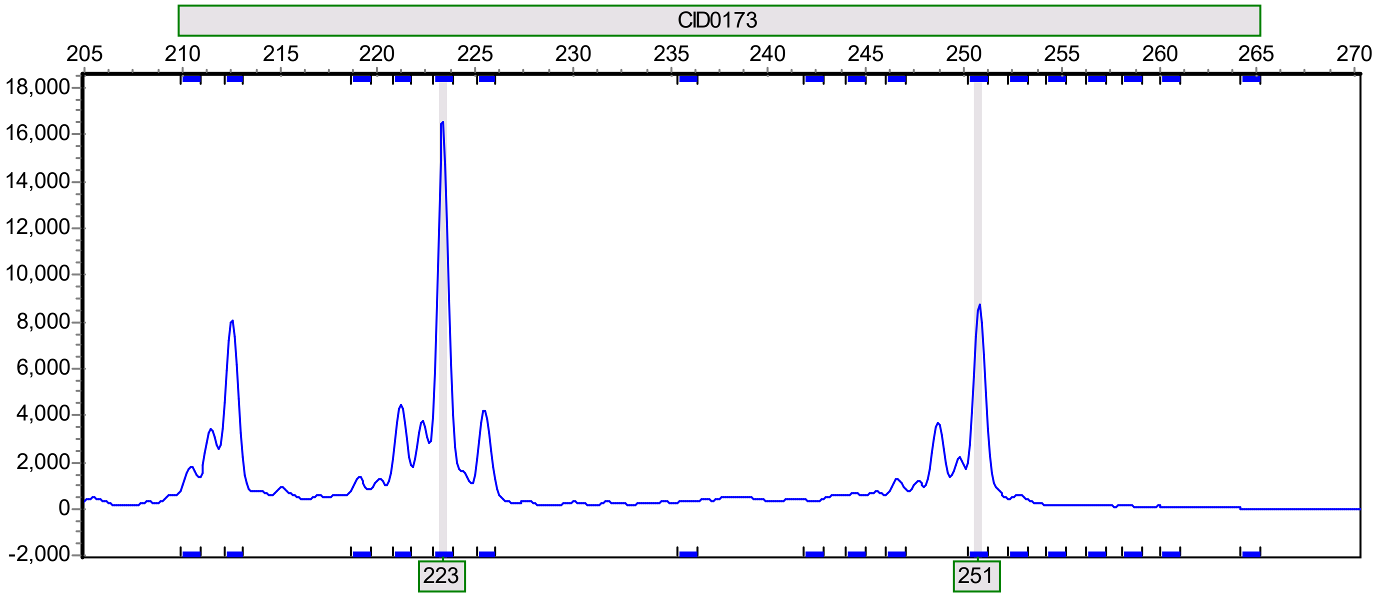

No	Size	Height	Area	Marker	Allele	Difference	Quality	Score	Allele Comments	Sample Comments
1	163.0	31408	214757	EST1573	163	0.10	Pass	500.0	[<Confirmed>]	
2	172.6	24581	163449	EST1573	173	0.10	Pass	500.0	[<Confirmed>]	
3	212.6	8028	60944	CID0173	213	0.10	Undetermined	500.0	[<Deleted>]	
4	223.4	16538	124527	CID0173	223	0.00	Pass	500.0	[<Confirmed>]	
5	250.8	8704	62405	CID0173	251	0.00	Pass	500.0	[<Confirmed>]	

Sample 164: CID0173_EST1573_KC70_J11.fsa Run date and time: 09/21/2024 - 03:27:32 -> 09/21/2024 - 03:55:03

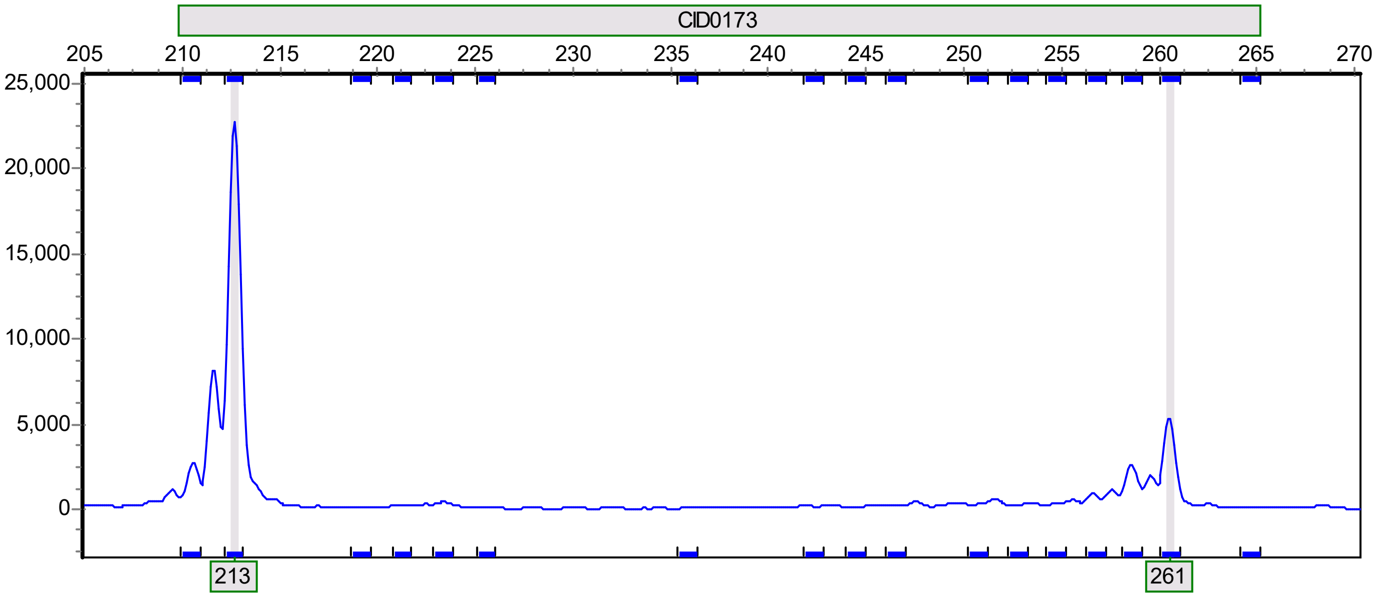

No	Size	Height	Area	Marker	Allele	Difference	Quality	Score	Allele Comments	Sample Comments
1	174.4	31237	207295	EST1573	174	0.00	Pass	500.0	[<Confirmed>]	
2	211.6	8112	53309	CID0173	212	0.00	Pass	500.0	[<Deleted>]	
3	212.7	22759	162522	CID0173	213	0.00	Pass	500.0	[<Confirmed>]	
4	260.6	5275	38696	CID0173	261	0.00	Pass	500.0	[<Confirmed><Inserted>]	

Sample 165: CID0173_EST1573_KC71_L11.fsa Run date and time: 09/21/2024 - 03:27:32 -> 09/21/2024 - 03:55:03

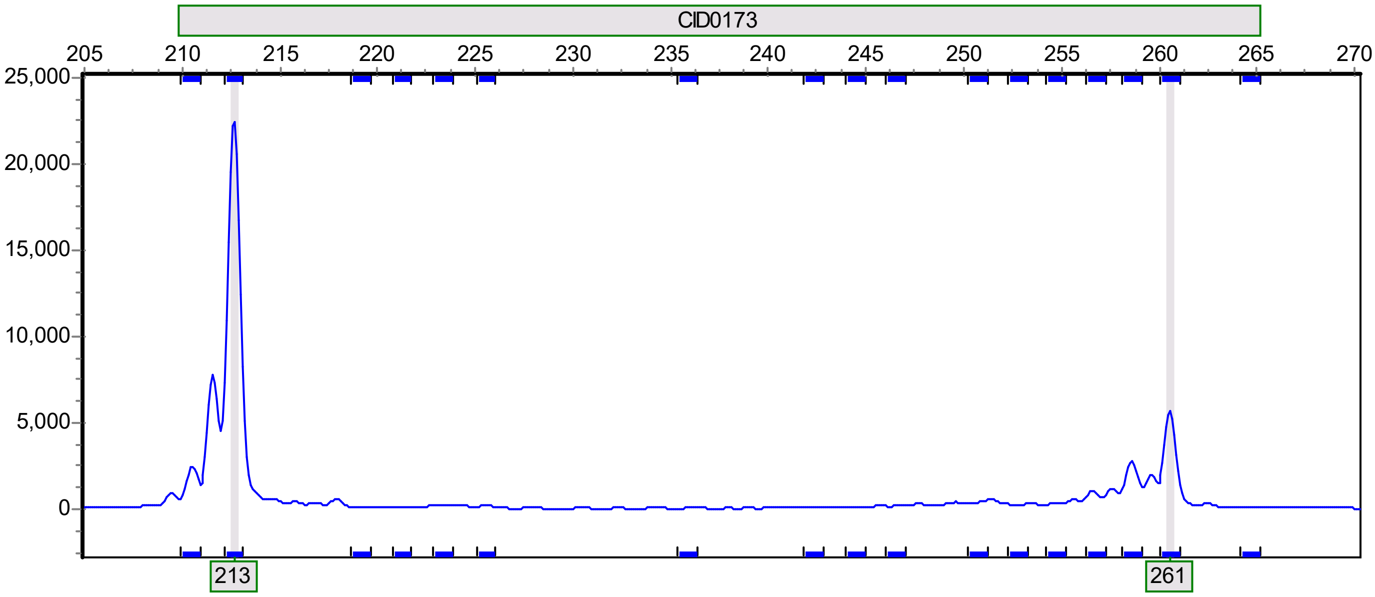

No	Size	Height	Area	Marker	Allele	Difference	Quality	Score	Allele Comments	Sample Comments
1	174.3	30909	203146	EST1573	174	0.10	Pass	500.0	[<Confirmed>]	
2	211.6	7777	54650	CID0173	212	0.00	Pass	500.0	[<Deleted>]	
3	212.7	22489	165556	CID0173	213	0.00	Pass	500.0	[<Confirmed>]	
4	260.6	5714	40817	CID0173	261	0.00	Pass	500.0	[<Confirmed><Inserted>]	

Sample 166: CID0173_EST1573_KC72_N11.fsa Run date and time: 09/21/2024 - 03:27:32 -> 09/21/2024 - 03:55:03

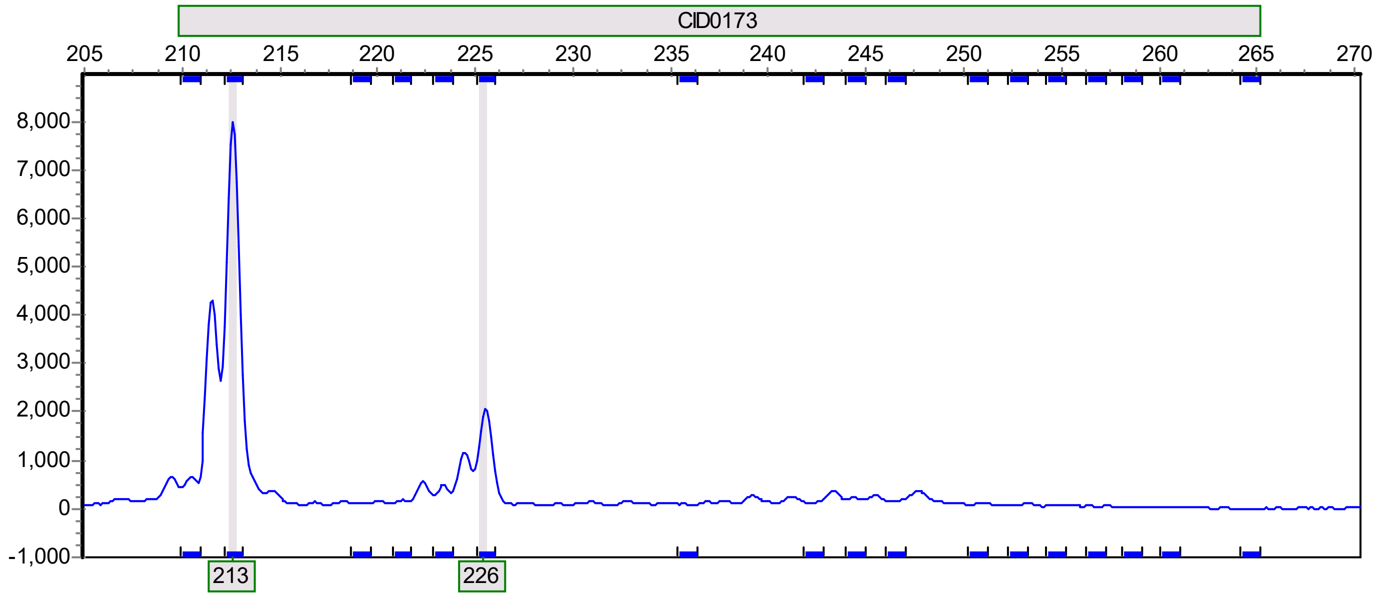

No	Size	Height	Area	Marker	Allele	Difference	Quality	Score	Allele Comments	Sample Comments
1	174.2	11104	77811	EST1573	174	0.20	Pass	500.0	[<Confirmed>]	
2	181.8	3063	21429	EST1573	182	0.20	Pass	500.0	[<Confirmed><Inserted>]	
3	211.6	4296	30369	CID0173	212	0.00	Pass	500.0	[<Deleted>]	
4	212.6	7981	61836	CID0173	213	0.10	Pass	500.0	[<Confirmed>]	
5	225.5	2045	15508	CID0173	226	0.10	Pass	258.8	[<Confirmed><Inserted>]	

Allele Report

Sample 167: CID0173_EST1573_KC73_P11.fsa Run date and time: 09/21/2024 - 03:27:32 -> 09/21/2024 - 03:55:03

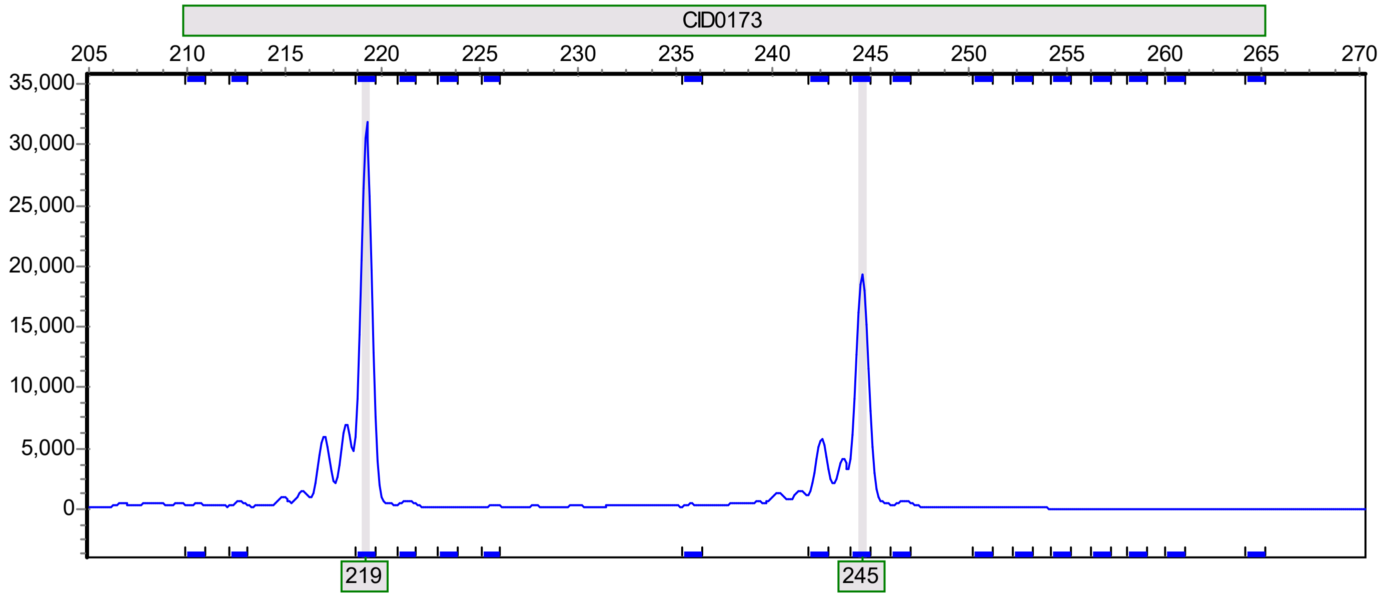

No	Size	Height	Area	Marker	Allele	Difference	Quality	Score	Allele Comments	Sample Comments
1	172.6	30694	273032	EST1573	173	0.10	Pass	500.0	[<Confirmed>]	
2	174.4	29340	188261	EST1573	174	0.00	Pass	500.0	[<Confirmed>]	
3	219.2	31875	229655	CID0173	219	0.00	Pass	500.0	[<Confirmed>]	
4	244.6	19288	139170	CID0173	245	0.10	Pass	500.0	[<Confirmed>]	

Sample 168: CID0173_EST1573_KC74_B13.fsa Run date and time: 09/21/2024 - 03:27:32 -> 09/21/2024 - 03:55:03

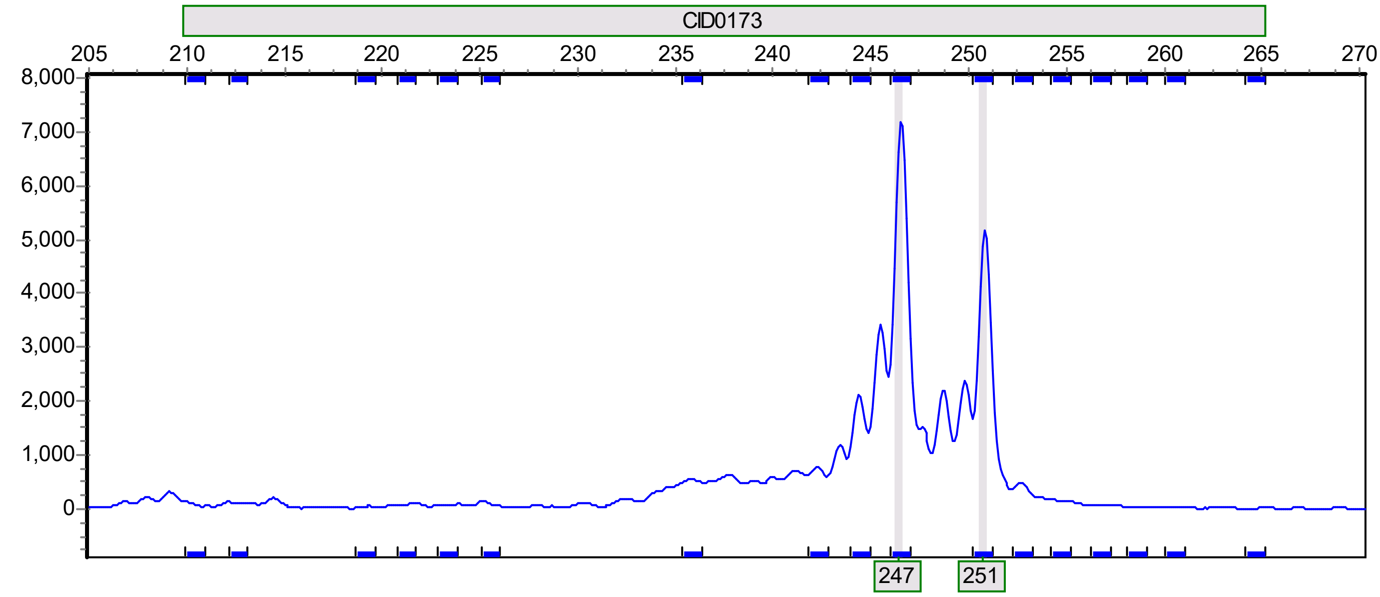

No	Size	Height	Area	Marker	Allele	Difference	Quality	Score	Allele Comments	Sample Comments
1	172.5	18743	128287	EST1573	173	0.00	Pass	500.0	[<Confirmed>]	
2	176.4	19283	129550	EST1573	176	0.00	Pass	500.0	[<Confirmed>]	
3	246.5	7180	58517	CID0173	247	0.10	Pass	500.0	[<Confirmed>]	
4	249.8	2369	17503	CID0173	250	0.00	Undetermined	203.1	[<Deleted>]	
5	250.8	5176	40060	CID0173	251	0.00	Pass	500.0	[<Confirmed>]	

Sample 169: CID0173_EST1573_KC75_D13.fsa Run date and time: 09/21/2024 - 03:27:32 -> 09/21/2024 - 03:55:03

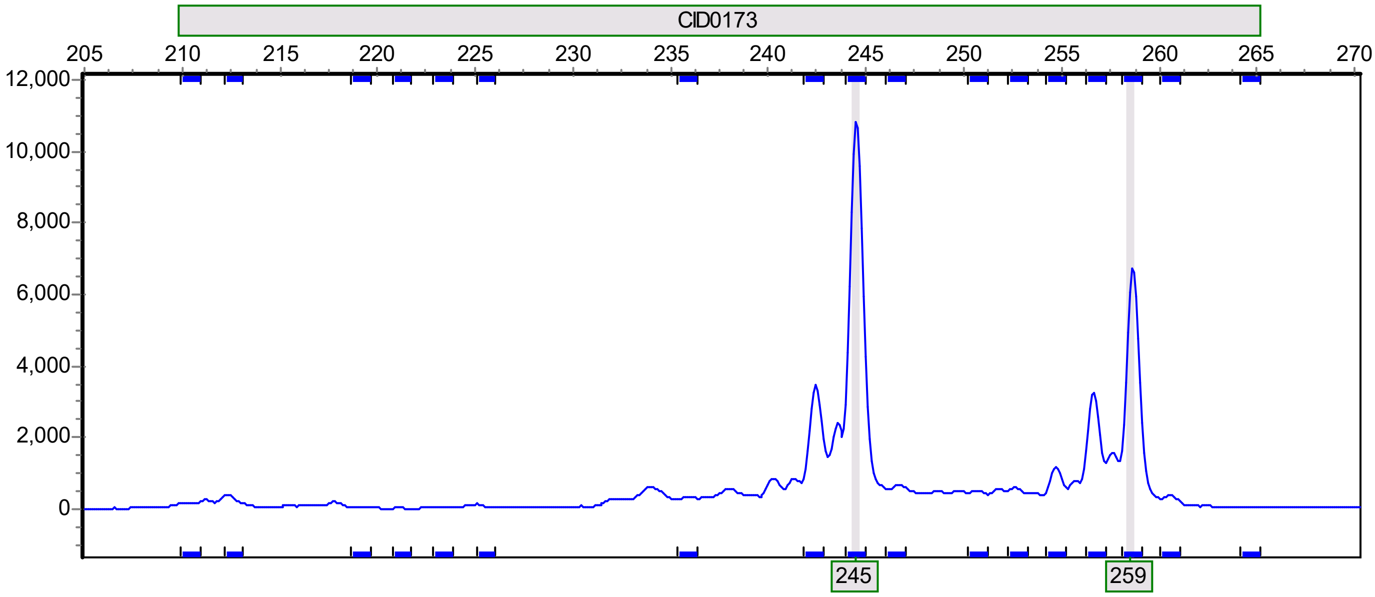

No	Size	Height	Area	Marker	Allele	Difference	Quality	Score	Allele Comments	Sample Comments
1	162.9	31777	217715	EST1573	163	0.00	Pass	500.0	[<Confirmed>]	
2	176.1	12120	82383	EST1573	176	0.30	Pass	500.0	[<Confirmed>]	
3	244.5	10814	83982	CID0173	245	0.00	Pass	500.0	[<Confirmed>]	
4	258.6	6734	48106	CID0173	259	0.10	Pass	500.0	[<Confirmed>]	

Sample 170: CID0173_EST1573_KC76_F13.fsa Run date and time: 09/21/2024 - 03:27:32 -> 09/21/2024 - 03:55:03

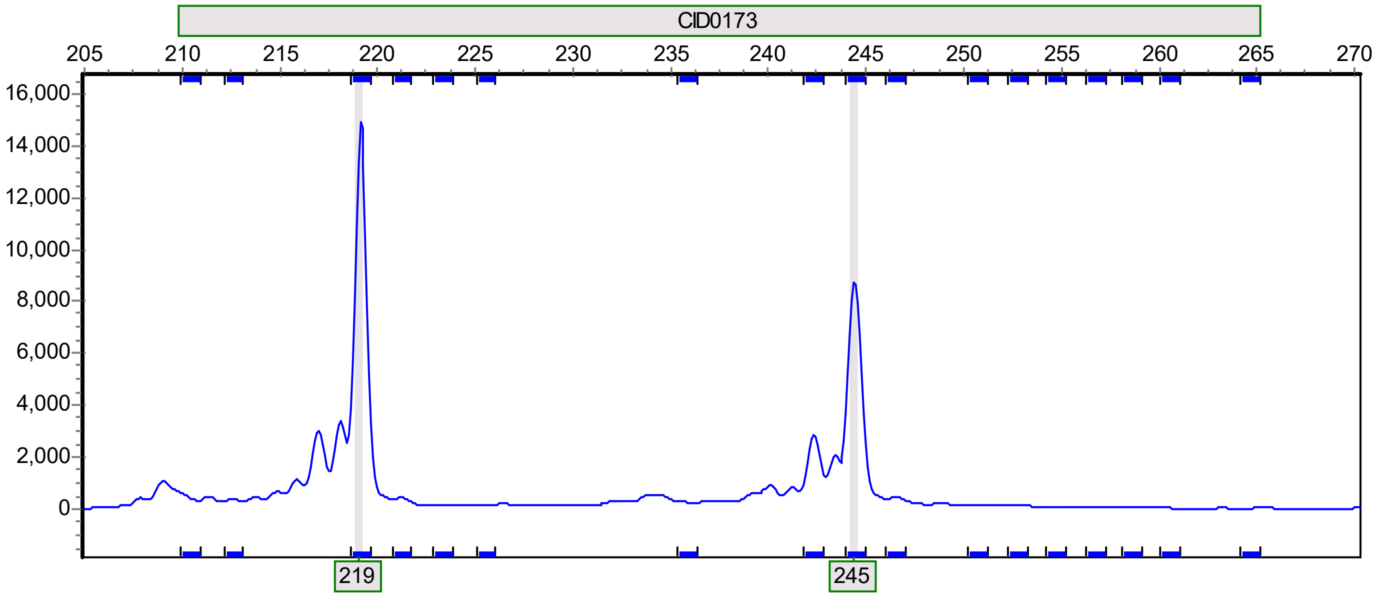

No	Size	Height	Area	Marker	Allele	Difference	Quality	Score	Allele Comments	Sample Comments
1	162.8	24664	163829	EST1573	163	0.10	Pass	500.0	[<Confirmed>]	
2	164.8	20323	138328	EST1573	165	0.00	Pass	500.0	[<Confirmed>]	
3	219.1	14913	112165	CID0173	219	0.10	Pass	500.0	[<Confirmed>]	
4	244.4	8698	70265	CID0173	245	0.10	Pass	500.0	[<Confirmed>]	

Sample 171: CID0173_EST1573_KC77_H13.fsa Run date and time: 09/21/2024 - 03:27:32 -> 09/21/2024 - 03:55:03

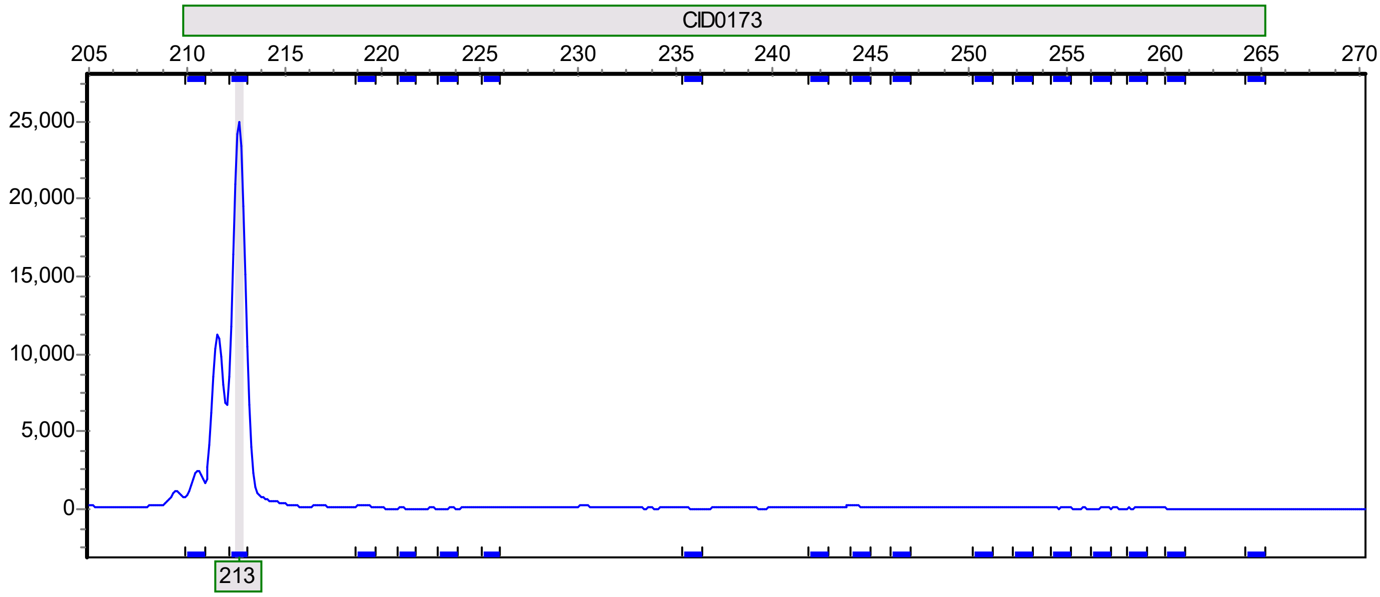

No	Size	Height	Area	Marker	Allele	Difference	Quality	Score	Allele Comments	Sample Comments
1	162.7	21308	142903	EST1573	163	0.20	Pass	500.0	[<Confirmed>]	
2	170.5	18716	122583	EST1573	171	0.10	Pass	500.0	[<Confirmed>]	
3	211.6	11314	78719	CID0173	212	0.00	Pass	500.0	[<Deleted>]	
4	212.7	24975	182096	CID0173	213	0.00	Pass	500.0	[<Confirmed>]	

Sample 172: CID0173_EST1573_KC78_J13.fsa Run date and time: 09/21/2024 - 03:27:32 -> 09/21/2024 - 03:55:03

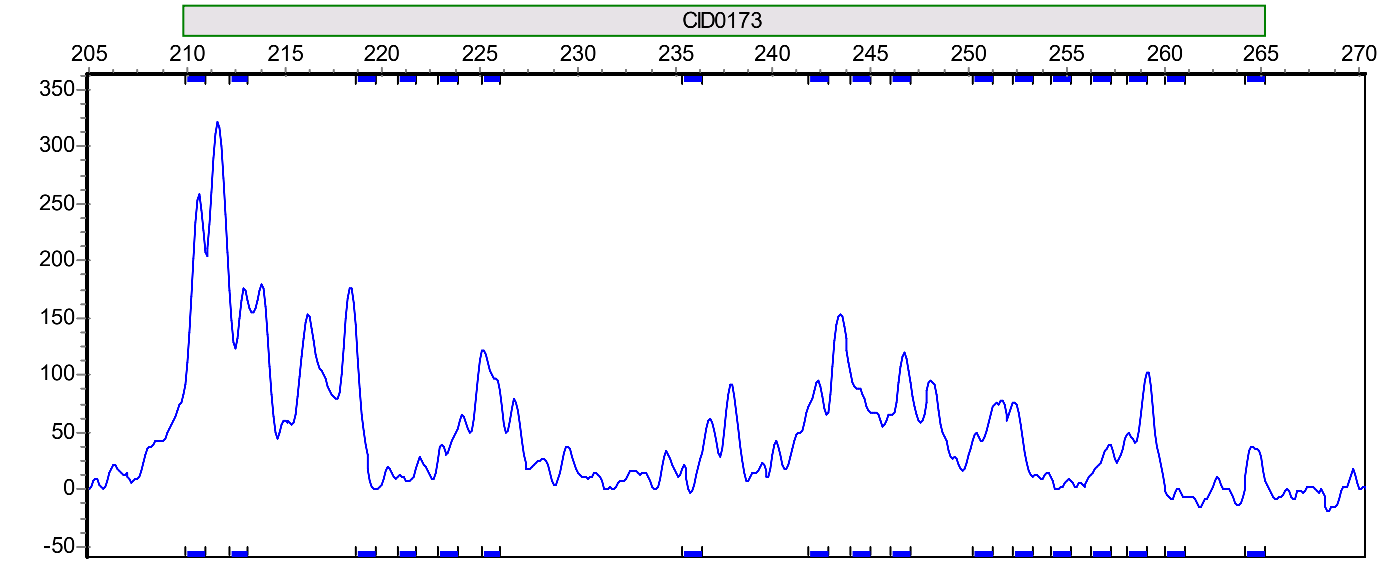

No	Size	Height	Area	Marker	Allele	Difference	Quality	Score	Allele Comments	Sample Comments
1	163.9	398	4105	EST1573	164	0.10	Pass	10.2	[<Deleted>]	
2	165.6	326	3333	EST1573	166	0.20	Undetermined	5.7	[<Deleted>]	
3	169.1	418	3195	EST1573	169	0.40	Pass	17.3	[<Deleted>]	
4	170.9	376	4071	EST1573	171	0.30	Undetermined	5.2	[<Deleted>]	
5	178.0	372	3261	EST1573	178	0.20	Undetermined	9.1	[<Deleted>]	
6	210.6	259	2137	CID0173	211	0.10	Check	3.6	[<Deleted>]	
7	211.6	322	3286	CID0173	212	0.00	Check	4.5	[<Deleted>]	

Allele Report

Sample 173: CID0173_EST1573_KC79_L13.fsa Run date and time: 09/21/2024 - 03:27:32 -> 09/21/2024 - 03:55:03

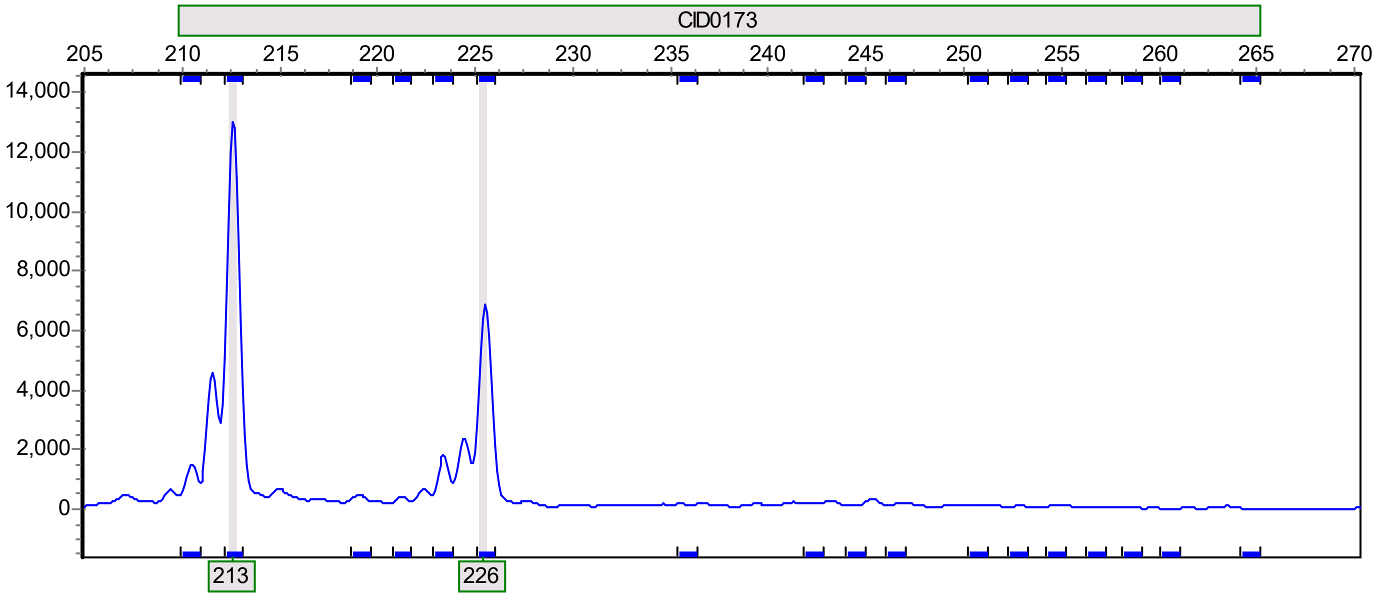

No	Size	Height	Area	Marker	Allele	Difference	Quality	Score	Allele Comments	Sample Comments
1	172.4	21349	143944	EST1573	173	0.10	Pass	500.0	[<Confirmed>]	
2	174.2	10983	76857	EST1573	174	0.20	Pass	500.0	[<Confirmed>]	
3	211.6	4596	29601	CID0173	212	0.00	Undetermined	500.0	[<Deleted>]	
4	212.6	13008	90833	CID0173	213	0.10	Pass	500.0	[<Confirmed>]	
5	225.5	6871	50182	CID0173	226	0.10	Pass	500.0	[<Confirmed>]	

Sample 174: CID0173_EST1573_KC7_K19.fsa Run date and time: 09/21/2024 - 03:00:19 -> 09/21/2024 - 03:27:31

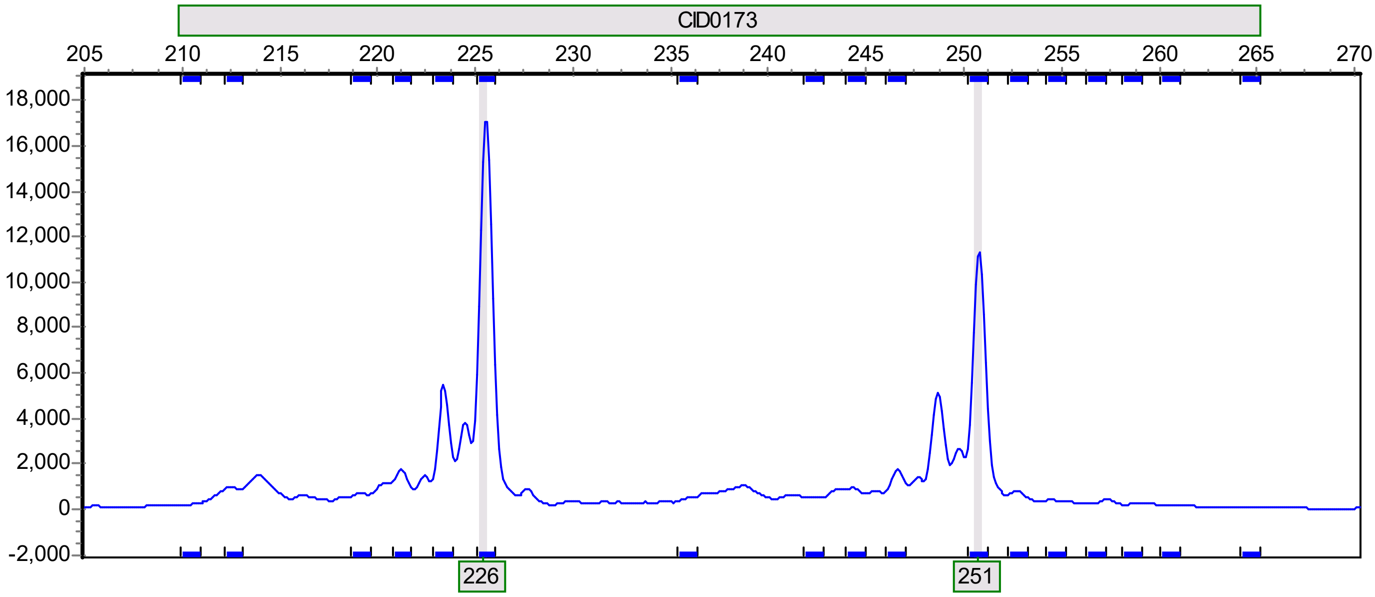

No	Size	Height	Area	Marker	Allele	Difference	Quality	Score	Allele Comments	Sample Comments
1	176.5	31306	221412	EST1573	176	0.10	Pass	500.0	[<Confirmed>]	
2	182.0	8665	61231	EST1573	182	0.00	Pass	500.0	[<Confirmed><Inserted>]	
3	225.5	17056	123533	CID0173	226	0.10	Pass	500.0	[<Confirmed>]	
4	250.8	11330	82310	CID0173	251	0.00	Pass	500.0	[<Confirmed>]	

Sample 175: CID0173_EST1573_KC80_N13.fsa Run date and time: 09/21/2024 - 03:27:32 -> 09/21/2024 - 03:55:03

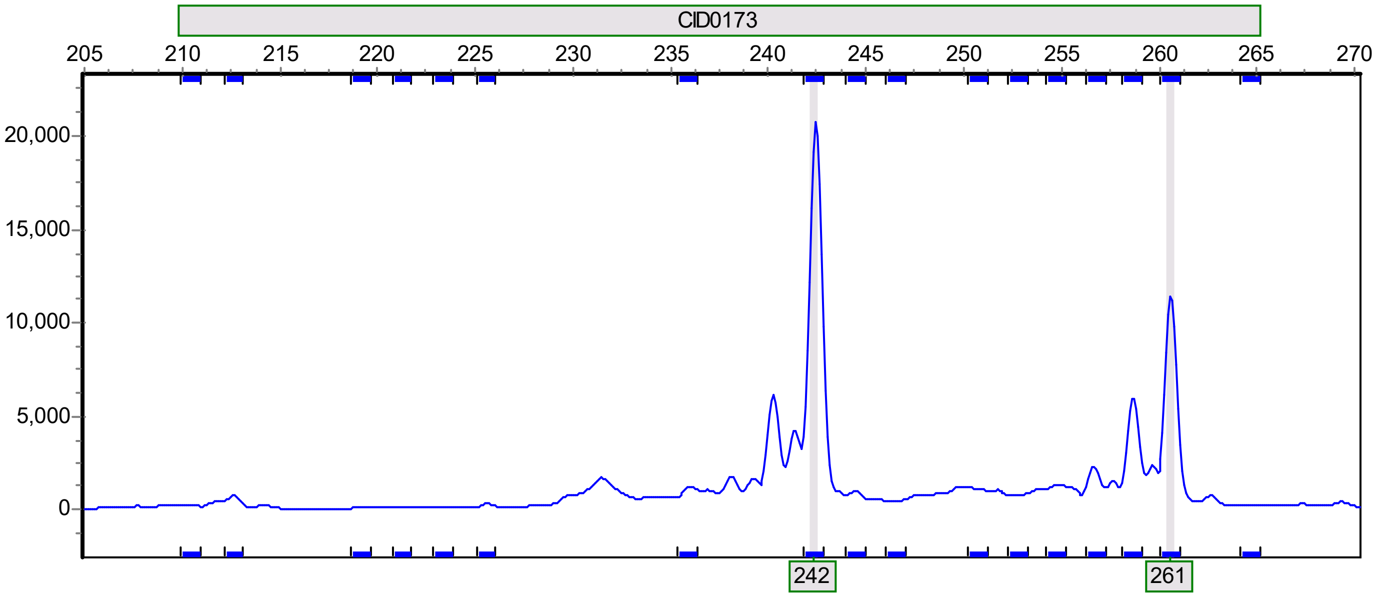

No	Size	Height	Area	Marker	Allele	Difference	Quality	Score	Allele Comments	Sample Comments
1	174.4	38137	265506	EST1573	174	0.00	Pass	500.0	[<SAT (Repaired)><Confirmed>]	
2	242.4	20801	152472	CID0173	242	0.00	Pass	500.0	[<Confirmed>]	
3	260.6	11431	78556	CID0173	261	0.00	Pass	500.0	[<Confirmed>]	

Sample 176: CID0173_EST1573_KC81_P13.fsa Run date and time: 09/21/2024 - 03:27:32 -> 09/21/2024 - 03:55:03

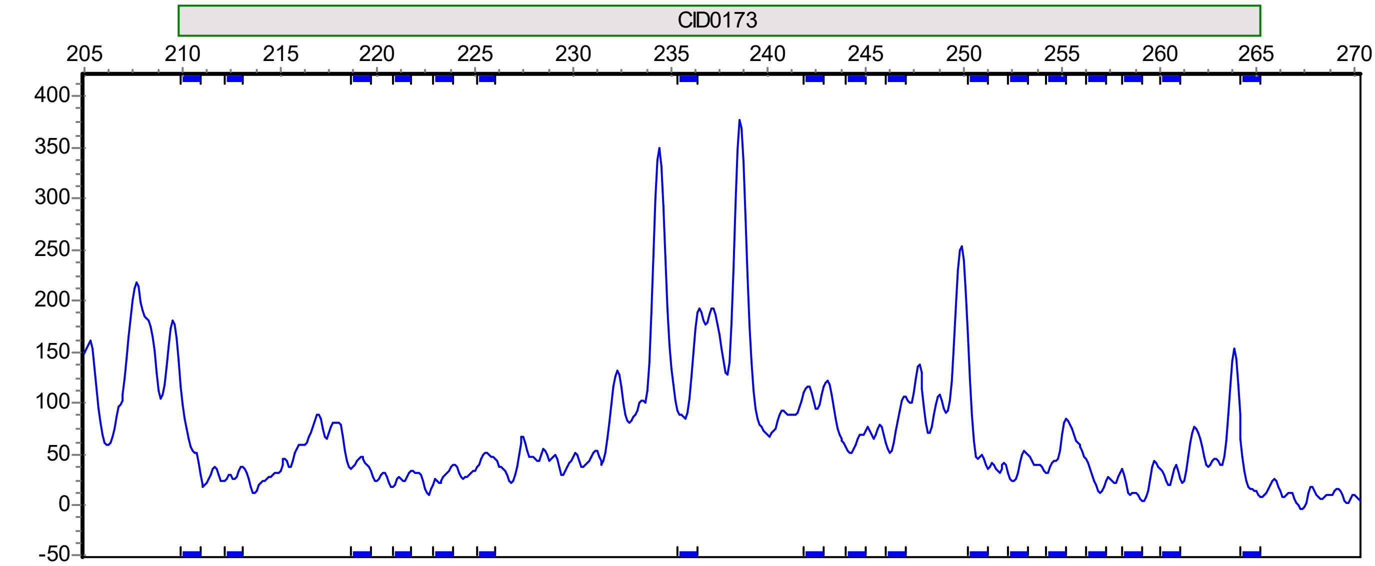

No	Size	Height	Area	Marker	Allele	Difference	Quality	Score	Allele Comments	Sample Comments
1	162.3	723	5015	EST1573	163	0.60	Pass	65.7	[<Deleted>]	
2	168.7	862	6897	EST1573	169	0.00	Pass	60.3	[<Deleted>]	
3	170.8	496	3839	EST1573	171	0.20	Undetermined	20.6	[<Deleted>]	

Sample 177: CID0173_EST1573_KC82_B15.fsa Run date and time: 09/21/2024 - 03:27:32 -> 09/21/2024 - 03:55:03

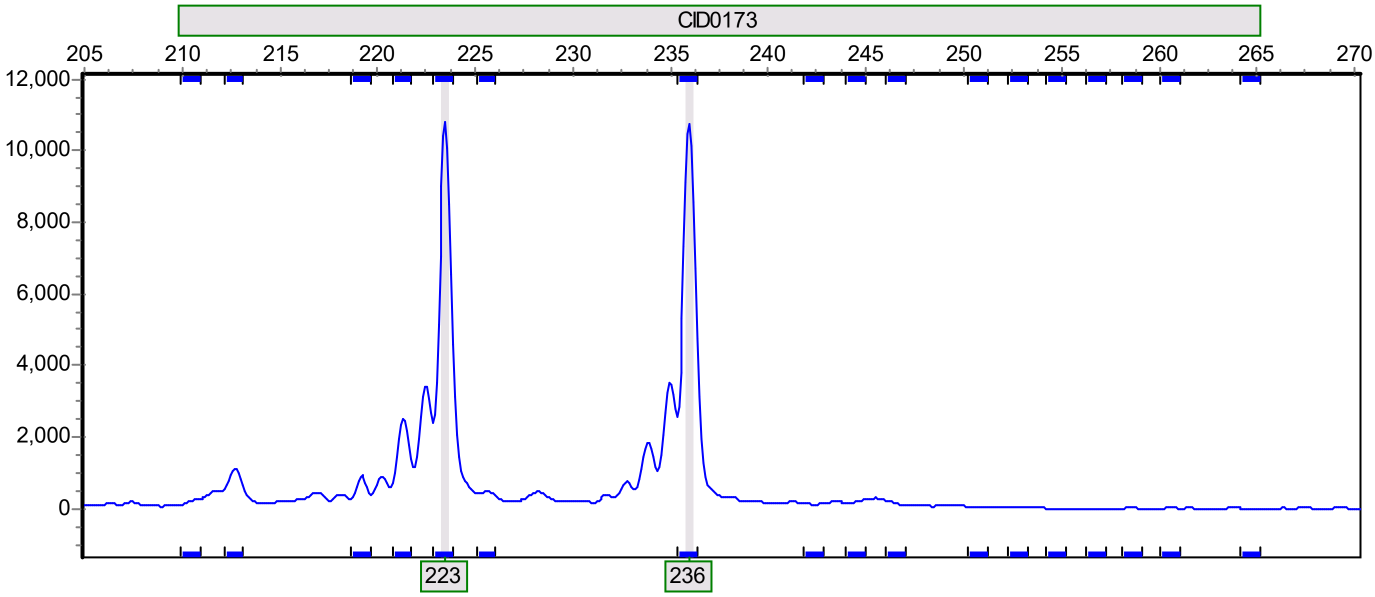

No	Size	Height	Area	Marker	Allele	Difference	Quality	Score	Allele Comments	Sample Comments
1	162.9	25672	162097	EST1573	163	0.00	Pass	500.0	[<Confirmed>]	
2	174.4	13206	89012	EST1573	174	0.00	Pass	500.0	[<Confirmed>]	
3	222.4	3430	24097	CID0173	222	0.00	Undetermined	466.8	[<Deleted>]	
4	223.5	10798	79427	CID0173	223	0.10	Pass	500.0	[<Confirmed>]	
5	236.0	10754	80531	CID0173	236	0.10	Pass	500.0	[<Confirmed>]	

Sample 178: CID0173_EST1573_KC83_D15.fsa Run date and time: 09/21/2024 - 03:27:32 -> 09/21/2024 - 03:55:03

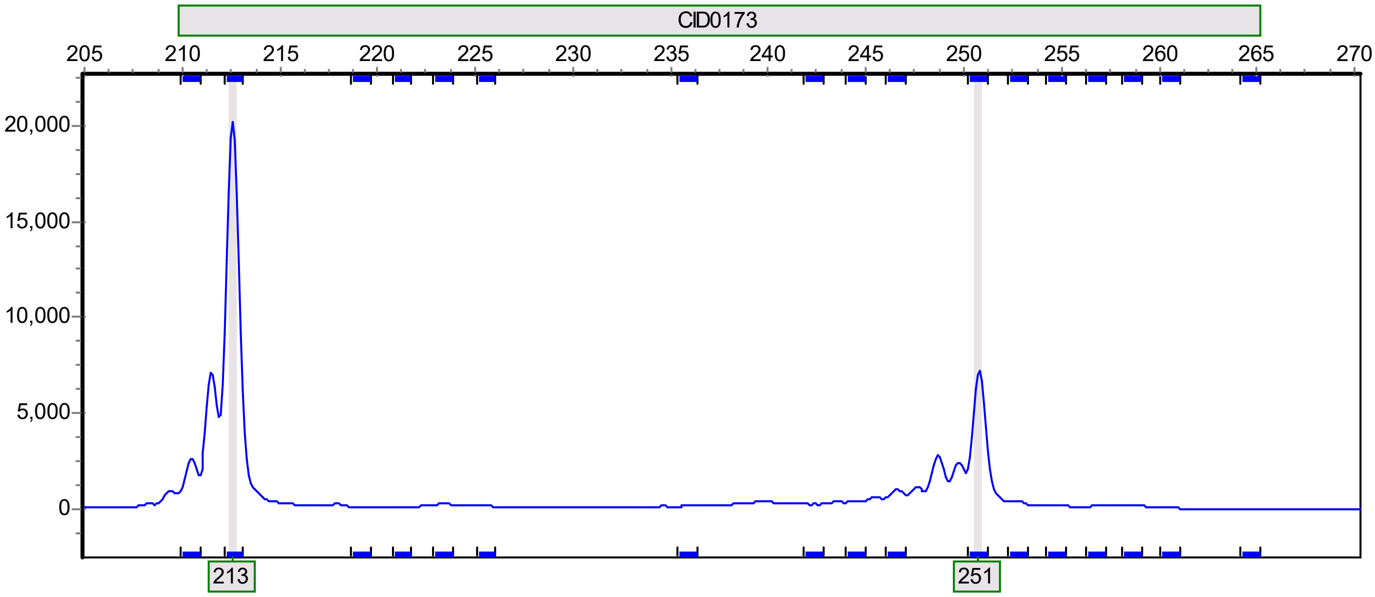

No	Size	Height	Area	Marker	Allele	Difference	Quality	Score	Allele Comments	Sample Comments
1	172.5	27786	188041	EST1573	173	0.00	Pass	500.0	[<Confirmed>]	
2	174.3	14575	100370	EST1573	174	0.10	Pass	500.0	[<Confirmed>]	
3	211.5	7126	49255	CID0173	212	0.10	Undetermined	500.0	[<Deleted>]	
4	212.6	20206	150066	CID0173	213	0.10	Pass	500.0	[<Confirmed>]	
5	250.8	7187	54011	CID0173	251	0.00	Pass	500.0	[<Confirmed>]	

Sample 179: CID0173_EST1573_KC84_F15.fsa Run date and time: 09/21/2024 - 03:27:32 -> 09/21/2024 - 03:55:03

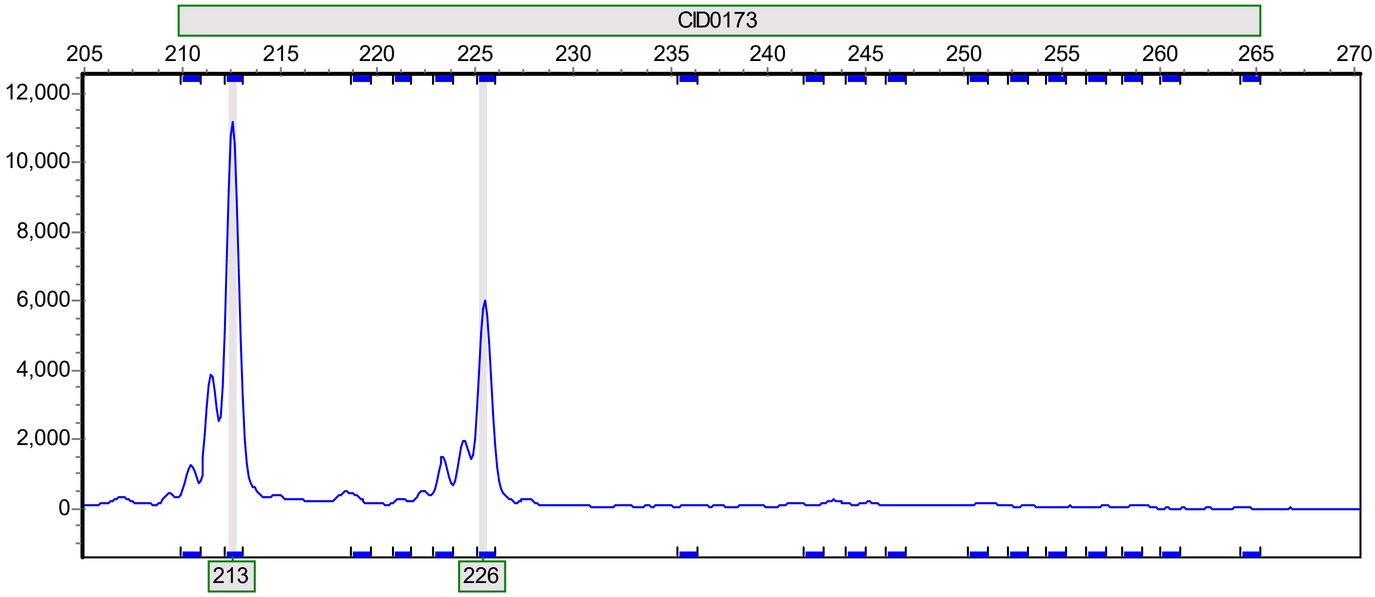

No	Size	Height	Area	Marker	Allele	Difference	Quality	Score	Allele Comments	Sample Comments
1	176.4	19142	129131	EST1573	176	0.00	Pass	500.0	[<Confirmed>]	
2	181.9	3880	28988	EST1573	182	0.10	Pass	500.0	[<Confirmed><Inserted>]	
3	211.5	3898	27646	CID0173	212	0.10	Undetermined	500.0	[<Deleted>]	
4	212.6	11162	81681	CID0173	213	0.10	Pass	500.0	[<Confirmed>]	
5	225.5	5993	44950	CID0173	226	0.10	Pass	500.0	[<Confirmed>]	

Sample 180: CID0173_EST1573_KC85_H15.fsa Run date and time: 09/21/2024 - 03:27:32 -> 09/21/2024 - 03:55:03

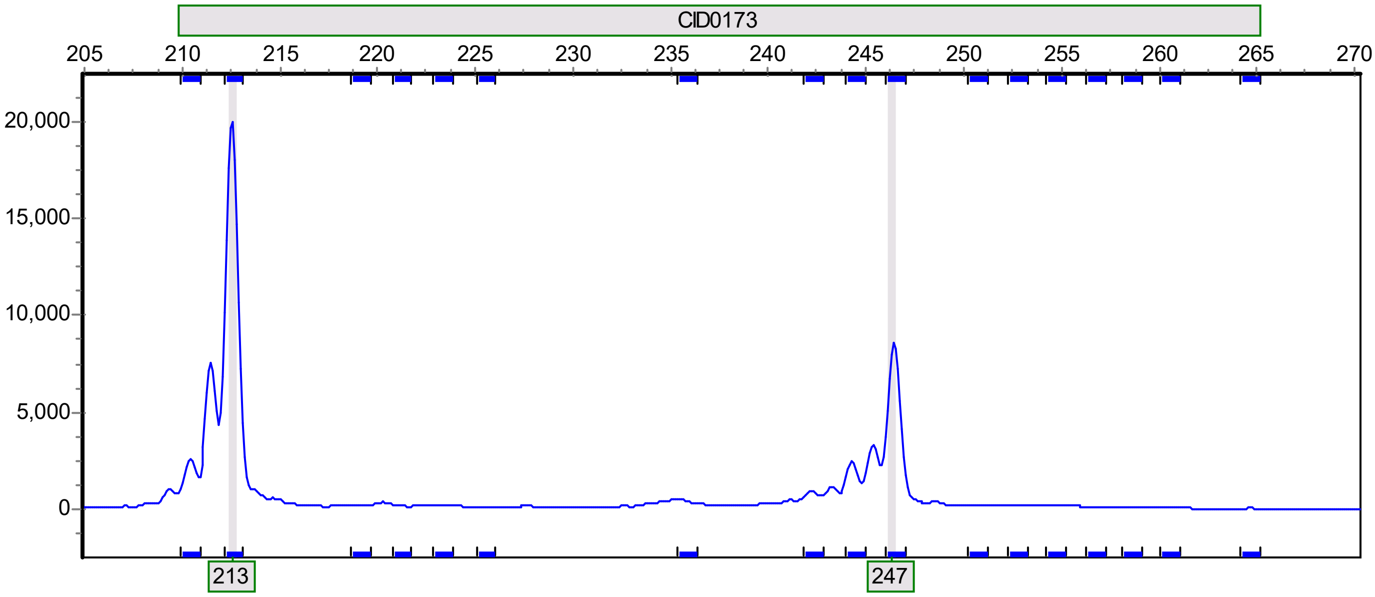

No	Size	Height	Area	Marker	Allele	Difference	Quality	Score	Allele Comments	Sample Comments
1	172.5	21175	135732	EST1573	173	0.00	Pass	500.0	[<Confirmed>]	
2	176.5	22316	145541	EST1573	176	0.10	Pass	500.0	[<Confirmed>]	
3	211.5	7539	53570	CID0173	212	0.10	Undetermined	500.0	[<Deleted>]	
4	212.6	19971	146637	CID0173	213	0.10	Pass	500.0	[<Confirmed>]	
5	246.4	8613	63408	CID0173	247	0.20	Pass	500.0	[<Confirmed>]	

Sample 181: CID0173_EST1573_KC86_J15.fsa Run date and time: 09/21/2024 - 03:27:32 -> 09/21/2024 - 03:55:03

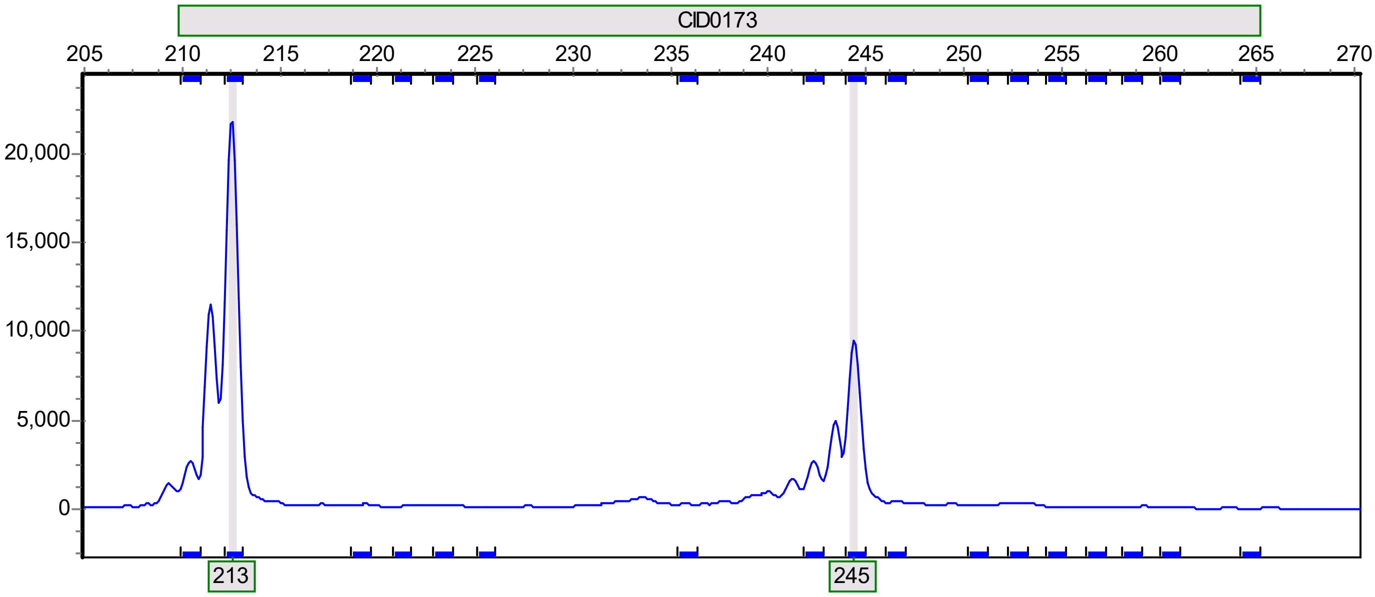

No	Size	Height	Area	Marker	Allele	Difference	Quality	Score	Allele Comments	Sample Comments
1	174.3	26342	175208	EST1573	174	0.10	Pass	500.0	[<Confirmed>]	
2	176.1	18819	124690	EST1573	176	0.30	Pass	500.0	[<Confirmed>]	
3	211.5	11527	79728	CID0173	212	0.10	Pass	500.0	[<Deleted>]	
4	212.6	21813	157678	CID0173	213	0.10	Pass	500.0	[<Confirmed>]	
5	244.4	9463	69464	CID0173	245	0.10	Pass	500.0	[<Confirmed>]	

Sample 182: CID0173_EST1573_KC87_L15.fsa Run date and time: 09/21/2024 - 03:27:32 -> 09/21/2024 - 03:55:03

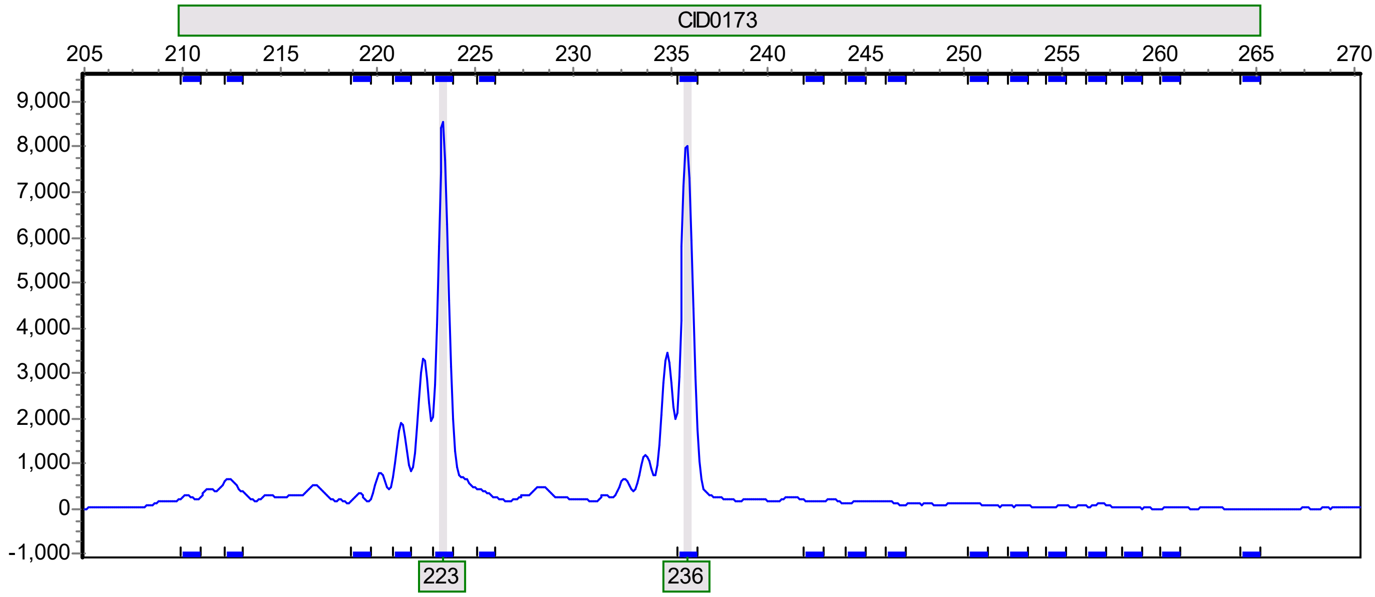

No	Size	Height	Area	Marker	Allele	Difference	Quality	Score	Allele Comments	Sample Comments
1	162.9	22908	140478	EST1573	163	0.00	Pass	500.0	[<Confirmed>]	
2	174.3	11118	73441	EST1573	174	0.10	Pass	500.0	[<Confirmed>]	
3	222.3	3322	22196	CID0173	222	0.10	Undetermined	500.0	[<Deleted>]	
4	223.4	8552	60353	CID0173	223	0.00	Pass	500.0	[<Confirmed>]	
5	235.9	8028	60021	CID0173	236	0.00	Pass	500.0	[<Confirmed>]	

Sample 183: CID0173_EST1573_KC88_N15.fsa Run date and time: 09/21/2024 - 03:27:32 -> 09/21/2024 - 03:55:03

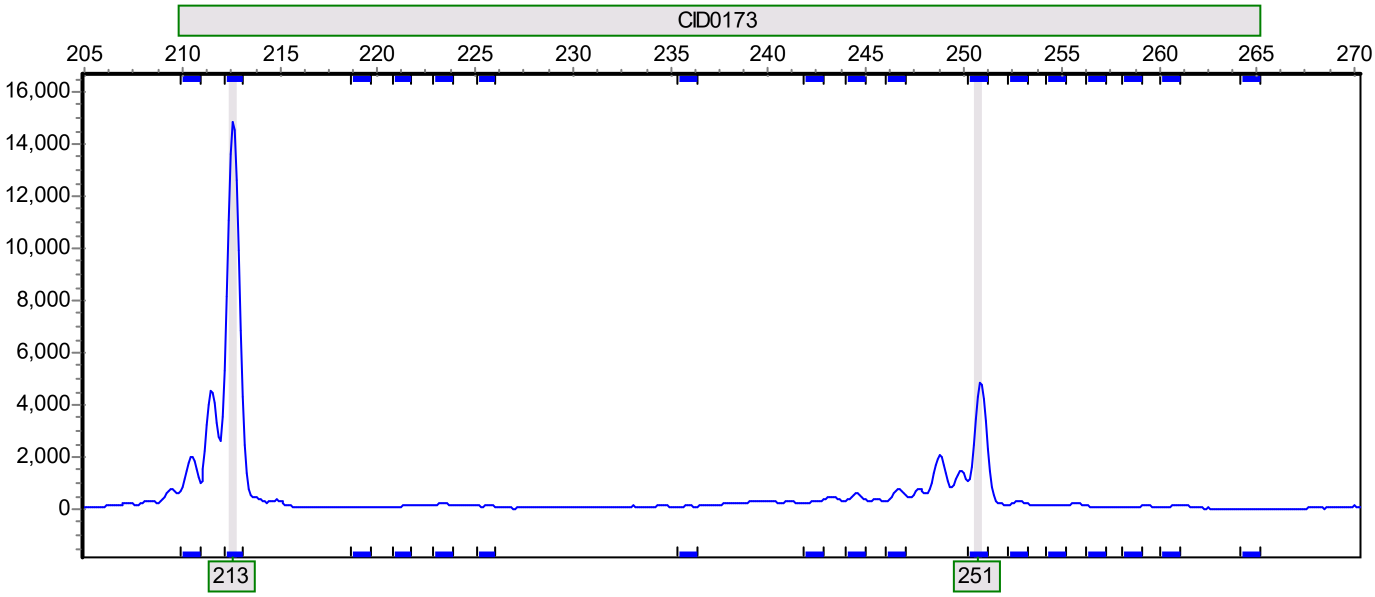

No	Size	Height	Area	Marker	Allele	Difference	Quality	Score	Allele Comments	Sample Comments
1	162.7	22844	142366	EST1573	163	0.20	Pass	500.0	[<Confirmed>]	
2	164.7	17038	106366	EST1573	165	0.10	Pass	500.0	[<Confirmed>]	
3	211.5	4509	29894	CID0173	212	0.10	Undetermined	500.0	[<Deleted>]	
4	212.6	14837	101106	CID0173	213	0.10	Pass	500.0	[<Confirmed>]	
5	250.8	4831	33037	CID0173	251	0.00	Pass	500.0	[<Confirmed>]	

Sample 184: CID0173_EST1573_KC89_P15.fsa Run date and time: 09/21/2024 - 03:27:32 -> 09/21/2024 - 03:55:03

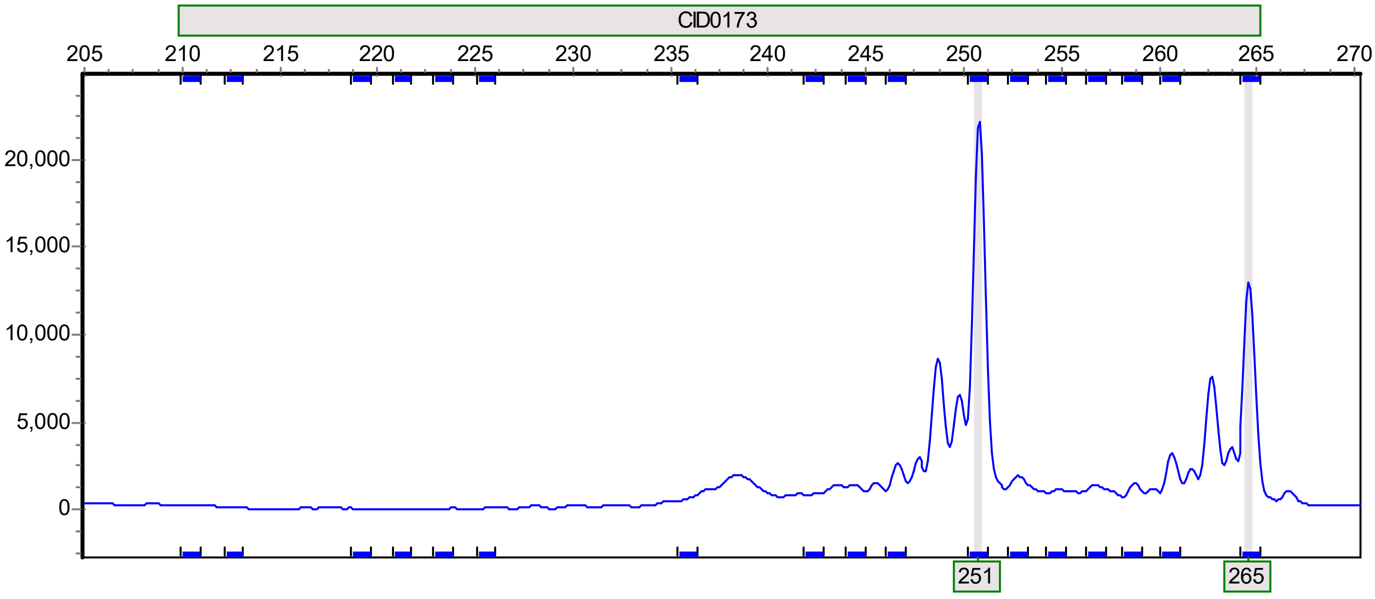

No	Size	Height	Area	Marker	Allele	Difference	Quality	Score	Allele Comments	Sample Comments
1	162.8	43288	300856	EST1573	163	0.10	Pass	500.0	[<SAT (Repaired)><Confirmed>]	
2	189.9	16341	110995	EST1573	190	0.10	Pass	500.0	[<Confirmed>]	
3	250.8	22156	155930	CID0173	251	0.00	Pass	500.0	[<Confirmed>]	
4	264.6	12971	90718	CID0173	265	0.10	Pass	500.0	[<Confirmed>]	

Allele Report

Sample 185: CID0173_EST1573_KC8_M19.fsa Run date and time: 09/21/2024 - 03:00:19 -> 09/21/2024 - 03:27:31

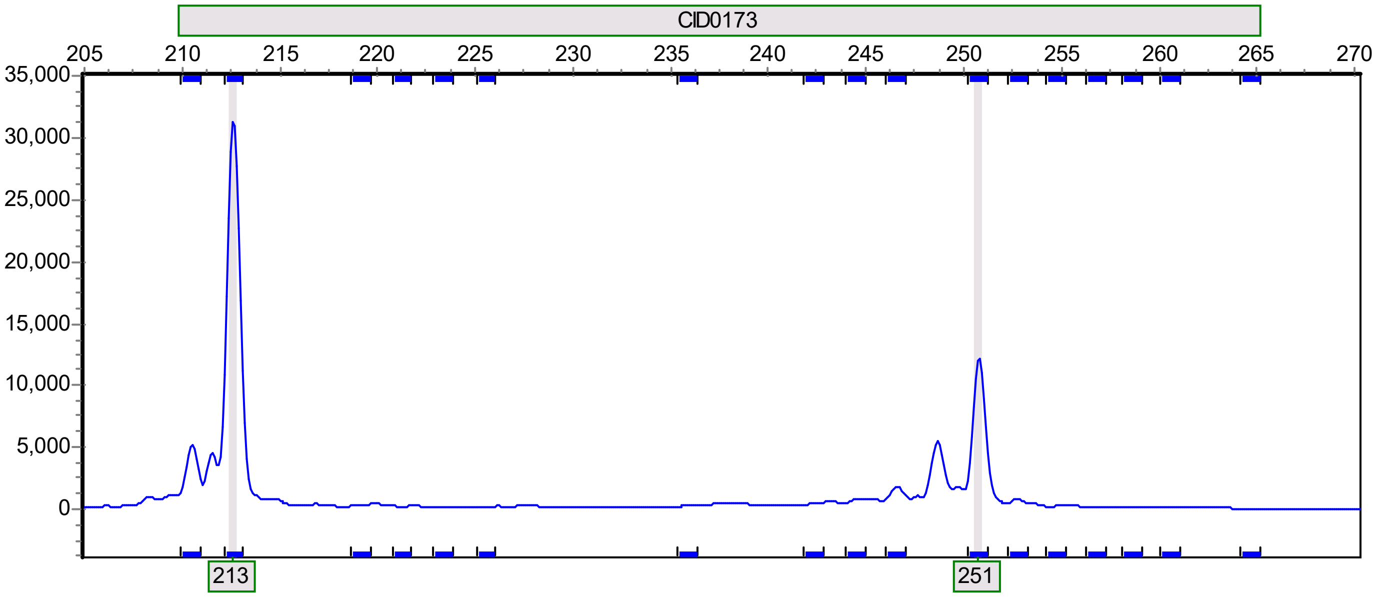

No	Size	Height	Area	Marker	Allele	Difference	Quality	Score	Allele Comments	Sample Comments
1	170.6	31519	216917	EST1573	171	0.00	Pass	500.0	[<Confirmed>]	
2	181.9	9054	62105	EST1573	182	0.10	Pass	500.0	[<Confirmed><Inserted>]	
3	212.6	31338	230495	CID0173	213	0.10	Pass	500.0	[<Confirmed>]	
4	250.8	12187	87402	CID0173	251	0.00	Pass	500.0	[<Confirmed>]	

Sample 186: CID0173_EST1573_KC90_B17.fsa Run date and time: 09/21/2024 - 03:27:32 -> 09/21/2024 - 03:55:03

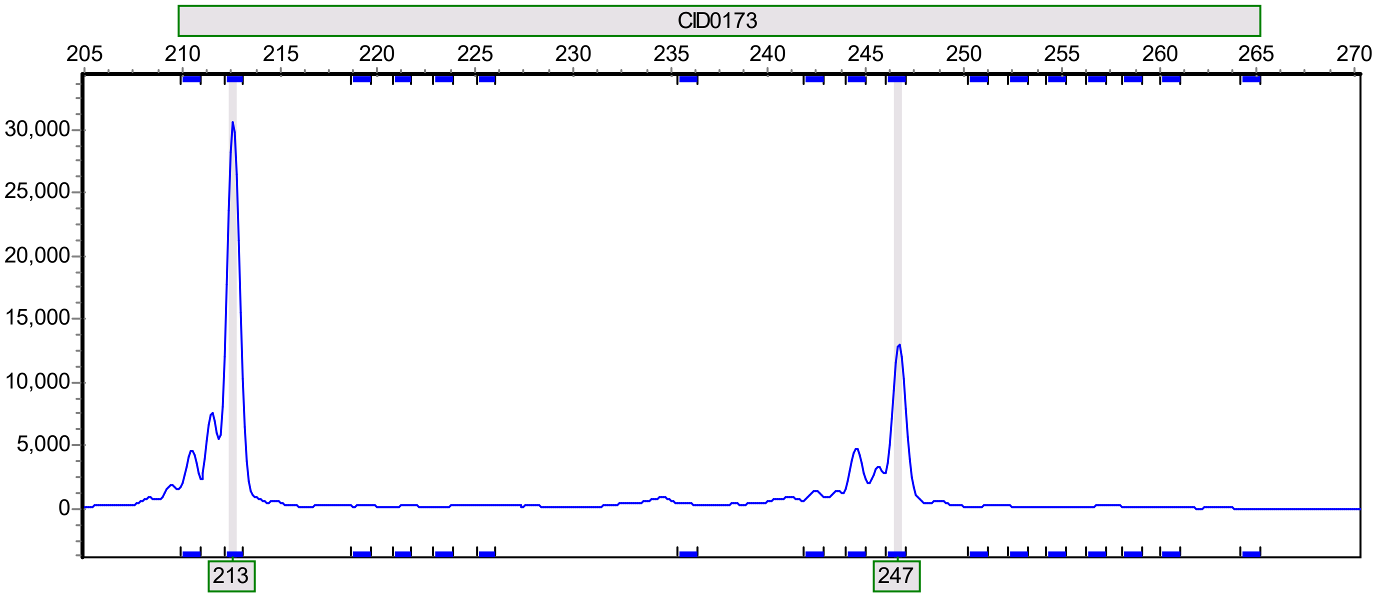

No	Size	Height	Area	Marker	Allele	Difference	Quality	Score	Allele Comments	Sample Comments
1	162.9	28171	211633	EST1573	163	0.00	Pass	500.0	[<SAT (Repaired)><Confirmed>]	
2	181.9	8314	58213	EST1573	182	0.10	Pass	500.0	[<Confirmed><Inserted>]	
3	212.6	30618	230241	CID0173	213	0.10	Pass	500.0	[<Confirmed>]	
4	246.7	13057	103499	CID0173	247	0.10	Pass	500.0	[<Confirmed>]	

Allele Report

Sample 187: CID0173_EST1573_KC91_D17.fsa Run date and time: 09/21/2024 - 03:27:32 -> 09/21/2024 - 03:55:03

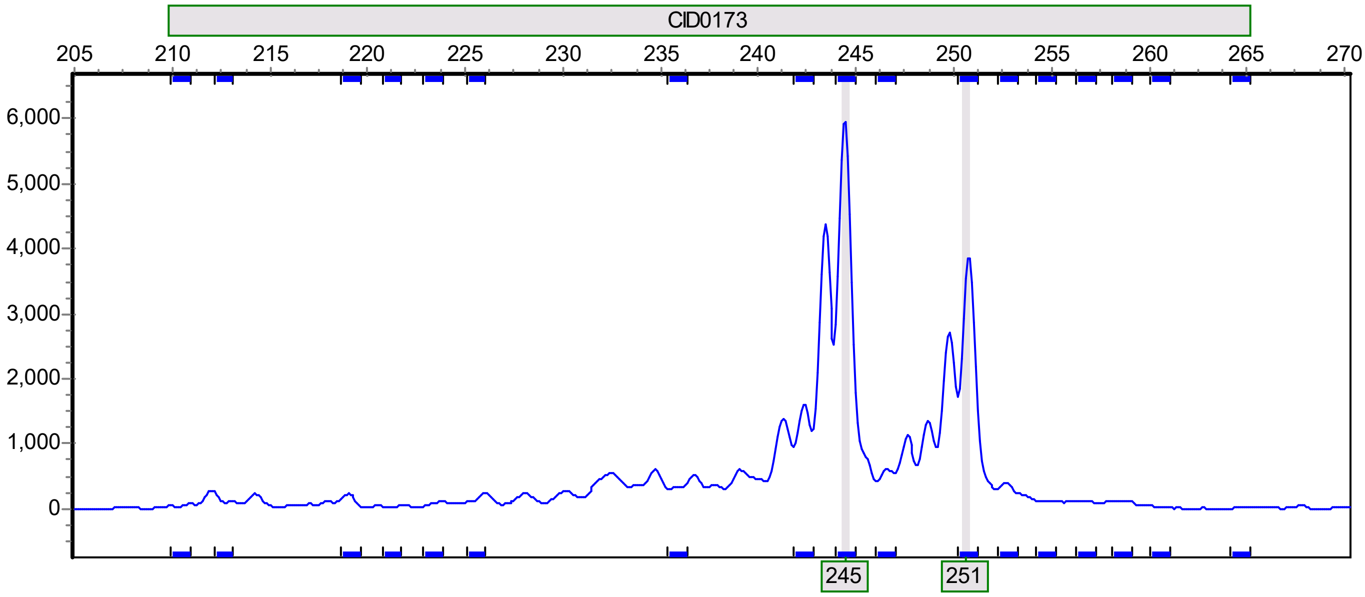

No	Size	Height	Area	Marker	Allele	Difference	Quality	Score	Allele Comments	Sample Comments
1	162.9	30839	198809	EST1573	163	0.00	Pass	500.0	[<Confirmed>]	
2	164.8	25798	167986	EST1573	165	0.00	Pass	500.0	[<Confirmed>]	
3	244.5	5949	45677	CID0173	245	0.00	Pass	500.0	[<Confirmed>]	
4	249.8	2702	19001	CID0173	250	0.00	Undetermined	286.1	[<Deleted>]	
5	250.7	3864	29665	CID0173	251	0.10	Pass	500.0	[<Confirmed>]	

Sample 188: CID0173_EST1573_KC92_F17.fsa Run date and time: 09/21/2024 - 03:27:32 -> 09/21/2024 - 03:55:03

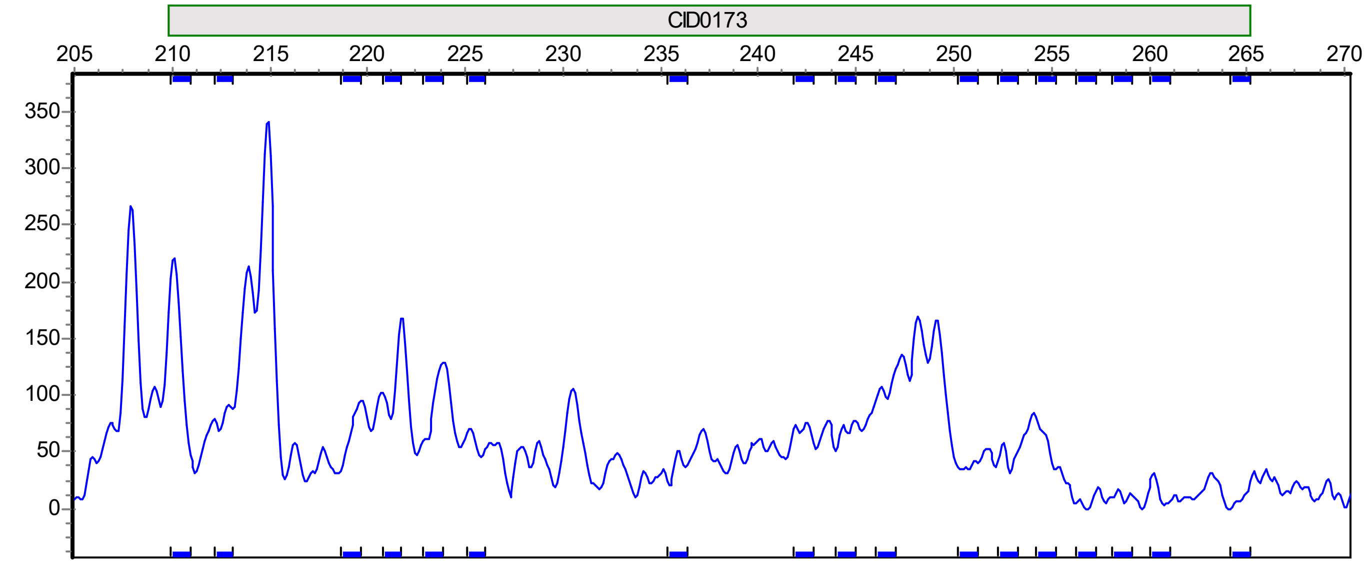

No	Size	Height	Area	Marker	Allele	Difference	Quality	Score	Allele Comments	Sample Comments
1	167.7	351	2905	EST1573	168	0.20	Pass	11.3	[<Deleted>]	
2	174.3	976	7590	EST1573	174	0.10	Pass	94.4	[<Deleted>]	
3	207.9	267	2110	CID0173	208	0.50	Pass	10.4	[<Deleted>]	

Allele Report

Sample 189: CID0173_EST1573_KC93_H17.fsa Run date and time: 09/21/2024 - 03:27:32 -> 09/21/2024 - 03:55:03

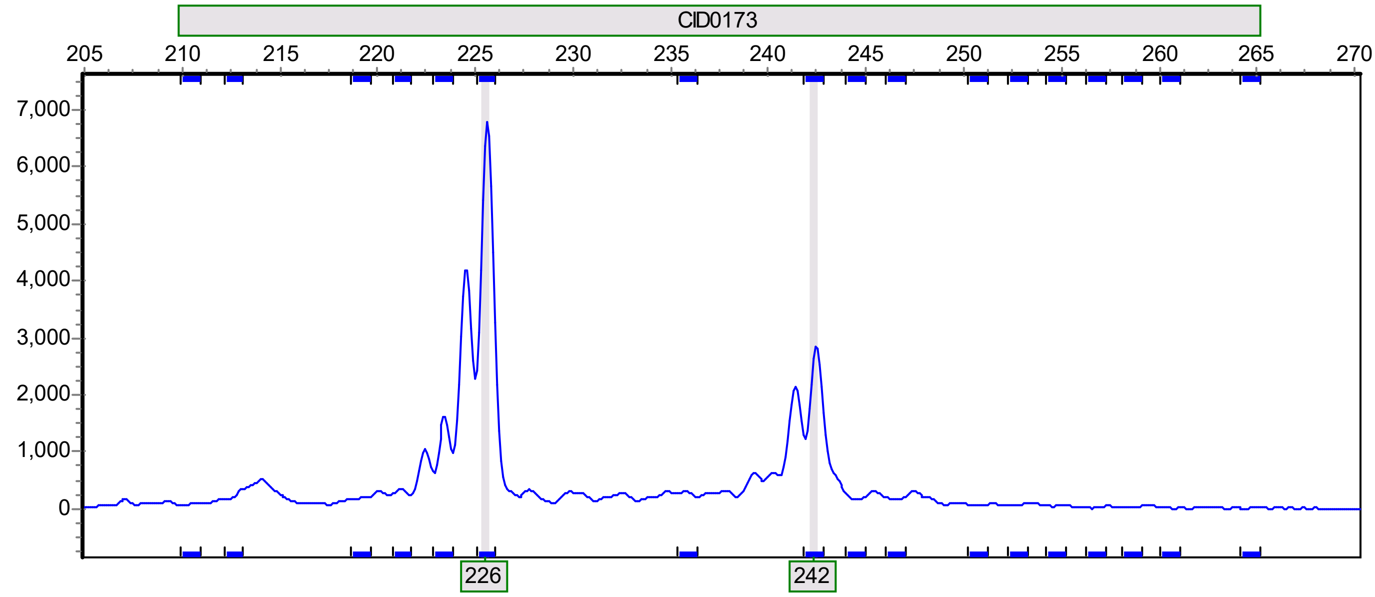

No	Size	Height	Area	Marker	Allele	Difference	Quality	Score	Allele Comments	Sample Comments
1	170.6	26941	169885	EST1573	171	0.00	Pass	500.0	[<Confirmed>]	
2	174.3	12724	82651	EST1573	174	0.10	Pass	500.0	[<Confirmed>]	
3	225.6	6794	48450	CID0173	226	0.00	Pass	500.0	[<Confirmed>]	
4	242.4	2831	22074	CID0173	242	0.00	Pass	382.9	[<Confirmed>]	

Sample 190: CID0173_EST1573_KC94_J17.fsa Run date and time: 09/21/2024 - 03:27:32 -> 09/21/2024 - 03:55:03

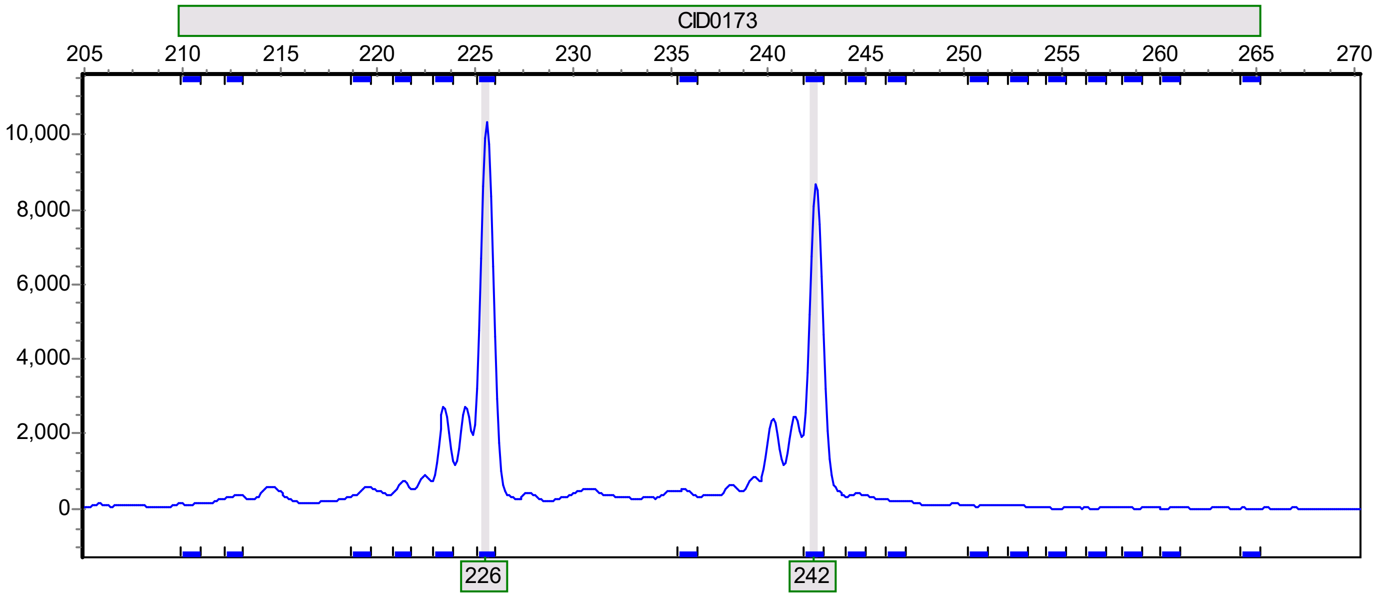

No	Size	Height	Area	Marker	Allele	Difference	Quality	Score	Allele Comments	Sample Comments
1	170.7	21751	144674	EST1573	171	0.10	Pass	500.0	[<Confirmed>]	
2	174.5	16727	109851	EST1573	174	0.10	Pass	500.0	[<Confirmed>]	
3	225.6	10342	75781	CID0173	226	0.00	Pass	500.0	[<Confirmed>]	
4	242.4	8664	66846	CID0173	242	0.00	Pass	500.0	[<Confirmed>]	

Allele Report

Sample 191: CID0173_EST1573_KC95_L17.fsa Run date and time: 09/21/2024 - 03:27:32 -> 09/21/2024 - 03:55:03

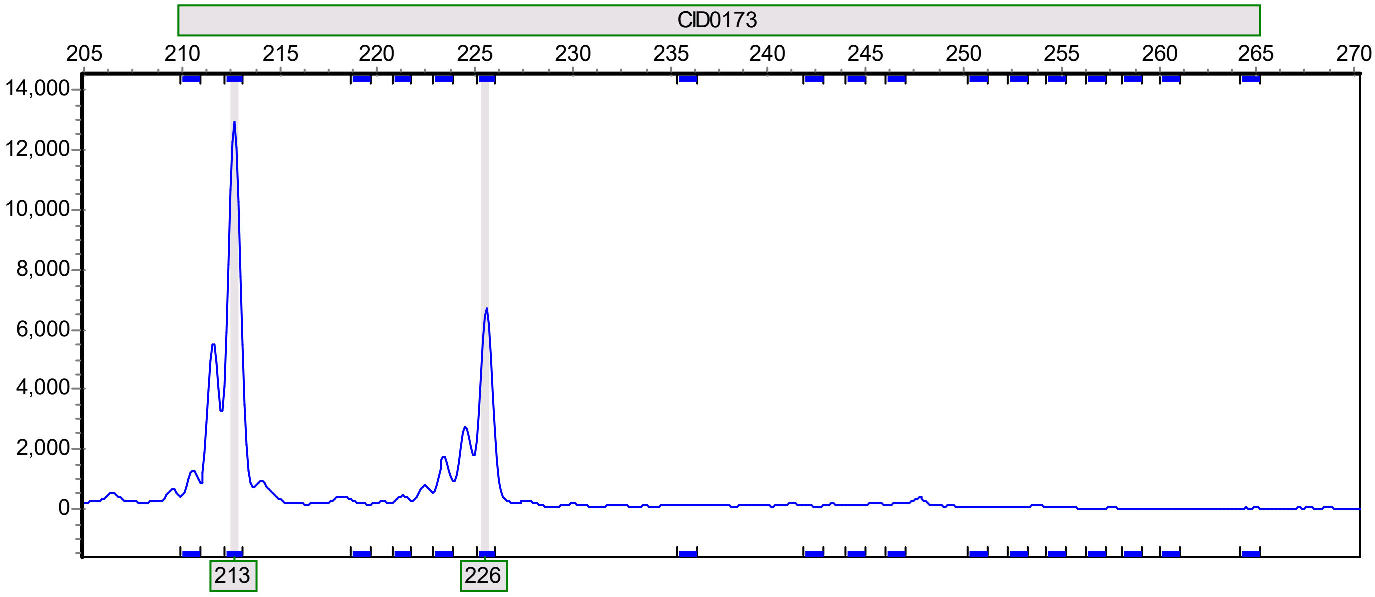

No	Size	Height	Area	Marker	Allele	Difference	Quality	Score	Allele Comments	Sample Comments
1	176.6	21571	145648	EST1573	176	0.20	Pass	500.0	[<Confirmed>]	
2	182.0	5029	34306	EST1573	182	0.00	Pass	500.0	[<Confirmed><Inserted>]	
3	211.6	5516	36682	CID0173	212	0.00	Undetermined	500.0	[<Deleted>]	
4	212.7	12954	93188	CID0173	213	0.00	Pass	500.0	[<Confirmed>]	
5	225.6	6700	47596	CID0173	226	0.00	Pass	500.0	[<Confirmed>]	

Sample 192: CID0173_EST1573_KC96_N17.fsa Run date and time: 09/21/2024 - 03:27:32 -> 09/21/2024 - 03:55:03

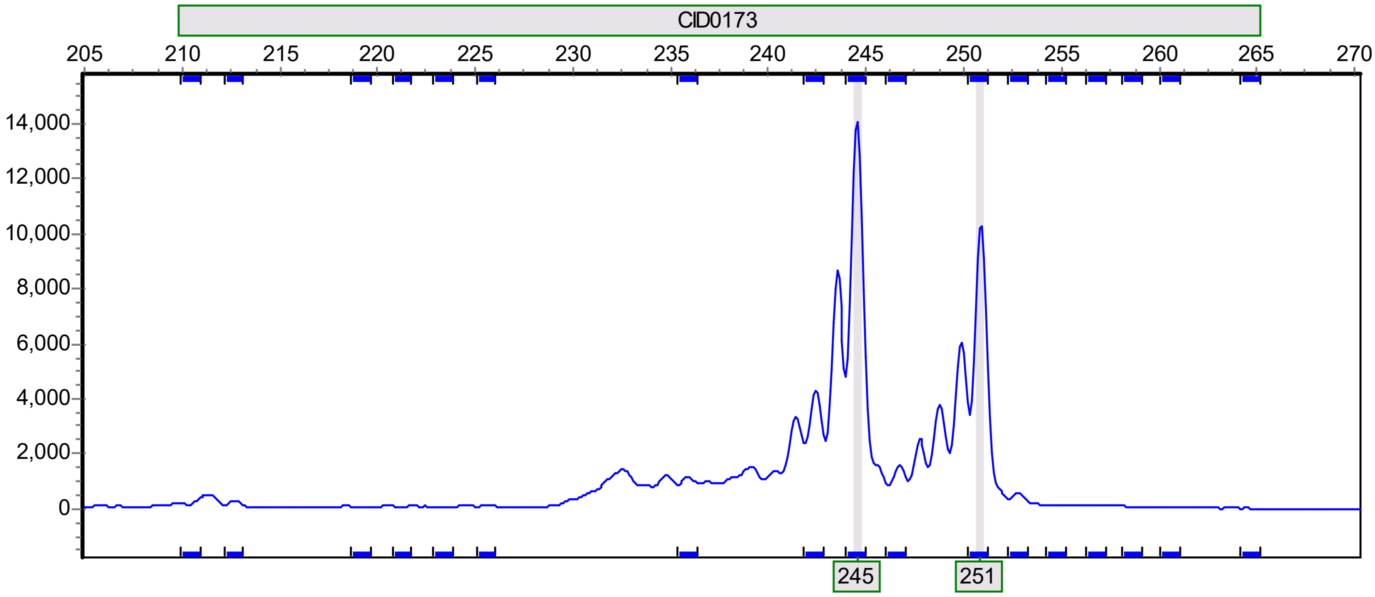

No	Size	Height	Area	Marker	Allele	Difference	Quality	Score	Allele Comments	Sample Comments
1	164.8	31432	214033	EST1573	165	0.00	Pass	500.0	[<Confirmed>]	
2	172.5	31542	203353	EST1573	173	0.00	Pass	500.0	[<Confirmed>]	
3	244.6	14057	103302	CID0173	245	0.10	Pass	500.0	[<Confirmed>]	
4	249.9	6043	40660	CID0173	250	0.10	Undetermined	500.0	[<Deleted>]	
5	250.9	10263	70327	CID0173	251	0.10	Pass	500.0	[<Confirmed>]	

Allele Report

Sample 193: CID0173_EST1573_KC97_P17.fsa Run date and time: 09/21/2024 - 03:27:32 -> 09/21/2024 - 03:55:03

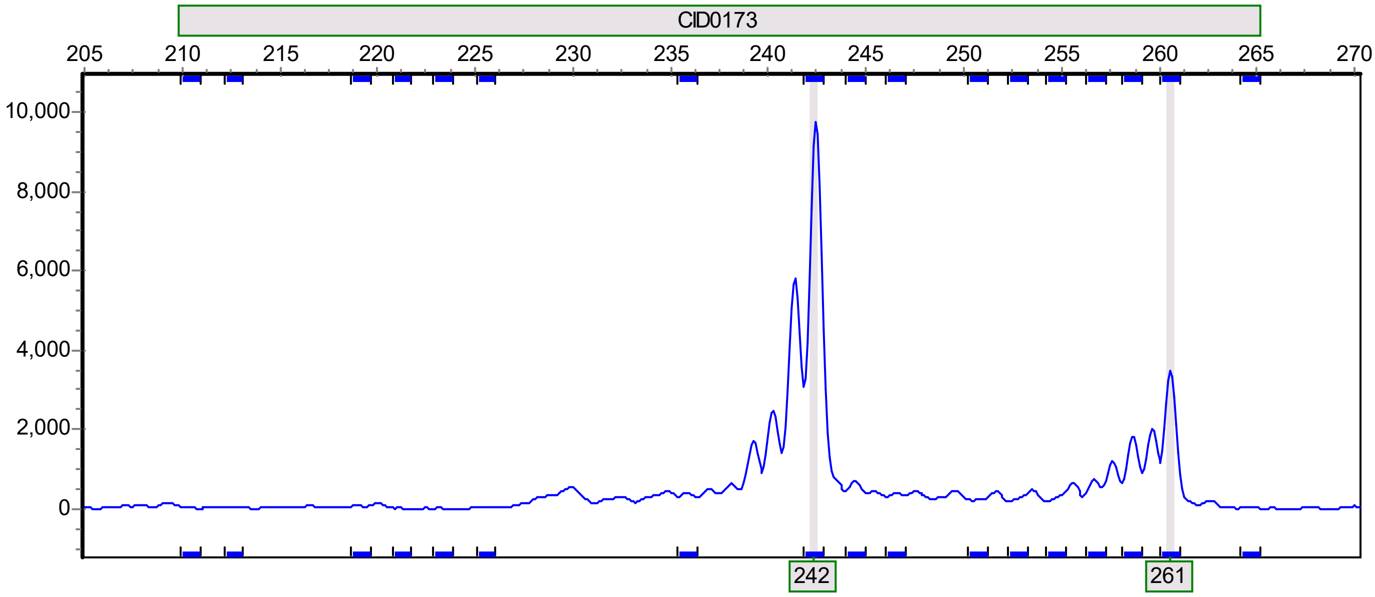

No	Size	Height	Area	Marker	Allele	Difference	Quality	Score	Allele Comments	Sample Comments
1	163.0	28066	199902	EST1573	163	0.10	Pass	500.0	[<SAT (Repaired)><Confirmed>]	
2	174.3	17807	112164	EST1573	174	0.10	Pass	500.0	[<Confirmed>]	
3	242.4	9764	68734	CID0173	242	0.00	Pass	500.0	[<Confirmed>]	
4	260.6	3483	23333	CID0173	261	0.00	Pass	500.0	[<Confirmed>]	

Sample 194: CID0173_EST1573_KC98_B19.fsa Run date and time: 09/21/2024 - 03:27:32 -> 09/21/2024 - 03:55:03

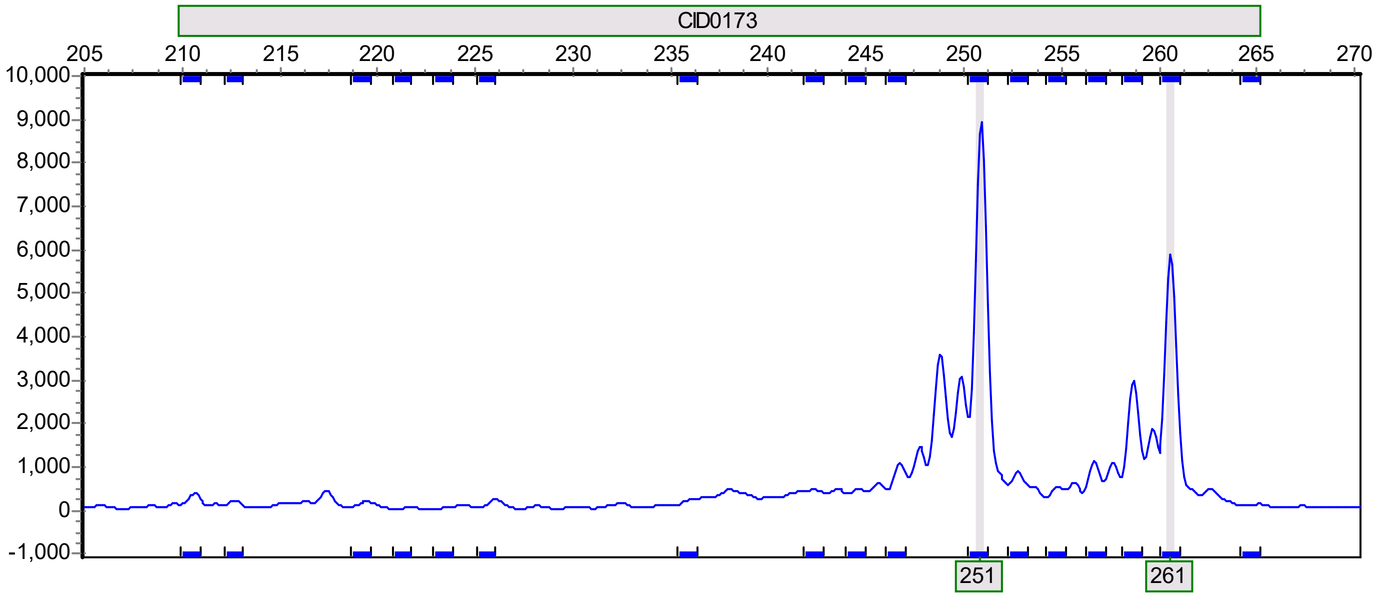

No	Size	Height	Area	Marker	Allele	Difference	Quality	Score	Allele Comments	Sample Comments
1	174.4	20108	132049	EST1573	174	0.00	Pass	500.0	[<Confirmed>]	
2	181.9	9407	60856	EST1573	182	0.10	Pass	500.0	[<Confirmed>]	
3	249.9	3064	22118	CID0173	250	0.10	Undetermined	281.0	[<Deleted>]	
4	250.9	8944	59887	CID0173	251	0.10	Pass	500.0	[<Confirmed>]	
5	260.6	5897	40019	CID0173	261	0.00	Pass	500.0	[<Confirmed>]	

Sample 195: CID0173_EST1573_KC99_D19.fsa Run date and time: 09/21/2024 - 03:27:32 -> 09/21/2024 - 03:55:03

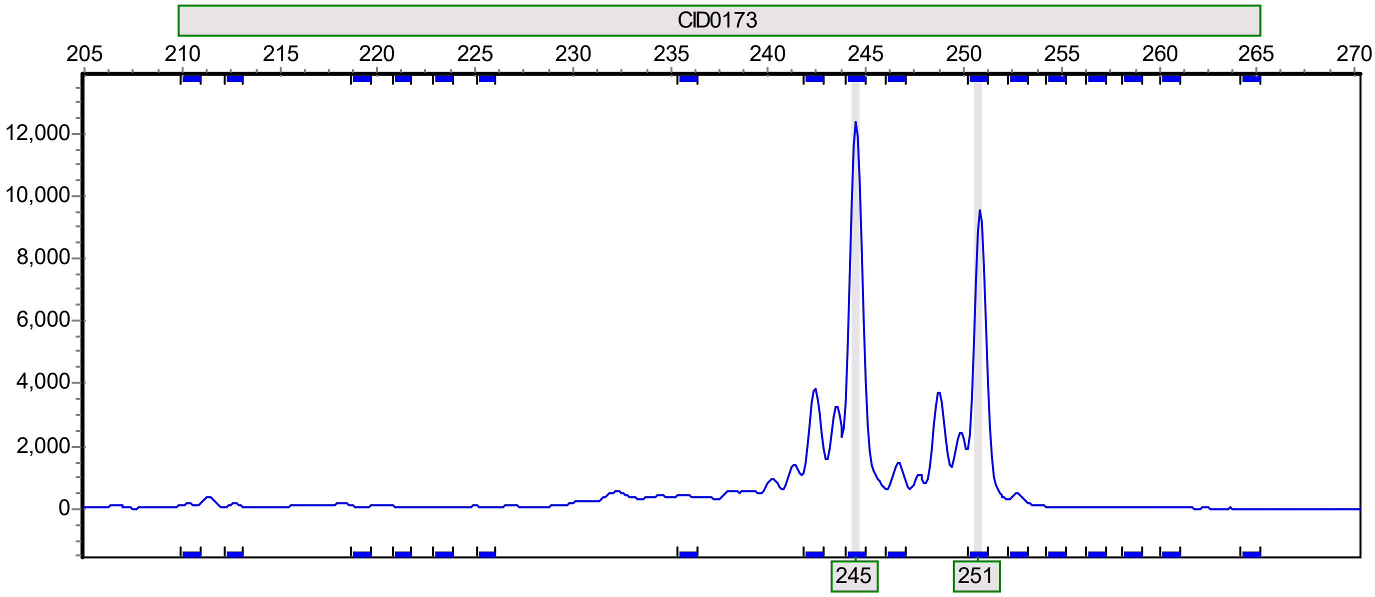

No	Size	Height	Area	Marker	Allele	Difference	Quality	Score	Allele Comments	Sample Comments
1	162.9	31123	203114	EST1573	163	0.00	Pass	500.0	[<Confirmed>]	
2	164.9	26247	165274	EST1573	165	0.10	Pass	500.0	[<Confirmed>]	
3	244.5	12360	92197	CID0173	245	0.00	Pass	500.0	[<Confirmed>]	
4	250.8	9540	67711	CID0173	251	0.00	Pass	500.0	[<Confirmed>]	

Sample 196: CID0173_EST1573_KC9_O19.fsa Run date and time: 09/21/2024 - 03:00:19 -> 09/21/2024 - 03:27:31

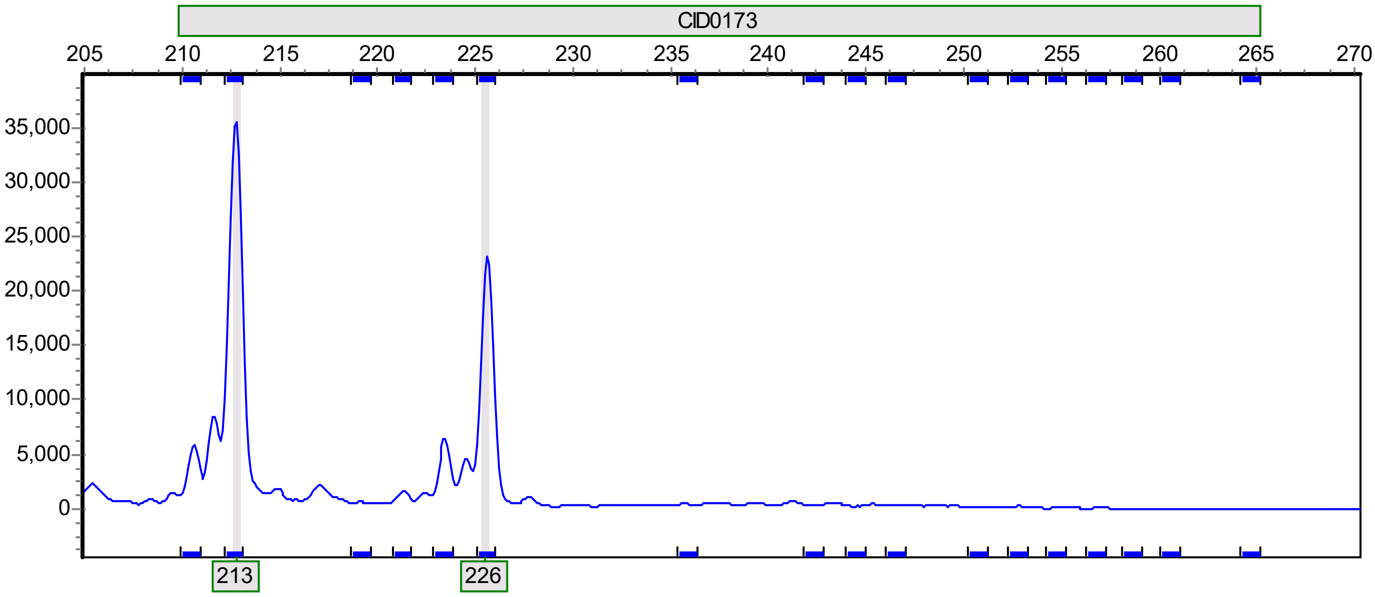

No	Size	Height	Area	Marker	Allele	Difference	Quality	Score	Allele Comments	Sample Comments
1	174.3	28419	186286	EST1573	174	0.10	Pass	500.0	[<Confirmed>]	
2	182.0	11423	76613	EST1573	182	0.00	Pass	500.0	[<Confirmed>]	
3	212.8	35490	274459	CID0173	213	0.10	Pass	500.0	[<SAT (Repaired)><Confirmed>]	
4	225.6	23175	165369	CID0173	226	0.00	Pass	500.0	[<Confirmed>]	
