# Supplementary material for: Assessment of Germplasm Improvement in Three Farmed Grass Carp Populations Based on Genetic Variability
Source: Biology (Basel). 2025 Feb 25;14(3):230. doi: 10.3390/biology14030230 (PMC11939604; doi:10.3390/biology14030230)
Supplement: Supplementary file 1 [file biology-14-00230-s001.zip › TableS1-S5.pdf]

Table S1 The primers information used in this study

Primers	Repeat motif	Sequence of Primers	annealing temperature (℃)	Genbank ID
Ci03	(CA)17	F:CATACTTGTTCCACAGCCTCTC<br>R:CATTCCTTCACCGTTGTATCAT	60	EF408894
CID0004	(TG)18	F: ATCCCCTCTCAATTGACTCACAGTT<br>R:GCTGGCATCTATTTTGAATTCTTATT<br>G	53	FJ227904
Ci398	(CA)11	F: GTTAAGTTTAGGTATTGGGTAG<br>R: CAGCAGGAAATGTAGATGG	48	JX847640
CID0012	(TG)15	F:FAM-ACAGTGCTAAACCTGCCAGTC<br>AGTG<br>R: ACAGCAGCACCAGTGGACATCAT	55	FJ227909
CID1533	(GA)44	F: FAM-GCCCGCTTGCCATTCTCAGT<br>R: GTCGACGATCTGTCCATCAGTGTG	55	FJ883458
Cid0036	(CA)26	F:FAM-CCAGGGGCAAAACACAGACA<br>ATACTC<br>R:AGGAAGCCATTCTTTGGATCTCATT<br>AG	57	FJ227923
HLJC126	(CT)20	F: TTCTCTGCCCTGTTGTGTTG<br>R: AAATTGAGGCCCTGAGGACT	55	FJ213575
CID0002	(AC)23	F: FAM-GCAGGCTGCTGAAGAATA<br>R: AACTTACTGACCCCAAACC	56	FJ227902
EST1573	(AC)10(AC)4	F: GTCATACTATCAACCAGCAA<br>R: GGAACATCCACCTGAACT	50	JG731382
CID0173	(TG)7	F: FAM-CGTGCCGACTTTCCAGACTA<br>R: CCAATTCCTTCACCTTGCTAATAC	55	FJ883248
CID0283	(CT)14	F: CATATAATTACTCAGGTGTGTG<br>R: ACGAAAATTGTATCCGTCTTTGA	49	FJ883284
CID0382	(AC)17	F:FAM-CCAGCTTAGGCTAACTGTTTGT<br>T<br>R: CATGCTGCCAGTCACTCTTATTA	55	FJ883312
CID0474	(GT)12	F: FAM-GGGCGCTGTCTTGAAACAA<br>R: TTGGGAATGAGCTGCTAACAAG	55	FJ883334
CID0347	(CA)12	F: GTAACCCAACCGCTGAACCA<br>R: TTCCCGTCTGAACATCGTGAT	54	FJ883304
CID0869	(GT)8	F:HEX-GCACGAGCCTCTAAAGTCCGT<br>CAG R: CATAGCGCCACCGCGAGTTCA	55	FJ883407
CID1512	(CT)22	F: FAM-GCGCGTTGTTTGGTGTGC<br>R: GCAGGACTAGGATCGTGGTCATT	55	FJ883438

GC39	(AC)15(AT)5	F: TCCACCAGCATCACATAGT<br>R: TTTTGCTATVVTCACTTACAT	50	CR548626
HLJC107	(CT)21	F: GCAAGCTGCATTCACTCTGA<br>R: TGGAGAAAAGGGCTGTAGGA	55	FJ213566
CID0017	(CA)20	F: FAM-CTGGCCCCGGAGGAGACG<br>R: AGCAGCGACCGCAGAAGATGAT	58	CP108482
CID0909	(CA)13	F: HEX-CATGTAGTCCACCGCCTGATGAT<br>R: GAAGGGGCAGCTTGAAATCCA	55	FJ883413
CID0042	(GT)8	F:FAM-CACGGATAAATCCAGATACACTCA<br>R: GTCCGGCGACCAGACTCAC	55	FJ883183
Ci240	(TG)10	F: AGCCTTTGTTTGGCATTA<br>R: CAGCAGGGAGTCCACTTT	48	JX847631

Table S2 Summary of genetic diversity parameters of all grass carp based on 22 microsatellite loci.

Locus	*Na*	*N*	*Ho*	*He*	*PIC*	*Fis*
Ci03	14	194	0.49	0.791	0.761	0.237
CID0004	21	196	0.77	0.859	0.846	0.0568
Ci398	15	196	0.653	0.85	0.83	0.1315
CID0012	16	196	0.673	0.797	0.771	0.0681
CID1533	29	195	0.779	0.935	0.929	0.0905
Cid0036	16	196	0.73	0.888	0.875	0.0973
HLJC126	12	192	0.729	0.843	0.823	0.0743
CID0002	22	194	0.753	0.902	0.892	0.0912
EST1573	12	196	0.633	0.753	0.721	0.0879
CID0173	20	196	0.704	0.86	0.844	0.1021
CID0283	13	196	0.709	0.844	0.827	0.097
CID0382	14	196	0.74	0.878	0.863	0.0813
CID0474	13	196	0.577	0.804	0.774	0.1608
CID0347	9	196	0.52	0.659	0.626	0.0922
CID0869	15	196	0.709	0.794	0.766	0.0511
CID1512	25	196	0.77	0.911	0.902	0.0794
GC39	16	196	0.704	0.84	0.82	0.0844
HLJC107	14	196	0.694	0.866	0.849	0.1069
CID0017	16	196	0.724	0.871	0.855	0.094
CID0909	18	196	0.74	0.9	0.889	0.0949
CID0042	22	196	0.781	0.911	0.902	0.0756
Ci240	21	196	0.699	0.823	0.804	0.0761

Table S3 Summary of genetic diversity parameters of common grass carp based on 22 microsatellite loci.

Locus	*N*	*Na*	*Ho*	*He*	*PIC*	*Fis*
Ci03	32	5	0.344	0.492	0.425	0.2092
CID0004	32	10	0.938	0.833	0.796	-0.0721
Ci398	32	9	0.906	0.825	0.786	-0.0583
CID0012	32	7	0.781	0.779	0.733	-0.0196
CID1533	32	18	1	0.898	0.873	-0.0643
Cid0036	32	10	0.906	0.799	0.757	-0.0758
HLJC126	32	8	0.781	0.856	0.823	0.0404
CID0002	32	12	0.969	0.851	0.818	-0.0799
EST1573	32	6	0.719	0.661	0.61	-0.0891
CID0173	32	11	0.719	0.759	0.718	0.0299
CID0283	32	11	0.938	0.858	0.825	-0.058
CID0382	32	9	0.844	0.782	0.74	-0.0551
CID0474	32	5	0.875	0.625	0.541	-0.2035
CID0347	32	7	0.813	0.599	0.537	-0.1939
CID0869	32	7	0.875	0.604	0.516	-0.2035
CID1512	32	10	0.938	0.836	0.799	-0.0702
GC39	32	9	0.906	0.821	0.783	-0.0717
HLJC107	32	6	0.688	0.638	0.561	-0.051
CID0017	32	6	0.531	0.616	0.565	0.0698
CID0909	32	8	0.719	0.748	0.69	0.0135
CID0042	32	12	0.969	0.843	0.808	-0.0791
Ci240	32	7	0.906	0.739	0.681	-0.1185

Table S4 Summary of genetic diversity parameters of gynogenetic grass carp based on 22 microsatellite loci.

Locus	*N*	*Na*	*Ho*	*He*	*PIC*	*Fis*
Ci03	34	8	0	0.534	0.511	0.9996
CID0004	34	5	0	0.402	0.374	0.9959
Ci398	34	5	0	0.611	0.564	0.9999
CID0012	34	5	0	0.692	0.629	1
CID1533	34	10	0	0.739	0.705	1
Cid0036	34	7	0.029	0.786	0.742	0.9264
HLJC126	34	6	0.059	0.576	0.54	0.8149
CID0002	34	6	0	0.664	0.615	1
EST1573	34	3	0	0.514	0.405	0.9996
CID0173	34	6	0.029	0.644	0.6	0.9126
CID0283	34	3	0.059	0.42	0.364	0.7481
CID0382	34	5	0	0.701	0.647	1
CID0474	34	4	0	0.637	0.573	1
CID0347	34	6	0	0.62	0.58	1
CID0869	34	6	0.176	0.583	0.54	0.5698

CID1512	34	9	0	0.753	0.716	1
GC39	34	5	0	0.522	0.487	0.9995
HLJC107	34	7	0	0.723	0.673	1
CID0017	34	4	0	0.507	0.46	0.9994
CID0909	34	7	0	0.702	0.661	1
CID0042	34	6	0.118	0.678	0.63	0.7031
Ci240	34	6	0.088	0.672	0.627	0.7662

Table S5 Summary of genetic diversity parameters of disease-resistant grass carp based on 22 microsatellite loci.

Locus	*N*	*K*	*Ho*	*He*	*PIC*	*Fis*
Ci03	128	11	0.656	0.799	0.768	0.093
CID0004	130	19	0.931	0.885	0.872	-0.0321
Ci398	130	13	0.762	0.859	0.839	0.0552
CID0012	130	14	0.823	0.756	0.723	-0.0613
CID1533	129	27	0.93	0.936	0.929	0.0014
Cid0036	130	14	0.869	0.883	0.868	0.0057
HLJC126	126	12	0.897	0.847	0.826	-0.0348
CID0002	128	19	0.898	0.882	0.868	-0.0114
EST1573	130	11	0.777	0.783	0.756	0.0012
CID0173	130	17	0.877	0.874	0.86	-0.0072
CID0283	130	11	0.823	0.852	0.832	0.0194
CID0382	130	13	0.908	0.87	0.854	-0.0244
CID0474	130	13	0.654	0.761	0.727	0.0792
CID0347	130	9	0.585	0.55	0.531	-0.0569
CID0869	130	14	0.808	0.811	0.785	-0.0087
CID1512	130	21	0.931	0.888	0.874	-0.0266
GC39	130	15	0.838	0.822	0.803	-0.0241
HLJC107	130	14	0.877	0.88	0.864	-0.0019
CID0017	130	16	0.962	0.88	0.864	-0.0478
CID0909	130	16	0.938	0.901	0.888	-0.0233
CID0042	130	19	0.908	0.907	0.895	-0.0027
Ci240	130	20	0.808	0.81	0.793	-0.0036
