# Supplementary figures and images for: Assessment of Germplasm Improvement in Three Farmed Grass Carp Populations Based on Genetic Variability
Source: Biology (Basel). 2025 Feb 25;14(3):230. doi: 10.3390/biology14030230 (PMC11939604; doi:10.3390/biology14030230)

0.6

0.4

0.2

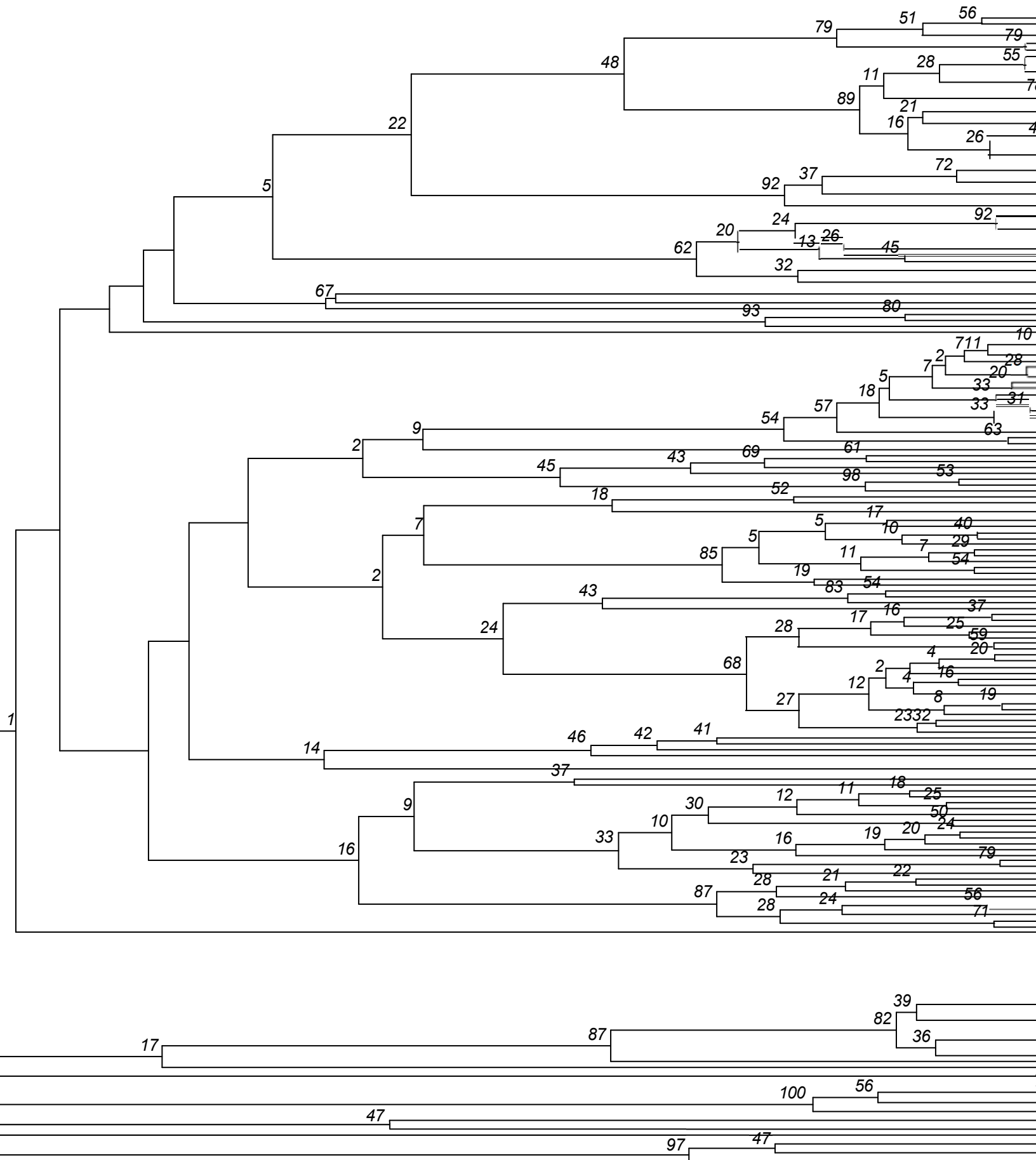

Supplement: Supplementary file 1 [file biology-14-00230-s001.zip › FigureS1.pdf]
